# Evolution of pollen morphology in Loranthaceae

**DOI:** 10.1080/00173134.2016.1261939

**Published:** 2017-02-20

**Authors:** Friðgeir Grímsson, Guido W. Grimm, Reinhard Zetter

**Affiliations:** ^a^ Department of Palaeontology, University of Vienna, Vienna, Austria

**Keywords:** Santalales, genus phylogeny, data gaps, diagnostic pollen

## Abstract

Earlier studies indicate a strong correlation of pollen morphology and ultrastructure with taxonomy in Loranthaceae. Using high-resolution light microscopy and scanning electron microscopy imaging of the same pollen grains, we document pollen types of 35 genera including 15 studied for the first time. Using a molecular phylogenetic framework based on currently available sequence data with good genus-coverage, we reconstruct trends in the evolution of Loranthaceae pollen and pinpoint traits of high diagnostic value, partly confirming earlier intuitive hypotheses based on morphological observations. We find that pollen morphology in Loranthaceae is strongly linked to phylogenetic relationships. Some pollen types are diagnostic for discrete genera or evolutionary lineages, opening the avenue to recruit dispersed fossil pollen as age constraints for dated phylogenies and as independent data for testing biogeographic scenarios; so far based exclusively on modern-day data. Correspondences and discrepancies between palynological and molecular data and current taxonomic/systematic concepts are identified and suggestions made for future palynological and molecular investigations of Loranthaceae.

The Loranthaceae (order Santalales) is a moderately large family containing about 76 genera and approximately 1076 species in five tribes (Nickrent 1997 onwards; Nickrent et al. 2010). The family has a wide geographical distribution, occurring in tropical to temperate climates of Central and South America, Europe, Africa, the Middle East, across Asia and Australasia. Of the currently 76 genera comprising the Loranthaceae, three are root parasites and the rest are aerial branch parasites. A recent study using molecular data clarified some phylogenetic relationships within the family (Vidal-Russell & Nickrent 2008b); that study forms – to some degree – the basis of a revised systematic framework (Nickrent et al. 2010; summarised in Table I). The root parasite *Nuytsia* (monotypic Nuytsieae) has been suggested to represent the first diverging lineage within the Loranthaceae, forming a ‘basal’ grade with the Gaiadendreae comprising the only other two root parasites, *Atkinsonia* and *Gaiadendron* (Su et al. 2015). The remaining aerial parasitic genera of the family are considered to be monophyletic (Vidal-Russell & Nickrent 2008b; Nickrent et al. 2010; Su et al. 2015); the corresponding clade in molecular phylograms is, however, poorly supported by bootstrapping (BS < 60) and moderately to high using Bayesian probabilities (PP > 0.8; see online Supplemental Material [OSM] File S1). Nickrent et al. (2010) recognised one possibly para- or polyphyletic tribe (Psittacantheae) and two monophyletic tribes (Elytrantheae, type genus not sequenced yet, and Lorantheae) within the aerial parasite clade. Although four of Nickrent et al.’s (2010) 11 Psittacantheae and Lorantheae subtribes do not conform with molecular clades reconstructed by Vidal-Russell and Nickrent (2008b) and Su et al. (2015), they are also not rejected by molecular data with strong support. Many branches in the molecular phylograms produced so far, simply lack high support. Hence, it may be possible that there are competing signals in the molecular data (see File S1), some of which may be in better agreement with morphological groups and that adding more molecular data will eventually confirm some of these groups with sufficient support (anonymous reviewer, personal communication).

**Table I.** Current systematic framework of Loranthaceae (Nickrent et al. 2010).

**Tribe**/subtribe; clade	Genus (number of species)
**Nuytsiae**; sister to all other Loranthaceae	*Nuytsia* (1)
**Gaiadendreae**; forming a ‘basal’ grade	*Atkinsonia* (1), *Gaiadendron* (1)
**Elytrantheae**; Clade A [A] or Clade B [B]	[A]*Alepis* (1), [B]*Amylotheca* (4), *Cyne (6), [B]*Decaisnina* (25), *Elytranthe (7), *Lampas (1), *Lepeostegeres*^a^ (9), [B]*Lepidaria* (14), [B]*Loxanthera* (1),[B]*Lysiana* (8), [B]*Macrosolen* (50), [A]*Peraxilla* (2), *Trilepideae (1)
**Psittacantheae**	
Ligarinae; part of Clade D	*Ligaria* (2), *Tristerix* (13)
Notantherinae; part of Clade D	*Desmaria* (1), *Notanthera* (1)
Psittacanthinae; Clade E, subclade of D	*Aetanthus*^b^ (15), *Cladocolea* (28), *Dendropemon* (33), *Maracanthus (4), *Oryctanthus* (15), *Oryctina (7), *Panamanthus (1), *Passovia* (21), *Phthirusa* (7), *Psittacanthus* (120), *Struthanthus* (86), *Tripodanthus* (30)
Tupeinae; unlabelled clade	*Tupeia* (1)
**Lorantheae**; Clade F (includes three main subclades: Clade G, H, and I/J)	
Amyeminae; Clade I or part of Clade I/J	*Amyema* (93), *Baratranthus* (3), *Benthamina* (1), *Dactyliophora* (3), *Diplatia* (3), *Distrianthes (1), *Helicanthes* (1), *Sogerianthe* (4)
Dendrophthinae; part of Clade J or Clade I/J	*Dendrophthoe* (59), *Helixanthera* (55), *Tolypanthus* (7), *Trithecanthera (5)
Emelianthinae; part of Clade J	*Emelianthe* (2), *Erianthemum* (16), *Globimetula* (13), *Moquinella* (1), *Oliverella* (3), *Phragmanthera* (35), *Spragueanella* (2)
Ileostylinae; Clade H	*Ileostylus* (1), *Muellerina* (5)
Loranthinae; Clade G	*Cecarria* (1), *Loranthus* (9)
Scurrulinae; part of Clade I/J	*Scurrula* (43), *Taxillus* (30)
Tapinanthinae; part of Clade J	*Actinanthella* (2), *Agelanthus* (59), *Bakerella* (16), *Berhautia* (1), *Englerina* (26), *Oedina* (4), *Oncella* (4), *Oncocalyx* (12), *Pedistylis (1), *Plicosepalus* (12), *Septulina* (2), *Socratina* (2), *Tapinanthus* (30), *Vanwykia* (1)

Note: Clades following Vidal-Russell and Nickrent (2008b); genera and species numbers according to Nickrent (1997 onwards). Asterisks denote genera for which no molecular data are available. ^a^ Not included in the tree based on the concatenated matrix; sister to Clade B in the 25S rDNA tree shown by Vidal-Russell and Nickrent (2008b, figure 1); ^b^ Sister to *Psittacanthus* in the cp-tree Vidal-Russell and Nickrent (2008b, figure 2); based on signal from the included *mat*K sequence; not included in the tree based on the concatenated matrix (sequence not used here, see File S2).

The pollen morphology of a number of extant Loranthaceae has been studied in detail using combined light microscopy (LM), scanning electron microscopy (SEM) and transmission electron microscopy (TEM), and revealed characteristic pollen features (Feuer & Kuijt 1978, 1979, 1980, 1985; Roldán & Kuijt 2005; Caires 2012). The micrographs of two studies focussing on Chinese Loranthaceae (Liu & Qiu 1993; Han et al. 2004) are difficult to interpret. The distinct pollen types of (most) Loranthaceae, types that cannot be confused with pollen from other angiosperm families including other Santalales (Balanophoraceae [*s.str*.]: Hansen 1980; Misodendraceae: Feuer 1981; Del Carmen Zamaloa & Fernández 2016; ‘Olacaceae’ [*s.l*.]/Schoepfiaceae: Maguire et al. 1974; Feuer 1978; Santalaceae: Feuer & Kuijt 1982; Feuer et al. 1982) should have high potential to trace modern lineages back in the past. Feuer and Kuijt (1980) noted that pollen types are often genus-specific in the group of large-flowered Neotropical taxa and their putative Australian relatives, including all root-parasitic genera. The same holds for some of the Neotropical small-flowered taxa (Feuer & Kuijt 1985). Based on found similarities and dissimilarities and intra-generic variation in these groups, they established a first hypothetical framework for the evolution of pollen morphs. However, there has been no attempt so far to link pollen morphology to molecular phylogenies and the current systematic framework for the family. Feuer and Kuijt (1980, 1985) based their evolutionary interpretations on the assumption that the small- and large-flowered Neotropical taxa form natural groups, a hypothesis rejected by later molecular phylogenies (Wilson & Calvin 2006; Vidal-Russell & Nickrent 2008b).

Using published accounts and our own scanning-electron imaging of 35 to 36 genera (54 species and 1 sp. indet.) of Loranthaceae, including 15 (37 species) studied here for the first time, we evaluate the correlation of pollen morphology and phylogenetic relationships within Loranthaceae as inferred from molecular sequence data. We find that most pollen types in Loranthaceae can be linked to a single genus or discrete evolutionary lineages (molecular-supported clades, currently recognised tribes and subtribes). We discuss hypotheses about the evolution of Loranthaceae pollen, which can serve as basis for the future revision, description and interpretation of fossil pollen of the family. We note that in the light of resolution issues of current molecular data (File S1), the study of Loranthaceae pollen can assist in the identification of critical or problematic species, which should be covered in any future molecular assessment.

## Material and methods

### Preparation of samples

At the herbarium, depending on the material, a single anther, few anthers or an entire flower was removed under a dissecting microscope (stereoscope) and placed into small sample bags. In the laboratory, a single to few anthers from each sample were placed into drops of acetolysis liquid (nine to one mix of 99% acetic anhydrite and 95–97% sulphuric acid) on microscopic glass slides. The slides were heated over a candle flame for a short time to soften up the anthers, release the pollen grains from anthers, dissolve extra organic material on pollen grain surfaces, rehydrate pollen grains and release their cell contents, and finally, to stain, the pollen grains for LM photography. Selected pollen grains were then transferred into fresh drops of glycerine on new glass slides using a micromanipulator and photographed under LM. Selected LM-photographed grains were transferred to SEM stubs using a micromanipulator and washed with drops of absolute ethanol to remove any remaining glycerine. The stubs were sputter-coated with gold and the pollen grains photographed under a JEOL 6400 SEM.

### Conservation of material

SEM stubs produced under this study are stored in the collection of the Department of Palaeontology, University of Vienna, Austria, under accession numbers IPUW 7513/001–075.

### Molecular analyses

Data on Loranthaceae (in total 711 accessions) were harvested using the NCBI GenBank portal (accessed 3 April 2014) following the procedure outlined in Grimm and Renner (2013): GenBank flatfiles were read-out with gbk2fas (Göker et al. 2009), data aligned using mafft (Katoh et al. 2005; Katoh & Standley 2013) and visual inspections and curation of alignments done using Mesquite v. 2.75 (Maddison & Maddison 2011). The set-up and curation details can be found in File S2.

The complete data (File S3) was filtered for gene regions with broadest taxonomic coverage, providing us with six potential gene regions for analyses: the nuclear-encoded rRNA genes and internal transcribed spacers of the 35S rDNA cistron, (1) the 18S rDNA, (2) the ITS region including the internal transcribed spacers ITS1 and ITS2, interspersed by the 5.8S rDNA, and (3) the 25S rDNA; from the plastome (4) the *mat*K gene, (5) the *trn*L/*trn*LF region including the *trn*L intron, *trn*L 3ʹ exon, *trn*L-*trn*F intergenic spacer, and *trn*F gene, and (6) the *rbc*L gene. The ITS1 and ITS2 can be highly divergent (even unalignable) between main Loranthaceae lineages. Being only interested in inter-generic relationships, we refrained from using the ITS region in further analyses to avoid alignment-and-gap-bias. To mask intrageneric differentiation patterns that may obscure signal from deep divergences and to minimise missing data in the final alignment, we computed strict genus-consensus sequences for 18S, 25S, *mat*K, *trn*L/*trn*LF and *rbc*L data using the programme g2cef (Göker & Grimm 2008). All data files, primary matrix NEXUS files optimised for Mesquite, can be found in the OSM (File S2). Phylogenetic tree inference and bootstrapping relied on RAxML v. 7.2.6 (Stamatakis 2006; Stamatakis et al. 2008; set-up details are provided in File S2).

We performed a full tree inference under the general-time-reversible model allowing for site-specific rate variation and partition-wise optimised substitution rates coupled with a fast bootstrapping, where the number of necessary bootstrap replicates was determined by the extended majority rule consensus bootstop criterion (Pattengale et al. 2009). To test for potential sampling or missing data bias – a number of Loranthaceae genera are only incompletely known for the five gene regions (File S3) – a gene-jackknifing procedure was applied, using a restricted data set with no missing gene region (full results included in the OSM; summarised in File S1). Incompatible splits patterns in the bootstrap samples were investigated using bipartition (bootstrap support) networks, a special form of consensus networks (Holland & Moulton 2003; Grimm et al. 2006).

### Terminology

**Figure 1.**
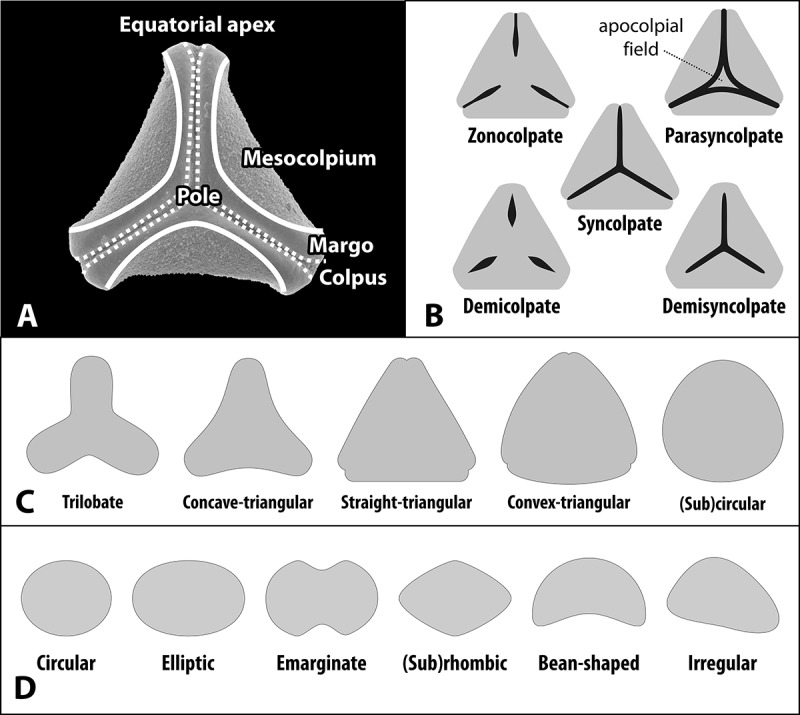
Basic terminology used for the description and categorisation of Loranthaceae pollen. **A.** Zonation of a prototypical Loranthaceae pollen grain. **B.** General aperture types. **C, D.** Outlines in polar (C) and equatorial (D) views.

We follow the terminology of Punt et al. (2007) and Hesse et al. (2009). Pollen morphology can be relatively complex in Loranthaceae even within the same genus, and morphological gradients are not uncommon. Sculpturing as seen under the SEM is typically minute but distinct, with sculptural elements usually smaller than 1.5 µm and often densely packed, in particular in the mesocolpium. Following Punt et al. (2007), we use ‘micro-’ whenever elements are not larger than 1 µm (or rarely are slightly higher/wider), ‘nano-’ is used to denote sculpturing with elements smaller than 0.5 µm. ‘Granulate’ is reserved for surfaces with a sand-sputtered appearance or matrix, i.e. very small sculptural elements (≤ 0.1 µm), in contrast to Hesse et al. (2009, p. 177). Figure 1 provides an overview over a prototypical Loranthaceae pollen grain explaining the basic zonation and surface features and schematic drawings of the basic categories that have been used to characterise the pollen of Loranthaceae.

## Results

### Molecular phylogenetic framework and basic pollen types of extant Loranthaceae

**Figure 2.**
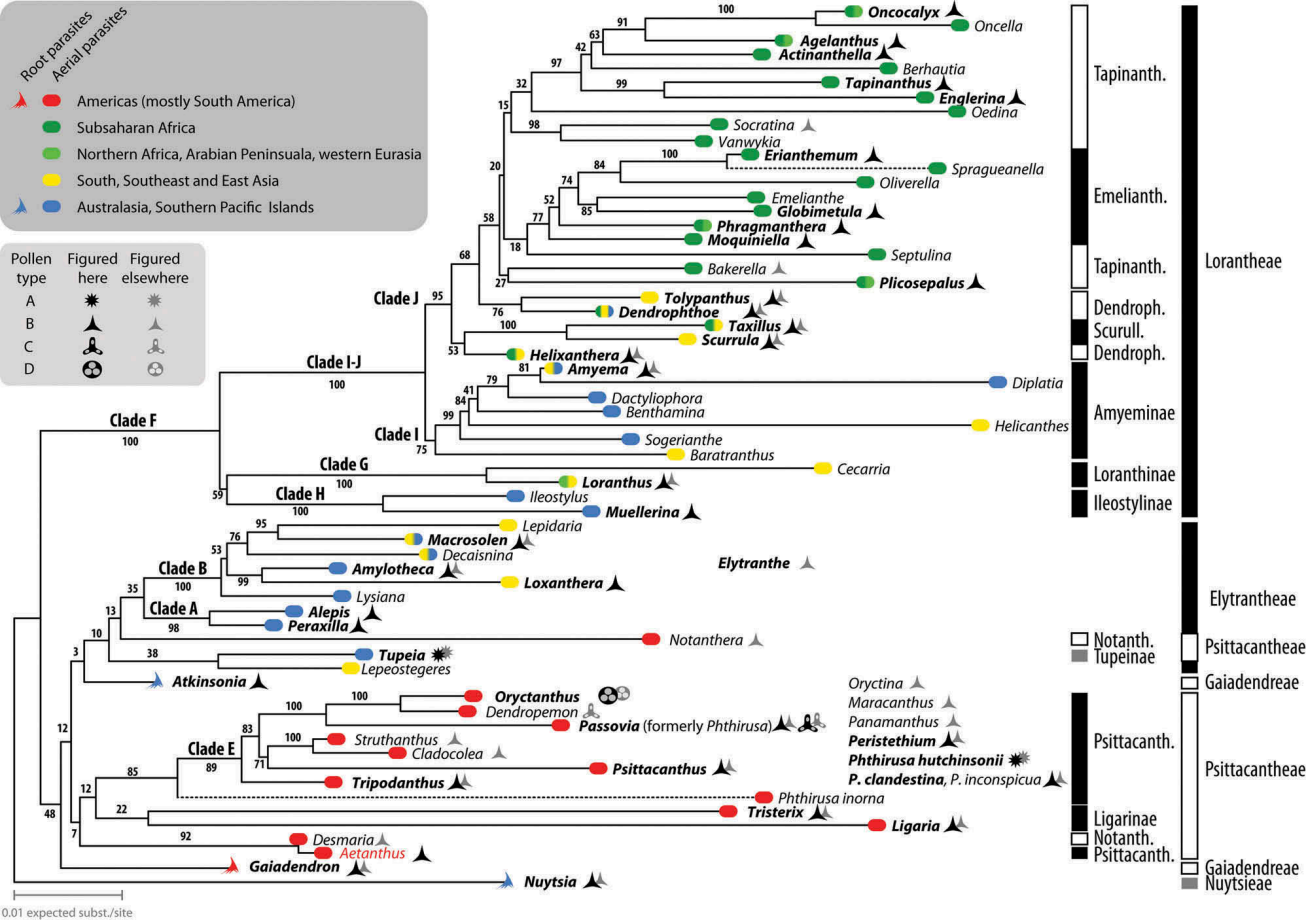
Molecular phylogram and phylogeographic-systematic framework for the Loranthaceae. Shown is the ‘best-known’ maximum likelihood tree based on a concatenated genus-consensus sequence matrix including data from two nuclear-encoded ribosomal DNA regions (18S, 25S rDNA) and three plastid regions (*mat*K gene, *trn*L intron and *trn*L-*trn*F intergenic spacer, and *rbc*L gene) rooted with *Nuytsia* as the first diverging lineage (following Vidal-Russell & Nickrent 2008b; Su et al. 2015). Stippled lines indicate branches that have been reduced by factor 2. Number at branches indicate non-parametric bootstrap (BS) support based on 1000 BS replicates. Genera palynologically studied by us in bold font. Clades labelled according to Vidal-Russell and Nickrent (2008b); systematic framework follows Nickrent et al. (2010; black bars: potential monophyletic groups; white bars: putative paraphyletic or polyphyletic groups; grey bars: monotypic groups). Red font, misplaced *Aetanthus* (missing data artefact).

To complement and verify published data (Feuer & Kuijt 1978, 1980; Kuijt 1988; Liu & Qiu 1993; Han et al. 2004; Roldán & Kuijt 2005; Caires 2012; Caires et al. 2012, 2014; see File S4), we studied 35 genera of Loranthaceae, including 15 studied for the first time to our knowledge (Figure 2; File S5). The micrographs in Liu and Qiu (1993) and Han et al. (2004) are of mediocre quality, typically out of focus, and do not allow identifying ornamental or structural details. Hence, they could not be considered for compiling pollen traits. Except for one genus (*Elytranthe*), all genera treated by Liu and Qiu (1993) and Han et al. (2004) are covered by new material in our study.

The pollen of Loranthaceae can be grouped into four readily distinguishable types (A–D) based on the features observed under LM and SEM (Table II). Of these, Type A, found in one of the palynologically studied *Phthirusa* species and the monotypic *Tupeia*, are strikingly distinct from the common Type B, and would probably not be recognised as Loranthaceae during routine LM but also not SEM (palaeo-)palynological studies. Type A is very similar to types found in other Santalales families such as the Santalaceae (Feuer & Kuijt 1978, 1982; Feuer et al. 1982). It differs from all other Loranthaceae (exclusively 3-colpate) by being 3–4- (*Tupeia*) or (3–)4–5-zonocolpate (*Phthirusa hutchisonii* [Kuijt] Kuijt) with short colpi, spheroidal and echinate. The demicolpate Types C and D share the principle aperture organisation with the common Type B but differ in overall appearance and aperture form. Moreover, Type D has a unique morphology and ornamentation and is easily distinguishable under LM. Types A, C and D are found scattered in the Loranthaceae tree (Figure 2; Table II).

**Table II.** Principle discriminating features of general pollen types in Loranthaceae.

Pollen type	Diagnostic feature or character suite	Found in
A	Spherical; zonocolpate with 3–5 very short colpi; echinate	*Tupeia, Phthirusa hutchisonii* (Psittacantheae)
B	Trilobate or triangular in polar view, basically trisyncolpate	*Nuytsia* (Nuytsieae), *Gaiadendron* (Gaiadendreae), most Psittacantheae, all Elytrantheae and Lorantheae
C	Broadly trilobate; demicolpate with broad and short colpi	*Dendropemon, Passovia pyrifolia* (Psittacantheae)
D	(Sub)circular in polar view, lophate	*Oryctanthus* (Psittacantheae)

**Figure 3.**
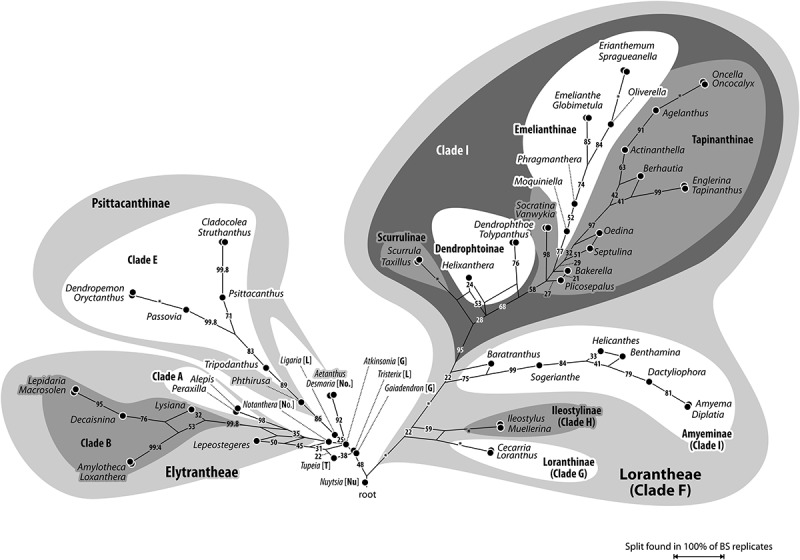
Bipartition (bootstrap support) network inferred from the maximum likelihood (ML) bootstrap (BS) sample. Edge-lengths in the graph are proportional to the frequency of the according taxon bipartition in the ML-BS replicate sample. This frequency translates into BS support when mapped on a given tree/branch; BS support values are given for each corresponding edge. Box-like structures show support of alternative, competing, partly incompatible splits, which limit BS support of putative clades in any hypothetical or optimised topology (Figure 2); hence, can provide a more comprehensive overview about the signal in the used data set. Molecular clades as found by Vidal-Russell and Nickrent (2008b) and systematic groups as defined by Nickrent et al. (2010) are annotated. Abbreviations: [G], Gaiadendreae; [Nu], Nuytsieae; Psittacantheae (not annotated in figure): [L], Ligarinae; [No], Notantherinae; Psittacanthinae (not abbreviated); [T], Tupeinae.

One of the two taxa with Type A pollen, the monotypic *Tupeia* from New Zealand, is placed as low supported (BS_ML_ = 38) sister to *Lepeostegeres*, a clade emerging from the essentially unresolved proximal part of the Loranthaceae tree. In the most recent Santalales tree (Su et al. 2015), *Tupeia* is nested within the unresolved portion of the aerial Loranthaceae clade. *Lepeostegeres* is the only member of the Elytrantheae placed outside the Elytrantheae clade in Figure 2, although its phylogenetic affinities are relatively clear (Figure 3; Su et al. 2015) and confirm its placement in the Elytrantheae (Nickrent et al. 2010). Its pollen is unknown.

The genus *Phthirusa*, including the second species with Type A pollen (*P. hutchisonii* [Kuijt] Kuijt) and represented by data from a single species (*P. inorna* [B.L. Rob. et Greenm.] Kuijt), forms an extremely long-branch sister to most other Psittacanthinae (save *Aetanthus*; a missing data artefact; BS_ML_ = 85) within an exclusively New World subtree. Also *Phthirusa’*s ITS region is hardly alignable with that of other Psittacanthinae (see File S2 and according matrix included in the OSM). The only other available data are from the most variable regions included in our data set (the nuclear-encoded 25S rDNA and the non-coding plastid *trn*L/LF region). The data (see also Wilson & Calvin 2006) has not been included in the studies of Vidal-Russell and Nickrent (2008b, figure 1, the 25S rDNA tree) and Su et al. (2015). It has to be noted that the sequenced *Phthirusa* species is, however, none of the palynologically studied species.

The enigmatic Type D is restricted to *Oryctanthus*, a genus deeply nested in the Psittacanthinae clade and resolved with unambiguous support as sister to *Dendropemon* (Figure 2; Su et al. 2015); the genus that has exclusively Type C pollen. The second genus with a species showing Type C pollen is *Passovia* (*P. pyrifolia* [Kunth] Tiegh.), resolved as member of the same subtree. No *Passovia* species with Type B pollen has been sequenced so far.

#### Pollen Type A

Small to medium-sized pollen grains, spheroidal to slightly oblate, more or less circular in polar view and equatorial view; zono(3–5)colpate; sculpturing uniform, echinate in SEM. This pollen type has so far only been found in two species: *Phthirusa* (formerly *Ixocactus*) *hutchisonii* (figured in Feuer & Kuijt 1985; see also Kuijt 2011; this study) and *Tupeia antarctica* (monotypic genus and subtribe: Tupeniae; figured in Feuer & Kuijt 1978 and this study).

#### Pollen Type B

Small to medium-sized, rarely large pollen grains, oblate to distinctly oblate (usually 1.5- to more than 3-times wider than high), trilobate to triangular in polar view, often elliptic in equatorial view, sometimes emarginate, rarely (sub)rhombic or bean-shaped (heteropolar grains), equatorial apices truncated or with more or less protruding ‘lips’ (obcordate, T-shaped), sometimes (broadly) rounded (typical for demicolpate grains); basically syn(3)colpate, in some lineages the colpi are bridged at the equator (demicolpate) or not fusing at the poles (zonocolpate with long colpi) or possibly forming an apocolpial field (parasyncolpate; Feuer & Kuijt 1985) or combinations thereof; sculpturing uniform or variable across the grain; margo mostly well-developed. This pollen type is found in the root-parasitic genera *Nuytsia* (Nuytsiae), *Atkinsonia*, *Gaiadendron* (Gaiadendreae), most Psittancantheae and all Lorantheae. Individual genera and evolutionary lineages can have more or less unique variants of pollen Type B (see following sections).

#### Pollen Type C

(Small to) medium-sized pollen grains, oblate, broadly trilobate in polar view, more or less subrhombic in equatorial view, equatorial apices (broadly) rounded; demi(3)colpate with broad and short colpi; sculpturing micro-rugulate, fossulate, perforate as far as observed in SEM (this study). This pollen type is restricted to *Dendropemon* and two species of *Passovia*, *Passovia pyrifolia* and *Passovia platyclada* (Ule) Kuijt (Feuer & Kuijt 1985; this study); the latter now treated as synonym of the former (Tropicos.org 2016). The monotypic genus *Panamanthus* (narrow colpi) has pollen that might be included in Type C.

#### Pollen Type D

Medium-sized pollen, oblate, (sub-)circular in polar view, elliptic in equatorial view; demi(3)colpate with short(?), very narrow colpi; sculpturing lophate, apocolpium with triradial symmetry, apocolpial lophae surrounding three large (intercolpial) lacunae, radial lophae straight and joining at the pole, mesocolpial lophae irregularly spaced and decreasing in height towards the ridged equator; sculpturing typically psilate with granulate patches in SEM, margo absent. This pollen type appears to be limited to genus *Oryctanthus* (Feuer & Kuijt 1985; Rubik & Moreno 1991; Caires 2012; this study).

In the following subsections, the pollen of Loranthaceae is described in a phylogenetic/systematic framework and figured. Each section starts with an overview about conservative and variable pollen traits in the respective group/lineage, followed by comprehensive species-level descriptions.

### Loranthaceae of ambiguous phylogenetic affinity

**Figure 4.**
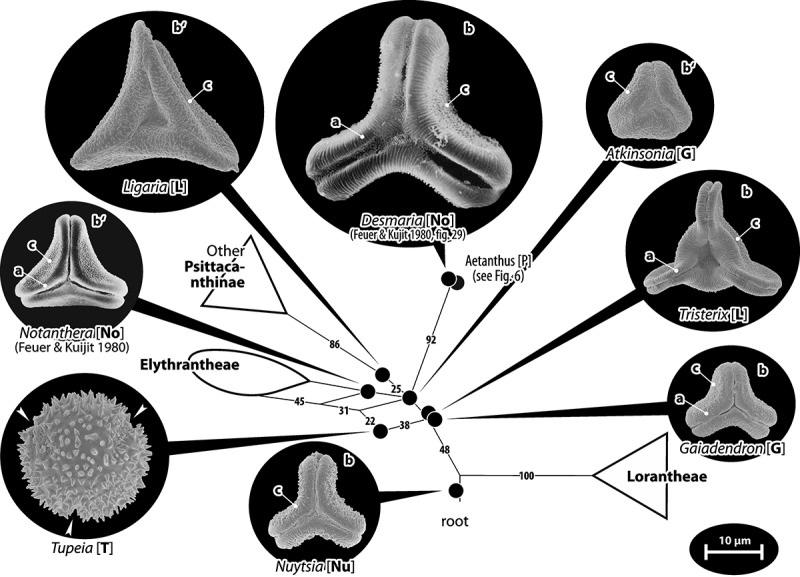
Pollen of ‘basal’ Loranthaceae, genera with ambiguous phylogenetic affinities. Pollen images (polar views) are mapped on the corresponding part of the bipartition network shown in Figure 3; morphologically and genetically well-circumscribed lineages (Psittacantinae *s.str*., Elytrantheae, Lorantheae) are collapsed for better visibility. Note that Type B pollen found in all genera except for *Tupeia* (Type A) share several, possibly plesiomorphic features of Loranthaceae such as a more or less prominently striate, well-developed margo (a), an essentially trilobate (b, modified in *Tristerix*) or convex-triangular (b′) outline in polar view, and a distinct (micro)baculate to -echinate mesocolpial sculpturing (c). White arrows indicate the position of colpi in *Tupeia*, all images are proportionally scaled to illustrate size differences. Abbreviations: [G], Gaiadendreae; [Nu], Nuytsieae; Psittacantheae: [L], Ligarinae; [No], Notantherinae; [P], Psittacanthinae (*Aetanthus*; misplaced due to a missing data artefact, see text); [T], Tupeinae.

Current molecular data fail to unambiguously resolve the phylogenetic position of the root parasites *Gaidendron* and *Atkinsonia* in relation to the aerial-parasitic lineages, as well as the placement of several Psittacantheae genera such as *Tupeia* (monotypic Tupeinae), *Ligaria, Tristerix* (Ligarinae), *Desmaria* and *Notanthera* (Notantherinae), for which palynological data are available (Figure 4). With exception of *Tupeia* (Pollen Type A), pollen grains of these genera (Type B) are (distinctly) oblate (about two-times wider than high) with a trilobate or convex-triangular outline (*Atkinsonia, Ligaria, Notanthera*) in polar view. The lobes’ apices in the equatorial plane are typically obcordate. The apertures are syncolpate with narrow to medium-wide colpi, and have, except for *Atkinsonia* and *Ligaria*, a well-developed margo. The margo’s sculpturing can be distinctly striate (*Desmaria, Gaiadendron, Tristerix, Notanthera* to some degree), with the striae perpendicular to the colpi. Pollen grains of genera in this heterogenous group differ in size and sculpturing (Table III).

**Table III.** Tabulation of differentiating pollen features in ‘basal’ Loranthaceae/Loranthaceae with ambiguous phylogenetic affinities (unresolved using current molecular data).

Classification	Genus	PT	Apertures	Size, shape, outline (e.v., p.v.)^a^	Sculpturing	Special exine features
Nuytsieae	*Nuytsia*	B	Syn(3)colpate	Small, oblate, elliptic, trilobate	M: distinct, **mostly psilate** MC: (micro)echinate	Hexagonal thickening of polar nexine (LM)
Gaiadendreae	*Atkinsonia*	B	Syn(3)colpate	Small, oblate, elliptic, triangular (straight to concave)	**Micro-rugulate**, margo indistinct	Sexine thickened in MC (LM)
	*Gaiadendron*	B	Syn(3)colpate	Small, oblate, elliptic, trilobate	M: distinct, striate MC: nano-baculate/-echinate	Hexagonal thickening of polar nexine (LM)
Psittacantheae: Ligarinae	*Ligaria*	B	Syn(3)colpate	Medium, distinctly oblate, emarginate, concave-triangular	**Micro-baculate**, margo indistinct and indifferent	**Triangular thickening of polar nexine (LM), polar sexine reduced (SEM)**
	*Tristerix*	B	Syn(3)colpate	Small or medium, distinctly oblate, emarginate, **modified trilobate**	M: distinct, striate, forming triangular polar protrusions MC: nano- to micro-echinate/-baculate	**Triradial thickening of polar nexine between colpi (LM), polar sexine reduced (SEM)**
Notantherinae	*Desmaria*	B	Syn(3)colpate	Medium, distinctly oblate, elliptic, trilobate	M: distinct, striate, forming triangular polar protrusions MC: micro-baculate/-echinate (?)	Polar ectexine and endexine thickened (TEM)
	*Notanthera*	B	Syn(3)colpate	Small, oblate, outline in e.v. unknown, concave-triangular	M: **weakly striate**, forming triangular polar protrusions MC: micro(?)echinate/-baculate	Inconspicuous
Tupeinae	*Tupeia*	**A**	**Zono(3–4)-colpate**	Small to medium, **± spheroidal**	**Uniform: (micro)echinate, echini widely spaced**	Inconspicuous

Note: Abbreviations: PT, pollen type; e.v., equatorial view; p.v., polar view; M, margo; MC, mesocolpium; most diagnostic features in bold font; ^a^ Size: small, < 25 µm; medium, 25–50 µm; shape: oblate, less than two-times wider than high; distinctly oblate, more than two-times wider than high.

Nuytsieae

#### Remark

A monogeneric tribe including a single, root-parasitic species, *Nuytsia floribunda*, considered to represent the first diverging lineage of the Loranthaceae.

*Nuytsia floribunda* (Labill.) G.Don (Figure 9)

**Figure 5.**
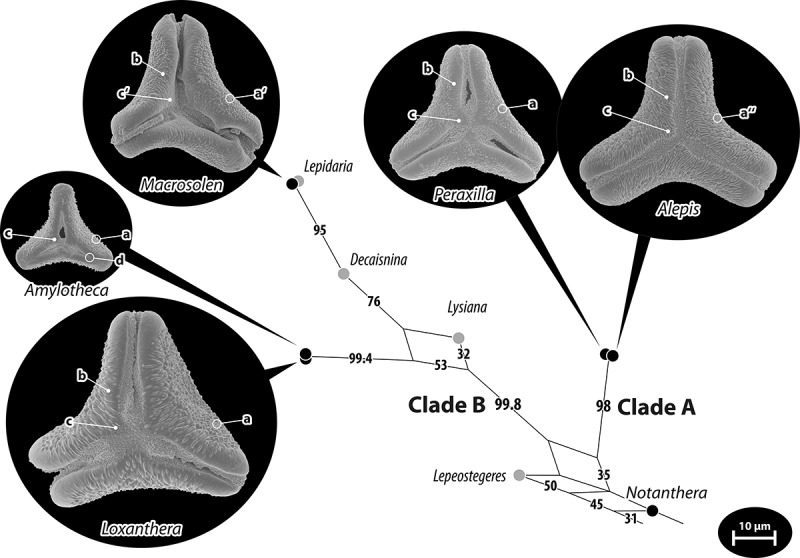
Pollen of Elytrantheae, an Australasian-East Asian Loranthaceae lineage. Pollen images (polar views) are mapped on the corresponding part of the bipartition network shown in Figure 3; Clades A and B according Vidal-Russell and Nickrent (2008b) are indicated. Diagnostic features are highlighted: (a) distinct micro-echinate mesocolpium, becoming micro-verrucate (a′) in *Macrosolen* and rugulate in *Alepis* (a″); (b) the partly or fully (*Alepis*) striate margo, forming triangular polar protrusions (c), weakly developed in *Macrosolen* (c′); and (d) demisyncolpate apertures found only in *Amylotheca.*

**Figure 6.**
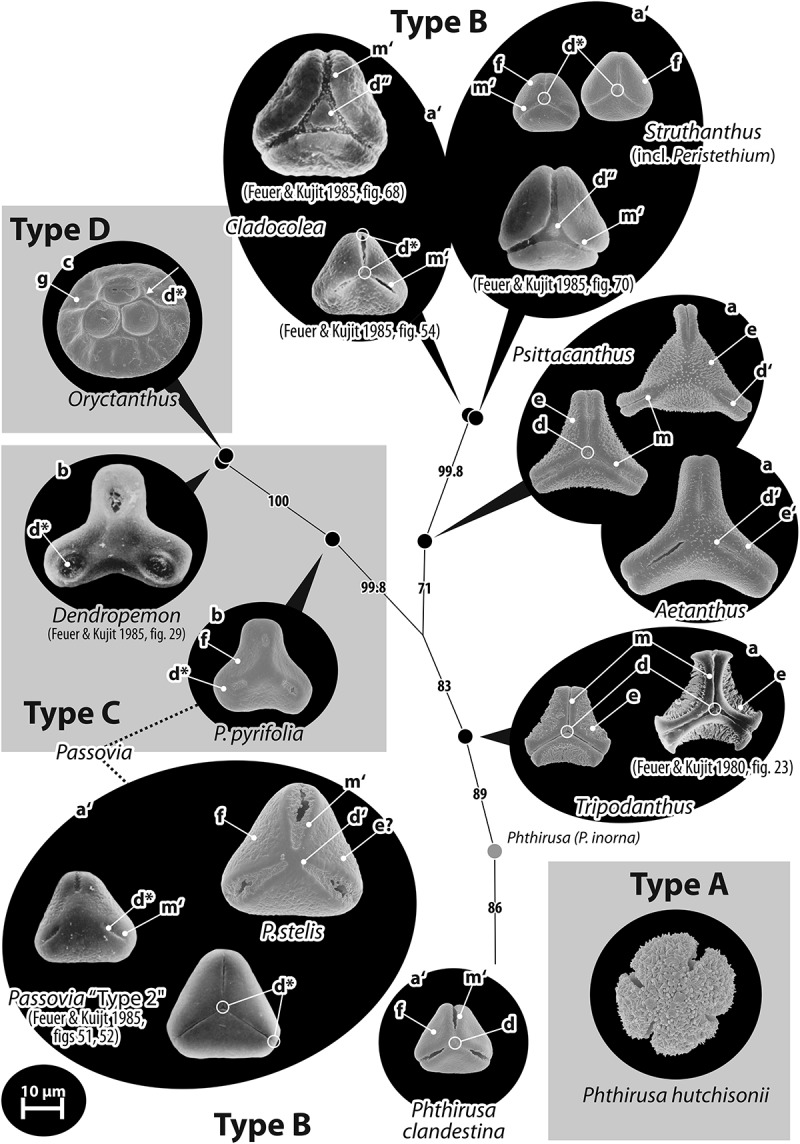
Pollen of Psittacanthinae, an exclusively New World Loranthaceae lineage. Pollen images (polar views) are mapped on the corresponding part of the bipartition network shown in Figure 3. According to the phylogenetic framework, pollen Types C and D are derived from pollen Type B; the relationship of the only species with pollen Type A is unknown. Several evolutionary trends are indicated: polar outline changes from convex-triangular (a) to straight- to concave-triangular (a′), broadly trilobate (b), and (sub)circular (c); apertures evolve from syncolpate (d) to zonocolpate (d′), parasyncolpate (d″) and demi(syn)colpate (d*); the margo (m) is reduced and becomes indistinct (m′); and the sculpturing of the exine surface smoothens, it can be (micro)baculate or -echinate (e), micro-rugulate (f), or psilate to granulate (g).

**Figure 7.**
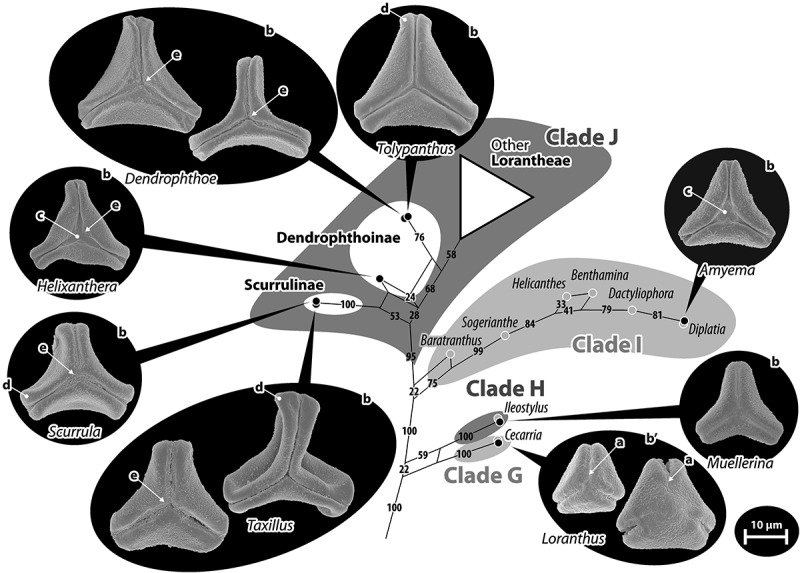
Pollen of Lorantheae: Loranthinae, Amyeminae, Scurrulinae, and Dendrophthoinae. Pollen images (polar views) are mapped on the corresponding part of the bipartition network shown in Figure 3; Clades G–J according to Vidal-Russell and Nickrent (2008b) are indicated. Pollen of *Loranthus* (Loranthinae, Clade G) differs by its zonocolpate aperture organisation (particular on the distal face of heteropolar grains), where the colpi do not join at the pole (a), and the straight-triangular outline in polar view (b′) in contrast to convex-triangular to trilobate (b). Colpi are usually ± narrow, occasionally widening towards the pole joining in a polar depression (c). Note the trend towards T-shaped apices (d) and formation of more or less developed triangular polar protrusions (e) of the more or less prominent margo in the core Lorantheae (Clade J).

**Figure 8.**
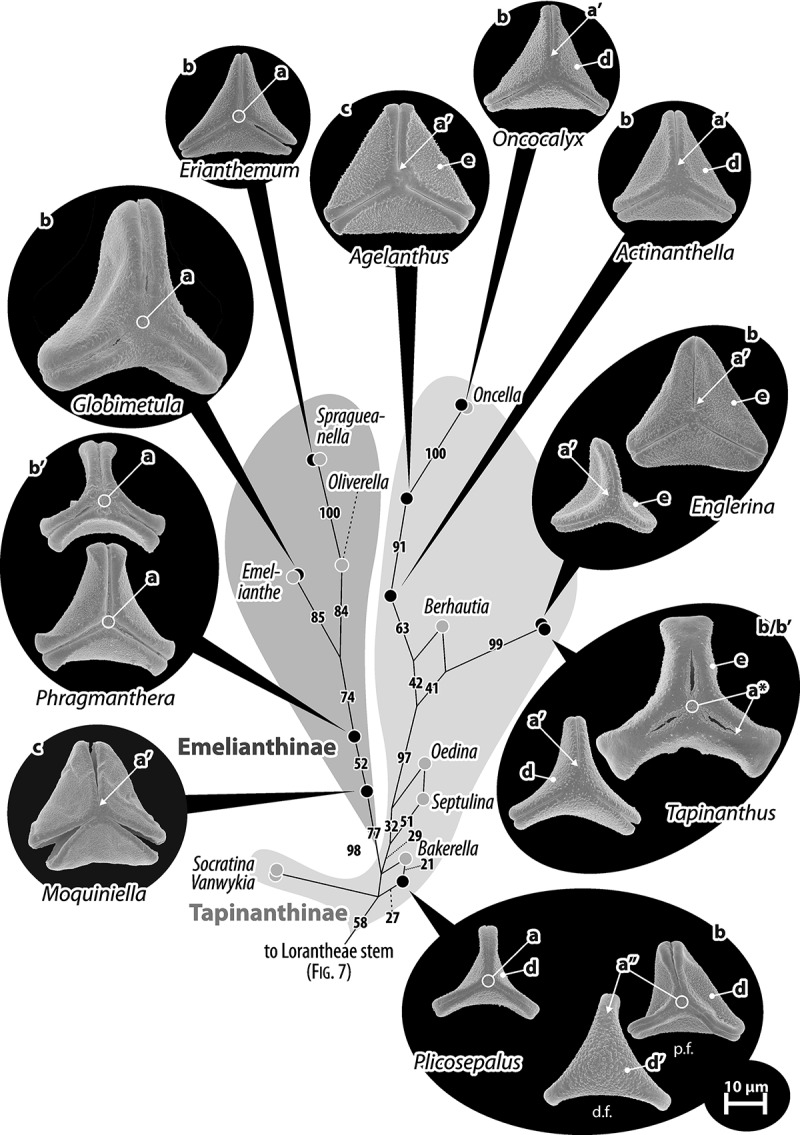
Pollen of Lorantheae: Tapinanthinae and Emelianthinae (subclade of Clade J of Vidal-Russell & Nickrent 2008b). Pollen images (polar views) are mapped on the corresponding part of the bipartition network shown in Figure 3. Grains can be isopolar-syncolpate (a), isopolar-zonocolpate (a′) or heteropolar with a syncolpate proximal and zonocolpate distal side (a″); two of the three *Tapinanthus* species have demisyncolpate pollen (a*). The pollen are either convex-triangular to trilobate in outline (b), sometimes with T-shaped equatorial apices (b′), or more or less straight-triangular (c). Sculpturing in the mesocolpium is nano-verrucate to granulate (d), rarely micro-verrucate (d′), or nano- to micro-baculate/-echinate (e). Abbreviations: d.f., distal face; p.f., proximal face.

**Figure 9.**
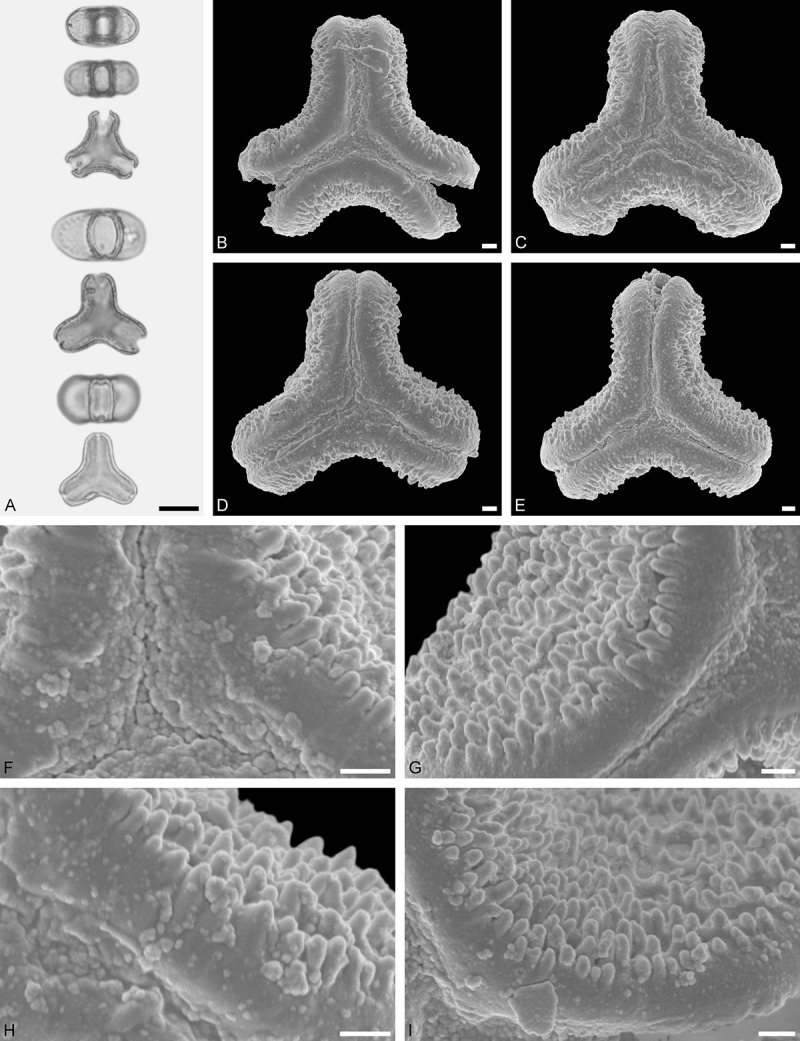
LM (A) and SEM (B–I) micrographs of *Nuytsia floribunda* (WU: origin unknown, coll. Baron F. von Mueller, s.n.). **A.** Three pollen grains in equatorial and polar view. **B–E.** Pollen grains in polar view. **F.** Close-up of central polar area. **G.** Close-up of apex. **H, I.** Close-up of mesocolpium. Scale bars – 10 µm (A), 1 µm (B–I).

#### Description

Pollen, oblate, trilobate in polar view, elliptic in equatorial view, equatorial apices obcordate; size small, polar axis 10.0–13.3 µm long in LM, equatorial diameter 15.0–18.3 µm in LM, 11.7–13.3 µm in SEM; syn(3)colpate; exine 0.8–1.3 µm thick, nexine thinner than sexine, nexine hexagonally thickened in polar area (LM); tectate; sculpturing psilate in LM, micro-echinate to echinate in area of mesocolpium in SEM, echini 0.5–1.4 µm long, 0.4–0.6 µm wide at base; margo well developed, margo mostly psilate around colpi, sometimes partly granulate; colpus membrane nano-verrucate to nano-echinate (SEM).

#### Remark

Pollen Type B. In overall appearance and size, the pollen of *Nuytsia* is most similar to that of *Gaiadendron*, but differs in the sculpturing of the margo (psilate vs. striate in *Gaiadendron*).

Gaiadendreae

#### Remark

A tribe with two monotypic (*Atkinsonia*) or bitypic (*Gaiadendron*), root-parasitic genera of uncertain phylogenetic affinity (Figures 2, 3) and with markedly different Type B pollen. The pollen of the Australian *Atkinsonia* is unique (micro-rugulate sculpturing) within the early diverging Loranthaceae. The Central to northern South American *Gaiadendron punctatum* has a potentially archetypical Type B pollen (Figure 11) relatively similar to pollen of *Nuytsia* and several Psittacantheae genera of unclear phylogenetic affinity.

**Figure 10.**
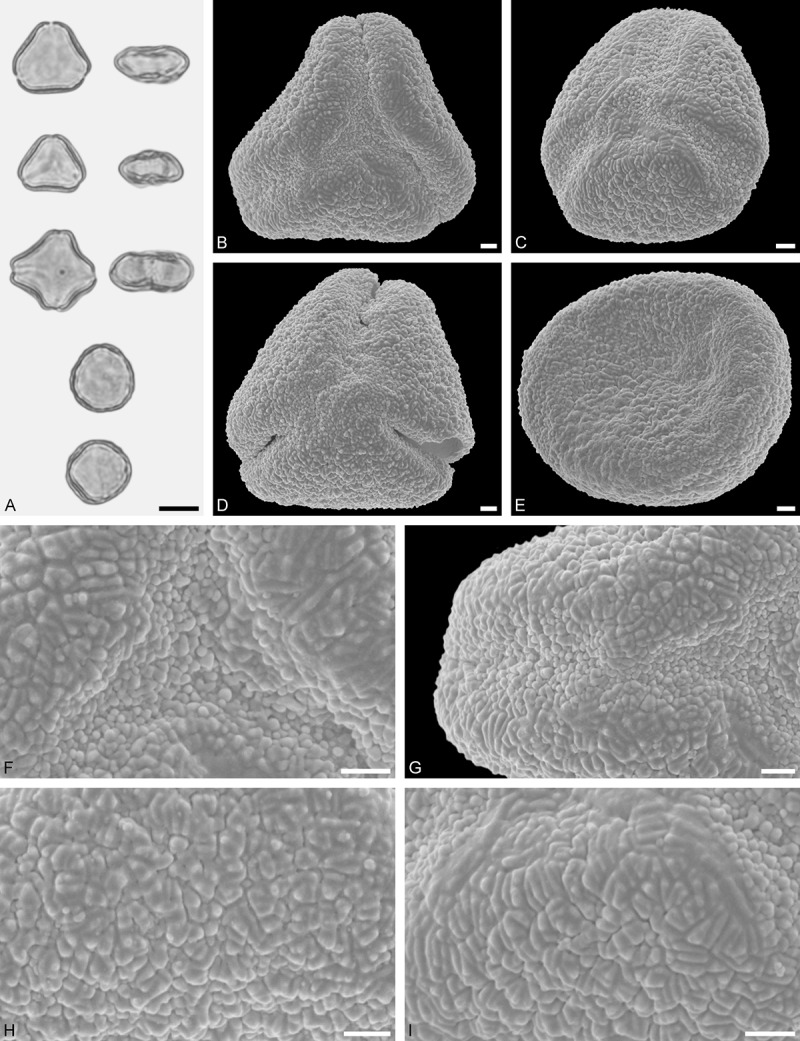
LM (A) and SEM (B–I) micrographs of *Atkinsonia ligustrina* (MEL 2214054). **A.** Two triangular pollen grains in polar and equatorial view (normal types). One quadrangular pollen grain in polar and equatorial view (rare, abnormal). Two subcircular grains in polar view (aberrant types). **B–D.** Pollen grains in polar view (normal types). **E.** Subcircular aberrant pollen grain. **F.** Close-up of central polar area. **G.** Close-up of apex. **H, I.** Close-ups of mesocolpium. Scale bars – 10 µm (A), 1 µm (B–I).

**Figure 11.**
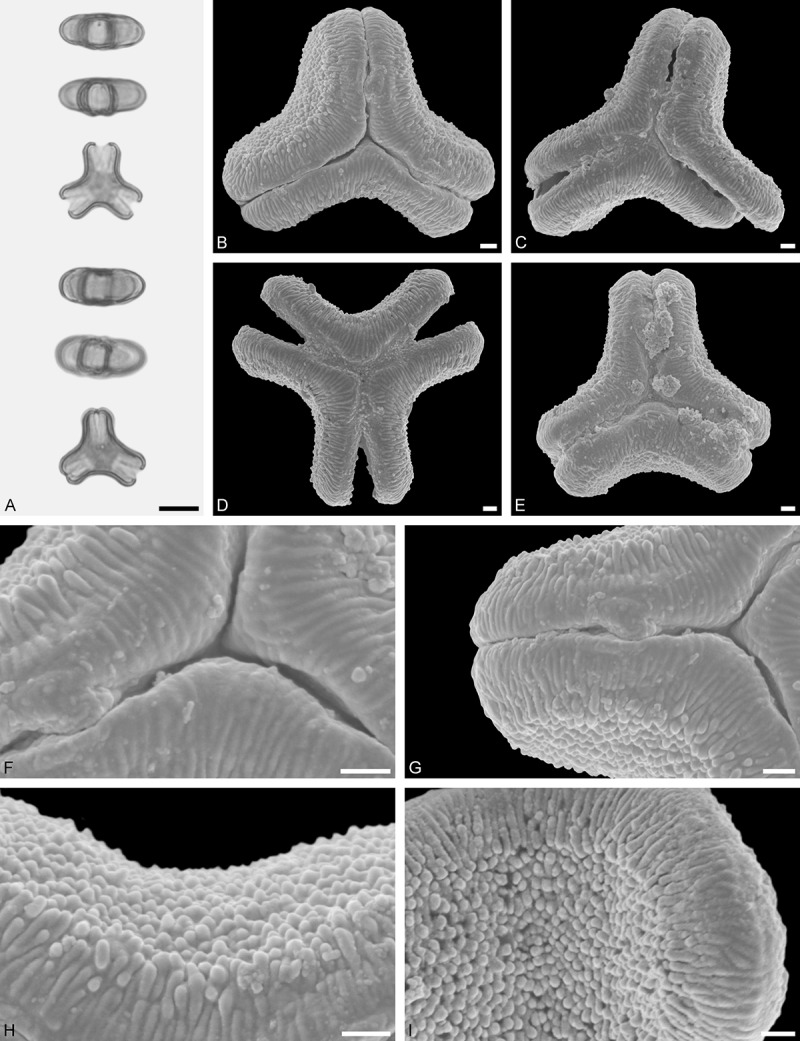
LM (A) and SEM (B–I) micrographs of *Gaiadendron punctatum* (WU: from Costa Rica, collector unknown, 5.2.94–912). **A.** Two pollen grains in equatorial and polar view. **B–E.** Pollen grains in polar view. **F.** Close-up of central polar area. **G.** Close-up of apex. **H, I.** Close-ups of mesocolpium. Scale bars – 10 µm (A), 1 µm (B–I).

*Atkinsonia ligustrina* F.Muell (Figure 10)

#### Description

Pollen, oblate, straight-triangular to convex-triangular in polar view, elliptic in equatorial view, equatorial apices obcordate to rounded; size small, polar axis 8.3–11.7 µm long in LM, equatorial diameter 15.0–18.3 µm in LM, 11.5–16.3 µm in SEM; syn(3)colpate; exine 1.2–1.4 µm thick, nexine thinner than sexine, sexine slightly thickened in area of mesocolpium (LM); tectate, sculpturing psilate in LM, micro-rugulate to micro-areolate in area of mesocolpium in SEM, microareolae often with nano-echinate suprasculpture; margo indistinct, sculptured like mesocolpium; colpus membrane nano-verrucate to nano-echinate (SEM).

#### Remark

Pollen Type B. Pollen from anthers of *Atkinsonia ligustrina* are ≥75% syn(3)colpate and straight-triangular to convex-triangular. Aberrant, deformed pollen grains are relatively frequent, ≈20%, and differ in shape/outline (circular to irregular in polar view) and form/arrangement of apertures (irregularly distributed, short/pori like, or absent) and partly SEM sculpturing. Previous studies by Feuer and Kuijt (1980) described and figured only untypical aberrant pollen grains of this species leading them to the wrong conclusion that *Atkinsonia* is characterised by inaperturate pollen.

*Gaiadendron punctatum* (Ruiz et Pav.) G.Don (Figure 11)

#### Description

Pollen, oblate, trilobate in polar view, elliptic in equatorial view, equatorial apices obcordate; size small, polar axis 8.3–15.0 µm long in LM, equatorial diameter 15.8–18.3 µm in LM, 12.0–15.3 µm in SEM; syn(3)colpate; exine 1.1–1.4 µm thick, nexine thinner than sexine, nexine hexagonally thickened in polar area (LM); tectate; sculpturing psilate in LM, nano-baculate to nano-echinate in area of mesocolpium in SEM, bacula/echini 0.2–0.5 µm wide at base; margo well developed, margo striate, striae perpendicular to colpi; colpus membrane nano-verrucate to nano-echinate (SEM). – Pollen Type B.

Psittacantheae

#### Remark

Paraphyletic tribe with one larger and several few-species subtribes including genera of ambiguous phylogenetic affinity (Figures 2, 3; Table III): Ligarinae with *Ligaria* (monotypic) and *Tristerix*, Notantherinae with the monotypic genera *Desmaria* and *Notanthera*, and the monotypic Tupeinae with *Tupeia*. Most of the Central and South American members of the Psittacantheae are part of the Psittacanthinae that fall within a distinct and well-supported clade in molecular trees, and are described in the next section. The only Psittacanthinae genus placed outside the Psittacanthinae clade in our reconstructions (Figures 2, 3) is *Aetanthus*. Note that this is a misplacement due to insufficient data (only 18S rDNA data included here, see File S2). The correct placement is as sister to *Psittacanthus* (anonymous reviewer, personal communication, 2016), as informed by *mat*K data (Vidal-Russell & Nickrent 2008b; Su et al. 2015).

Ligarinae

**Figure 12.**
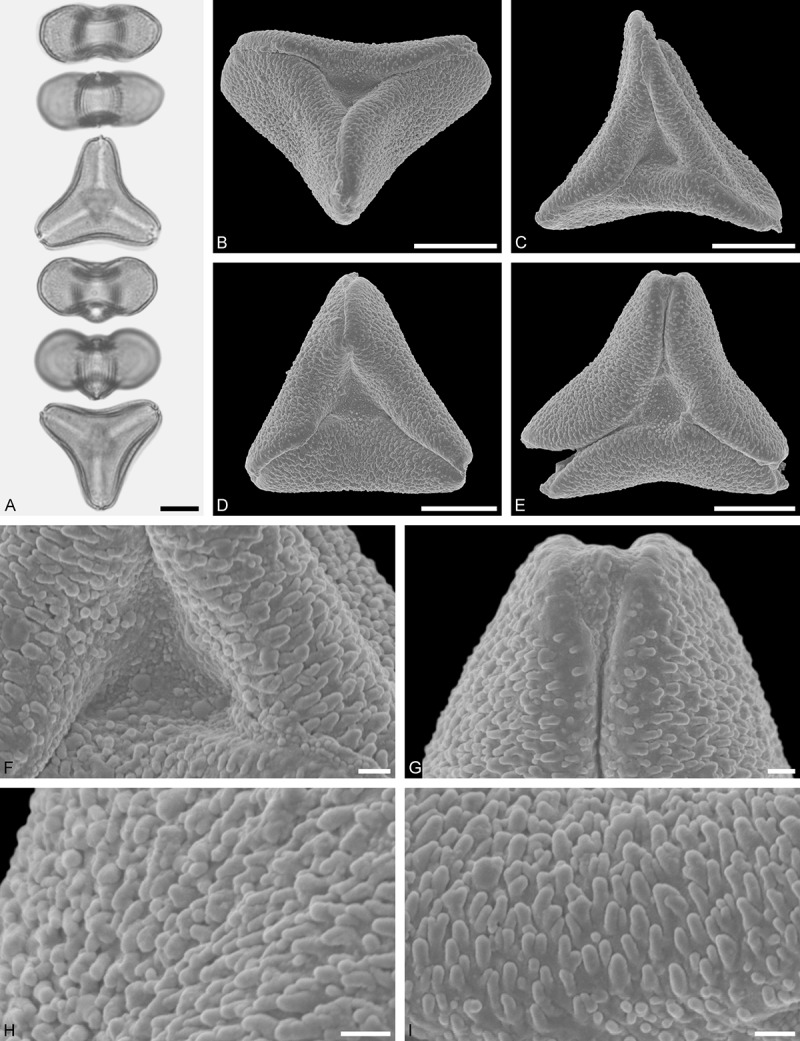
LM (A) and SEM (B–I) micrographs of *Ligaria cuneifolia* (WU: from Argentina, collector unknown, s.n.). **A.** Two pollen grains in equatorial and polar view. **B–E.** Pollen grains in polar view. **F.** Close-up of central polar area. **G.** Close-up of apex. **H, I.** Close-ups of mesocolpium. Scale bars – 10 µm (A–E), 1 µm (F–I).

*Ligaria cuneifolia* (Ruiz et Pav.) Tiegh. (Figure 12)

#### Description

Pollen, distinctly oblate, (concave-)triangular in polar view, emarginate in equatorial view, equatorial apices rounded to obcordate; size medium, polar axis 11.7–13.3 µm long in LM, equatorial diameter 25.0–30.0 µm in LM, 24.1–29.0 µm in SEM; syn(3)colpate; exine 1.1–1.3 µm thick, nexine thinner than sexine, nexine hexagonally thickened in polar area (LM); tectate; sculpturing psilate in LM, micro-baculate in area of mesocolpium in SEM, microbacula 0.5–1.1 µm long, 0.2–0.5 µm wide at base; margo indistinct, margo sculptured like mesocolpium; colpus membrane nano-verrucate to nano-echinate and granulate (SEM).

#### Remark

Pollen Type B. Pollen of *Ligaria* is very distinct, being characterised by a very indistinct margo sculptured in the same way as the mesocolpium. Differing markedly in outline, it shares the feature of a markedly reduced polar sexine with its putative sister genus *Tristerix*.

**Figure 13.**
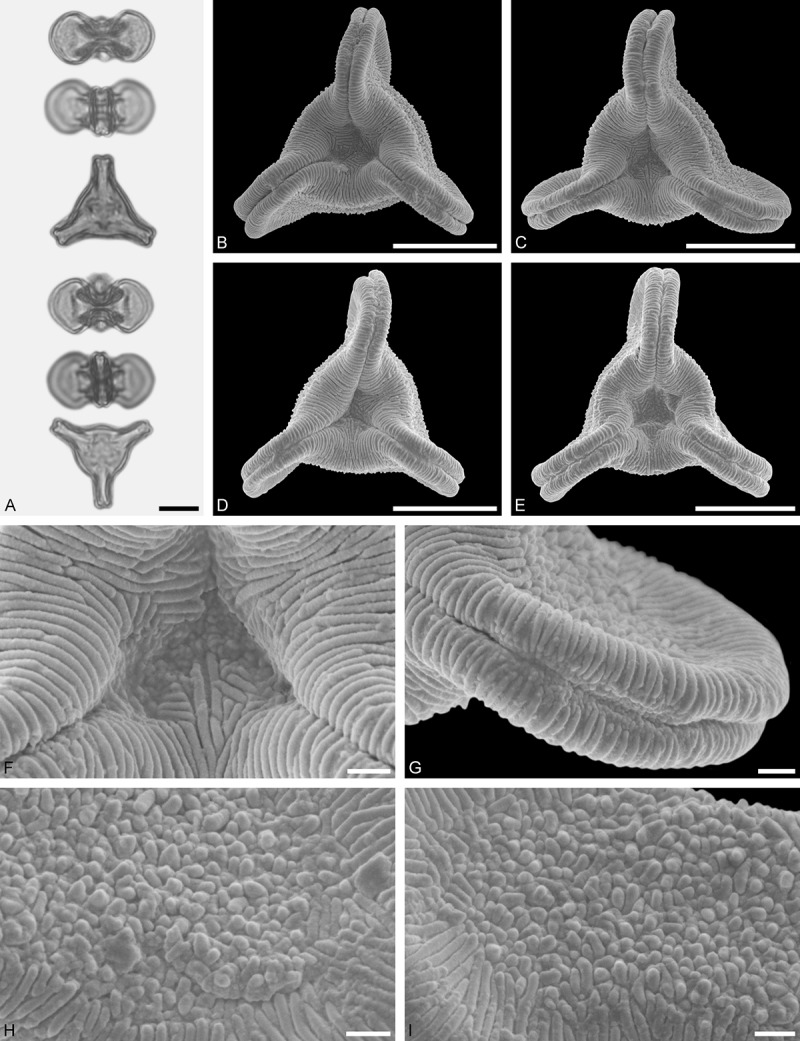
LM (A) and SEM (B–I) micrographs of *Tristerix aphyllus* (WU 066237). **A.** Two pollen grains in equatorial and polar view. **B–E.** Pollen grains in polar view. **F.** Close-up of central polar area. **G.** Close-up of apex. **H, I.** Close-ups of mesocolpium. Scale bars – 10 µm (A–E), 1 µm (F–I).

*Tristerix aphyllus* (Miers ex DC.) Barlow et Wiens (Figure 13)

#### Description

Pollen, distinctly oblate, trilobate (gear wheel-like) in polar view, emarginate in equatorial view, equatorial apices obcordate; size small, polar axis 6.6–8.3 µm long in LM, equatorial diameter 20.0–21.7 µm in LM, 18.6–20.5 µm in SEM; syn(3)colpate; exine 1.1–1.5 µm thick, nexine thinner than sexine, triangular intercolpial nexine thickenings in polar area (LM), sexine partly reduced in polar area, colpi widening to a small field (SEM); tectate; sculpturing psilate in LM, nano-/micro-echinate to nano-/micro-baculate in area of mesocolpium in SEM, echini/bacula 0.3–0.8 µm long, 0.2–0.4 µm wide at base; margo well developed, margo striate, striae perpendicular to colpi, margo with triangular protrusions in polar area (SEM).

#### Remark

Pollen Type B. The outline of *Tristerix* pollen in polar view is unique within the family, reflecting the genetic distinctness of the genus (Figure 2; Su et al. 2015). The pollen has a nearly rounded central body and narrow, straight equatorial apices (lobes), giving it the appearance of a gear wheel with three teeth. Similar equatorial apices can be found occasionally in species of other genera (*Psittacanthus rhynchanthus*, Psittacanthinae; *Actinanthella menyhartii*, Tapinanthinae), but these pollen differ in the shape of the central body (essentially triangular with retracting mesocolpium towards equatorial apices) and the sculpturing of the margo and mesocolpium.

**Figure 14.**
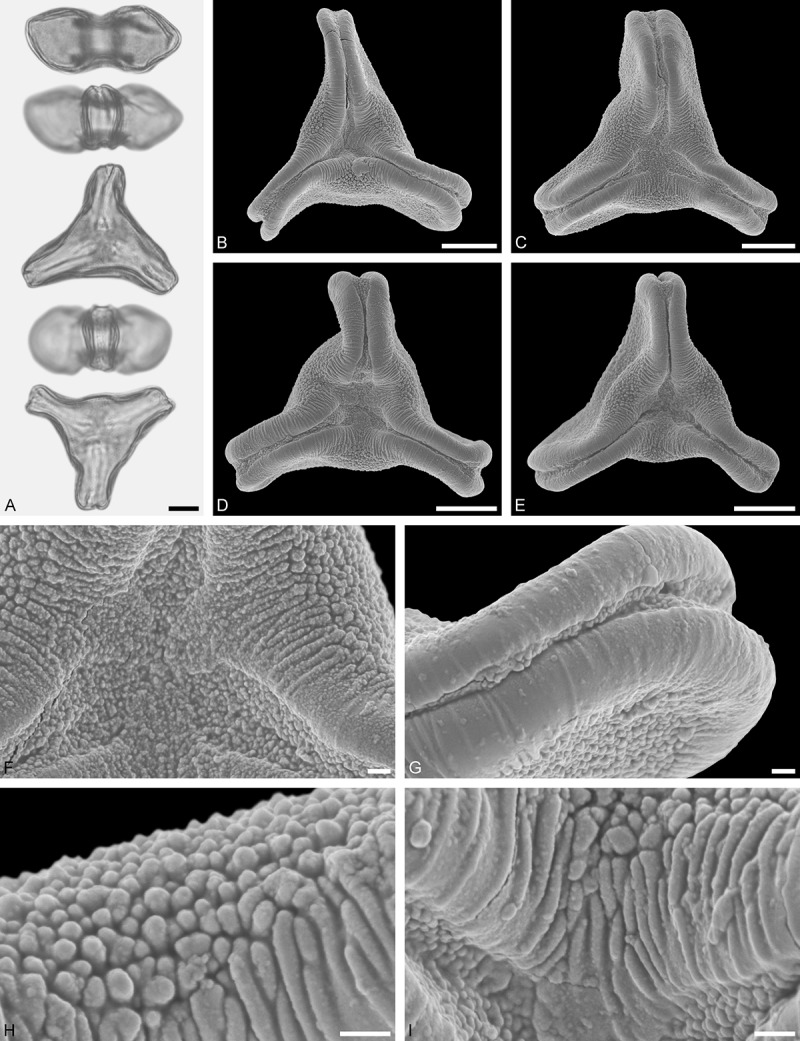
LM (A) and SEM (B–I) micrographs of *Tristerix longibracteatus* (WU: from Ecuador, coll. J. Jaramillo, s.n.). **A.** Two pollen grains in equatorial and polar view. **B–E.** Pollen grains in polar view. **F.** Close-up of central polar area. **G.** Close-up of apex. **H.** Close-up of mesocolpium. **I.** Close-up of margo in central polar area. Scale bars – 10 µm (A–E), 1 µm (F–I).

*Tristerix longebracteatus* (Desr.) Barlow et Wiens (Figure 14)

#### Description

Pollen, distinctly oblate, trilobate in polar view, emarginate in equatorial view, equatorial apices obcordate; size medium, polar axis 13.3–15.0 µm long in LM, equatorial diameter 36.6–41.7 µm in LM, 32.7–38.2 µm in SEM; syn(3)colpate; exine 1.3–1.7 µm thick, nexine thinner than sexine, triangular intercolpial nexine thickenings in polar area (LM), sexine partly reduced in polar area, colpi widening to a small field (SEM); tectate; sculpturing psilate in LM, nano-/micro-echinate to nano-/micro-baculate in area of mesocolpium in SEM, echini/bacula 0.3–0.7 µm long, 0.2–0.5 µm wide at base; margo well developed, margo striate, striae perpendicular to colpi, margo with triangular protrusions in polar area; colpus membrane nano-verrucate to nano-echinate (SEM).

#### Remark

Pollen Type B. Pollen of *Tristerix longebracteatus* are very similar to those of *T. aphyllus*, they differ manly in size. *Tristerix longebracteatus* pollen grains are much larger. In addition, the striae on the margo are less distinct (compare Figure 13G with Figure 14G).

Notantherinae

*Desmaria mutabilis* (Poepp. et Endl.) Tiegh. ex B.D.Jacks.

#### Description (cf. Feuer & Kuijt 1980, figures 29, 31, 32, 34)

Pollen, distinctly oblate, trilobate in polar view, elliptic in equatorial view, equatorial apices obcordate; size medium (cf. Feuer & Kuijt 1980, table 1); syn(3)colpate; polar ectexine and entexine thickened (TEM); tectate; not figured in LM, sculpturing probably micro-baculate to micro-echinate in SEM; margo well developed, forming triangular polar protrusions, striate, with striae perpendicular to colpi (SEM).

#### Remark

Pollen Type B. The pollen of *Desmaria* is substantially different from that of its morphologically closest relative *Notanthera*, the other genus of the Notantherinae (but see Su et al. 2015). It is similar to the pollen found in the Elytrantheae, but shares the striate margo seen in several of the early diverging Loranthaceae listed in Table III. Its phylogenetic position is essentially unresolved based on molecular data (Figures 2, 3; Su et al. 2015).

*Notanthera heterophylla* (Ruiz et Pav.) G.Don

#### Description (cf. Feuer & Kuijt 1980, figures 5, 22)

Pollen, oblate, concave-triangular in polar view, not figured in equatorial view, equatorial apices obcordate; size small (cf. Feuer & Kuijt 1980, table 1); syn(3)colpate; tectate; not figured in LM, sculpturing probably nano-baculate/-echinate in SEM; margo well developed, forming triangular polar protrusions, psilate at pole, weakly striate in mesocolpium (SEM).

#### Remark

Pollen Type B. Within the group of Loranthaceae with unclear affinity the character suite showed by pollen of *Notanthera* is unique. It combines features such as a (partly) striate margo as seen in *Desmaria* (same subtribe), *Gaiadendron*, and *Tristerix*, a prominent margo as seen in large-flowered Psittacanthinae (the sister group according to Vidal-Russell & Nickrent [2008b, figure 3], and Su et al. [2015, figure 2]) with a sculpturing in the mesocolpium that shows an overall resemblance to pollen of the Lorantheae.

Tupeinae

**Figure 15.**
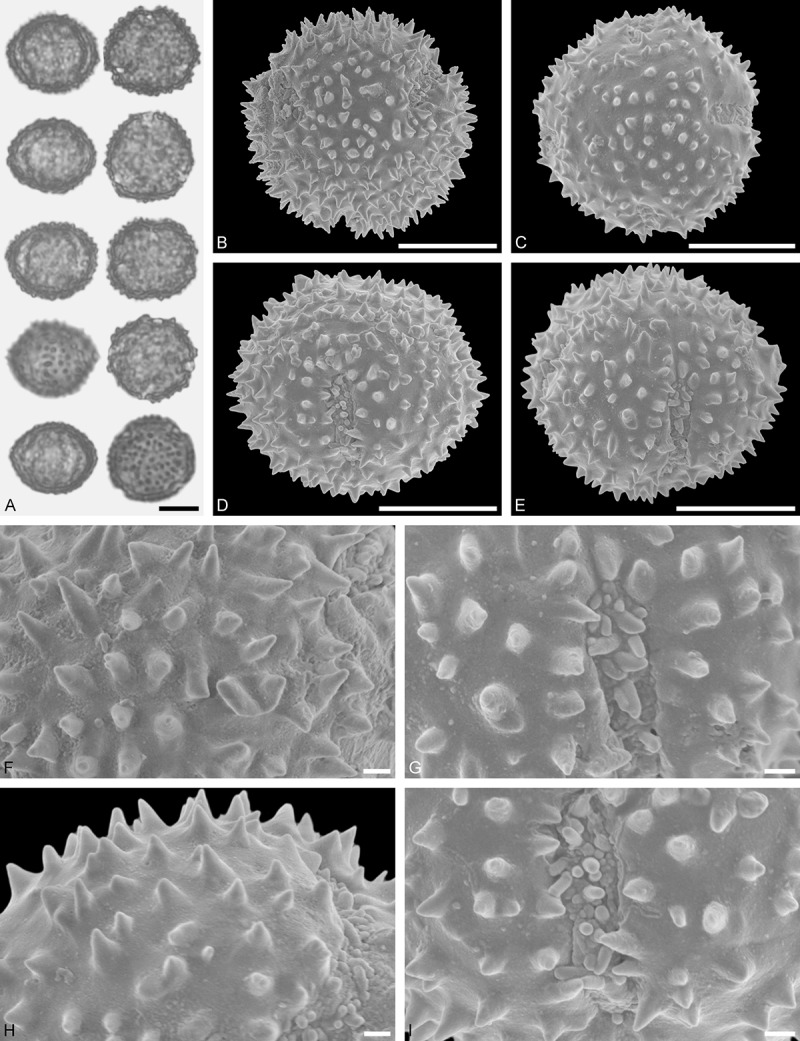
LM (A) and SEM (B–I) micrographs of *Tupeia antarctica* (MO: from New Zealand, coll. R. Gardner, s.n.). **A.** Five pollen grains in equatorial and polar view, two zono(3)colpate grains followed by three zono(4)colpate grains. **B.** Zono(3)colpate grain in polar view. **C.** Zono(4)colpate grain in polar view. **D, E.** Pollen grains in equatorial view. **F.** Close-up of central polar area. **G.** Close-up of colpi. **H.** Close-up of mesocolpium. **I.** Close-up of colpi and colpus membrane. Scale bars – 10 µm (A–E), 1 µm (F–I).

*Tupeia antarctica* (G. Forst.) Cham. et Schltdl. (Figure 15)

**Figure 16.**
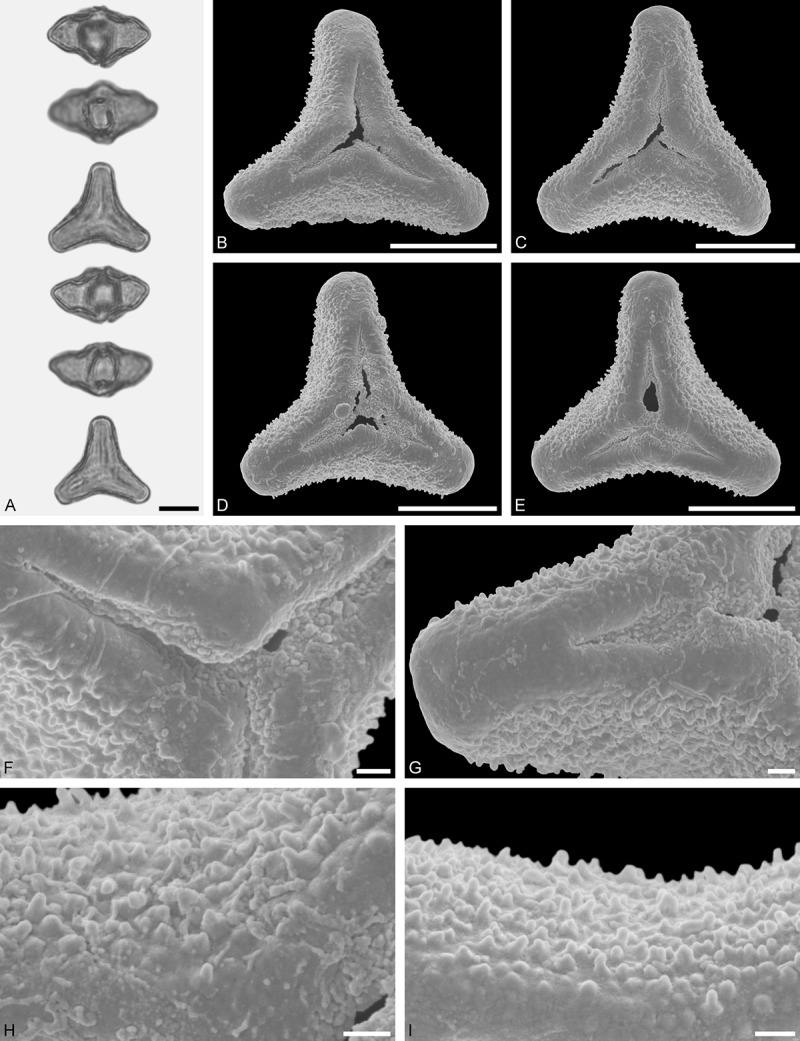
LM (A) and SEM (B–I) micrographs of *Amylotheca* sp. (WU: from Philippines, coll. Sterner, s.n.). **A.** Two pollen grains in equatorial and polar view. **B–E.** Pollen grains in polar view. **F.** Close-up of central polar area. **G.** Close-up of apex. **H, I.** Close-ups of mesocolpium. Scale bars – 10 µm (A–E), 1 µm (F–I).

**Figure 17.**
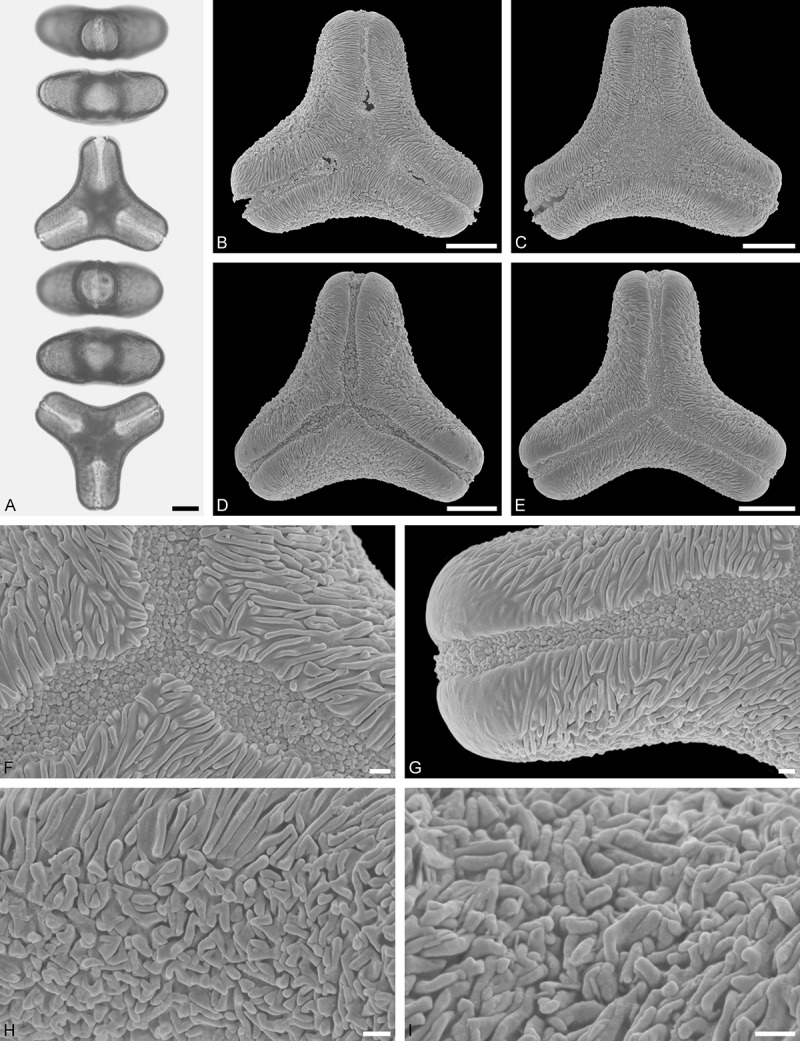
LM (A) and SEM (B–I) micrographs of *Alepis flavida* (WU: from New Zealand, coll. Raven, s.n.). **A.** Two pollen grains in equatorial and polar view. **B–E.** Pollen grains in polar view. **F.** Close-up of central polar area. **G.** Close-up of apex. **H, I.** Close-ups of mesocolpium. Scale bars – 10 µm (A–E), 1 µm (F–I).

#### Description

Pollen, spheroidal to slightly oblate, subcircular in polar and equatorial view; size small to medium, polar axis 18.3–21.7 µm long in LM, 20.0–22.0 in SEM, equatorial diameter 23.0–25.0 µm in LM, 21.4–24.4 µm wide in SEM; zono(3–4)colpate, colpi short and wide, colpi 8.0–9.5 µm long in SEM; exine 1.2–1.7 µm thick, nexine thinner than sexine (LM); tectate; sculpturing uniform, echinate in LM and SEM, echini 0.8–1.6 µm long, 0.5–1.2 µm wide at base; colpus membrane micro-echinate to echinate (SEM).

#### Remark

Pollen Type A. The spheroidal, echinate pollen of *Tupeia* is most distinct within Loranthaceae. The only species with a similar pollen morphology and structural elements of similar size is *Phthirusa hutchisonii*, an isolated Psittacanthinae species of unknown phylogenetic relationships (see ‘Discussion’).

Elytrantheae clade

**Figure 18.**
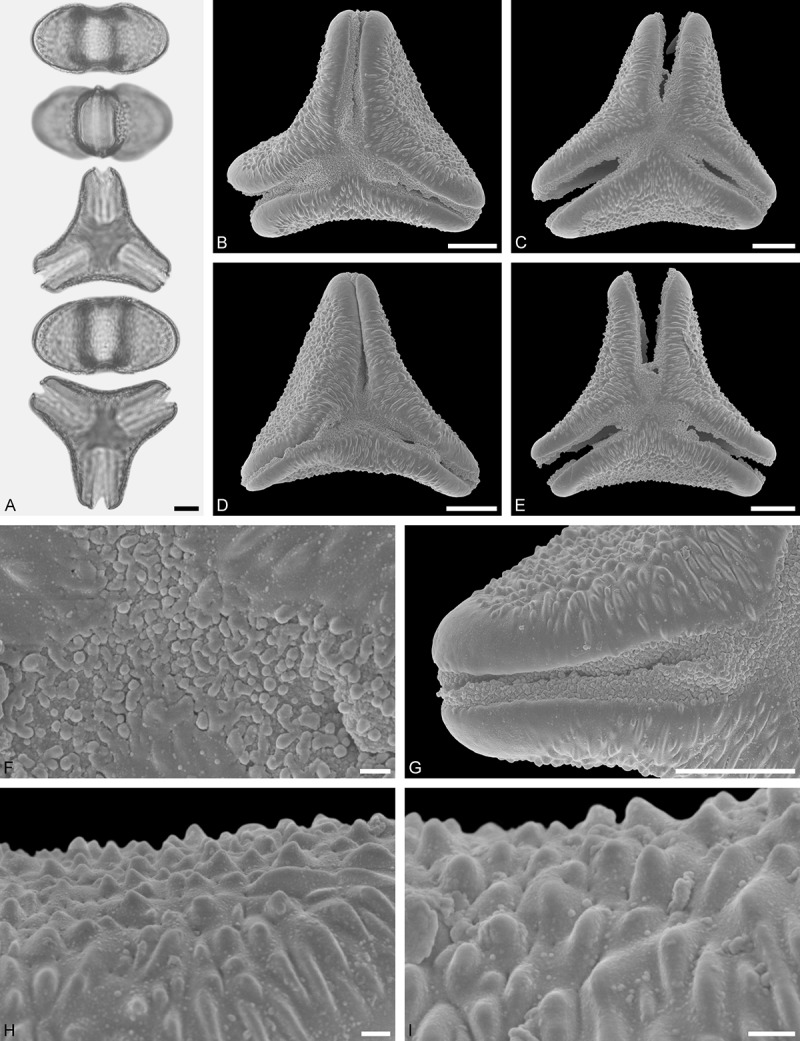
LM (A) and SEM (B–I) micrographs of *Loxanthera speciosa* (WU: from Sumatra, coll. H. O. Forbes, s.n.). **A.** Two pollen grains in equatorial and polar view. **B–E.** Pollen grains in polar view. **F.** Close-up of central polar area. **G.** Close-up of apex. **H, I.** Close-ups of mesocolpium. Scale bars – 10 µm (A–E, G), 1 µm (F, H, I).

**Figure 19.**
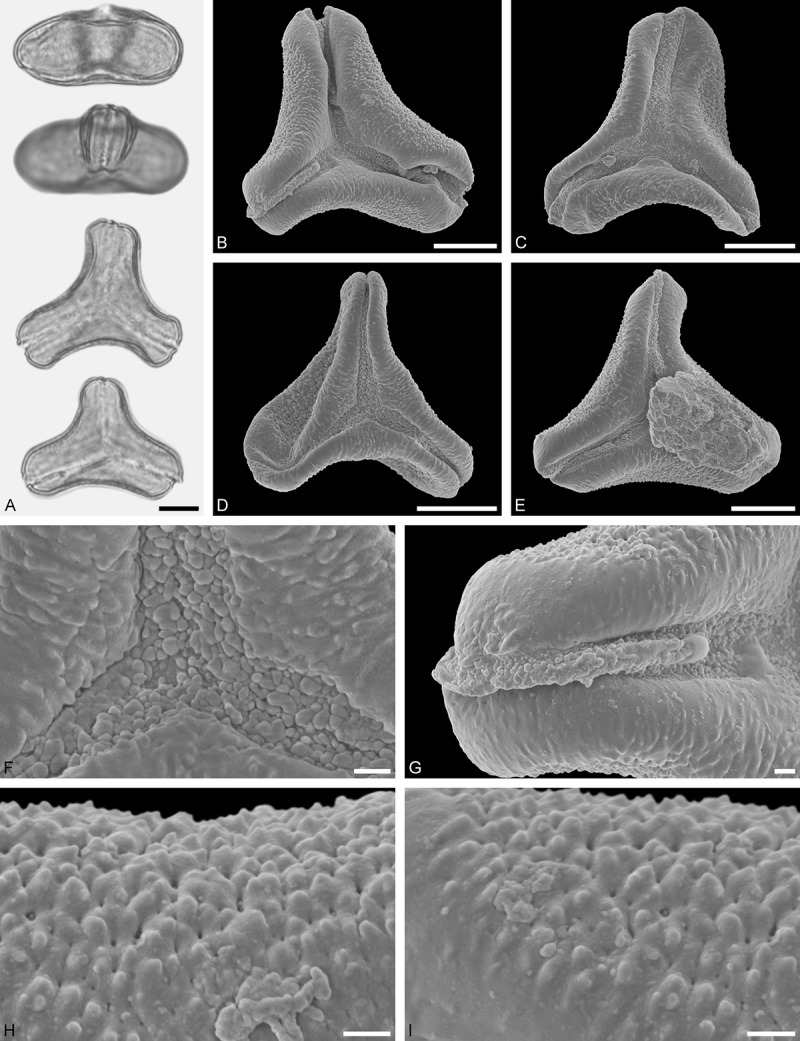
LM (A) and SEM (B–I) micrographs of *Macrosolen cochinchinensis* (WU 039103). **A.** Two pollen grains, upper in equatorial view and lower in polar view. **B–E.** Pollen grains in polar view. **F.** Close-up of central polar area. **G.** Close-up of apex. **H, I.** Close-ups of mesocolpium. Scale bars – 10 µm (A–E), 1 µm (F–I).

**Figure 20.**
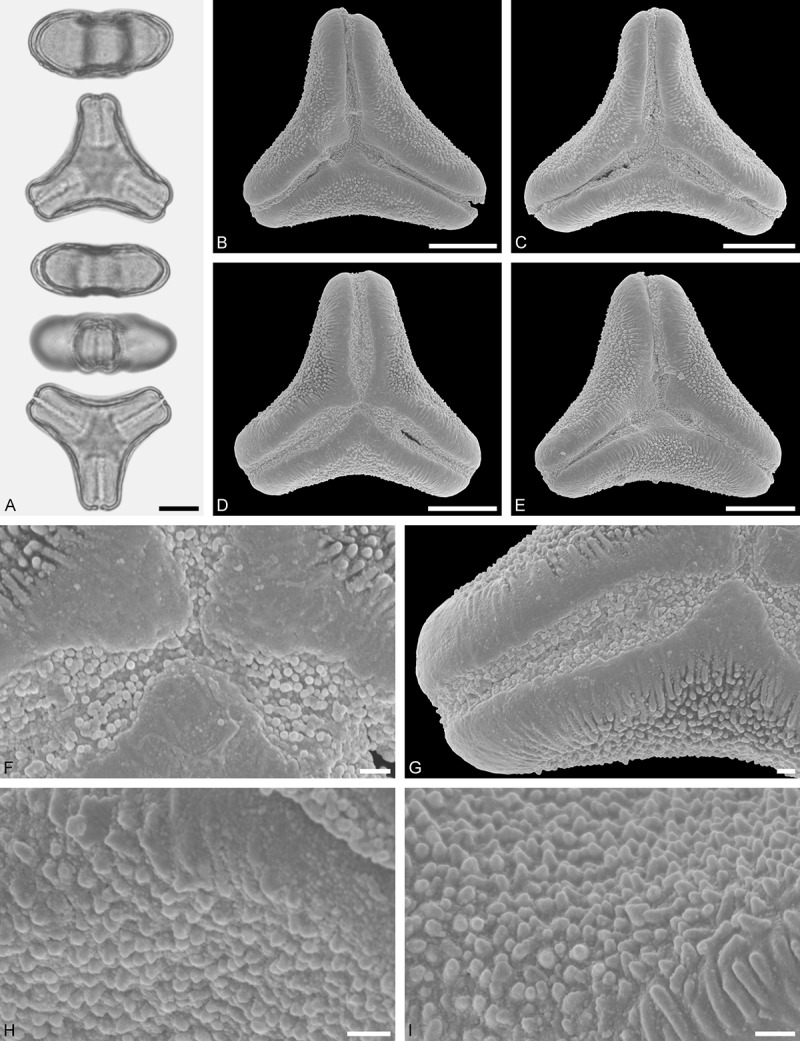
LM (A) and SEM (B–I) micrographs of *Peraxilla colensoi* (WU: from New Zealand, coll. G. Schneeweiß, P. Schönswetter and A. Tribsch, s.n.). **A.** Two pollen grains in equatorial and polar view. **B–E.** Pollen grains in polar view. **F.** Close-up of central polar area. **G.** Close-up of apex. **H, I.** Close-ups of mesocolpium. Scale bars – 10 µm (A–E), 1 µm (F–I).

**Figure 21.**
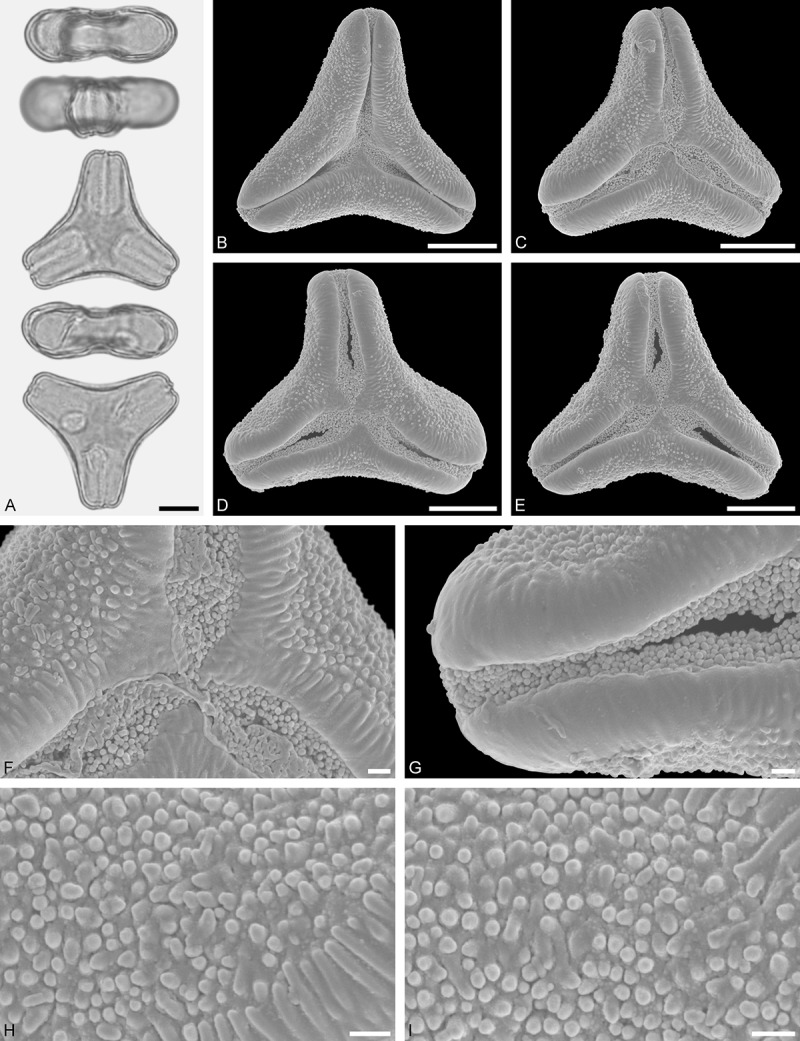
LM (A) and SEM (B–I) micrographs of *Peraxilla tetrapetala* (WU: from New Zealand, coll. G. Schneeweiß, P. Schönswetter and A. Tribsch, s.n.). **A.** Two pollen grains in equatorial and polar view. **B–E.** Pollen grains in polar view. **F.** Close-up of central polar area. **G.** Close-up of apex. **H, I.** Close-ups of mesocolpium. Scale bars – 10 µm (A–E), 1 µm (F–I).

All five genera of the Elytrantheae studied here using LM and SEM (Figure 5: *Amylotheca* [Figure 16], *Alepis* [Figure 17], *Loxanthera* [Figure 18], *Macrosolen* [Figure 19], *Peraxilla* [Figures 20, 21]) share similar pollen of Type B. Pollen grains are syn(3)colpate except for *Amylotheca*, (distinctly) oblate and more or less deeply concave-triangular in polar outline; the equatorial apices are truncated to broadly rounded. The margo is well-developed, encompassing the equatorial apices, and producing three more or less pronounced triangular intercolpial protrusions at the poles, a shared feature of the lineage. Linked to this is that the colpi are typically widening towards the polar area. Another diagnostic SEM feature is that the sculpturing of the margo gradually changes from psilate (the basic sculpturing in all putatively derived Loranthaceae) at the equator to more or less distinctly striate (as in several Loranthaceae of ambiguous affinity; Figure 4; Table III) in the polar area. *Amylotheca*, a genus deeply nested in the clade as sister to *Loxanthera*, differs from the basic type by several characteristics, all of which appear to be directly derived from the basic type seen in the other genera (Figure 5). The pollen of the eight Elytrantheae species including one sp. indet. (two *Amylotheca*, one *Elytranthe*, five *Macrosolen*) figured in Liu and Qiu (1993) and Han et al. (2004) seem to fall within the here documented general type.

Elytrantheae

*Amylotheca* sp.

(Figure 16)

#### Description

Pollen, oblate, concave-triangular in polar view, rhombic (acuminate-obtuse) in equatorial view, equatorial apices rounded; size small, polar axis 10.0–15.0 µm long in LM, equatorial diameter 18.3–21.7 µm in LM, 19.5–20.9 µm in SEM; demisyn(3)colpate, colpi short, 6.7–8.5 µm long in SEM, colpi widening towards polar area; exine 1.0–1.3 µm thick, nexine thinner than sexine, nexine thickened in polar area (LM); tectate; sculpturing psilate in LM, nano-echinate in area of mesocolpium in SEM, echini 0.2–0.6 µm long, 0.2–0.4 µm wide at base; margo well developed, margo psilate, margo with triangular protrusions in polar area; colpus membrane nano-verrucate to nano-echinate (SEM).

#### Remark

Pollen Type B. The unique characteristics of *Amylotheca* pollen within the Elytrantheae include its small size, its rhombic outline in equatorial view, its psilate margo, and that it is demisyncolpate.

*Alepis flavida* Tiegh. (Figure 17)

#### Description

Pollen, distinctly oblate, concave-triangular to trilobate in polar view, elliptic in equatorial view, equatorial apices broadly rounded; size medium, polar axis 18.3–20.0 µm long in LM, equatorial diameter 36.7–43.3 µm in LM, 35.8–41.8 µm in SEM; syn(3)colpate, colpi widening towards polar area; exine 1.3–1.7 µm thick, nexine thinner than sexine, nexine hexagonally thickened in polar area (LM); tectate; sculpturing psilate in LM, rugulate in area of mesocolpium in SEM; margo well developed, margo striate, striae mostly perpendicular to colpi, margo sometimes psilate at equatorial apices, margo with triangular protrusions in polar area; colpus membrane nano-verrucate to nano-echinate (SEM).

#### Remark

Pollen Type B. Pollen of *Alepis* and its sister genus *Peraxilla* are very similar in general morphology (outline, size, thickenings, etc.). A unique feature of *Alepis* pollen is the rugulate sculpturing in the mesocolpium, and linked to this, a distinctly striate margo. The margo is weakly but visibly striate in *Peraxilla*, which has a nano-echinate sculpturing in the area of mesocolpium.

*Elytranthe albida* (Blume) Blume

#### Description (cf. Liu & Qiu 1993, plate I, figures 12–15; Han et al. 2004, figures 13–15)

Pollen, distinctly(?) oblate, concave-triangular in polar view, equatorial view unknown, equatorial apices truncated(?); size medium (to large?; cf. tables in Liu & Qiu 1993; Han et al. 2004); syn(3)colpate; further details not visible.

#### Remark

Pollen Type B. As far as can be judged from the original micrographs, pollen of this species falls within the typical morphology of the Elytrantheae.

*Loxanthera speciosa* Blume (Figure 18)

#### Description

Pollen, oblate, concave-triangular to straight-triangular in polar view, elliptic in equatorial view, equatorial apices broadly rounded; size medium to large, polar axis 30.0–32.0 µm long in LM, equatorial diameter 46.7–58.3 µm in LM, 40.0–51.1 µm in SEM; syn(3)colpate, colpi widening towards polar area; exine 1.3–1.7 µm thick, nexine thinner than sexine, nexine hexagonally thickened in polar area (LM); tectate; sculpturing psilate in LM, micro-echinate to echinate in area of mesocolpium in SEM, echini broadly based, echini 0.3–1.2 µm long, 0.5–1.7 µm wide at base; margo well developed, margo striate to rugulate in polar area, margo psilate at equatorial apices, striae/rugulae perpendicular to colpi, margo with triangular protrusions in polar area; colpus membrane nano-verrucate to nano-echinate (SEM).

#### Remark

Pollen Type B. The pollen stands out within this group because of its size and the size of its sculpturing elements.

*Macrosolen cochinchinensis* (Lour.) Tiegh. (Figure 19)

#### Description

Pollen, oblate, concave-triangular in polar view, elliptic in equatorial view, equatorial apices broadly rounded; size medium, polar axis 18.0–20.0 µm long in LM, equatorial diameter 30.0–35.0 µm in LM, 23.5–32.8 µm in SEM; syn(3)colpate, colpi widening towards polar area; exine 1.2–1.7 µm thick, nexine thinner than sexine (LM); tectate; sculpturing psilate in LM, micro-verrucate to micro-echinate, perforate in area of mesocolpium in SEM; margo well developed, margo psilate and/or indistinctly striate/rugulate, striae/rugulae perpendicular to colpi; colpus membrane nano-verrucate to nano-echinate (SEM).

#### Remark

Pollen Type B. This pollen differs from other pollen of the group by its variable mesocolpial sculpturing (micro-verrucate to -echinate, perforate).

*Peraxilla colensoi* (Hook.f.) Tiegh.

(Figure 20)

#### Description

Pollen, distinctly oblate, concave-triangular in polar view, elliptic in equatorial view, equatorial apices broadly rounded; size medium, polar axis 10.0–16.7 µm long in LM, equatorial diameter 28.3–31.7 µm in LM, 27.6–31.4 µm in SEM; syn(3)colpate, colpi widening towards polar area; exine 1.4–1.8 µm thick, nexine thinner than sexine, nexine hexagonally thickened in polar area, sexine thickened in area of mesocolpium (LM); tectate; sculpturing psilate in LM, nano-echinate in area of mesocolpium in SEM, echini 0.3–0.6 µm long, 0.2–0.5 µm wide at base; margo well developed, margo psilate and/or slightly striate/rugulate, striae/rugulae perpendicular to colpi, margo with prominent triangular protrusions in polar area; colpus membrane nano-verrucate to nano-echinate (SEM). – Pollen Type B.

*Peraxilla tetrapetala* (L.f.) Tiegh. (Figure 21)

#### Description

Pollen, distinctly oblate, concave-triangular in polar view, elliptic in equatorial view, equatorial apices broadly rounded; size medium, polar axis 8.3–11.7 µm long in LM, equatorial diameter 30.0–35.0 µm in LM, 27.1–32.1 µm in SEM; syn(3)colpate, colpi widening towards polar area; exine 1.1–1.5 µm thick, nexine thinner than sexine, nexine hexagonally thickened in polar area (LM); tectate; sculpturing psilate in LM, nano-echinate in area of mesocolpium in SEM, echini 0.3–0.6 µm long, 0.2–0.4 µm wide at base; margo well developed, margo psilate and/or striate/rugulate, striae/rugulae perpendicular to colpi, margo with prominent triangular protrusions in polar area; colpus membrane nano-verrucate to nano-echinate (SEM).

#### Remark

Pollen Type B. The pollen of both investigated species of *Peraxilla* are nearly identical.

Psittacanthinae clade

**Figure 22.**
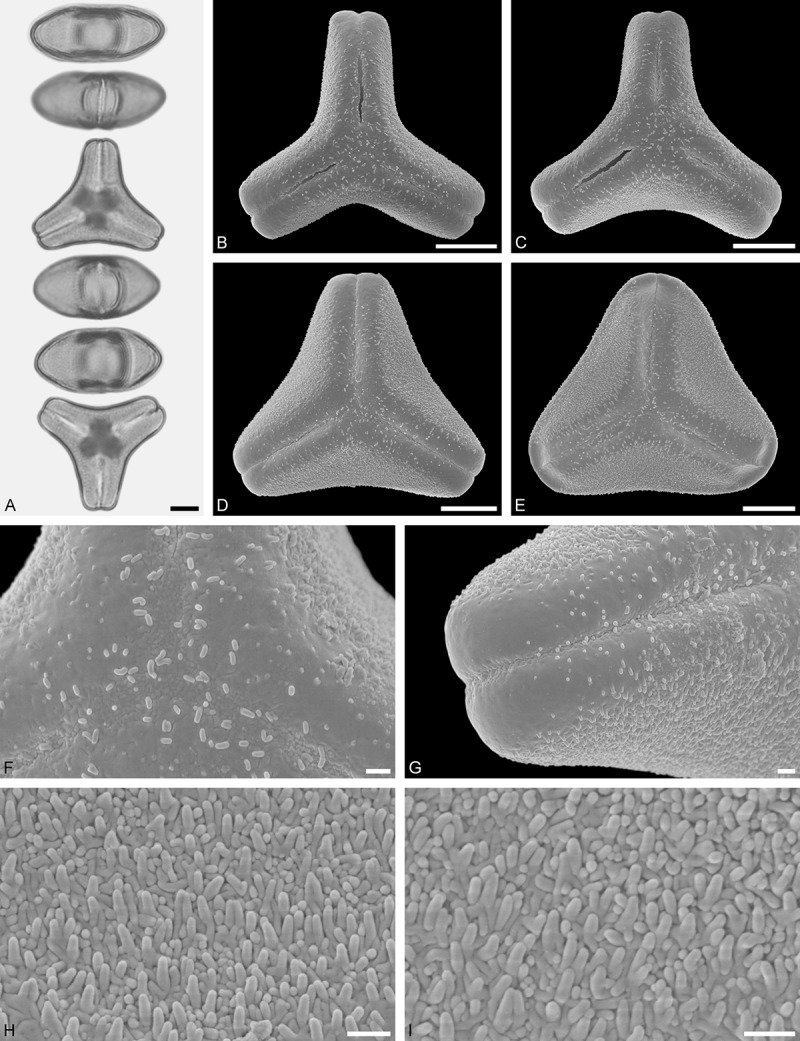
LM (A) and SEM (B–I) micrographs of *Aetanthus coriaeus* (MO: from Ecuador, coll. J. E. Madsen, s.n.). **A.** Two pollen grains in equatorial and polar view. **B–E.** Pollen grains in polar view. **F.** Close-up of central polar area. **G.** Close-up of apex. **H, I.** Close-ups of mesocolpium. Scale bars – 10 µm (A–E), 1 µm (F–I).

**Figure 23.**
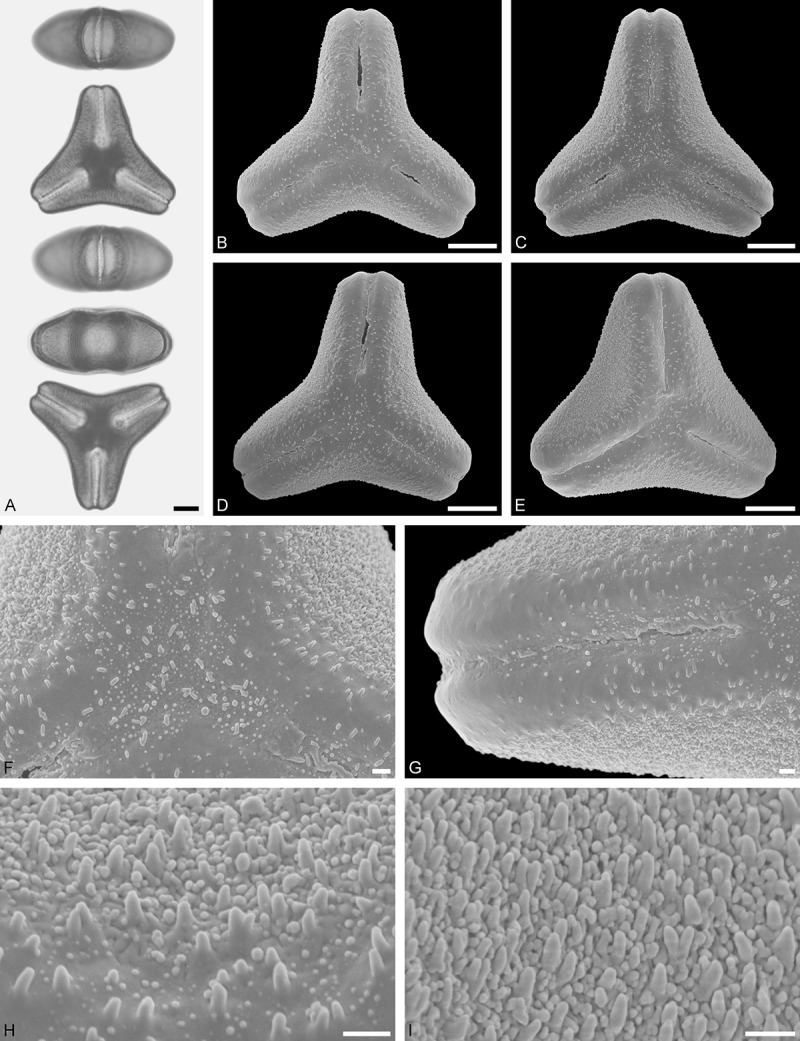
LM (A) and SEM (B–I) micrographs of *Aetanthus macranthus* (MO: from Ecuador, coll. unknown, s.n.). **A.** Two pollen grains in equatorial and polar view. **B–E.** Pollen grains in polar view. **F.** Close-up of central polar area. **G.** Close-up of apex. **H, I.** Close-ups of mesocolpium. Scale bars – 10 µm (A–E), 1 µm (F–I).

**Figure 24.**
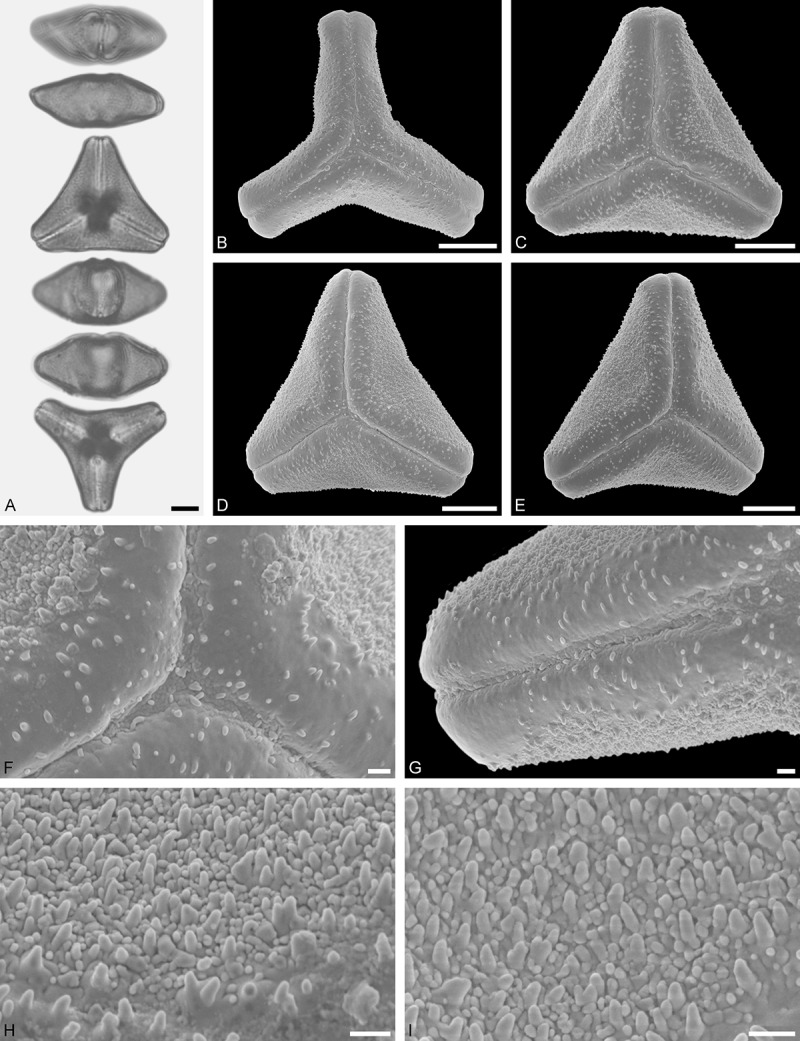
LM (A) and SEM (B–I) micrographs of *Aetanthus nodosus* (MO: from Ecuador, coll. S. González, s.n.). **A.** Two pollen grains in equatorial and polar view. **B–E.** Pollen grains in polar view. **F.** Close-up of central polar area. **G.** Close-up of apex. **H, I.** Close-ups of mesocolpium. Scale bars – 10 µm (A–E), 1 µm (F–I).

**Figure 25.**
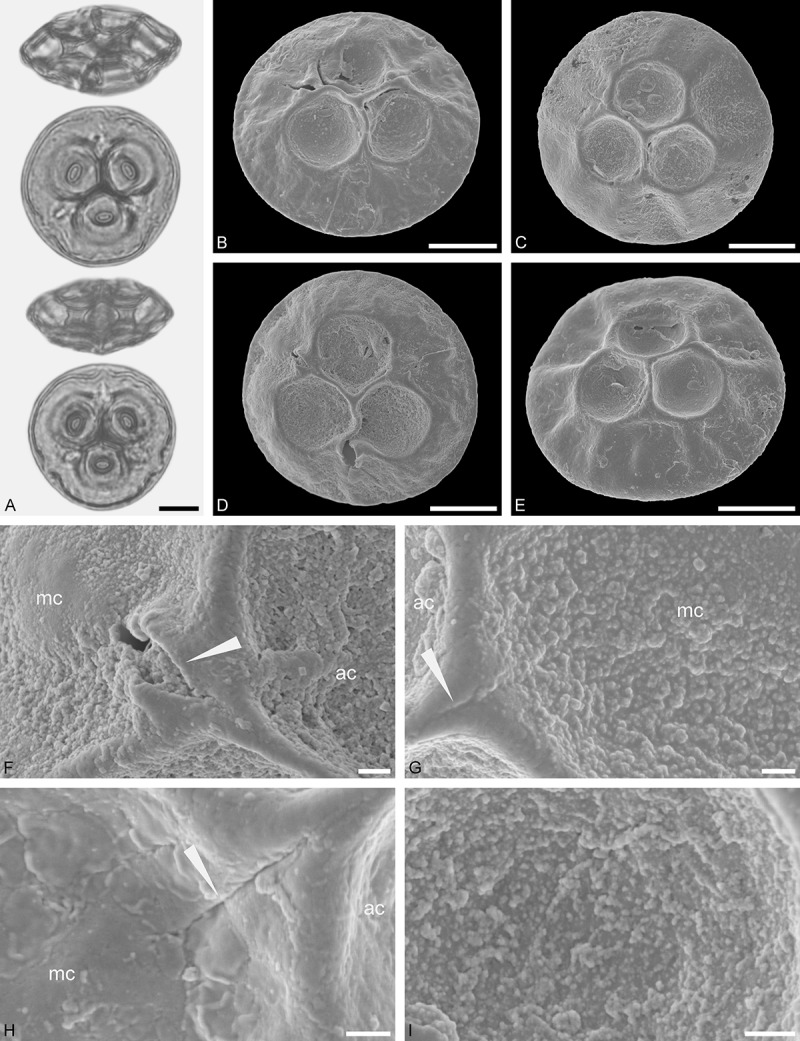
LM (A) and SEM (B–I) micrographs of *Oryctanthus alveolatus* (WU: from Ecuador, coll. J. R. Abbott, s.n.). **A.** Two pollen grains in equatorial and polar view. **B–E.** Pollen grains in polar view. **F, G, H.** Close-ups of lophae junction showing colpus (arrow) and adjacent mesocolpium (mc) and apocolpium (ac). **I.** Close-up of polar lacuna. Scale bars – 10 µm (A–E), 1 µm (F–I).

**Figure 26.**
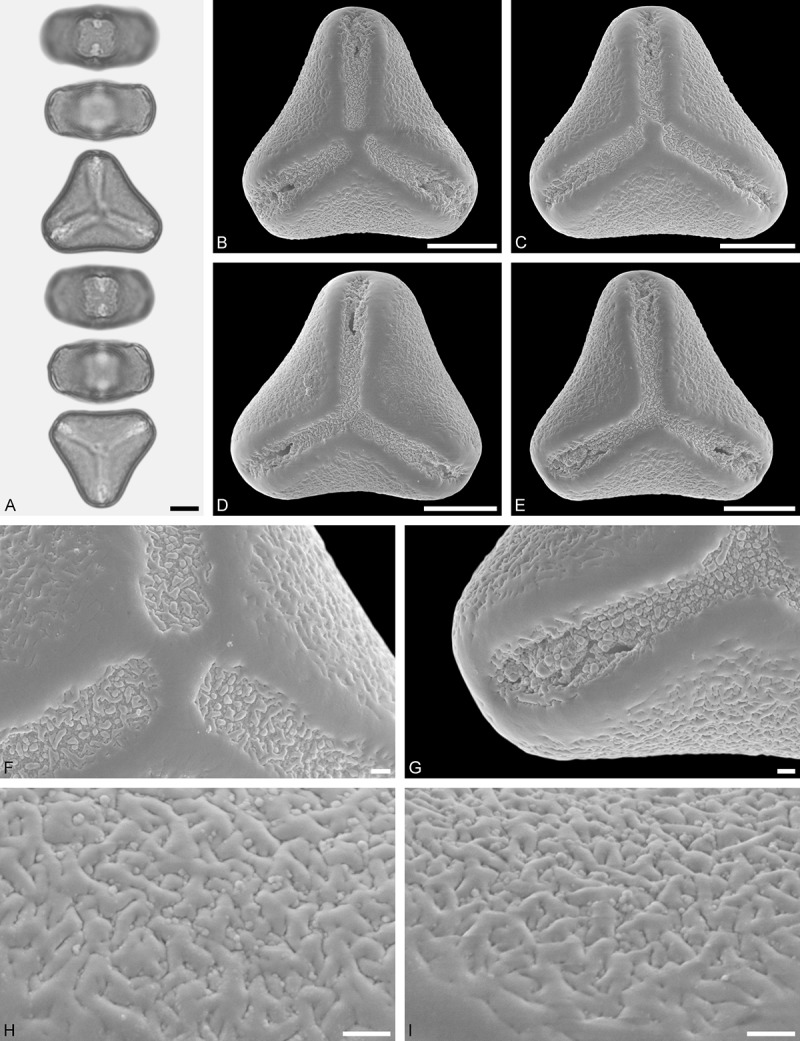
LM (A) and SEM (B–I) micrographs of *Passovia ovata* (MO: from Brazil, coll. J. Ratter, s.n.). **A.** Two pollen grains in equatorial and polar view. **B–E.** Pollen grains in polar view. **F.** Close-up of central polar area. **G.** Close-up of apex. **H, I.** Close-ups of mesocolpium. Scale bars – 10 µm (A–E), 1 µm (F–I).

**Figure 27.**
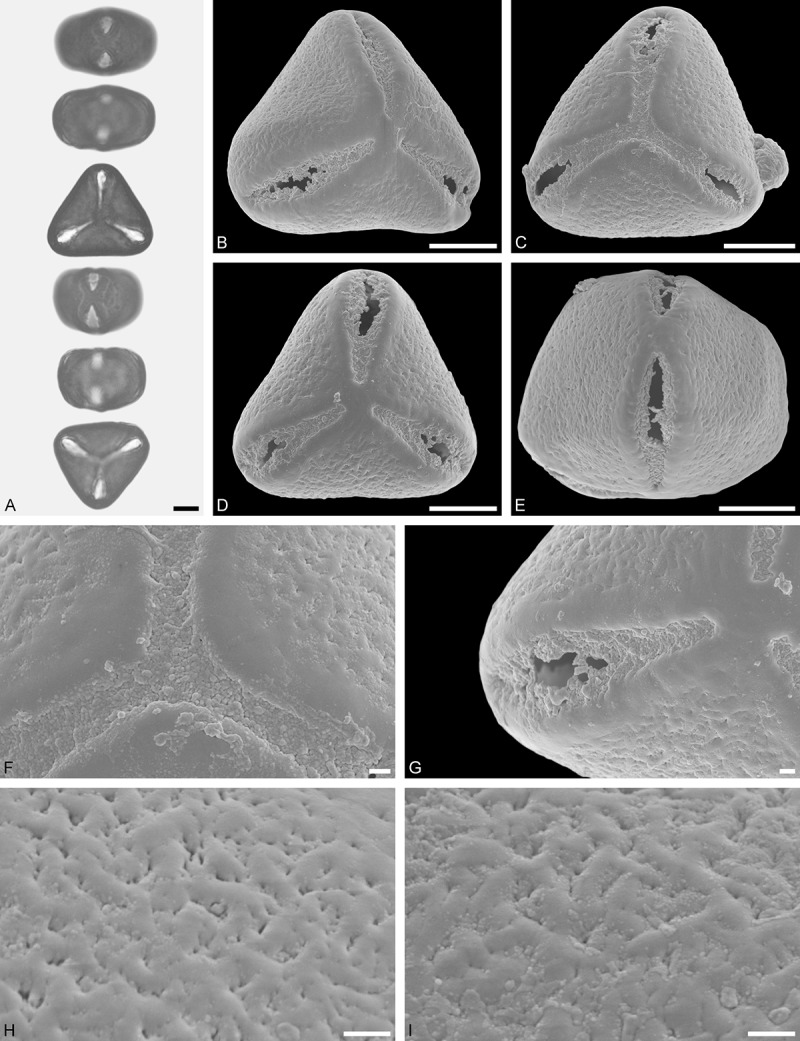
LM (A) and SEM (B–I) micrographs of *Passovia pedunculata* (MO: from Venezuela, coll. Liesner, s.n.). **A.** Two pollen grains in equatorial and polar view. **B–D.** Pollen grains in polar view. **E.** Pollen grain in equatorial view. **F.** Close-up of central polar area. **G.** Close-up of apex. **H, I.** Close-ups of mesocolpium. Scale bars – 10 µm (A–E), 1 µm (F–I).

**Figure 28.**
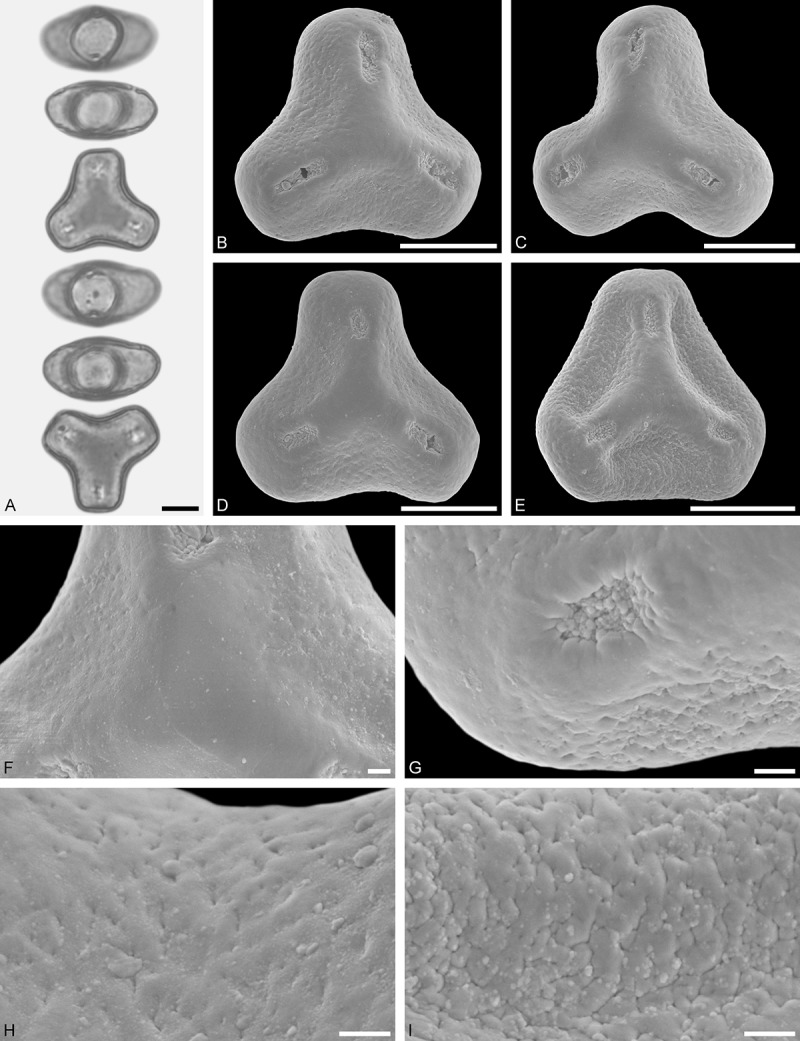
LM (A) and SEM (B–I) micrographs of *Passovia pyrifolia* (MO: from Peru, coll. R. Várgez & N. Jaramillo, s.n.). **A.** Two pollen grains in equatorial and polar view. **B–E.** Pollen grains in polar view. **F.** Close-up of central polar area. **G.** Close-up of apex. **H, I.** Close-ups of mesocolpium. Scale bars – 10 µm (A–E), 1 µm (F–I).

**Figure 29.**
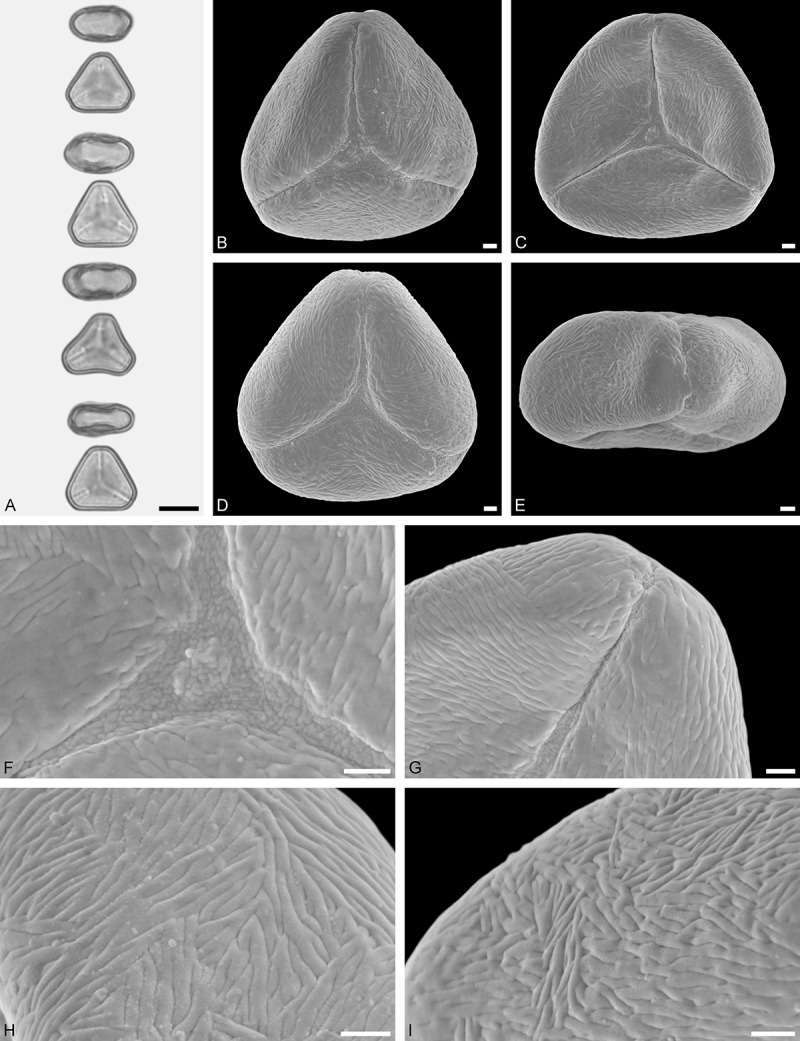
LM (A) and SEM (B–I) micrographs of *Peristethium leptostachyum* (WU 026391). **A.** Four pollen grains in equatorial and polar view. **B–D.** Pollen grains in polar view. **E.** Pollen grain in equatorial view. **F.** Close-up of central polar area. **G.** Close-up of apex. **H, I.** Close-ups of mesocolpium. Scale bars – 10 µm (A), 1 µm (B–I).

**Figure 30.**
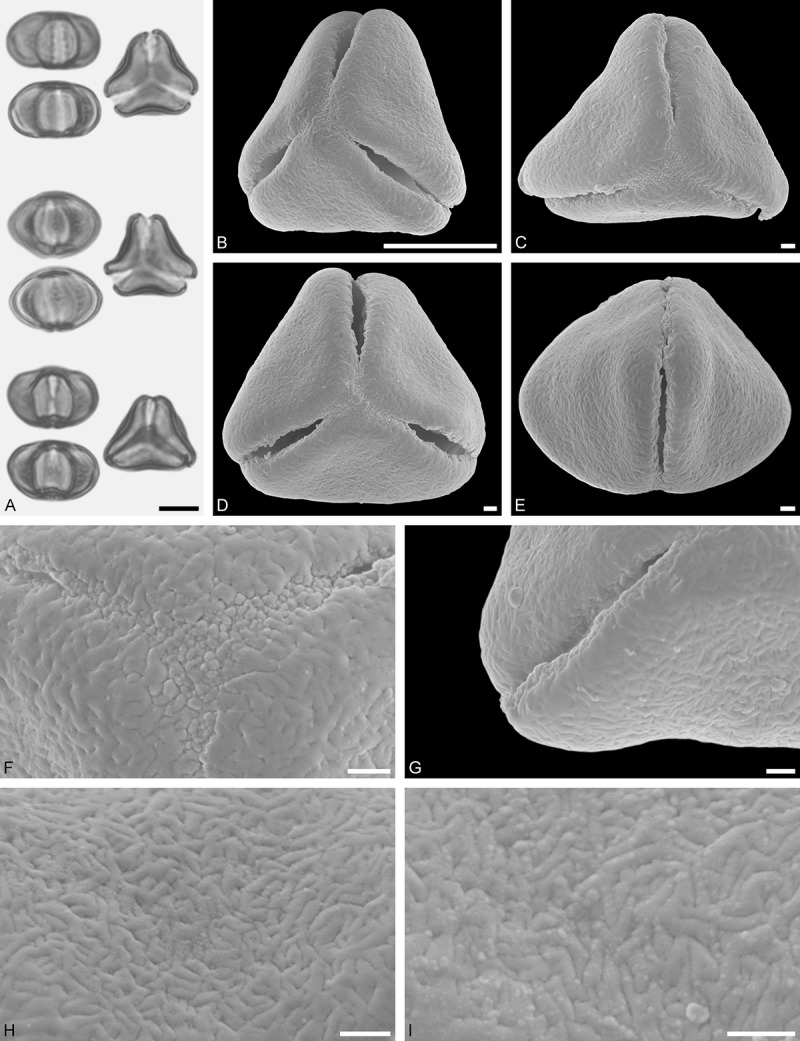
LM (A) and SEM (B–I) micrographs of *Phthirusa clandestina* (MO: from Brazil, coll. Harley et al., s.n.). **A.** Three pollen grains in equatorial and polar view. **B–D.** Pollen grains in polar view. **E.** Pollen grain in equatorial view. **F.** Close-up of central polar area. **G.** Close-up of apex. **H, I.** Close-ups of mesocolpium. Scale bars – 10 µm (A, B), 1 µm (C–I).

**Figure 31.**
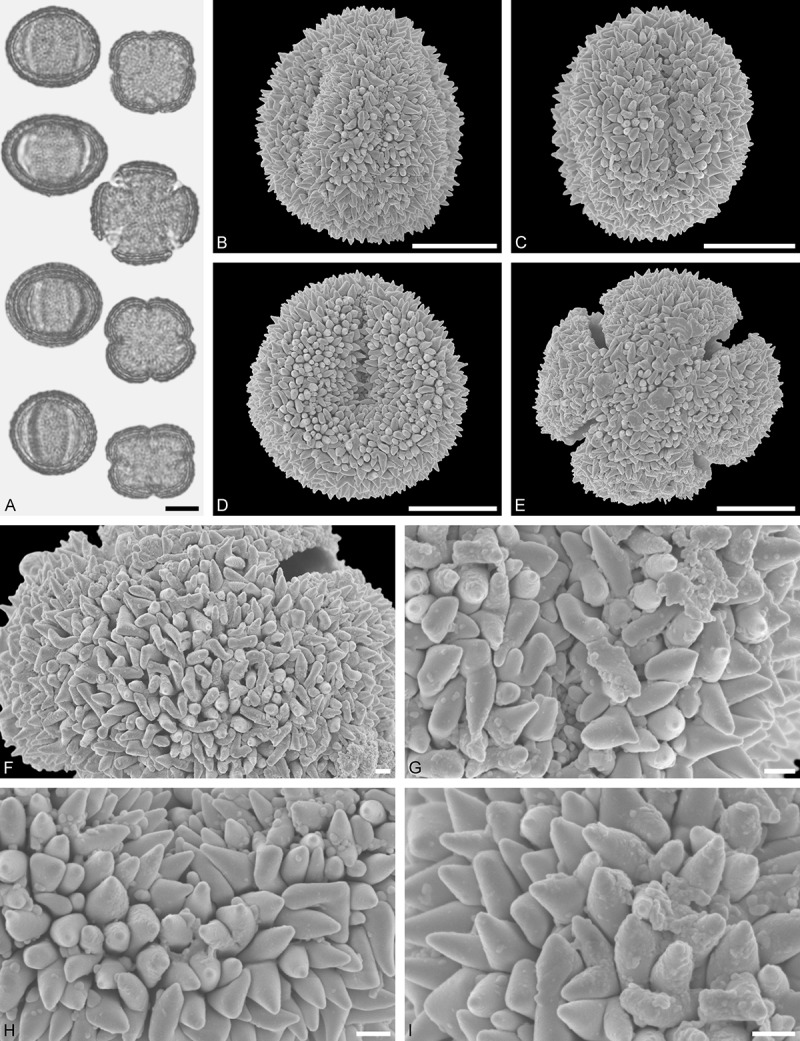
LM (A) and SEM (B–I) micrographs of *Phthirusa hutchisonii* (from Colombia, coll. Hutchinson & Idrobo, MO s.n.). **A.** Four pollen grains in equatorial and polar view. **B–D.** Pollen grains in equatorial view. **E.** Pollen grain in polar view. **F.** Close-up of central polar area. **G.** Close-up of colpi. **H, I.** Close-ups of mesocolpium. Scale bars – 10 µm (A–E), 1 µm (F–I).

**Figure 32.**
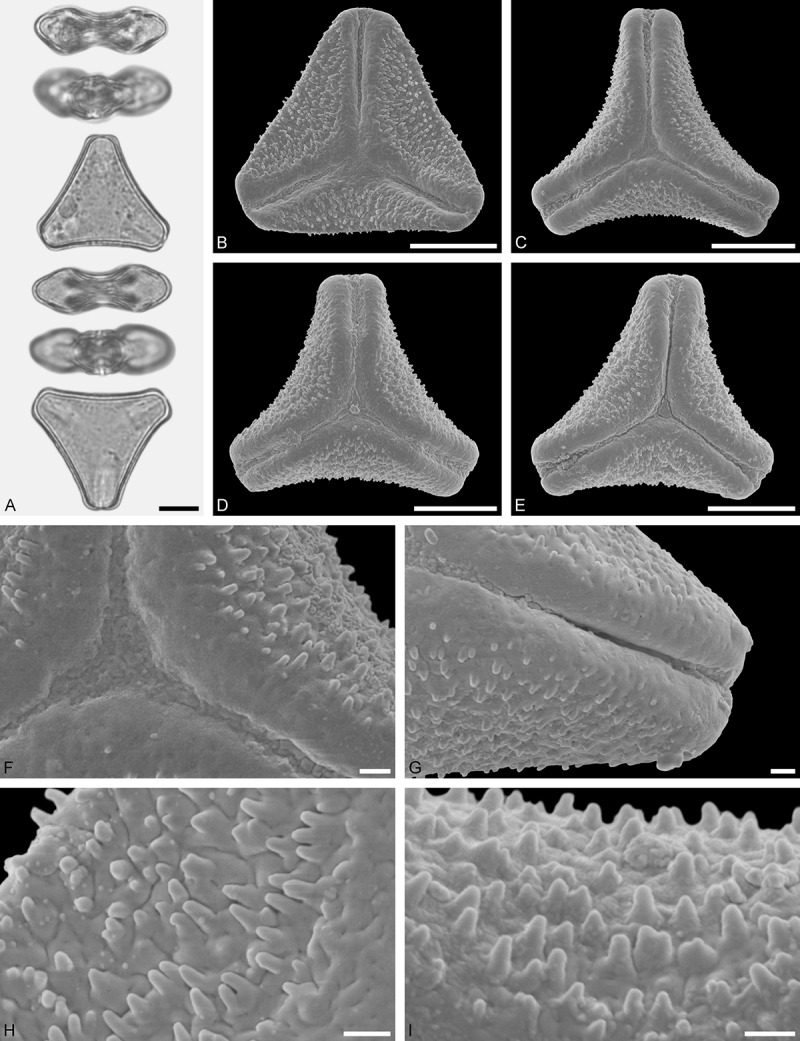
LM (A) and SEM (B–I) micrographs of *Psittacanthus calyculatus* (WU: from Mexico, coll. C. G. Pringle, s.n.). **A.** Two pollen grains in equatorial and polar view. **B–E.** Pollen grains in polar view. **F.** Close-up of central polar area. **G.** Close-up of apex. **H, I.** Close-ups of mesocolpium. Scale bars – 10 µm (A–E), 1 µm (F–I).

**Figure 33.**
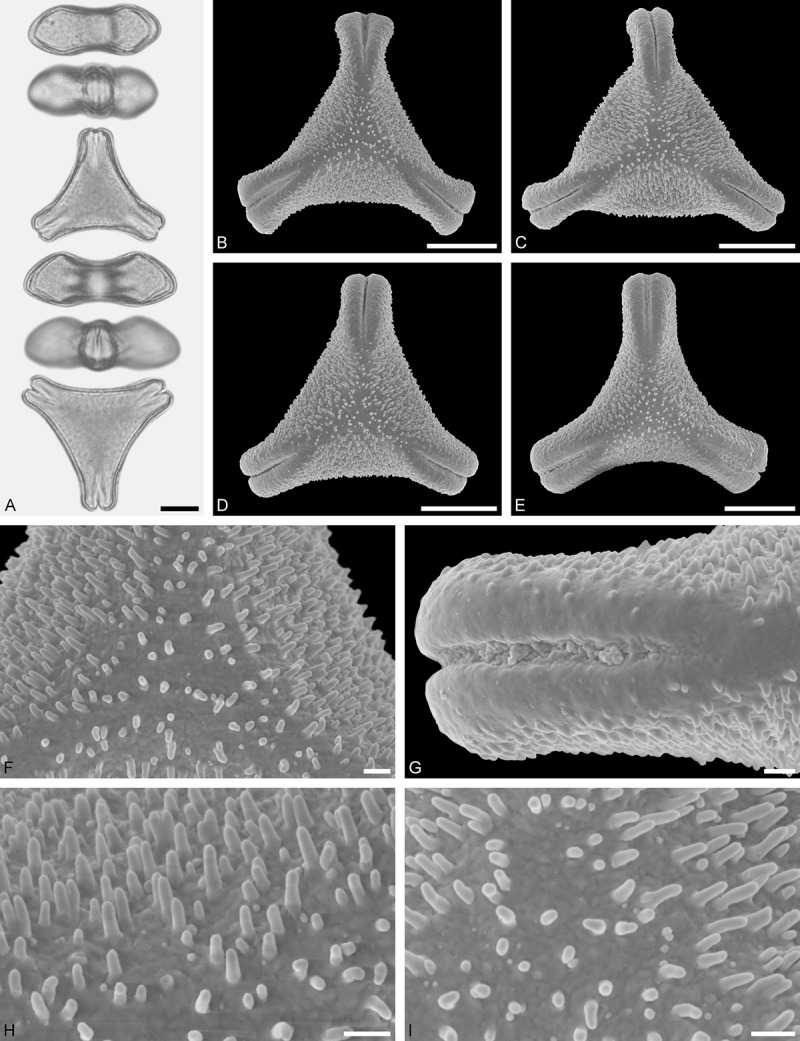
LM (A) and SEM (B–I) micrographs of *Psittacanthus rhynchanthus* (WU 020859). **A.** Two pollen grains in equatorial and polar view. **B–E.** Pollen grains in polar view. **F.** Close-up of central polar area. **G.** Close-up of apex. **H.** Close-up of mesocolpium. **I.** Close-up of central polar area. Scale bars – 10 µm (A–E), 1 µm (F–I).

**Figure 34.**
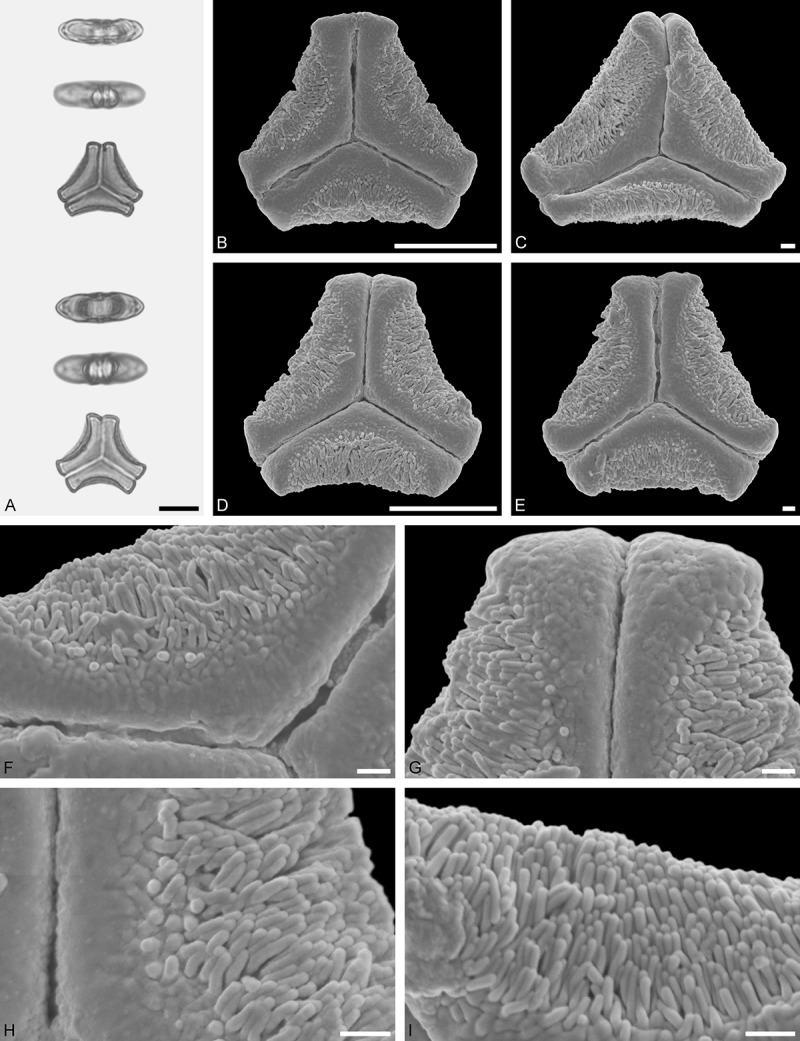
LM (A) and SEM (B–I) micrographs of *Tripodanthus acutifolius* (WU: from Bolivia, coll. N. A. Harriman, s.n.). **A.** Two pollen grains in equatorial and polar view. **B–E.** Pollen grains in polar view. **F.** Close-up of central polar area and mesocolpium. **G.** Close-up of apex. **H, I.** Close-ups of mesocolpium. Scale bars – 10 µm (A, B, D), 1 µm (C, E–I).

The Psittacanthinae show substantial variation in their pollen morphology, covering all four main pollen types, three of which (Type B, C, and D) can be linked with the molecular-phylogenetic framework (Figure 6). The Type D pollen characteristic for *Oryctanthus* is highly distinct in LM and SEM (Figure 25); the same holds for the pollen Type A of *Phthirusa hutchisonii* and pollen Type C of *Dendropemon* and its close relative *Passovia pyrifolia* (Figure 28; Feuer & Kuijt 1985). Also the pollen Type B found in other species of *Passovia* and *Phthirusa* and the remaining genera (Figures 22–24, 26, 27, 29–34) is divers. It includes small to large-sized (rare), oblate to extremely oblate (more than three-times wider than high in equatorial view) pollen grains that vary from (deeply) concave- to convex-triangular outline in polar view. Pollen grains are mostly elliptic in equatorial outline, but can also be (sub-)rhombic (*Struthanthus uraguensis* G.Don, *Tripodanthus belmirensis* F.J.Roldán et Kuijt) or emarginate (several *Psittacanthus* species; Tables V, VI). Aperture organisation is variable between, but also within genera; basically syncolpate, apertures are further modified into zono-, demi-, parasyn- or potentially demisyncolpate (Figure 6). For a few species of *Struthanthus*, heteropolar grains have been documented (*S. dichotrianthus* Eichler) or purported (*S. deppeanus* [Schltdl. et Cham.] G.Don, *S. marginatus* [Desr.] Blume; Feuer & Kuijt 1985). Colpi are narrow to wide, and occasionally widening towards the equatorial apices (*Psittacanthus calyculatus*) or polar area (*Cladocolea*, a few *Struthanthus* spp., less developed in *Peristethium*). The colpi form seems not to be phylogenetically correlated. Traits like a distinct, essentially psilate margo seem however to be restricted to certain genera and subclades of the Psittacanthinae (Figure 6; Tables V–VII). LM images of *Cladocolea* and species of its sister genus *Struthanthus* indicate a narrow-elliptical to U-shaped thinning perpendicular to the colpi in the nexine between the pole and the equator (see ‘Discussion’); in other *Struthanthus* and *Tripodanthus* species the sexine appears to be thickened in the equatorial mesocolpium (Tables V, VI). Polar thickening of inner (observed under LM) and outer exine layers (observed under SEM) is found in species of all genera with pollen Type B. Outer exine thickening manifests either as a polar dome overgrowing the colpi (in a *Passovia* species with pollen Type B synonymised with *Passovia pyrifolia*, which has pollen Type C) or as a more or less pronounced, protruding island in the apocolpial field in grains with parasyncolpate aperture organisation, a feature typical for the *Cladocolea-Struthanthus* lineage (minute in *Peristethium leptostachyum*). The mesocolpium sculpturing is often unclear in previously published images, but anything can be found from psilate or granulate to micro-baculate, -echinate and -verrucate. A unique ornamental feature within the Loranthaceae is the minute rugulate sculpturing found in the mesocolpium of pollen of several *Struthanthus* species (*Struthanthus* ‘Type 3’ in Feuer & Kuijt 1985), which can be rugulate to striate in *Peristethium leptostachyum*, which has been recently moved from *Struthanthus* (Figure 29; Table VI). Sculptural elements (bacula) >1 µm are restricted to pollen of the genus *Tripodanthus* (Figure 34), resolved as sister to all other Psittacanthinae except the extremely long-branching *Phthirusa* (Figures 2, 6).

**Table IV.** Tabulation of differentiating pollen features within Elytrantheae clade.

Genus	Clade	Apertures	Size, shape^a^	Outline in equatorial view	Equatorial apices	Margo	Polar triangular protrusions of margo	Polar thickening of the endexine (LM)	Sculpturing of mesocolpium (SEM)
*Alepis*	A	Syn(3)colpate	Medium, distinctly oblate	Elliptic	Broadly rounded	Striate	**Prominent**	**Hexagonal**	**Rugulate**
*Peraxilla*	A	Syn(3)colpate	Medium, (distinctly) oblate	Elliptic	Broadly rounded	Partly psilate, partly striate/rugulate	**Prominent**	**Hexagonal**	Nanoechinate
*Macrosolen*	B	Syn(3)colpate	Medium, distinctly oblate	Elliptic	Broadly rounded	Mostly psilate, indistinctly striate/rugulate	Indistinct	None	**Micro-verrucate** to -echinate, **perforate**
*Amylotheca*	B	**Demisyn(3)colpate**	**Small**, oblate	**Rhombic**	**Rounded**	**Psilate**	Present	Present	Nano-echinate
*Loxanthera*	B	Syn(3)colpate	Medium to large, oblate	Elliptic	Broadly rounded	Partly psilate, partly striate	**Prominent**	**Hexagonal**	(Micro)**echinate**

Note: ^a^ Size: small, < 25 µm; medium, 25–50 µm; shape: oblate, less than two-times wider than high; distinctly oblate, more than two-times wider than high.

**Table V.** Tabulation of differentiating pollen features in the Psittacanthinae: *Tripodanthus, Aetanthus*, and *Psittacanthus* (Pollen Type B).

Genus	Apertures	Size, shape	Outline in e.v. and p.v.	Equatorial apices	Margo	Exine features (LM)	Sculpturing
*Tripodanthus acutifolius*	Syn(3)colpate, colpi narrow	Small, distinctly oblate	Elliptic, concave-triangular	**Truncated to T-shaped**	Distinct, widening towards equatorial apices	Sexine thickened in MC; nexine triangular thickened in polar area	M: psilate MC: (Micro)**baculate**
*T. belmirensis*	Syn(3)colpate, colpi wide	Small to medium, distinctly oblate	Subrhomic, concave-triangular	Obcordate	Distinct	?	M: psilate ? MC: micro-verrucate ?
*T. flagellaris*	Syn(3)colpate, colpi narrow	Small, distinctly oblate	Not observed, concave-triangular	**Truncated**	Distinct, of equal width	?	M: psilate; MC: (Micro)**baculate**
*Aetanthus coriaceus, A. macranthus, A. nodosus*	Syn(3)colpate	Medium, oblate	Elliptic, straight- or concave-triangular to trilobate	Rounded to obcordate	Distinct	**Hexagonally thickened in polar area**	M: mostly psilate, partly nano-/micro-baculate MC: nano-/micro-baculate
*Psittacanthus acinarius*	**Zono(3)colpate**, colpi **short** and narrow	Medium, distinctly oblate	**Emarginate, convex-triangular**	Obcordate	Distinct	?	**Granulate**
*P. calyculatus, P. columbianus*, *P. clusiifolius, P. macranthus, P. schiedeanus, P. sonorae*	Syn(3)colpate, colpi narrow or wide	Small, medium, or large, (distinctly) oblate	Emarginate or elliptic, convex-(*P. clusiifolius)*, straight- or concave triangular or trilobate	Obcordate or truncated (*P. calyculatus, P. clusiifolius)*	More or less distinct, widening towards equatorial apices (*P. calyculatus*)	None or ?	M: psilate if distinct or as MC; MC: nano- to micro-echinate
*P. cucullaris, P. dilatatus, P. rhynchanthus*	**Zono(3)colpate**, colpi narrow or lense-like, and medium-long	Medium or large, (distinctly) oblate	Emarginate or elliptic, concave-triangular to (modified) trilobate	Obcordate or **rounded** (*P. dilatatus)*	Distinct	None or ?	M: psilate; MC: nano- to micro-baculate/-echinate; AC: **As MC, but SE less dense**
*P. hamulifer, P. peronopetalus*	**Demi(3)colpate**, colpi narrow and medium-long	Large, distinctly oblate		**Rounded**	Not visible	?	Uniform: micro-echinate
*P. robustus*	Syn(3)colpate, medium-wide	Medium, oblate	Elliptic, **convex-triangular**	Obcordate	?	?	M: ? MC: psilate, partly granulate; **minutely perforate**

Note: Abbreviations: e.v., equatorial view; p.v., polar view; M, margo; MC, mesocolpium; AC, apocolpium; SE, sculptural elements.

**Table VI.** Tabulation of differentiating pollen features in the Psittacanthinae: *Cladocolea-Struthanthus* lineage (including *Peristethium;* pollen Type B, pollen without margo).

Genus	Apertures	Polarity, size, shape	Outline in e.v. and p.v.	Equatorial apices	Special exine features (LM)	Pole	Sculpturing
*Cladocolea andrieuxii, C. loniceroides, C. microphylla*	**Demiparasyn(3)colpate**, colpi wide or widening towards equatorial apices	Isopolar, medium, oblate where observed	Elliptic, straight- to broadly convex-triangular	**Rounded**	**Nexine thinning perpendicular to colpi, triangular polar thickening**	**Protruding apocolpial field**	?
*C. glauca, C. pringlei*	**Parasyn(3)colpate**, colpi narrow or wide	Isopolar, medium, oblate where observed	Elliptic, straight- or convex-triangular	Obcordate	**Nexine thinning perpendicular to colpi, triangular polar thickening**	**Protruding apocolpial field**	?
*C. micrantha* (formerly *Phthirusa*)	**(Demi?)syn(3)colpate, colpi taperings towards pole and equator**	Isopolar, small to medium, shape not reported	Straight-triangular (e.v. not available)	**Rounded**	**?**	Indifferent	?
*Struthanthus dichotrianthus, S. quercicola, S. deppeanus, S. marginatus*	**Parasyn(3)colpate**^a^, colpi wide or widening towards equatorial apices	Iso- or **heteropolar**, medium, oblate where observed	Elliptic, straight- to convex-triangular	Obcordate or rounded	**Nexine thinning perpendicular to colpi, triangular polar thickening**	**Protruding apocolpial field**	?
*S. hartwegii*	**Demisyn(3)colpate**, colpi wide	Isopolar, small to medium, shape not reported	Straight-triangular (e.v. not available)	**Rounded**	**Nexine thinnings perpendicular to colpi, triangular polar thickening**	Indifferent	?
*S. concinnus*	Syn(3)colpate, colpi narrow	Isopolar, medium, shape not reported	Straight-triangular (e.v. not available)	Obcordate	None	**Triradiate triangular polar thickenings**	**Rugulate**
*S. marginatus*^b^	Syn(3)colpate, colpi widening towards polar area	Isopolar, medium, distinctly oblate	Slightly emarginate, straight-triangular	Obcordate	**Equatorial mesocolpium (sexine) and polar area (nexine) thickened**	**Triradiate triangular polar thickenings**	**Rugulate**
*S. uraguensis*	Syn(3)colpate, colpi wide	Isopolar, oblate	Subrhombic, slightly convex-triangular	Obcordate	**Equatorial mesocolpium (sexine) thickened**	Indifferent	**Rugulate**, rugulae forming mesocolpial clusters
*Peristethium leptostachyum* (formerly *Struthanthus*)	(**Demi**)syn(3)colpate, colpi narrow	Isopolar, small, oblate	Elliptic, straight- to slightly convex-triangular	**Broadly rounded**	**Triangular polar nexine thickening**	**Minute protruding island**	Rugulate, partly striate

Note: Abbreviations: e.v., equatorial view; p.v., polar view; ^a^ Face parasyncolpate with small apocolpial field; counterface essentially syncolpate, colpi interrupted at pole by triangular extrusions; ^b^ Specimen referred to as *S. vulgaris.*

*Aetanthus coriaceus* Patsch. (Figure 22)

#### Description

Pollen, oblate, trilobate to concave-triangular in polar view, elliptic in equatorial view, equatorial apices obcordate to rounded; size medium, polar axis 20.0–23.3 µm long in LM, equatorial diameter 36.6–45.0 µm in LM, 31.9–43.5 µm in SEM; syn(3)colpate; exine 1.3–1.6 µm thick, nexine thinner than sexine, nexine hexagonally thickened in polar area (LM); tectate; sculpturing psilate in LM, nano-baculate to micro-baculate in area of mesocolpium in SEM, bacula 0.3–0.9 µm long, 0.2–0.4 µm wide at base; margo well developed, margo mostly psilate, partly nano-/micro-baculate (SEM). – Pollen Type B.

*Aetanthus macranthus* (Hook.) Kuijt (Figure 23)

#### Description

Pollen, oblate, trilobate to concave-triangular in polar view, elliptic in equatorial view, equatorial apices obcordate; size medium, polar axis 23.3–26.7 µm long in LM, equatorial diameter 45.0–48.3 µm in LM, 39.0–45.0 µm in SEM; syn(3)colpate; exine 1.2–1.7 µm thick, nexine thinner than sexine, nexine hexagonally thickened in polar area (LM); tectate; sculpturing psilate in LM, nano-baculate to micro-baculate in area of mesocolpium in SEM, bacula 0.3–0.8 µm long, 0.2–0.4 µm wide at base; margo well developed, margo mostly psilate, partly nano-/micro-baculate (SEM). *–* Pollen Type B.

*Aetanthus nodosus* (Desr.) Engl. (Figure 24)

#### Description

Pollen, oblate, trilobate to straight-triangular in polar view, elliptic in equatorial view, equatorial apices obcordate to rounded; size medium, polar axis 18.3–25.0 µm long in LM, equatorial diameter 38.3–45.0 µm in LM, 31.5–40.0 µm in SEM; syn(3)colpate; exine 1.3–1.7 µm thick, nexine thinner than sexine, nexine hexagonally thickened in polar area (LM); tectate; sculpturing psilate in LM, nano-baculate to micro-baculate in area of mesocolpium in SEM, bacula 0.3–0.8 µm long, 0.2–0.5 µm wide at base; margo well developed, margo mostly psilate, partly nano-/micro-baculate (SEM).

#### Remark

Pollen Type B. The pollen of all three investigated *Aetanthus* species are very similar. In a larger taxonomic context, the pollen better fits with the systematic placement as member of the Psittacanthinae than as sister to *Desmaria* (Notantherinae, Figures 2, 3), a placement based exclusively on its 18S rDNA data. Within the Psittacantheae clade, the pollen of *Aetanthus* supports a closer relationship to *Psittacanthus* and *Tripodanthus*, which is in line with molecular trees relying on *mat*K data (File S1; Vidal-Russell & Nickrent 2008b, figure 2; Su et al. 2015).

*Cladocolea* spp. (Table VI)

#### General description based on the species figured in Feuer and Kuijt (1985; see Files S3, S4)

Pollen, oblate, straight- to convex-triangular in polar view, elliptic in equatorial view, equatorial apices rounded or obcordate; size medium (cf. Feuer & Kuijt 1985, table 1); (demi)parasyn(3)colpate, potentially demisyn(3)colpate in *Cladocolea micrantha* (Eichler) Kuijt; parasyncolpate grains typically with nexine thinning perpendicular to colpi and triangular polar thickening; tectate; sculpturing psilate in LM, not clear in SEM; parasyncolpate pollen with a protruding apocolpial field (SEM).

#### Remark

Pollen Type B. From its overall appearance and lacking all unique features of other species of the genus, the pollen of *Cladocolea micrantha* (a species formerly included in *Phthirusa*) would better fit within the variation seen in other genera of Psittacanthinae (*Struthanthus*, *Passovia*).

*Dendropemon* spp. (Table VII)

**Table VII.** Tabulation of differentiating pollen features in the Psittacanthinae: the remaining genera.

Genus	PT	Apertures	Size, shape, outline in e.v. and p.v.	Equatorial apices	Margo	Further exine features (LM, SEM, TEM)	Sculpturing (SEM)
*Phthirusa hutchisonii*	**A**	**Zono(4)colpate^a^**	Small to medium, **± spheroidal**	N/A	None	None	**Echinate**
*P. clandestina*	B	Syn(3)colpate	Small, oblate, broadly elliptic, straight-triangular	Broadly rounded to obcordate	Distinct, micro-rugulate to psilate, **perforate**	LM: sexine thickened in mesocolpium, nexine hexagonally thickened in polar area	**Micro-rugulate**
*P. inconspicua*	B	(Demi?)syn(3)colpate	Small, distinctly oblate, equatorial outline unknown, straight-triangular	Truncated	?	?	?
*Maracanthus*	B	Syn(3)colpate	Medium, oblate, elliptic, straight-triangular	Obcordate	?	LM: Nexine thickened in polar area	?
*Oryctina*^b^	B	(Demi)syn(3)-colpate	Small, shape and equatorial outline unknown, straight- or convex-triangular	Broadly rounded or obcordate (*O. scabrida*)	?	**LM: Triradial nexine thickening in polar area**	?
*Panamanthus*	C?	Possibly **demi(3)colpate**	Medium, shape and equatorial outline unknown, concave-triangular to broadly trilobate	Broadly Rounded	?	LM: Polar nexine thickened	?
‘Type 2’: *Passovia coarctata, P. ovata, P. lepidobotrys, P. pedunculata*	B	Demi(syn)(3)-colpate	Mostly medium, oblate, broadly elliptic (can be subrhombic)	Broadly rounded	Distinct, psilate	LM: nexine (triangular) thickend SEM: Raised polar area (*P. lepidobotrys*) TEM: polar ect- and entexine thickened (*P. ovata)*	**(Micro)rugulate, fossulate, perforate**
‘Type 1’: *Passovia pyrifolia* s.str., *P. platyclada*	**C**	**Demi(3)colpate**	Small to medium, oblate, ± subrhombic, broadly trilobate	Broadly rounded	Distinct, psilate	**LM: Triradial nexine thickening in polar area**	(**Micro)rugulate, fossulate, perforate**
*Dendropemon*	**C**	**Demi(3)colpate**	Medium, oblate, subrhombic?, broadly trilobate	Broadly rounded	?	**LM: Triradial nexine thickening in polar area**	?
*Oryctanthus*	**D**	**Demi(3)colpate, colpi indistinct**	Medium, oblate, elliptic, **(sub)circular**	N/A	None	Highly ornamented (see Figure 25)	Lophate

Note: Abbreviations: e.v., equatorial view; p.v., polar view; N/A, not applicable; LM, light microscopy; SEM, scanning electron microscopy; TEM, transmission electronic microscopy; ^a^ Feuer and Kuijt (1985) reported also 3- or 5-colpate grains; ^b^ *Oryctanthus scabridus* (*=Oryctina scabrida*) included here following Caires (2012) and evidence from pollen.

#### General description based on the species figured in Feuer and Kuijt (1985; see Files S3, S4)

Pollen, oblate, broadly trilobate in polar view, subrhombic (?) in equatorial view, equatorial apices broadly rounded; size medium (cf. Feuer & Kuijt 1985, table 1); demi(3)colpate with short and wide colpi; triradial nexine thickening in polar ares (LM; also seen in TEM); tectate; sculpturing psilate in LM, not clear in SEM.

#### Remark

Pollen Type C. The pollen of *Dendropemon* is readily distinct from most other Loranthaceae due to its unique apertures and shape. Only pollen of *Passovia pyrifolia* is somewhat similar (Figure 28; Feuer & Kuijt 1985).

*Maracanthus chlamydatus* (Rizzini) Kuijt

(Table VII)

#### Description (cf. Feuer & Kuijt 1985, figures 4, 50, 53, 67)

Pollen, oblate, straight-triangular in polar view, elliptic in equatorial view, equatorial apices obcordate; size medium (cf. Feuer & Kuijt 1985, table 1); syn(3)colpate with short and wide colpi; nexine thickened in polar ares (LM); tectate; sculpturing psilate in LM, not clear in SEM, but minute, perforate (TEM).

#### Remark

Pollen Type B. Nickrent et al. (2010) included this genus in *Oryctina*.

*Oryctanthus alveolatus* (Kunth) Kuijt (Figure 25; Table VII)

#### Description

Pollen, oblate, (sub-)circular in polar view, elliptic in equatorial view; size medium, polar axis 16.7–21.7 µm long in LM, equatorial diameter 33.3–41.6 µm in LM, 32.2–35.7 µm in SEM; demi(3)colpate, colpi very narrow; exine 1.1–1.6 µm thick, nexine thinner than sexine (LM); pollen lophate, apocolpium with triradial symmetry, apocolpial lophae surrounding three large (intercolpial) lacunae, radial lophae straight and joining at the pole, mesocolpial lophae irregularly spaced and decreasing in height towards the ridged equator (LM, SEM); tectate; sculpturing psilate in LM, mostly psilate with granulate patches in SEM.

#### Remark

Pollen Type D. The unique pollen of *Oryctanthus* cannot be confused with any other angiosperm, neither in LM nor SEM. Caires (2012) figured pollen from 11 species of the genus.

*Oryctina* spp. (Table VII)

#### General description based on the two species figured in Feuer and Kuijt (1985, LM) and Caires (2012, SEM)

Pollen, shape unknown, straight- or convex-triangular in polar view (no equatorial view available), equatorial apices broadly rounded or obcordate; size small; demi(?)syn(3)colpate, colpi narrow; triradial thickening of nexine in polar area (LM); tectate; sculpturing psilate in LM, unclear (uniform?) in SEM.

#### Remark

Pollen Type B. At the moment little can be said about the morphological affinity of *Oryctina* pollen because of the lack of published material. From the available micrographs *Oryctina* pollen is more similar to that of *Struthanthus* or pollen Type B of *Passovia* than those of the other genera.

*Panamanthus panamensis* (Rizzini) Kuijt (Table VII)

#### Description (cf. Feuer & Kuijt 1985, figures 19, 79, 90–92)

Pollen, oblate, concave-triangular to broadly lobate in polar view (no equatorial view available), equatorial apices broadly rounded; size medium; demi(3)colpate, colpi short and narrow, nexine thickened in polar area (LM); tectate; sculpturing psilate in LM, unclear in SEM.

#### Remark

Possibly pollen Type C. Except for its narrow colpi, the grain resembles the grains of *Passovia pyrifolia* and *Dendropemon* (to a lesser degree), and may represent an intermediate form between Type B and Type C or a less derived variant of Type C. Further palynological and genetic investigations are warranted, but the pollen can be used as argument against including this monotypic genus in *Struthanthus*.

*Passovia ovata* (DC.) Kuijt (Figure 26; Table VII)

#### Description

Pollen, oblate, concave-triangular in polar view, broadly elliptic in equatorial view, equatorial apices broadly rounded; size medium, polar axis 20.0–23.3 µm long in LM, equatorial diameter 33.3–36.7 µm in LM, 29.6–33.6 µm in SEM; demisyn(3)colpate to demi(3)colpate, colpi long and wide; exine 1.3–1.7 µm thick, nexine thinner than sexine (LM); tectate; sculpturing psilate in LM, micro-rugulate to rugulate, fossulate, and perforate in area of mesocolpium in SEM; margo well developed, margo psilate; colpus membrane nano-verrucate to nano-echinate (SEM). – Pollen Type B.

*Passovia pedunculata* (Jacq.) Kuijt (Figure 27; Table VII)

#### Description

Pollen, oblate, straight-triangular to convex-triangular in polar view, broadly elliptic in equatorial view, equatorial apices broadly rounded; size medium, polar axis 25.0–28.3 µm long in LM, equatorial diameter 35.0–40.0 µm in LM, 31.3–35.4 µm in SEM; demisyn(3)colpate to demi(3)colpate, colpi long and wide; exine 1.2–1.6 µm thick, nexine thinner than sexine, nexine thickened in polar area (LM); tectate; sculpturing psilate in LM, micro-rugulate to rugulate, fossulate, and perforate in area of mesocolpium in SEM; margo well developed, margo psilate; colpus membrane nano-verrucate to nano-echinate (SEM).

#### Remark

Pollen Type B. The pollen is highly similar to that of *Passovia ovata* (Figure 26), possibly also *P. coarctata* (A.C.Smith) Kuijt and *P. lepidobotrys* (Griseb.) Kuijt (cf. Feuer & Kuijt 1985, figures 1, 51, 52), but markedly different from *P. pyrifolia* (Figure 28).

*Passovia pyrifolia* (Kunth.) Tiegh. (Figure 28; Table VII)

#### Description

Pollen, oblate, broadly trilobate in polar view, elliptic to subrhombic in equatorial view, equatorial apices broadly rounded; sizes mall to medium, polar axis 15.0–20.0 µm long in LM, equatorial diameter 25.0–28.3 µm in LM, 20.4–23.7 µm in SEM; demi(3)colpate, colpi short and wide, colpi 2.2–5.1 µm long in SEM; exine 1.1–1.5 µm thick, nexine thinner than sexine, triradial thickening of nexine in polar area (LM); tectate; sculpturing psilate in LM, micro-rugulate to rugulate, fossulate, and perforate in area of mesocolpium in SEM; margo well developed, margo psilate; colpus membrane nano-verrucate to nano-echinate (SEM).

#### Remark

Pollen Type C. The pollen of *Passovia pyrifolia* (including *Phthirusa platyclada*, but not *Passovia lepidobotrys*; cf. Kuijt 2011; Tropicos.org 2016) is unlike the pollen Type B in all other *Passovia* species studied so far. It differs from that of *Panamanthus* (potential Type C) by its widened colpi, pronounced triradial thickening in the polar area visible both in LM and SEM, and general higher similarity to the pollen Type C of *Dendropemon.*

*Peristethium leptostachyum* (Kunth.) Tiegh. (Figure 29; Table VI)

#### Description

Pollen, oblate, convex-triangular to straight-triangular in polar view, elliptic in equatorial view, equatorial apices broadly rounded; size small, polar axis 8.3–10.0 µm long in LM, equatorial diameter 15.0–17.5 µm in LM, 14.3–17.3 µm in SEM; syn(3)colpate to demisyn(3)colpate, colpi long; exine 0.8–1.1 µm thick, nexine thinner than sexine, triangular intercolpial nexine thickenings in polar area (LM), some grains with a minute protruding island in central polar area (SEM); tectate; sculpturing psilate in LM, rugulate to striate in mesocolpium and polar area in SEM; margo absent, colpus membrane nano-rugulate (SEM).

#### Remark

Pollen Type B. Feuer and Kuijt (1979) classified the pollen of *Struthanthus leptostachyus* (Kunth) G.Don, which recently was included in *Peristethium*, as ‘Type Ia1/b1’ in their study of the genus (cf. File S4). Features shared by some *Struthanthus* species and *Peristethium leptostachyum* are (partly) demicolpate apertures (also found in *S. hartwegii* [Benth.] Standl.), a rugulate sculpturing (shared with several other species), and a polar area with a protruding central part (several species). Compared with available micrographs of *Struthanthus* species, the here figured pollen of *P. leptostachyum* shows a unique character suite. Most notably, it is so far the only pollen from the *Cladocolea-Struthanthus* lineage with a partly striate sculpturing. Caires et al. (2014) moved another species from *Struthanthus* to *Peristethium*, and also figure the pollen in SEM (polar view). The pollen has a similar general appearance, but details of the sculpturing are not visible in the micrograph. There is no molecular data on the genus.

*Phthirusa clandestina* (Mart. ex Roem. et Schult.f.) Mart. (Figure 30; Table VII)

#### Description

Pollen, oblate, straight-triangular in polar view, broadly elliptic in equatorial view, equatorial apices broadly rounded to obcordate; size small, polar axis 15.0–16.7 µm long in LM, equatorial diameter 18.3–23.3 µm in LM, 15.3–22.2 µm in SEM; syn(3)colpate; exine 0.9–1.4 µm thick, nexine thinner than sexine, nexine hexagonally thickened in polar area, sexine thickened in area of mesocolpium (LM); tectate; sculpturing psilate in LM, micro-rugulate in area of mesocolpium in SEM; margo fairly well developed, margo micro-rugulate to psilate and perforate; colpus membrane nano-verrucate to nano-echinate (SEM).

#### Remark

Pollen Type B. The pollen shows a unique character suite and is most similar to that of *Passovia ovata* and *P. pedunculata*. It differs from these by being syncolpate with a margo that is sculptured in the same way as the mesocolpium (micro-rugulate in contrast to psilate in species of *Passovia*).

*Phthirusa hutchisonii* (Kuijt) Kuijt (Figure 31; Table VII)

#### Description

Pollen, spheroidal to slightly oblate, outline circular to lobate in polar view, circular to elliptic in equatorial view; size small to medium, polar axis 23.3–26.7 µm long in LM, 22.8–28.6 µm in SEM, equatorial diameter 26.7–31.7 µm in LM, 22.3–32.0 µm in SEM; zono(4)colpate, colpi short; exine 1.0–1.5 µm thick, nexine thinner than sexine (LM); tectate; sculpturing uniform, echinate in LM and SEM, echini 0.9–2.5 µm long, 0.5–1.2 µm wide at base; colpus membrane nano-echinate (SEM).

#### Remark

Pollen Type A. The only Loranthaceae with an equally atypical pollen is *Tupeia antarctica* from New Zealand (see earlier). Feuer and Kuijt (1985) note additional 3- and 5-colpate grains, not observed in our material.

*Psittacanthus calyculatus* (DC.) G.Don (Figure 32; Table V)

#### Description

Pollen, distinctly oblate, straight-triangular to concave-triangular in polar view, emarginate in equatorial view, equatorial apices truncated; size small to medium, polar axis 6.7–10.0 µm long in LM, equatorial diameter 23.3–31.7 µm in LM, 22.5–26.7 µm in SEM; syn(3)colpate; exine 1.0–1.3 µm thick, nexine thinner than sexine (LM); tectate; sculpturing psilate in LM, nano-echinate to micro-echinate in area of mesocolpium in SEM, echini 0.3–0.9 µm long, 0.2–0.6 µm wide at base; margo well developed, widening in equatorial region, margo psilate (SEM).

#### Remark

Pollen Type B. Feuer and Kuijt (1979) studied the pollen of 30 *Psittacanthus* species (including 12 that are now treated as synonyms) and established several types and subtypes, figuring exemplary specimens including *P. calyculatus* (representing ‘Type Ib’). Species with similar syncolpate pollen and nano- to micro-echinate sculpturing are *P. columbianus* A.C.Sm., *P. macranthus* Hook (species does not exist, Tropicos.org 2016; R. Vidal-Russell, personal communication, 2016), *P. schiedeanus* (Schtdl. et Cham.) G.Don, *P. sonorae* (S.Watson) Kuijt. (‘Type Ia’), and *P. clusiifolius* Eichler (‘Type Id’; Files S3, S4; Table V).

#### Note

Pollen surface features seen in the LM micrographs are not reflecting sculptural elements but cell contain contents and flower material, due to incomplete acetolisation. Fully processed grains had the tendency to rupture and lose their form, so we opted to interrupt the acetolising process for LM photography.

*Psittacanthus rhynchanthus* (Benth.) Kuijt (Figure 33; Table V)

#### Description

Pollen, distinctly oblate, concave-triangular to trilobate in polar view, emarginate in equatorial view, equatorial apices obcordate; size medium, polar axis 10.0–13.0 µm long in LM, equatorial diameter 25.0–33.3 µm in LM, 25.3–28.2 µm in SEM; zono(3)colpate, colpi of medium length; exine 1.1–1.5 µm thick, nexine thinner than sexine (LM); tectate; sculpturing psilate in LM, nano-/micro-baculate to nano-/micro-echinate in area of mesocolpium and polar area in SEM, bacula/echini 0.3–1.0 µm long, 0.2–0.4 µm wide at base; margo well developed, margo psilate (SEM).

#### Remark

Pollen Type B. Zonocolpate grains are rarer in species of *Psittacanthus*. Further species with zonocolpate grains are *P. cucullaris* (Lam.) G.Don and *P. dilatatus* A.C.Sm. (Table V; Feuer & Kuijt 1979; Files S3, S4) Pollen of these two species are generally similar to the pollen of *P. rhynchanthus*. However, Feuer and Kuijt (1979) included these three species in types (‘Type Ib’, ‘Type Ic’) with syncolpate pollen (see ‘Discussion’).

*Struthanthus* spp.

#### General description based on the species figured in Feuer and Kuijt (1985; see Files S4, S5)

Pollen, (distinctly) oblate, straight- to convex-triangular in polar view, elliptic, slightly emarginate or subrhombic in equatorial view, equatorial apices truncated, rounded or obcordate; size medium (cf. Feuer & Kuijt 1985, table 1); demi- or parasyn(3)colpate or syn(3)colpate, some with nexine thinning perpendicular to colpi and, others with triangular polar thickening; tectate; sculpturing psilate in LM, not clear in SEM for most species, some (*S. concinnus* Mart., *S. marginatus*, *S. uraguensis*) rugulate; parasyncolpate pollen with a protruding apocolpial field (SEM).

#### Remark

Pollen of *Struthanthus* is divers, including types very similar to that of *Cladocolea*, and types unique to the genus and similar to pollen of *Peristethium leptostachyum*. Noting the generic re-associations of species included in *Cladocolea*, *Peristethium* and *Struthanthus*, a final assessment regarding the pollen diversity of the genus will have to wait until comprehensive (regarding species coverage) molecular data becomes available.

*Tripodanthus acutifolius* (Ruiz et Pav.) Tiegh. (Figure 34)

#### Description

Pollen, distinctly oblate, concave-triangular in polar view, elliptic in equatorial view, equatorial apices truncated; size small, polar axis 6.7–8.3 µm long in LM, equatorial diameter 16.7–23.3 µm in LM, 14.3–20.4 µm in SEM; syn(3)colpate; exine 1.1–1.6 µm thick, nexine thinner than sexine, sexine thickened in area of mesocolpium, triangular intercolpial nexine thickenings in polar area (LM); tectate; sculpturing psilate in LM, micro-baculate to baculate in area of mesocolpium in SEM, bacula 0.6–1.4 µm long, 0.2–0.3 µm wide at base; margo well developed, widening in equatorial region, margo psilate (SEM).

#### Remark

Pollen Type B. Pollen of *Tripodanthus acutifolius* and *T. flagellaris* (Cham. et Schltdl.) Tiegh. (cf. Feuer & Kuijt 1980) is highly distinct within the Psittacanthinae lineage and Loranthaceae in general. Diagnostic features are the extremely oblate grains (can be more than three-times wide as high), the characteristic margo, and the long, densely packed bacula in the mesocolpium. Pollen of the third species, *T. belmirensis*, is figured in Roldán and Kuijt (2005), and seems to lack the diagnostic features (Table V; but see ‘Discussion’).

Lorantheae clade

The Lorantheae have four main clades, three of which (Amyeminae, Ileostylinae, Loranthinae) correspond to a single subtribe (Table I). The Lorantheae core clade (Clade J in Vidal-Russell & Nickrent 2008b) comprises the remaining subtribes (Dendrophthoinae, Emelianthinae, Scurrulinae, Tapinanthinae; Figures 2, 3, 7, 8). All Lorantheae pollen is of Type B. One of the most distinct pollen within the clade is the small, compact pollen of *Loranthus*, one of the two genera in the Loranthinae (the pollen of the second genus, *Cecarria*, is unknown). Pollen of *Loranthus*, like that of *Plicosepalus* (Tapinanthinae), can be heteropolar and distinctly bean-shaped in equatorial view. Heteropolar pollen grains are typically syncolpate on the proximal face and zonocolpate on the distal face. While most isopolar pollen grains are syncolpate, the other studied Tapinanthinae, which form a distinct subclade within the Lorantheae core clade, are typically zonocolpate (Figure 8). The only Emelianthinae with zonocolpate pollen is *Moquiniella*. Pollen outline of isopolar grains of the Lorantheae can be elliptic, emarginate, or rarely (*Tapinanthus*) subrhombic in equatorial view; in polar view the outlines range from straight-triangular (e.g. *Loranthus*) to trilobate. Several species/genera show the entire range from (slightly) convex-triangular to distinctly trilobate pollen (exemplified in Figures 7, 8). The equatorial apices are mostly obcordate but can also be T-shaped in several genera of the core Lorantheae (*Agelanthus*, *Phragmanthera*, *Tapinanthus*). A distinct margo, often psilate but in some taxa (partly) granulate, nano- or micro-verrucate, can be seen in most pollen except for *Loranthus* (Loranthinae), *Helixanthera*, and one of the *Englerina* species (core Lorantheae). In general, the structural elements are very small (≤ 0.5 µm); typically nano-verrucate to granulate, the mesocolpium of some Tapinanthinae (*Agelanthus, Englerina, Oncocalyx* and *Tapinanthus*) and *Muellerina* (Ileostylinae) shows nanoechini and nanobacula. Nanoverrucae are composed of conglomerate granula. A variable but common feature that can be seen under the LM and SEM (to some degree) is the thickening of (parts of) the exine (nexine and/or sexine) in the polar area, and an occasional thickening of the sexine in the mesocolpium at the equator. In overall appearance, the Lorantheae pollen cannot be confused with pollen of the Elytrantheae, Psittacantheae or the root-parasitic species. An exception may be the pollen of *Muellerina*, which is similar to that of *Gaiadendron* (Figure 11). Problematic may also be distinguishing between pollen of *Aetanthus* (Figures 22–24) and one type of *Psittacanthus* (Figure 33) with the echinate/baculate pollen of several core Lorantheae.

Amyeminae

#### Remark

So far, palynological data are only available for a single member of this Indomalayan-Australasian subtribe, which includes eight genera, seven for which molecular data are available.

**Figure 35.**
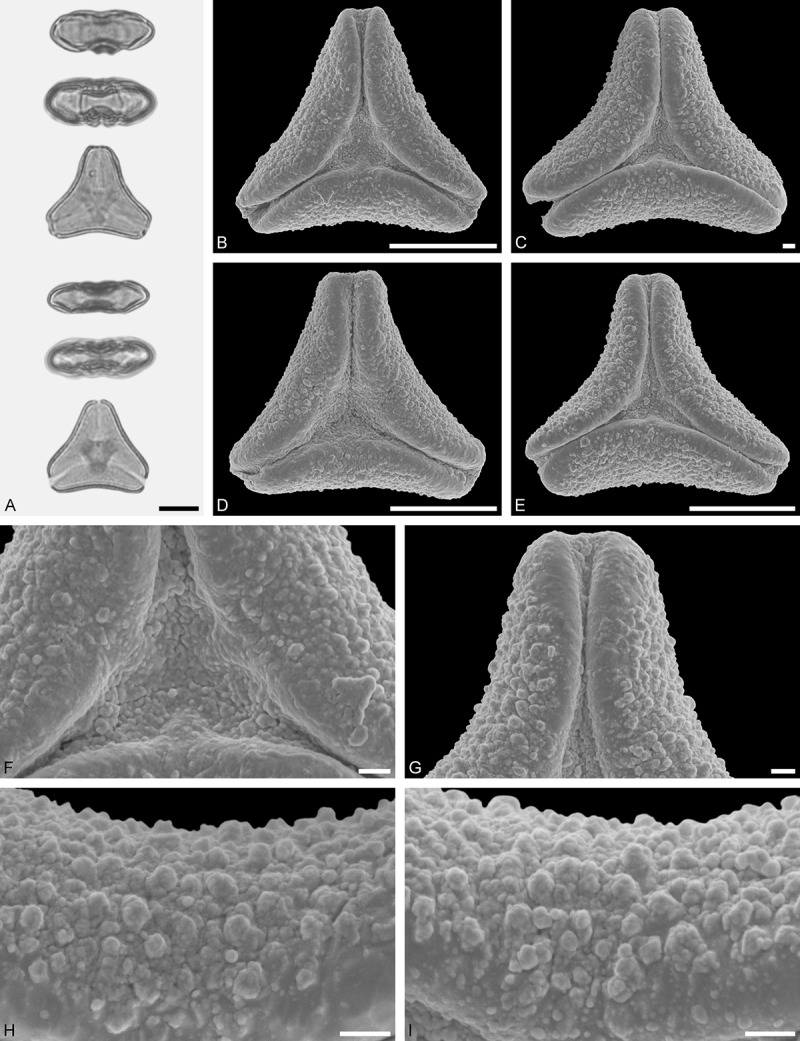
LM (A) and SEM (B–I) micrographs of *Amyema gibberula* (from South Australia N. N. Donner, WU s.n.). **A.** Two pollen grains in equatorial and polar view. **B–E.** Pollen grains in polar view. **F.** Close-up of central polar area. **G.** Close-up of apex. **H, I.** Close-ups of mesocolpium. Scale bars – 10 µm (A, B, D, E), 1 µm (C, F–I).

*Amyema gibberula* Danser (Figure 35)

#### Description

Pollen, distinctly oblate, concave-triangular in polar view, emarginate in equatorial view, equatorial apices obcordate; size small, polar axis 7.5–8.3 µm long in LM, equatorial diameter 20.8–24.2 µm in LM, 15.6–20.9 µm in SEM; syn(3)colpate, colpi widening towards apices and central polar area; exine 1.0–1.3 µm thick, nexine thinner than sexine, nexine hexagonally thickened in polar area (LM), sexine partly reduced in polar area, colpi widening to a small field (SEM); tectate; sculpturing psilate in LM, micro-verrucate to nano-verrucate and granulate in area of mesocolpium in SEM, verrucae 0.3–0.9 µm in diameter, verrucae composed of conglomerate granula; margo well developed, margo psilate or partly granulate to nano-/micro-verrucate, margo with small triangular protrusions in polar area (SEM); colpus membrane nano-verrucate to nano-rugulate and granulate (SEM).

#### Remark

Pollen Type B. Pollen of *Amyema* shows features rarely or not found in any other Lorantheae, but shared with other Loranthaceae lineages. The colpi are widening towards the pole, where the sexine is partly reduced forming a shallow depression (as seen in the Ligarinae), reminiscent but not to be confused with an apocolpial field. The margo may show small triangular protrusions into the central polar area, analogue to what can be found in the Elytrantheae.

Dendrophthoinae

#### Remark

This tropical African-Indomalayan subtribe, forming a grade in molecular phylograms, includes four genera, of which three are studied genetically and palynologically. No data are available for the western Malaysian *Trithecanthera*. Pollen of the three studied genera is similar, with pollen grains of *Helixanthera* being most distinct (paralleling the molecular evidence, Figure 7).

**Figure 36.**
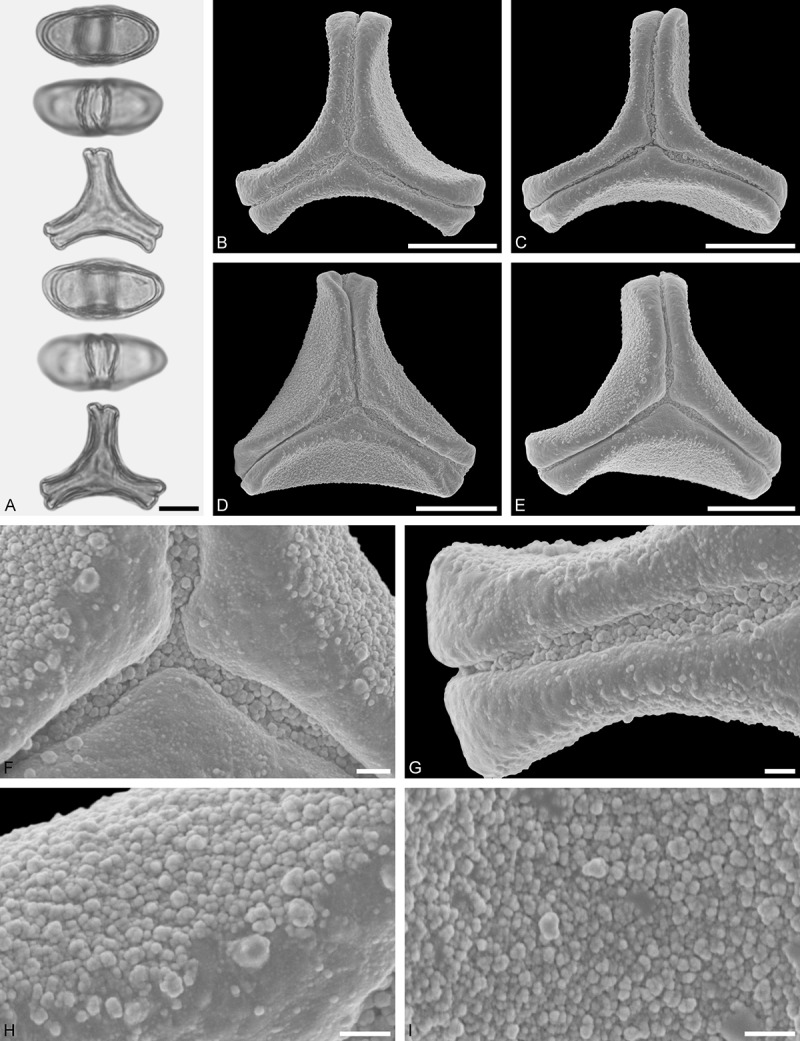
LM (A) and SEM (B–I) micrographs of *Dendrophthoe pentandra* (WU 0039138). **A.** Two pollen grains in equatorial and polar view. **B–E.** Pollen grains in polar view. **F.** Close-up of central polar area. **G.** Close-up of apex. **H, I.** Close-ups of mesocolpium. Scale bars – 10 µm (A–E), 1 µm (F–I).

*Dendrophthoe pentandra* (L.) Miq. (Figure 36)

#### Description

Pollen, oblate, concave-triangular to trilobate in polar view, elliptic in equatorial view, equatorial apices obcordate; size small, polar axis 13.3–15.0 µm long in LM, equatorial diameter 21.7–25.8 µm in LM, 20.5–26.7 µm in SEM; syn(3)colpate; exine 1.1–1.3 µm thick, nexine thinner than sexine (LM), sexine partly reduced in polar area, colpi widening to a small field (SEM); tectate; sculpturing psilate in LM, nano-verrucate and granulate in area of mesocolpium in SEM, verrucae 0.2–0.6 µm in diameter, verrucae composed of conglomerate granula; margo well developed, margo psilate or partly granulate to nano-verrucate, margo with triangular protrusions in polar area (SEM); colpus membrane nano-verrucate, granulate (SEM). – Pollen Type B.

**Figure 37.**
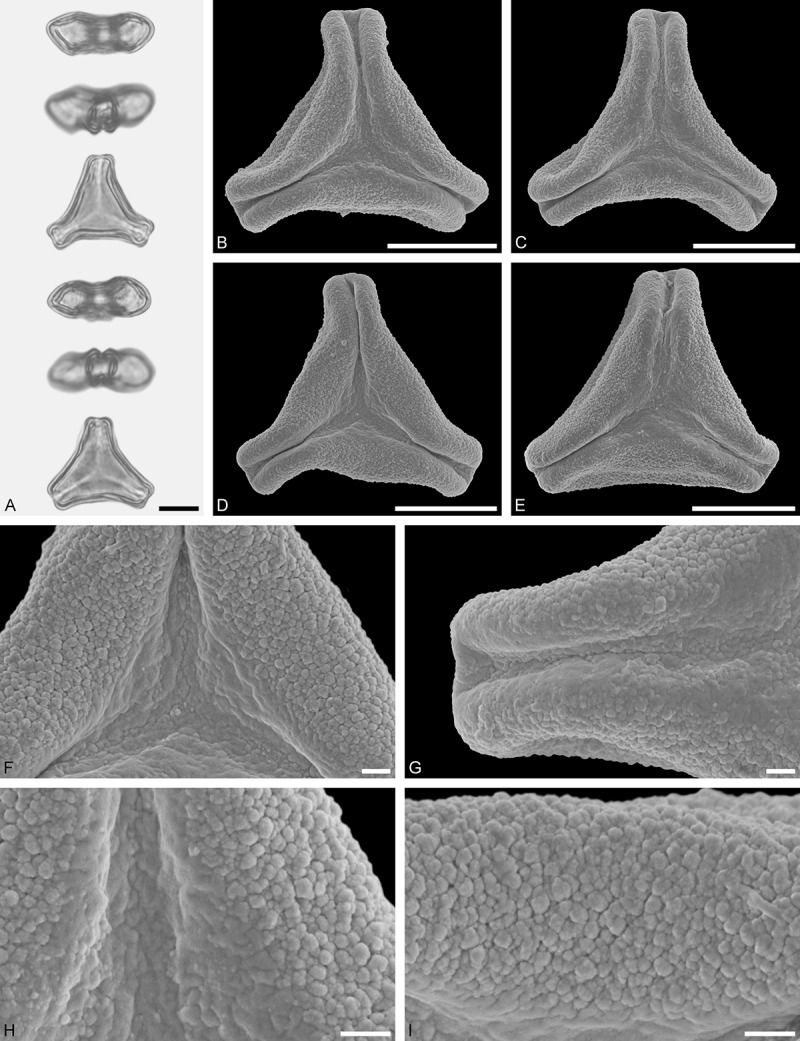
LM (A) and SEM (B–I) micrographs of *Helixanthera kirkii* (WU: from Zambezi River, S.E. Africa, coll. Meryhart, s.n.). **A.** Two pollen grains in equatorial and polar view. **B–E.** Pollen grains in polar view. **F.** Close-up of central polar area. **G.** Close-up of apex. **H, I.** Close-ups of mesocolpium. Scale bars – 10 µm (A–E), 1 µm (F–I).

*Helixanthera kirkii* (Oliv.) Danser (Figure 37)

#### Description

Pollen, distinctly oblate, concave-triangular in polar view, emarginate in equatorial view, equatorial apices obcordate; size small, polar axis 7.5–8.3 µm long in LM, equatorial diameter 20.0–24.2 µm in LM, 18.3–21.4 µm in SEM; syn(3)colpate; exine 1.0–1.3 µm thick, nexine thinner than sexine, triangular intercolpial nexine thickenings in polar area (LM), sexine partly reduced in polar area, colpi widening to a small field (SEM); tectate; sculpturing psilate in LM, nano-verrucate in area of mesocolpium in SEM, verrucae 0.2–0.6 µm in diameter, verrucae composed of conglomerate granula; margo indistinct, margo nano-verrucate, granulate or partly psilate, margo with triangular protrusions in polar area (SEM); colpus membrane nano-rugulate to nano-verrucate and granulate (SEM).

#### Remark

Pollen Type B. As in *Amyema*, the colpi widen towards the pole, where the sexine is partly reduced. This feature, which gives the pollen an emarginated outline in equatorial view, distinguishes it from that of the other two Dendrophthoinae. Another distinguishing feature is the indistinct margo of *Helixanthera kirkii.*

**Figure 38.**
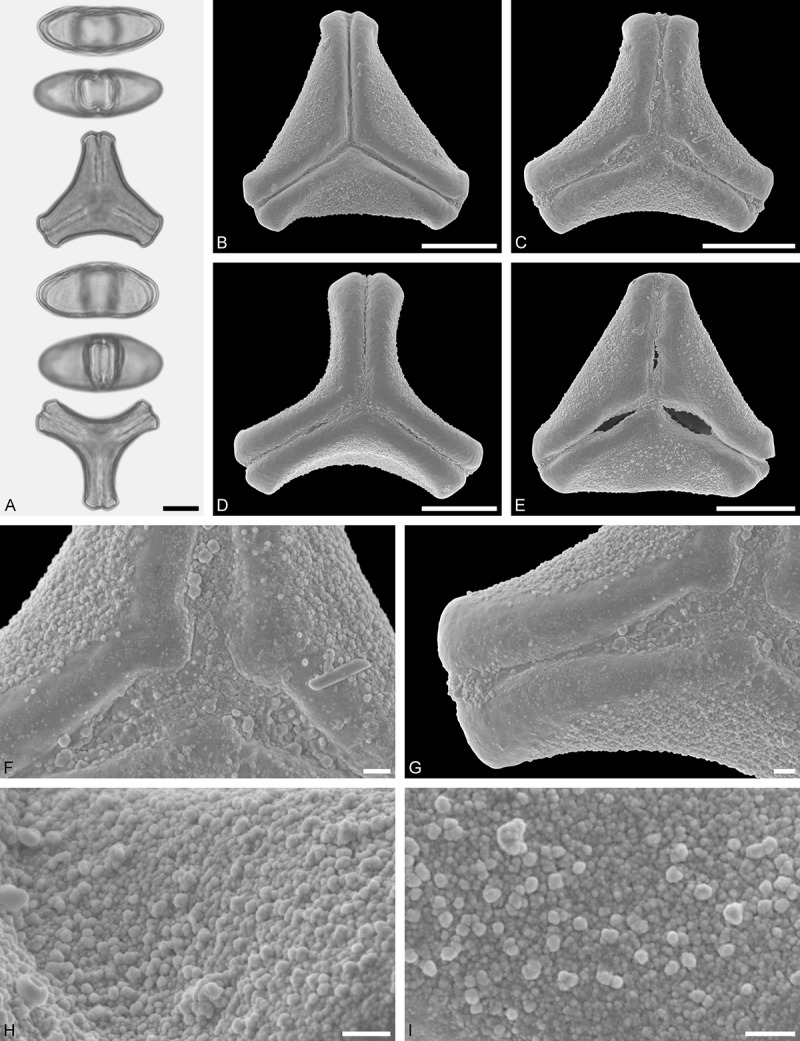
LM (A) and SEM (B–I) micrographs of *Tolypanthus maclurei* (WU 039104). **A.** Two pollen grains in equatorial and polar view. **B–E.** Pollen grains in polar view. **F.** Close-up of central polar area. **G.** Close-up of apex. **H, I.** Close-ups of mesocolpium. Scale bars – 10 µm (A–E), 1 µm (F–I).

*Tolypanthus maclurei* (Merr.) Danser (Figure 38)

#### Description

Pollen, oblate, trilobate to straight-triangular in polar view, elliptic in equatorial view, equatorial apices obcordate; size medium, polar axis 8.3–15.8 µm long in LM, equatorial diameter 25.0–30.0 µm in LM, 21.6–29.7 µm in SEM; syn(3)colpate; exine 0.8–1.3 µm thick, nexine thinner than sexine, triangular intercolpial nexine thickenings in polar area (LM); tectate; sculpturing psilate in LM, nano-verrucate and granulate in area of mesocolpium in SEM, verrucae 0.2–0.4 (–0.8) µm in diameter, verrucae composed of conglomerate granula; margo well developed, margo psilate or partly granulate, margo with triangular protrusions in polar area (SEM); colpus membrane nano-verrucate and granulate (SEM).

#### Remark

Pollen Type B. The pollen is nearly identical to that of *Dendrophthoe pentandra.*

Emelianthinae

#### Remark

The Emelianthinae form a distinct subclade within the Lorantheae. Palynological data are available for four out of the seven genera (Table IX). Except for *Moquiniella*, which is resolved as the first diverging lineage of the Emelianthinae (Figure 8), pollen is syncolpate. Pollen sculpturing is similar in all five species of the four studied genera, another shared feature is the thickening of the polar nexine (seen in LM).

**Table VIII.** Tabulation of differentiating pollen features in Lorantheae, part 1 (Figure 7).

Subtribe (clade)	Genus	Polarity, aperture	Size, shape, outline in e.v. and p.v.^a^	Margo	Further exine features (LM, SEM)	Sculpturing
Loranthinae (Clade G)	*Loranthus*	**Can be heteropolar**, syn(3)colpate or **zono(3)colpate**	Small, oblate, elliptic or **bean-shaped**, straight-triangular	Indistinct	LM: sexine thickened in MC, **polar (triradial) nexine thickening** SEM: polar area can be thickened	CM: nano-verrucate/-rugulate to granulate MC: micro-verrucate M: smoother than MC
Ileostylinae (Clade H)	*Muellerina*	Isopolar, syn(3)colpate	Small, (distinctly) oblate, elliptic to slightly emarginated, concave-triangular	Distinct	LM: nexine hexagonally thickened in polar area	CM: nano-verrucate/-echinate MC: nano-echinate/-baculate **M: striate to rugulate**
Amyeminae (Clade I)	*Amyema*	Isopolar, syn(3)colpate	Small, distinctly oblate, **emarginate**, concave-triangular	Distinct, **forming small triangular protrusions at pole**	LM: nexine hexagonally thickened in polar area **SEM: sexine partly reduced in polar area**	CM: nano-verrucate to -rugulate or granulate MC: nano-/micro-verrucate or granulate M: micro-verrucate, granulate or psilate
Dendroph-thinae (Clade J)	*Dendrophthoe*	Isopolar, syn(3)colpate	Small, oblate, elliptic, concave-triangular to trilobate	Distinct		CM: ? MC: nano-verrucate, granulate M: mostly psilate
	*Helixanthera*	Isopolar, syn(3)colpate	Small, distinctly oblate, **emarginate**, concave-triangular	Indistinct	**LM: nexine with triangular intercolpial thickenings** **SEM: sexine partly reduced in polar area**	CM: nano-rugulate to -verrucate, granulate MC: nano-verrucate M: nano-verrucate, granulate, partly psilate
	*Tolypanthus*	Isopolar, syn(3)colpate	Medium, oblate, elliptic, straight-triangular to trilobate	Distinct	**LM: nexine with triangular intercolpial thickenings**	CM, MC: nano-verrucate, granulate M: psilate, partly granulate
Scurullinae	*Scurrula, Taxillus*	Isopolar, syn(3)colpate	Small (to medium), (distinctly) oblate, elliptic, concave-triangular to trilobate	Distinct, most pronounced at apices, **triangular polar protrusions can be present**	LM: nexine in polar area (hexagonally) thickened	CM, MC: nano-verrucate to granulate M: Mostly psilate

Note: Abbreviations: e.v., equatorial view; p.v., polar view; LM, light microscopy; SEM, scanning electron microscopy; CM, colpane membrane; M, margo; MC, mesocolpium; ^a^ Size: small, <25 µm; medium, 25–50 µm; shape: oblate, less than two-times wider than high; distinctly oblate, more than two-times wider than high.

**Table IX.** Tabulation of differentiating pollen features in Lorantheae, part 2: Emelianthinae (subclade of Clade J, Figure 8).

Subtribe, genus	Polarity, aperture	Size, shape, outline in e.v. and p.v.^a^	Equatorial apices	Margo	Further exine features (LM, SEM)	Sculpturing
*Erianthemum*	Isopolar, syn(3)colpate	Small to medium, distinctly oblate, slightly **emarginate**, concave-triangular	Obcordate	Distinct, **broadening at equator, sometime with small triangular protrusions at pole**	LM: **nexine with triangular intercolpial thickenings**	CM: nano-verrucate to granulate MC: nano-verrucate M: psilate, partly nano-verrucate to granulate
*Globimetula*	Isopolar, syn(3)colpate	Medium, distinctly oblate, **emarginate**, concave-triangular to trilobate	Obcordate	Distinct, **broadening at equator**	LM: nexine hexagonally thickened in polar area; **sexine markedly thickened in MC**	CM, MC: nano-verrucate to granulate M: psilate to micro-verrucate, granulate
*Moquiniella*	Isopolar, **zono(3)colpate**	Medium, distinctly oblate, slightly **emarginate**, concave-triangular	Obcordate	Distinct	LM: nexine thickened in polar area	CM: ? MC: nano-verrucate to granulate M: psilate
*Phragmanthera*	Isopolar, syn(3)colpate	Medium, oblate, elliptic, concave-triangular to trilobate	**T-shaped**	Distinct, **covering the entire apices, can show intercolpial triangular build-ups at the pole**	LM: **nexine with triangular intercolpial thickenings**	CM, MC: nano-verrucate to granulate M: psilate, partly granulate

Note: Abbreviations: e.v., equatorial view; p.v., polar view; p.f., proximal face; d.f., distal face; LM, light microscopy; SEM, scanning electron microscopy; CM, colpus membrane; MC, mesocolpium; M, margo; ^a^ Size: small, <25 µm; medium, 25–50 µm; shape: oblate, less than two-times wider than high; distinctly oblate, more than two-times wider than high.

**Figure 39.**
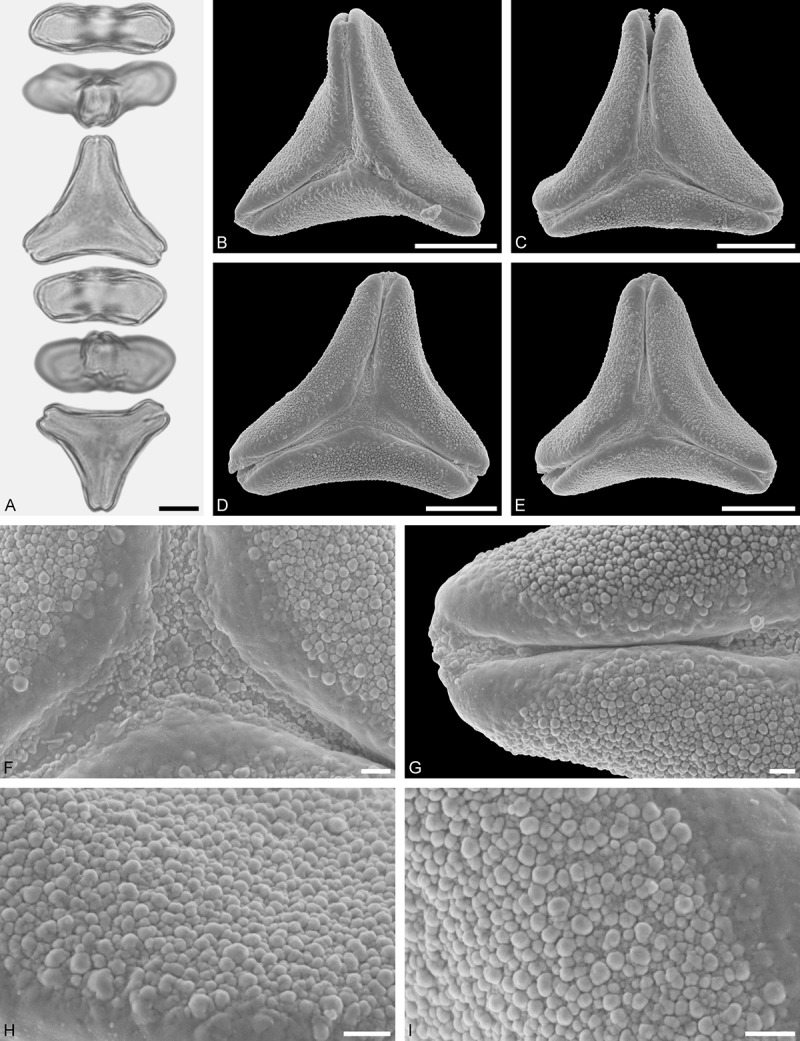
LM (A) and SEM (B–I) micrographs of *Erianthemum dregei* (WU 039040). **A.** Two pollen grains in equatorial and polar view. **B–E.** Pollen grains in polar view. **F.** Close-up of central polar area. **G.** Close-up of apex. **H, I.** Close-ups of mesocolpium. Scale bars – 10 µm (A–E), 1 µm (F–I).

*Erianthemum dregei* Tiegh. (Figure 39)

#### Description

Pollen, distinctly oblate, concave-triangular in polar view, slightly emarginate in equatorial view, equatorial apices obcordate; size small to medium, polar axis 6.7–15.0 µm long in LM, equatorial diameter 23.3–31.7 µm in LM, 24.2–30.6 µm in SEM; syn(3)colpate; exine 1.0–1.5 µm thick, nexine thinner than sexine, triangular intercolpial nexine thickenings in polar area (LM); tectate; sculpturing psilate in LM, nano-verrucate in area of mesocolpium in SEM, verrucae 0.2–0.6 µm in diameter; margo well developed, margo more distinct in equatorial regions, margo psilate or partly nano-verrucate to granulate, margo sometimes with small triangular protrusions in polar area (SEM); colpus membrane nano-verrucate and granulate (SEM). – Pollen Type B.

**Figure 40.**
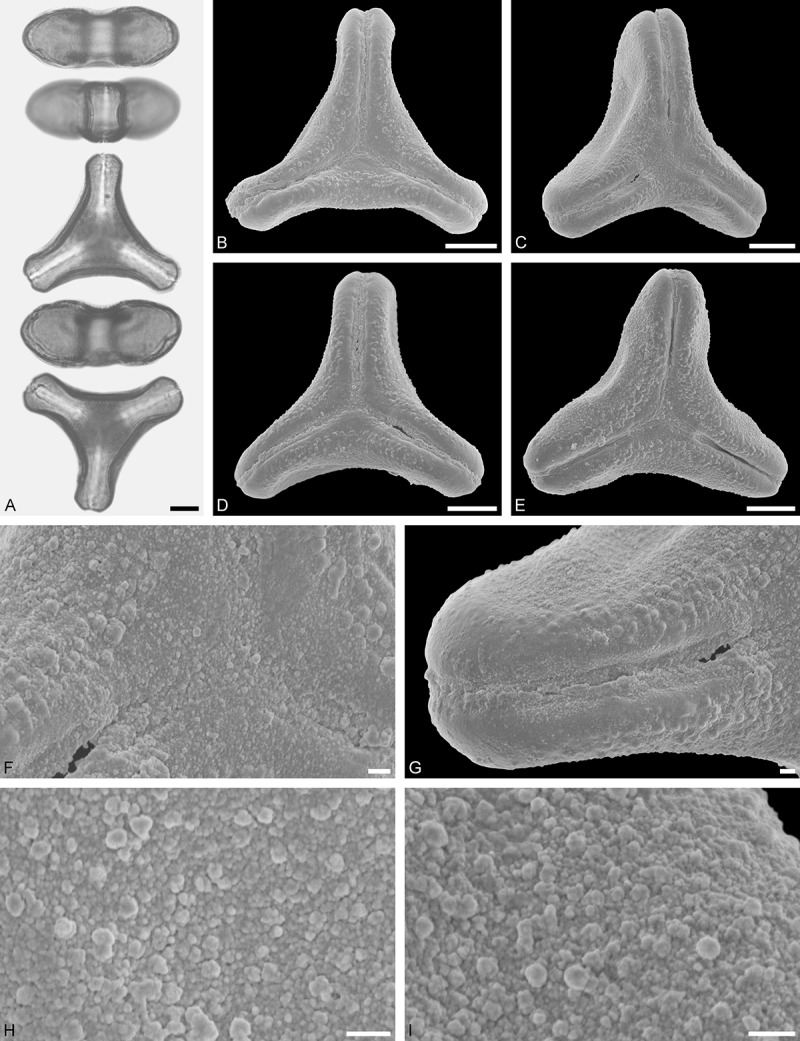
LM (A) and SEM (B–I) micrographs of *Globimetula dinklagei* (WU: from Cameroun, collector unknown, det. S. Balle, s.n.). **A.** Two pollen grains in equatorial and polar view. **B–E.** Pollen grains in polar view. **F.** Close-up of central polar area. **G.** Close-up of apex. **H, I.** Close-ups of mesocolpium. Scale bars – 10 µm (A–E), 1 µm (F–I).

*Globimetula dinklagei* (Engl.) Danser (Figure 40)

#### Description

Pollen, distinctly oblate, trilobate to concave-triangular in polar view, emarginate in equatorial view, equatorial apices obcordate; size medium, polar axis 15.0–18.3 µm long in LM, equatorial diameter 41.7–48.3 µm in LM, 39.1–45.3 µm in SEM; syn(3)colpate; exine 1.3–2.4 µm thick, nexine thinner than sexine, nexine hexagonally thickened in polar area, sexine markedly thickened in area of mesocolpium (LM); tectate; sculpturing psilate in LM, nano-verrucate to granulate in area of mesocolpium in SEM, verrucae 0.2–0.5 (−0.7) µm in diameter, verrucae composed of conglomerate granula; margo well developed, margo micro-verrucate and partly granulate in polar area, margo psilate to partly granulate in equatorial regions (SEM); colpus membrane nano-verrucate and granulate (SEM).

#### Remark

Pollen Type B. Pollen of *Globimetula* is the largest pollen in Lorantheae and one of the largest pollen in the entire family: only pollen of *Loxanthera* (Elytrantheae) and some *Psittacanthus* (Psittacantheae: Psittacanthinae) are larger. A unique feature within the Lorantheae is the markedly thickened mesocolpial sexine.

**Figure 41.**
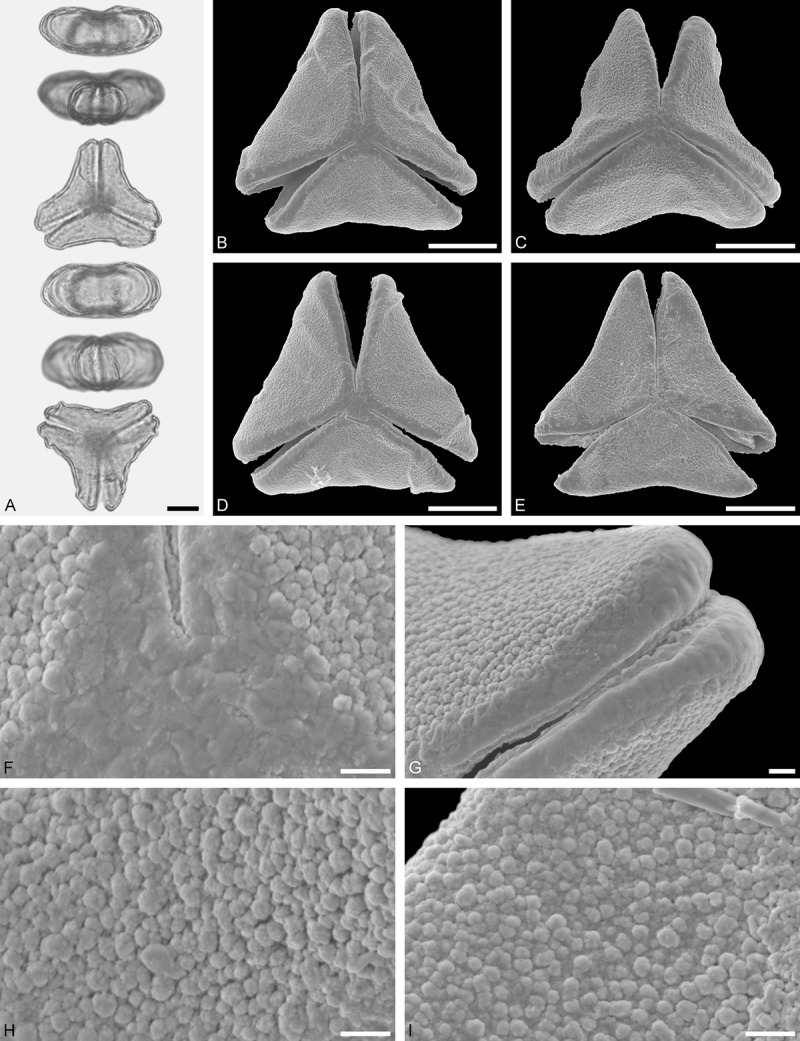
LM (A) and SEM (B–I) micrographs of *Moquiniella rubra* (WU: origin and collector unknown, det. S. Balle, s.n.). **A.** Two pollen grains in equatorial and polar view. **B–E.** Pollen grains in polar view. **F.** Close-up of central polar area. **G.** Close-up of apex. **H, I.** Close-ups of mesocolpium. Scale bars – 10 µm (A–E), 1 µm (F–I).

*Moquiniella rubra* (A.Spreng.) Balle (Figure 41)

#### Description

Pollen, distinctly oblate, concave-triangular in polar view, slightly emarginate in equatorial view, equatorial apices obcordate; size medium, polar axis 15.0–17.5 µm long in LM, equatorial diameter 30.0–35.0 µm in LM, 27.1–32.9 µm in SEM; zono(3)colpate, colpi very long; exine 1.1–1.5 µm thick, nexine thinner than sexine, nexine thickened in central polar area (LM); tectate; sculpturing psilate in LM, nano-verrucate to granulate in area of mesocolpium in SEM, verrucae 0.2–0.5 µm in diameter, verrucae composed of conglomerate granula; margo well developed, margo psilate (SEM).

#### Remark

Pollen Type B. This pollen differs from all other Emelianthinae pollen by being zonocolpate, a feature shared with other core Lorantheae.

**Figure 42.**
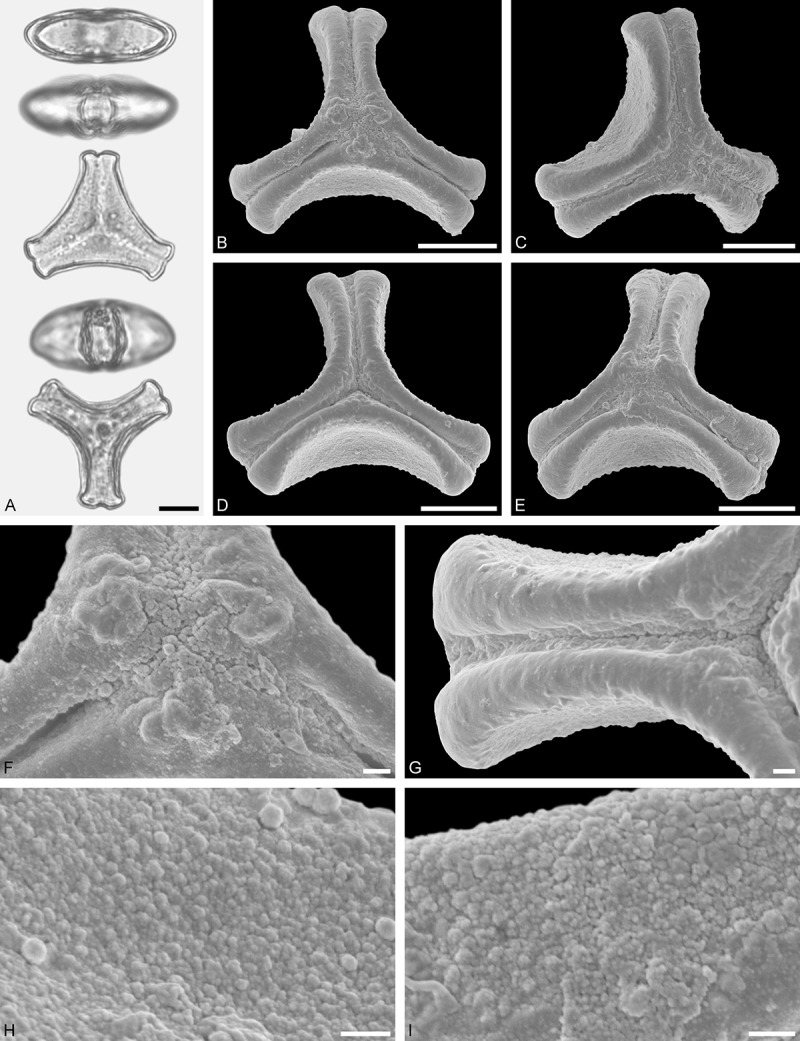
LM (A) and SEM (B–I) micrographs of *Phragmanthera capitata* (WU: from Cameroun, collector unknown, det. S. Balle, s.n.). **A.** Two pollen grains in equatorial and polar view. **B–E.** Pollen grains in polar view. **F.** Close-up of central polar area. **G.** Close-up of apex. **H, I.** Close-ups of mesocolpium. Scale bars – 10 µm (A–E), 1 µm (F–I).

*Phragmanthera capitata* (Spreng.) Balle (Figure 42)

#### Description

Pollen, oblate, trilobate to concave-triangular in polar view, elliptic in equatorial view, equatorial apices T-shaped; size medium, polar axis 13.3–18.3 µm long in LM, equatorial diameter 25.0–30.0 µm in LM, 23.6–26.4 µm in SEM; syn(3)colpate; exine 1.1–1.6 µm thick, nexine thinner than sexine, triangular intercolpial nexine thickenings in polar area (LM); tectate; sculpturing psilate in LM, nano-verrucate to granulate in area of mesocolpium in SEM, verrucae 0.2–0.5 µm in diameter, verrucae composed of conglomerate granula; margo well developed, margo psilate or partly granulate, margo often with three large intercolpial sexine thickenings (irregular build-ups) in central polar area (SEM); colpus membrane nano-verrucate and granulate (SEM).

#### Remark

Pollen Type B. The irregular, intercolpial build-ups in the central polar area seen in SEM (Figure 42F) are unique within the family.

#### Note

Pollen surface features seen in the LM micrographs are not reflecting sculptural elements but cell contents and flower material, due to incomplete acetolisation. Fully processed grains had the tendency to rupture and lose their form, so we opted to interrupt the acetolising process for LM photography.

**Figure 43.**
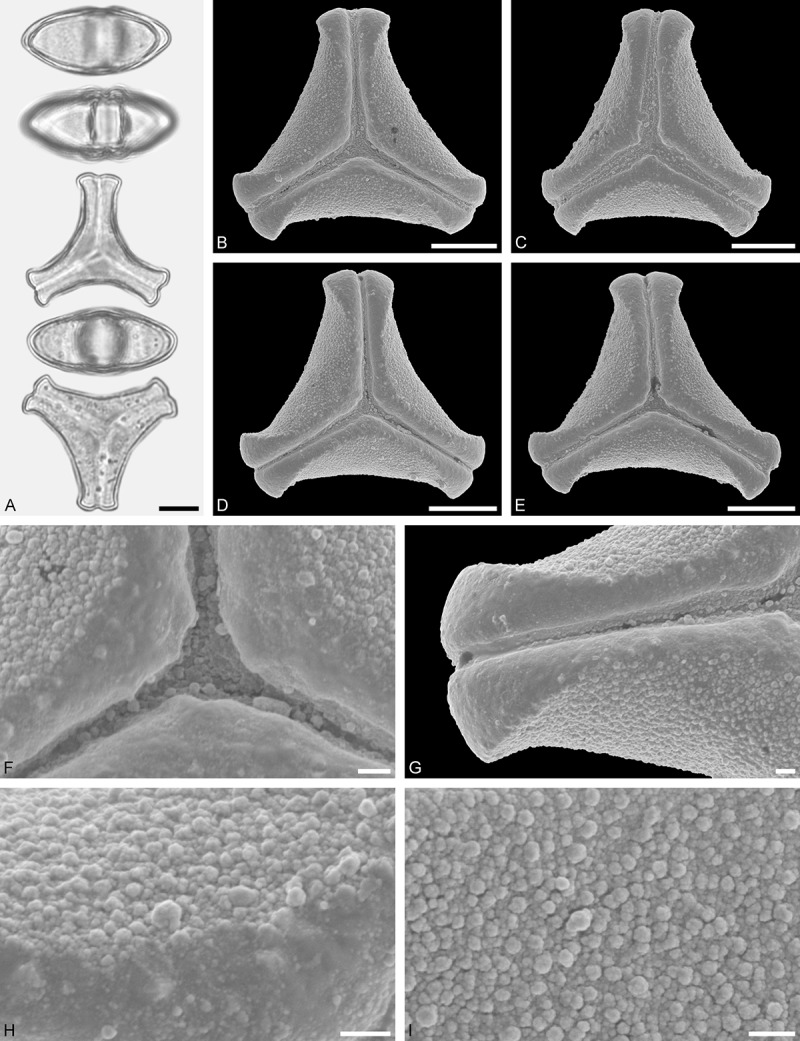
LM (A) and SEM (B–I) micrographs of *Phragmanthera rufescens* (WU: from Cameroun, collector unknown, det. S. Balle, s.n.). **A.** Two pollen grains in equatorial and polar view. **B–E.** Pollen grains in polar view. **F.** Close-up of central polar area. **G.** Close-up of apex. **H, I.** Close-ups of mesocolpium. Scale bars – 10 µm (A–E), 1 µm (F–I).

*Phragmanthera rufescens* (DC.) Balle (Figure 43)

#### Description

Pollen, oblate, concave-triangular in polar view, elliptic in equatorial view, equatorial apices T-shaped; size medium, polar axis 15.0–18.3 µm long in LM, equatorial diameter 26.7–31.7 µm in LM, 27.9–33.3 µm in SEM; syn(3)colpate; exine 1.1–1.4 µm thick, nexine thinner than sexine, triangular intercolpial nexine thickenings in polar area (LM); tectate; sculpturing psilate in LM, nano-verrucate to granulate in area of mesocolpium in SEM, verrucae 0.1–0.6 µm in diameter, verrucae composed of conglomerate granula; margo well developed, margo psilate or partly granulate (SEM); colpus membrane nano-verrucate and granulate (SEM).

#### Remark

Pollen Type B. The pollen of this species is very similar to that of *Phragmanthera capitata* except for the intercolpial, polar sexine build-ups in that species.

#### Note

Pollen surface features seen in the LM micrographs are not reflecting sculptural elements but cell contents and flower material, due to incomplete acetolisation. Fully processed grains had the tendency to rupture and lose their form, so we opted to interrupt the acetolising process for LM photography.

Ileostylinae

#### Remark

The Ileostylinae are a genetically distinct, but very small clade of Lorantheae with one monotypic genus in New Zealand, *Ileostylus* (not studied palynologically in detail; LM micrographs can be found in the Australasian Pollen and Spore Atlas at http://apsa.anu.edu.au), and the eastern Australian genus *Muellerina*, studied here for the first time using SEM.

**Figure 44.**
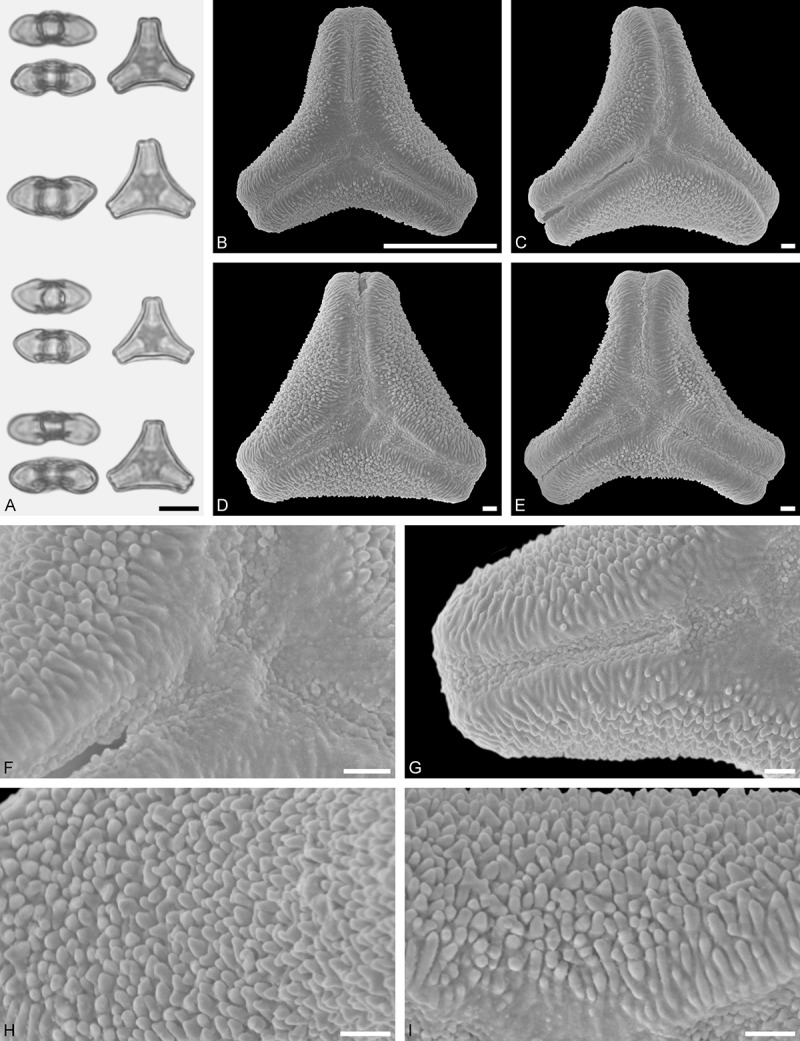
LM (A) and SEM (B–I) micrographs of *Muellerina eucalyptoides* (MEL 2064534). **A.** Four pollen grains in equatorial and polar view. **B–E.** Pollen grains in polar view. **F.** Close-up of central polar area. **G.** Close-up of apex. **H, I.** Close-ups of mesocolpium. Scale bars – 10 µm (A, B), 1 µm (C–I).

*Muellerina eucalyptoides* (DC.) Barlow (Figure 44)

#### Description

Pollen, distinctly oblate, concave-triangular in polar view, elliptic to slightly emarginate in equatorial view, equatorial apices obcordate to broadly rounded; size small, polar axis 6.7–9.2 µm long in LM, equatorial diameter 15.8–18.3 µm in LM, 13.7–17.9 µm in SEM; syn(3)colpate; exine 1.1–1.5 µm thick, nexine thinner than sexine, nexine hexagonally thickened in polar area (LM); tectate; sculpturing psilate in LM, nano-echinate to nano-baculate in area of mesocolpium in SEM, echini/bacula 0.3–0.6 µm long, 0.2–0.3 µm wide at base; margo well developed, margo striate to rugulate, striae/rugulae perpendicular to colpi; colpus membrane nano-verrucate to nano-echinate (SEM).

#### Remark

Pollen Type B. The pollen grains of *Muellerina eucalyptoides* from Australia are distinct from all other Lorantheae and remarkably similar to pollen grains of *Gaiadendron punctatum* (Gaiadendreae; Figure 11) and ‘*Struthanthus*’ *mapirensis* Rusby (a likely *Gaiadendron*; see ‘Discussion’) from South America. So far, only a single grain of *Muellerina* has been published in LM (Macphail et al. 2012). Another image can be found in the Australasian pollen and spore atlas (http://apsa.anu.edu.au). Both images match with our pollen in size and overall appearance.

Loranthinae

#### Remark

The Loranthinae include the Eurasian *Loranthus* with *c*. ten species, the only Loranthaceae extending into Europe, and the monotypic genus *Cecarria* (Philippines, New Guinea, New Britain, Solomon Islands), for which no palynological data are available.

**Figure 45.**
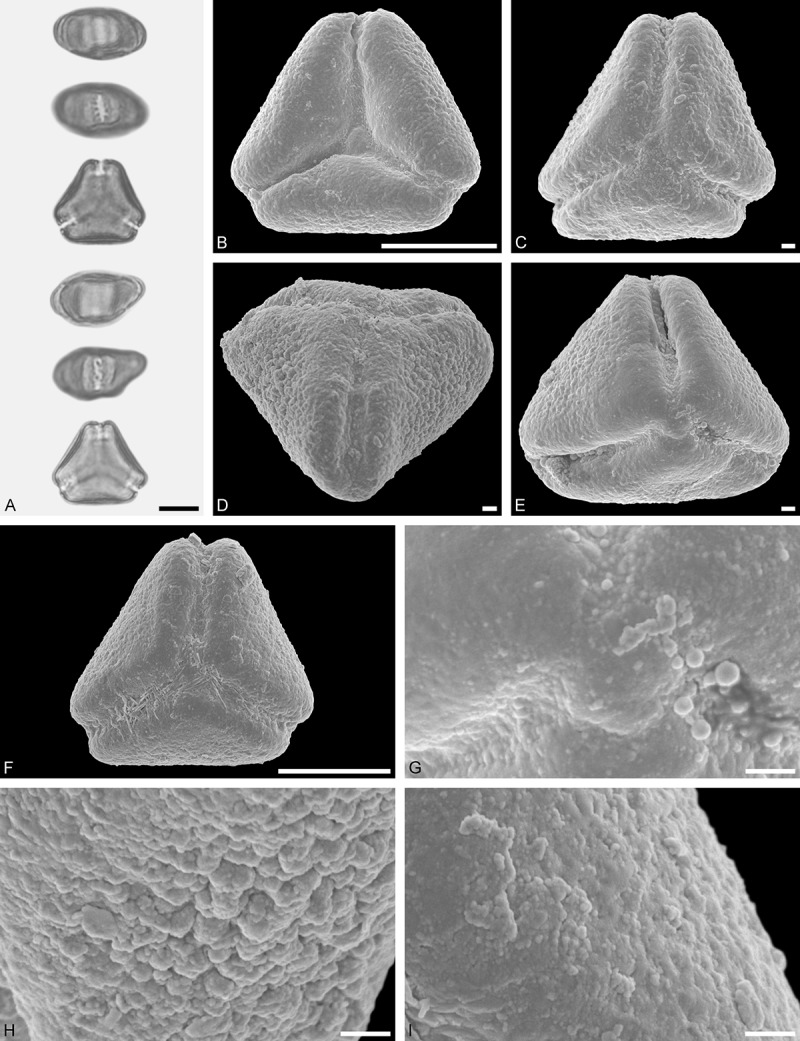
LM (A) and SEM (B–I) micrographs of *Loranthus delavayi* (WU 039101). **A.** Two pollen grains in equatorial and polar view. **B–C, E, F.** Pollen grains in polar view. **D.** Pollen grain in oblique equatorial view. **G.** Close-up of central polar area. **H, I.** Close-ups of mesocolpium. Scale bars – 10 µm (A, B, F), 1 µm (C–E, G–I).

*Loranthus delavayi* Tiegh. (Figure 45)

#### Description

Pollen, oblate, straight-triangular in polar view, elliptic in equatorial view, equatorial apices obcordate; size small, polar axis 11.7–15.0 µm long in LM, equatorial diameter 20.5–25.0 µm in LM, 16.8–20.0 µm in SEM; syn(3)colpate (isopolar grains) or syn-/zono-(3)colpate, colpi very long (heteropolar grains); exine 1.1–1.7 µm thick, nexine thinner than sexine, triradial thickening of nexine in polar area, sexine markedly thickened in area of mesocolpium (LM); tectate; sculpturing psilate in LM, micro-verrucate and granulate, perforate, in area of mesocolpium in SEM, verrucae 0.4–1.1 µm in diameter; margo indistinct, margo micro-verrucate to psilate and partly granulate (SEM). – Pollen Type B.

**Figure 46.**
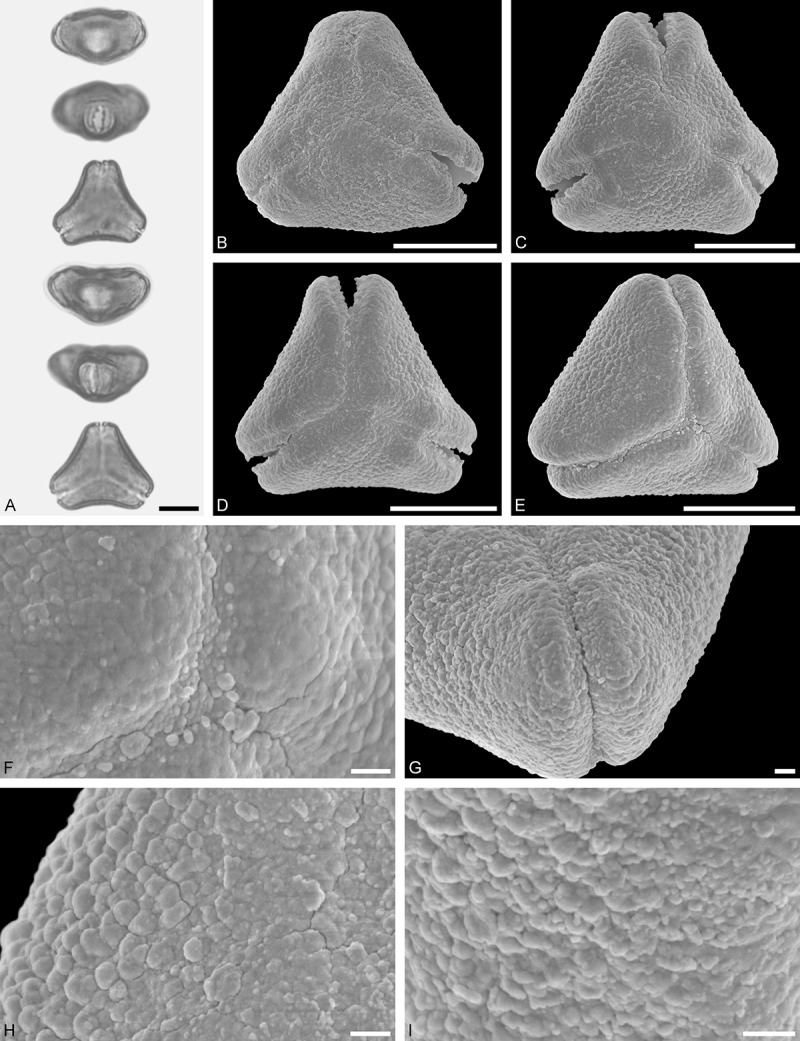
LM (A) and SEM (B–I) micrographs of *Loranthus europaeus* (WU 060561). **A.** Two pollen grains in equatorial and polar view. **B–E.** Pollen grains in polar view. **F.** Close-up of central polar area. **G.** Close-up of apex. **H, I.** Close-ups of mesocolpium. Scale bars – 10 µm (A–E), 1 µm (F–I).

*Loranthus europaeus* Jacq. (Figure 46)

#### Description

Pollen, oblate, straight-triangular in polar view, elliptic to slightly bean-shaped in equatorial view, equatorial apices obcordate; size small, polar axis 13.3–15.0 µm long in LM, equatorial diameter 20.0–23.3 µm in LM, 19.5–23.9 µm in SEM; syn-(3)colpate (isopolar grains) or syn-/zono-(3)colpate, colpi very long (heteropolar grains); exine 1.2–1.7 µm thick, nexine thinner than sexine, nexine thickened in polar area, sexine thickened in area of mesocolpium (LM); tectate; sculpturing psilate in LM, micro-verrucate and granulate, perforate in area of mesocolpium in SEM, verrucae 0.5–1.1 µm in diameter; margo indistinct, margo micro-verrucate and partly granulate; colpus membrane nano-verrucate to nano-rugulate and granulate (SEM).

#### Remark

Pollen Type B. The pollen of both species analysed here is very similar. Isopolar (elliptic in equatorial outline) and heteropolar grains (bean-shaped) can be found. Diagnostic features of the compact *Loranthus* pollen are the straight-triangular outline in polar view and thick walls compared to size (thickening of mesocolpial sexine and polar nexine).

Scurrulinae

#### Remark

A relatively large tribe with only two, but speciose genera: the east to southeast Asian *Scurrula* with 43 species; and the eastern African to tropical Asian *Taxillus* with *c*. 35 species (File S3). Pollen grains of the two genera figured so far and herein are very similar, and even using SEM it is impossible to differentiate between genera or species using pollen morphology. Notably all studied species show the entire range of (slightly) convex-triangular to trilobate pollen (polar view). They differ from other Lorantheae by their well-developed hexagonally thickened polar nexine (visible in LM). Grains are always nano=verrucate to granulate, both in the mesocolpium and colpus membrane. The margo is always very distinct and mostly psilate.

**Figure 47.**
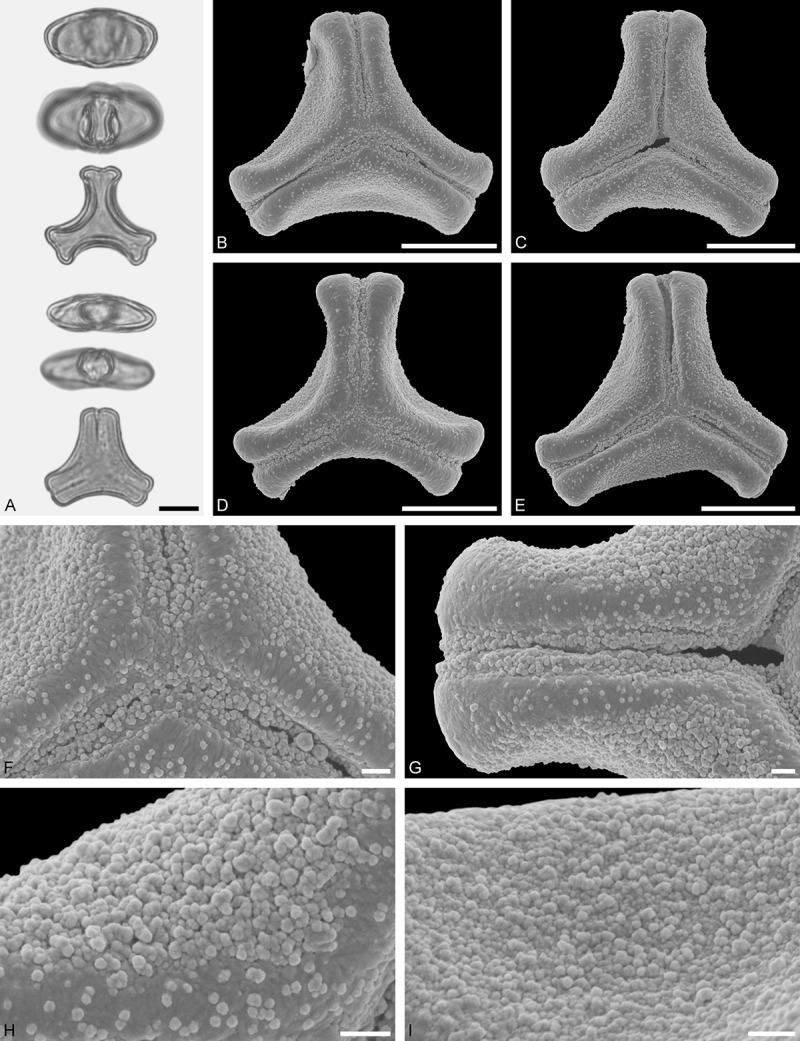
LM (A) and SEM (B–I) micrographs of *Scurrula parasitica* (WU 039110). **A.** Two pollen grains in equatorial and polar view. **B–E.** Pollen grains in polar view. **F.** Close-up of central polar area. **G.** Close-up of apex. **H, I.** Close-ups of mesocolpium. Scale bars – 10 µm (A–E), 1 µm (F–I).

*Scurrula parasitica* L. (Figure 47)

#### Description

Pollen, oblate, trilobate to concave-triangular in polar view, elliptic in equatorial view, equatorial apices obcordate to T-shaped; size small, polar axis 10.0–15.0 µm long in LM, equatorial diameter 18.3–25.0 µm in LM, 19.5–22.7 µm in SEM; syn-(3)colpate; exine 1.1–1.3 µm thick, nexine thinner than sexine, nexine thickened in polar area (LM); tectate; sculpturing psilate in LM, nano-verrucate to granulate in area of mesocolpium in SEM, verrucae 0.1–0.5 µm in diameter, verrucae composed of conglomerate granula; margo well developed, margo psilate or partly nano-verrucate (SEM); colpus membrane nano-verrucate and granulate (SEM). – Pollen Type B.

**Figure 48.**
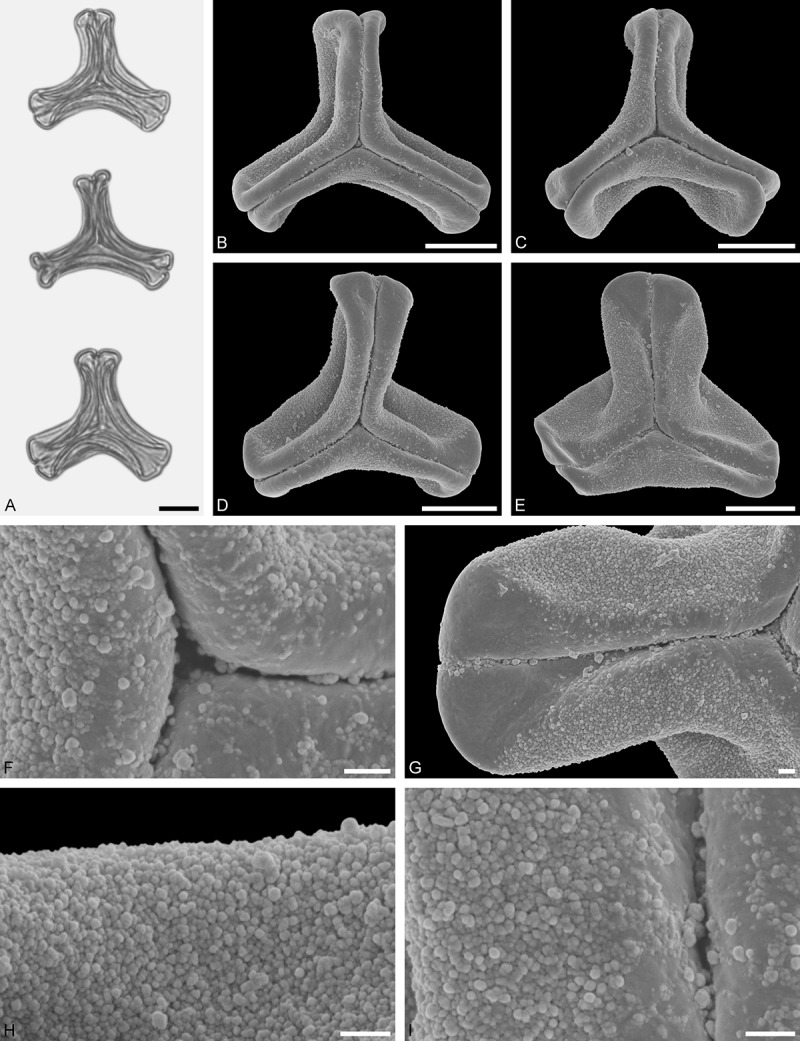
LM (A) and SEM (B–I) micrographs of *Taxillus aldabrensis* (WU: from Madagascar, coll. J. M. Hildebrandt, s.n.). **A.** Three pollen grains in polar view. **B–E.** Pollen grains in polar view. **F.** Close-up of central polar area. **G.** Close-up of apex. **H, I.** Close-ups of mesocolpium. Scale bars – 10 µm (A–E), 1 µm (F–I).

*Taxillus aldabrensis* (Turill) Danser (Figure 48)

#### Description

Pollen, oblate, trilobate in polar view, elliptic in equatorial view, equatorial apices obcordate to T-shaped; size small to medium, polar axis 13.5–15.6 µm long in LM, equatorial diameter 25.0–28.3 µm in LM, 21.3–31.4 µm in SEM; syn-(3)colpate; exine 1.0–1.3 µm thick, nexine thinner than sexine, nexine thickened in polar area (LM); tectate; sculpturing psilate in LM, nano-verrucate to granulate in area of mesocolpium in SEM, verrucae 0.1–0.5 µm in diameter, verrucae composed of conglomerate granula; margo well developed, most pronounced at apices, margo psilate with few nanoverrucae or granula in polar area (SEM); colpus membrane nano-verrucate and granulate (SEM). – Pollen Type B.

**Figure 49.**
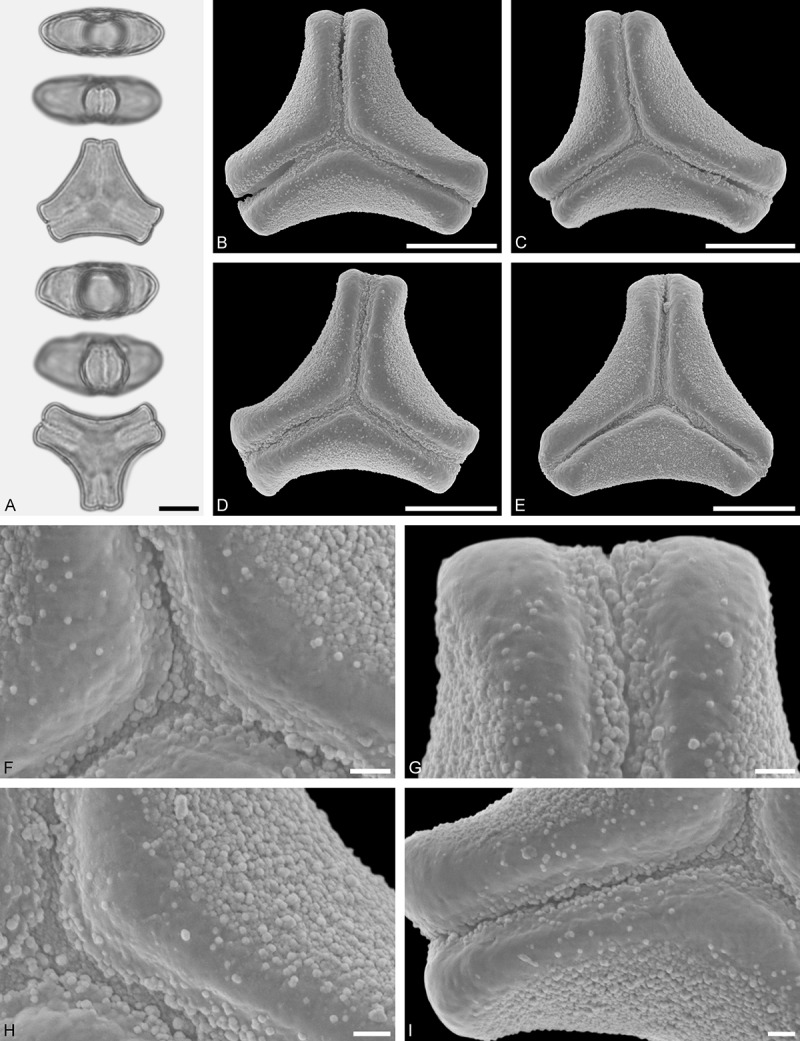
LM (A) and SEM (B–I) micrographs of *Taxillus caloreas* (WU 039396). **A.** Two pollen grains in equatorial and polar view. **B–E.** Pollen grains in polar view. **F.** Close-up of central polar area. **G.** Close-up of apex. **H.** Close-up of mesocolpium. **I.** Close-up of apex and mesocolpium. Scale bars – 10 µm (A–E), 1 µm (F–I).

*Taxillus caloreas* (Diels) Danser (Figure 49)

#### Description

Pollen, distinctly oblate, concave-triangular in polar view, elliptic in equatorial view, equatorial apices obcordate; size small to medium, polar axis 11.7–15.0 µm long in LM, equatorial diameter 23.3–30.0 µm in LM, 21.1–26.0 µm in SEM; syn-(3)colpate; exine 1.0–1.3 µm thick, nexine thinner than sexine, hexagonal nexine thickening in polar area (LM); tectate; sculpturing psilate in LM, nano-verrucate to granulate in area of mesocolpium in SEM, verrucae 0.1–0.5 µm in diameter, verrucae composed of conglomerate granula; margo well developed, margo psilate with few nanoverrucae or granula in polar area (SEM); colpus membrane nano-verrucate and granulate (SEM). – Pollen Type B.

**Figure 50.**
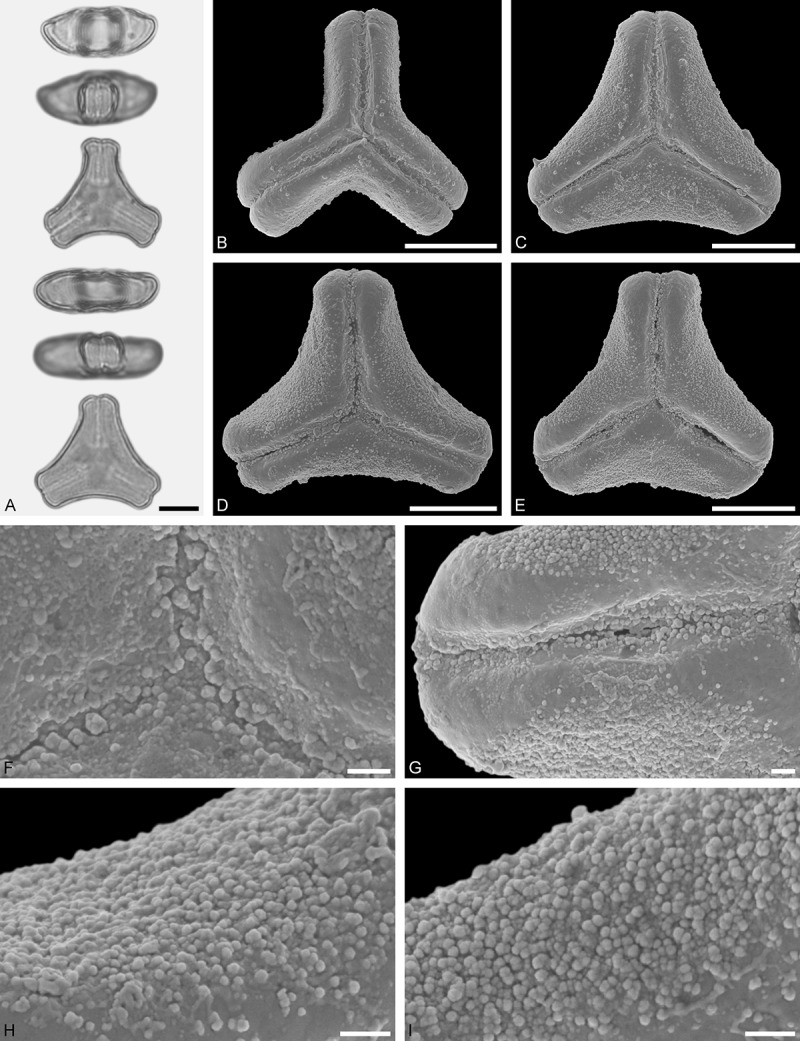
LM (A) and SEM (B–I) micrographs of *Taxillus delavayi* (WU 039090). **A.** Two pollen grains in equatorial and polar view. **B–E.** Pollen grains in polar view. **F.** Close-up of central polar area. **G.** Close-up of apex. **H, I.** Close-ups of mesocolpium. Scale bars – 10 µm (A–E), 1 µm (F–I).

*Taxillus delavayi* (Tiegh.) Danser (Figure 50)

#### Description

Pollen, distinctly oblate, trilobate to concave-triangular in polar view, elliptic in equatorial view, equatorial apices obcordate; size small to medium, polar axis 10.0–13.3 µm long in LM, equatorial diameter 21.7–26.7 µm in LM, 18.9–27.8 µm in SEM; syn-(3)colpate; exine 1.0–1.3 µm thick, nexine thinner than sexine, hexagonal nexine thickening in polar area (LM); tectate; sculpturing psilate in LM, nano-verrucate to granulate in area of mesocolpium in SEM, verrucae 0.1–0.4 µm in diameter, verrucae composed of conglomerate granula; margo well developed, margo psilate with few nanoverrucae or granula, margo sometimes with triangular protrusion in polar area (SEM); colpus membrane nano-verrucate and granulate (SEM). – Pollen Type B.

Tapinanthinae

#### Remark

The African-Arabian Tapinanthinae represents the largest subtribe of the Lorantheae with 14 genera and more than 170 species (File S3). We here present the first palynological data on six genera (13 species). Table X includes also information on two additional (Madagascan) genera based on grains figured in Muller et al. (1989).

**Table X.** Tabulation of differentiating pollen features in Lorantheae, part 3: Tapinanthinae (part of Clade J; Figure 8).

Genus	Polarity, aperture	Size, shape, outline in e.v. and p.v.^a^	Equatorial apices	Margo	Further exine features (LM, SEM)	Sculpturing
*Actinanthella*	Isopolar, **zono(3)colpate**	Medium, distinctly oblate, **emarginate**, concave-triangular to trilobate	Obcordate	Distinct	LM: nexine thickened in polar area	CM: ? MC: nano-verrucate to granulate M: mostly psilate
*Agelanthus bruneus, A. scassellatii*	Isopolar, **zono(3)colpate**	Medium, (distinctly) oblate, elliptic, straight-to concave-triangular	Obcordate or **T-shaped**	Distinct, **covering the entire apices**	**SEM: protruding polar dome**	CM: ? MC: nano- to micro-baculate/-echinate, granulate M: mostly psilate
*A. discolor* (formerly *Tapinanthus*)	Isopolar, **zono(3)colpate**	Medium, oblate, elliptic, concave-triangular to trilobate,	Obcordate	Distinct	LM: nexine thickened in polar area	CM: granulate MC: nano-verrucate to granulate M: psilate to nano-verrucate
*Bakerella*	Isopolar, syn(3)colpate	Medium, oblate, elliptic, concave-triangular to trilobate	Obcordate	Distinct	**LM: nexine can show triangular intercolpial thickenings**	CM: ? MC: nano- to micro-verrucate M: mostly psilate
*Englerina*	Isopolar, **zono(3)colpate**	Small to medium, (distinctly) oblate, elliptic, straight- or concave-triangular/trilobate	Obcordate	Indistinct or distinct	**SEM: ± protruding (small) polar dome**	CM: ? MC: nano- to micro-verrucate/-echinate, granulate M: As MC or psilate
*Oncocalyx*	Isopolar, **zono(3)colpate**	Medium, oblate, variable, straight- to concave-triangular	Obcordate	Distinct	LM: nexine can be thickened in polar area	CM: granulate (*O. welwitschii*) MC: nano-verrucate(-echinate) to granulate M: mostly psilate
*Plicosepalus acaciae, P. curviflorus*	Isopolar, syn(3)colpate	Small, oblate, elliptic, straight- or concave-triangular to trilobate	Obcordate	Distinct	**LM: triradial polar nexine thickening** (*P. curviflorus*)	CM, MC: (nano-verrucate), granulate M: segmented (*P. acaciae*); psilate to weakly micro-verrucate (*P. curviflorus*)
*P. sagittifolius*	**Heteropolar**, p.f.: syn(3)colpate, d.f.: zono(3)colpate	Medium, oblate, **bean-shaped**, concave-triangular	Obcordate	Distinct	**LM: triradial polar nexine thickening**	CM: nano-verrucate to granulate MC: nano- to micro-verrucate, verrucae increasing in size and decreasing in density from p.f. to pole of d.f. M: psilate
*Socratina*	Isopolar, syn(3)colpate	Medium, oblate, elliptic, concave-triangular to trilobate	Obcordate	Distinct	**LM: nexine with triangular intercolpial thickenings**	CM: ? MC: nano- to micro-verrucate M: mostly psilate
*Tapinanthus bangwensis, T. ogowensis*	Isopolar, **demisyn(3)colpate**	Medium, oblate, elliptic to **subrhombic**, trilobate	**T-shaped**	Very broad	LM: sexine thickened in MC (*T. ogowensis*), nexine hexagonally thickened in polar area	CM: nano-verrucate to granulate MC: nano- to micro-baculate/-echinate, granulate M: mostly psilate

Note: Abbreviations: e.v., equatorial view; p.v., polar view; p.f., proximal face; d.f., distal face; LM, light microscopy; SEM, scanning electron microscopy; CM, colpus membrane; MC, mesocolpium; M, margo; ^a^ Size: small, <25 µm; medium, 25–50 µm; shape: oblate, less than two-times wider than high; distinctly oblate, more than two-times wider than high.

**Figure 51.**
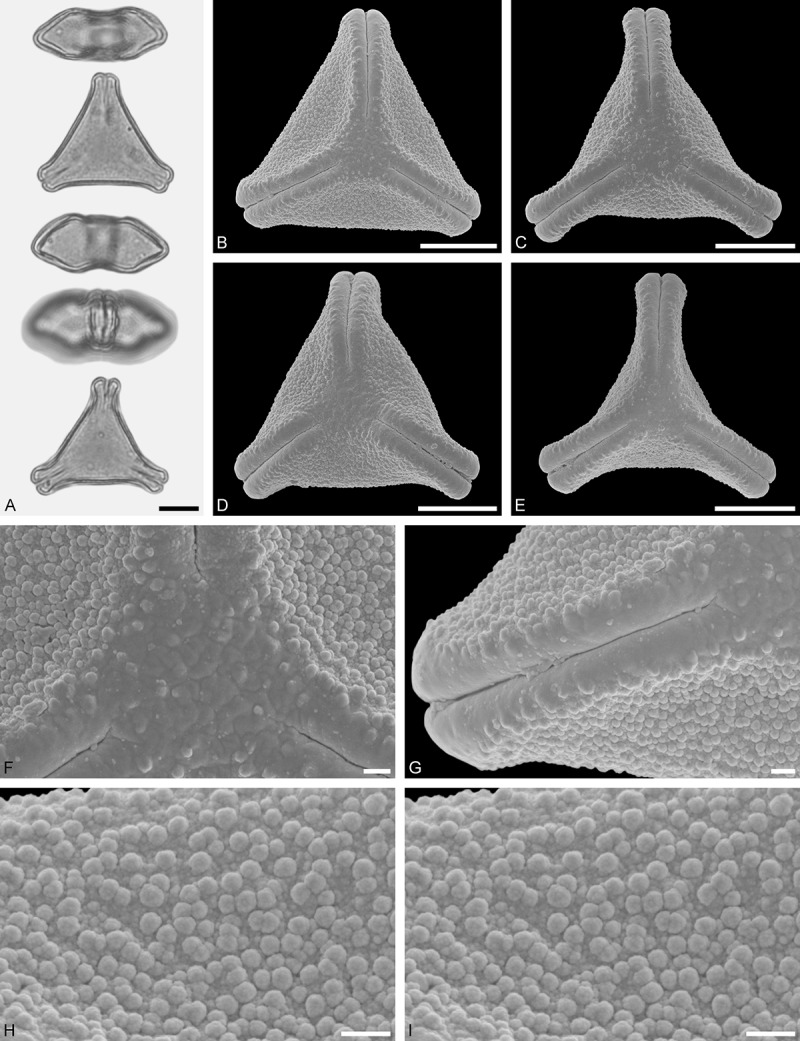
LM (A) and SEM (B–I) micrographs of *Actinanthella menyhartii* (WU 0029707). **A.** Two pollen grains in equatorial and polar view. **B–E.** Pollen grains in polar view. **F.** Close-up of central polar area. **G.** Close-up of apex. **H, I.** Close-ups of mesocolpium. Scale bars – 10 µm (A–E), 1 µm (F–I).

*Actinanthella menyhartii* (Engl. et Schinz ex Schinz) Balle (Figure 51)

#### Description

Pollen, distinctly oblate, trilobate to concave-triangular in polar view, emarginate in equatorial view, equatorial apices obcordate; size medium, polar axis 10.0–15.0 µm long in LM, equatorial diameter 25.0–30.0 µm in LM, 22.4–29.1 µm in SEM; zono-(3)colpate, colpi very long; exine 1.1–1.5 µm thick, nexine thinner than sexine, nexine thickened in central polar area (LM); tectate; sculpturing psilate in LM, nano-verrucate to granulate in area of mesocolpium in SEM, verrucae 0.2–0.5 µm in diameter, verrucae composed of conglomerate granula; margo well developed, margo psilate with few microverrucae (SEM).

#### Remark

Pollen Type B. The grains differ from the other zonocolpate members of the Tapinanthinae (*Agelanthus*, *Englerina*, *Oncocalyx*) by their nano-verrucate to granulate sculpturing and the occurrence of modified trilobate pollen (Figure 51A, lower grain; Figure 51C, E).

**Figure 52.**
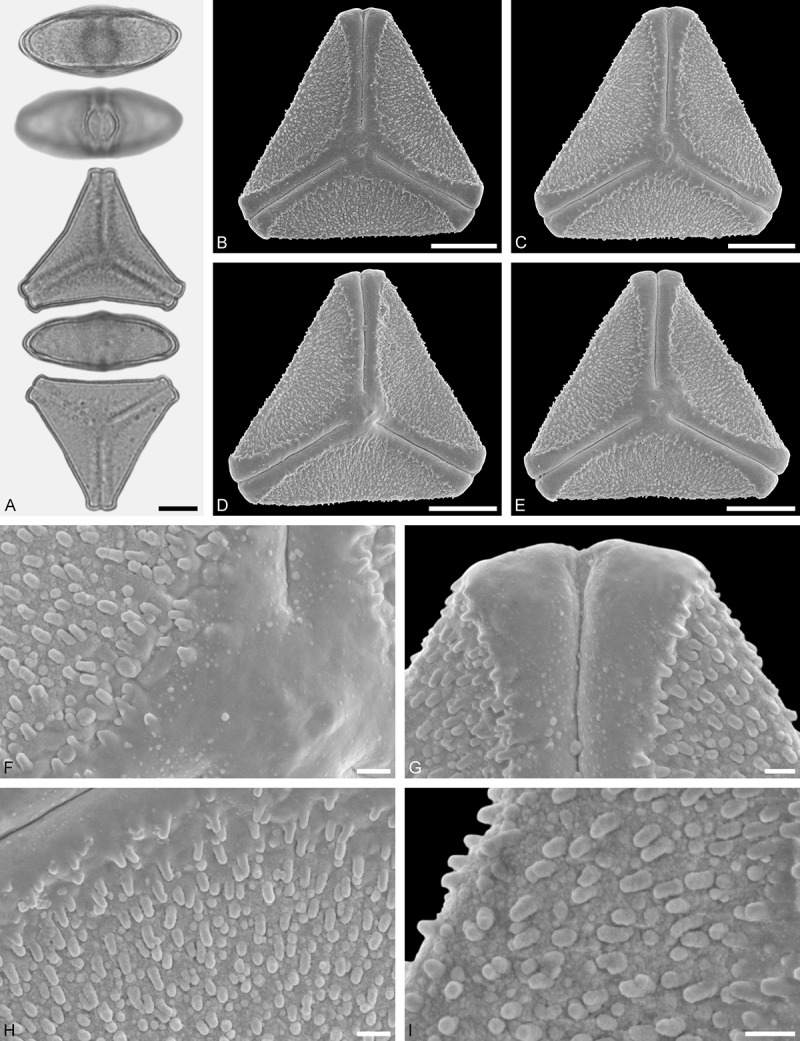
LM (A) and SEM (B–I) micrographs of *Agelanthus brunneus* (WU 039037). **A.** Two pollen grains in equatorial and polar view. **B–E.** Pollen grains in polar view. **F.** Close-up of central polar area. **G.** Close-up of apex. **H, I.** Close-ups of mesocolpium. Scale bars – 10 µm (A–E), 1 µm (F–I).

*Agelanthus brunneus* (Engl.) Tiegh. (Figure 52)

#### Description

Pollen, distinctly oblate, straight-triangular in polar view, elliptic in equatorial view, equatorial apices obcordate; size medium, polar axis 10.8–18.3 µm long in LM, equatorial diameter 28.3–36.7 µm in LM, 31.4–35.7 µm in SEM; zono-(3)colpate, colpi very long; exine 1.0–1.5 µm thick, nexine thinner than sexine, nexine thickened in central polar area (LM); tectate; sculpturing psilate in LM, nano-/micro-echinate to nano-/micro-baculate and granulate in area of mesocolpium in SEM, echini/bacula 0.3–0.9 µm long, 0.2–0.4 µm wide at base (SEM); margo well developed, widening in equatorial region, margo psilate (SEM); exine in central polar area protruding forming a small dome (SEM). – Pollen Type B.

#### Note

Pollen surface features seen in the LM micrographs are not reflecting sculptural elements but cell contents and flower material, due to incomplete acetolisation. Fully processed grains had the tendency to rupture and lose their form, so we opted to interrupt the acetolising process for LM photography.

**Figure 53.**
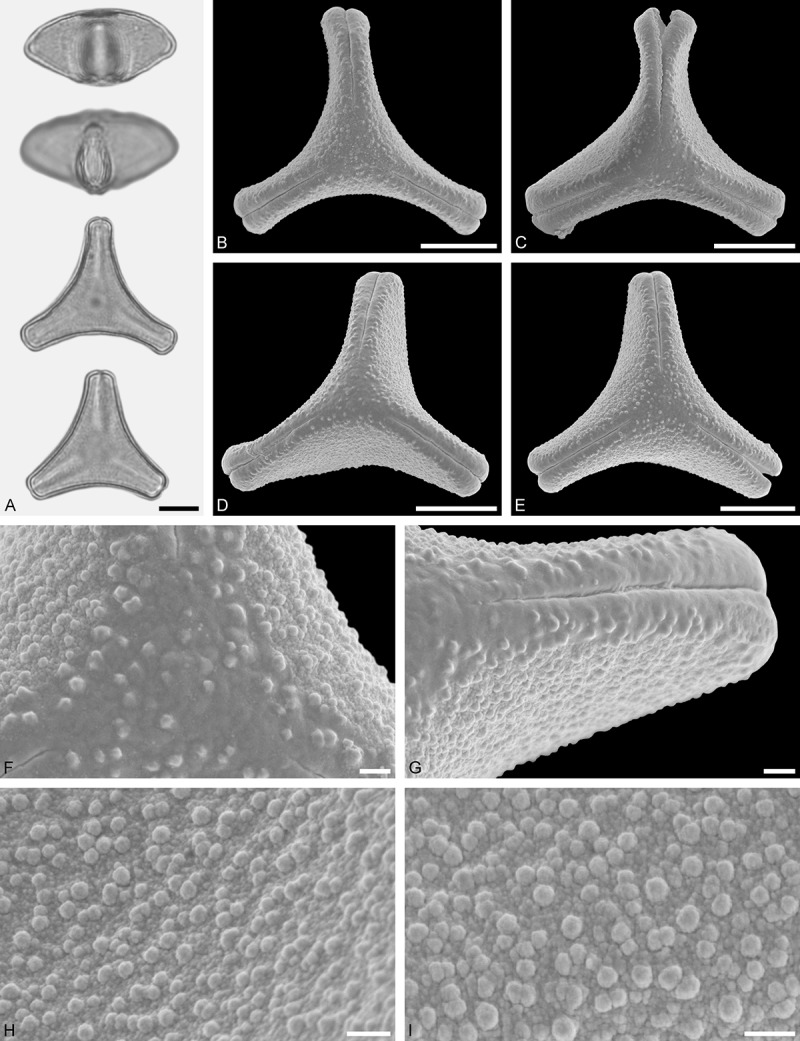
LM (A) and SEM (B–I) micrographs of *Agelanthus discolor* (WU 039038). **A.** Two pollen grains, one in equatorial and polar view, other in polar view. **B–E.** Pollen grains in polar view. **F.** Close-up of central polar area. **G.** Close-up of apex. **H, I.** Close-ups of mesocolpium. Scale bars – 10 µm (A–E), 1 µm (F–I).

*Agelanthus discolor* (Schinz) Balle [= *Tapinanthus discolor* (Schinz) Danser] (Figure 53)

#### Description

Pollen, oblate, trilobate to concave-triangular in polar view, elliptic in equatorial view, equatorial apices obcordate; size medium, polar axis 16.7–18.3 µm long in LM, equatorial diameter 26.7–33.3 µm in LM, 23.0–26.5 µm in SEM; zono-(3)colpate, colpi very long; exine 1.1–1.5 µm thick, nexine thinner than sexine, nexine thickened in central polar area (LM); tectate; sculpturing psilate in LM, nano-verrucate to granulate in area of mesocolpium and central polar area in SEM, verrucae 0.3–0.6 µm in diameter, verrucae composed of conglomerate granula; margo fairly well developed, margo psilate to nano-verrucate (SEM); colpus membrane granulate (SEM).

#### Remark

Pollen Type B. The species used to be included in the genus *Tapinanthus* (Tropicos.org 2016), but it belongs into *Agelanthus* (anonymous reviewer, personal communication, 2016). The pollen is indeed distinct from the unique pollen in the other two species of *Tapinanthus* (*T. bangwensis*, *T. ogowensis*). More similar to the pollen of its new congeners, it differs from the pollen of the two other *Agelanthus* species by its sculpturing. However, it resembles strongly the pollen of *Oncocalyx*, which is resolved as a close relative of *Agelanthus* based on the limited molecular data available so far for African Loranthaceae (Figures 2, 3; Su et al. 2015).

**Figure 54.**
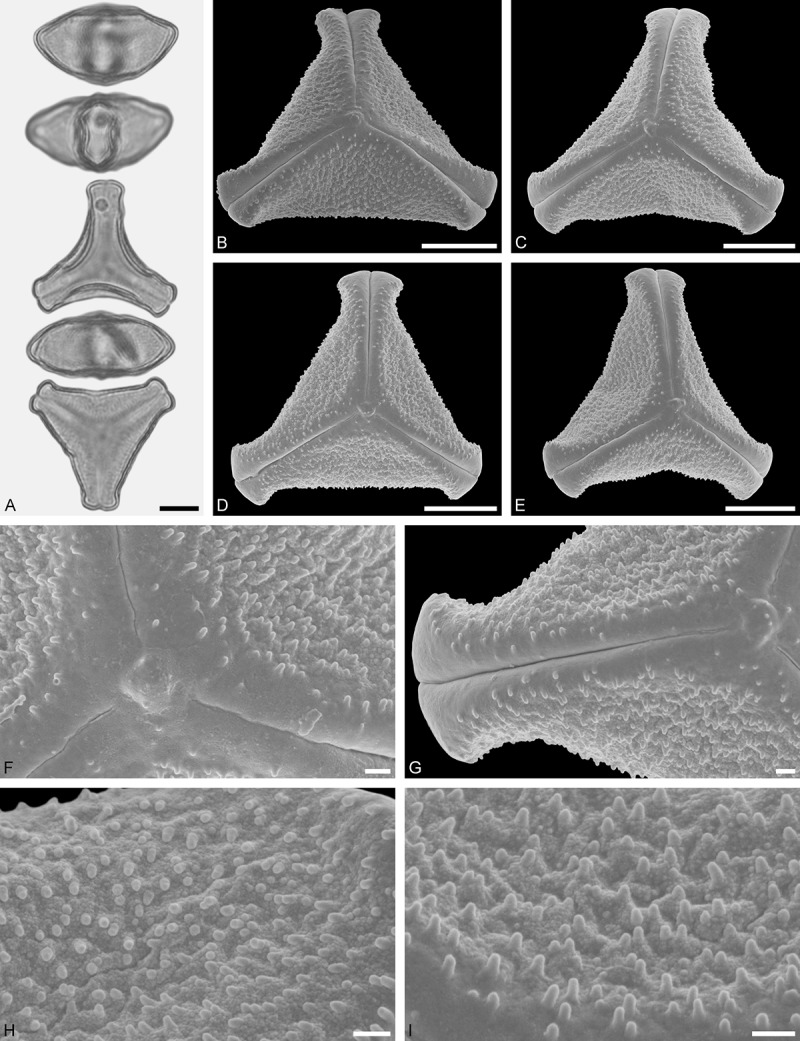
LM (A) and SEM (B–I) micrographs of *Agelantus scassellatii* (WU 004299). **A.** Two pollen grains in equatorial and polar view. **B–E.** Pollen grains in polar view. **F.** Close-up of central polar area. **G.** Close-up of apex. **H, I.** Close-ups of mesocolpium. Scale bars – 10 µm (A–E), 1 µm (F–I).

*Agelantus scassellatii* (Chiov.) Polhill et Wiens (Figure 54)

#### Description

Pollen, oblate, straight-triangular to concave-triangular in polar view, elliptic in equatorial view, equatorial apices T-shaped; size medium, polar axis 11.7–20.0 µm long in LM, equatorial diameter 26.7–35.0 µm in LM, 27.7–32.0 µm in SEM; zono-(3)colpate, colpi very long; exine 1.1–1.5 µm thick, nexine thinner than sexine, nexine thickened in central polar area (LM); tectate; sculpturing psilate in LM, nano-echinate to nano-baculate and granulate in area of mesocolpium in SEM, echini/bacula 0.2–0.6 µm long, 0.2–0.3 µm wide at base (SEM); margo well developed, widening in equatorial region, margo psilate with few nanoechini/bacula (SEM); exine in central polar area protruding forming a small dome (SEM).

#### Remark

Pollen Type B. Pollen of both *Agelanthus* *bruneus* and *A. scassellatii* are very similar. The only difference is that the equatorial apices are often T-shaped in *A. scassellatii*. A diagnostic feature of both species is the minute, protruding dome seen at the pole, at the junction of the colpi (SEM; Figures 52B–E, 54B–F).

#### Note

Pollen surface features seen in the LM micrographs are not reflecting sculptural elements but cell contents and flower material, due to incomplete acetolisation. Fully processed grains had the tendency to rupture and lose their form, so we opted to interrupt the acetolising process for LM photography.

**Figure 55.**
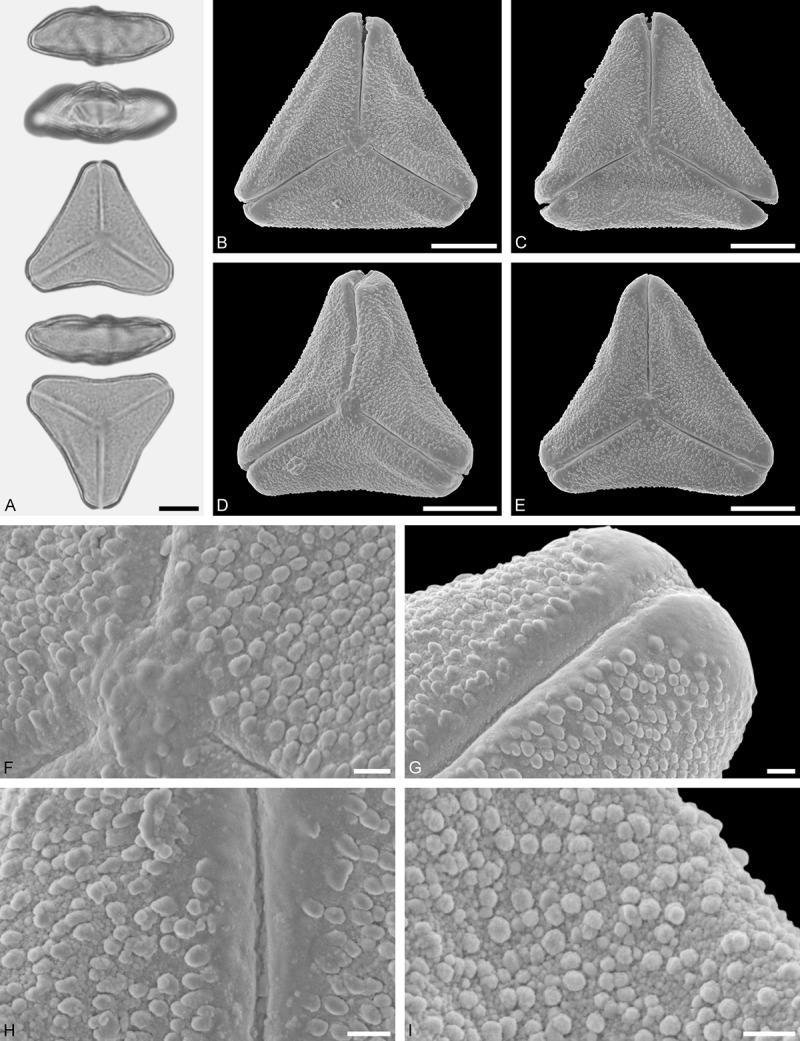
LM (A) and SEM (B–I) micrographs of *Englerina holstii* (WU: from East Africa, coll. J. Brunnthaler, s.n.). **A.** Two pollen grains in equatorial and polar view. **B–E.** Pollen grains in polar view. **F.** Close-up of central polar area. **G.** Close-up of apex. **H, I.** Close-ups of mesocolpium. Scale bars – 10 µm (A–E), 1 µm (F–I).

*Englerina holstii* (Engl.) Tiegh (Figure 55)

#### Description

Pollen, distinctly oblate, straight-triangular in polar view, elliptic in equatorial view, equatorial apices obcordate; size medium, polar axis 11.7–13.3 µm long in LM, equatorial diameter 33.3–36.7 µm in LM, 27.7–34.8 µm in SEM; zono-(3)colpate, colpi very long; exine 1.2–1.5 µm thick, nexine thinner than sexine (LM); tectate; sculpturing psilate in LM, nano-/micro-verrucate to nano-/micro-echinate and granulate in area of mesocolpium in SEM, verrucae/echini 0.2–0.9 µm long, 0.1–0.5 µm wide at base (SEM); margo indistinct, widening in equatorial region, margo nano-/micro-verrucate to nano-/micro-echinate and granulate in polar area, margo psilate in equatorial region (SEM); exine in central polar area protruding forming a small dome (SEM). – Pollen Type B.

**Figure 56.**
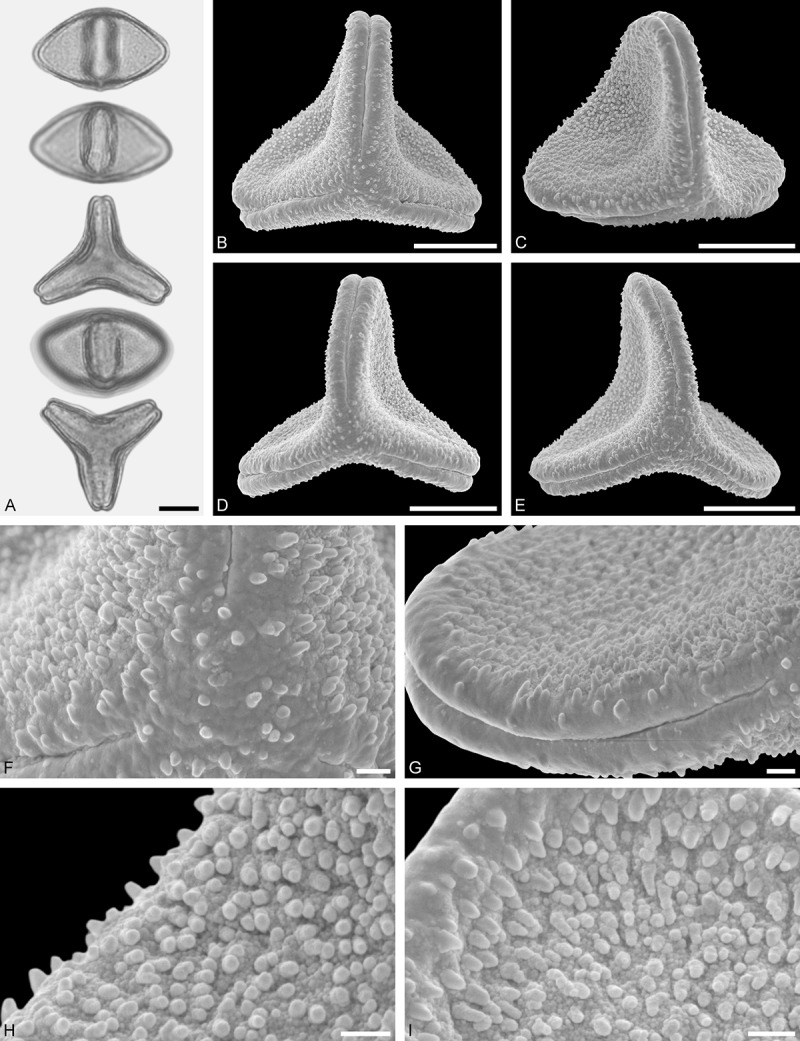
LM (A) and SEM (B–I) micrographs of *Englerina oedostemon* (WU: from Zimbabwe, collector unknown, det. W. Frostreder[?], s.n.). **A.** Two pollen grains in equatorial and polar view. **B–E.** Pollen grains in polar view. **F.** Close-up of central polar area. **G.** Close-up of apex. **H, I.** Close-ups of mesocolpium. Scale bars – 10 µm (A–E), 1 µm (F–I).

*Englerina oedostemon* (Danser) Polhill et Wiens (Figure 56)

#### Description

Pollen, oblate, trilobate to concave-triangular in polar view, elliptic in equatorial view, equatorial apices obcordate; size small to medium, polar axis 16.7–21.7 µm long in LM, equatorial diameter 23.3–26.7 µm in LM, 18.5–24.7 µm in SEM; zono-(3)colpate, colpi very long; exine 1.3–1.5 µm thick, nexine thinner than sexine, nexine thickened in central polar area (LM); tectate; sculpturing psilate in LM, micro-echinate to nano-echinate and granulate in area of mesocolpium and central polar area in SEM, echini 0.3–0.9 µm long, 0.2–0.4 µm wide at base (SEM); margo well developed, margo psilate with few microechini (SEM); exine in central polar area protruding slightly (SEM).

#### Remark

Pollen Type B. Regarding shape, outlines in equatorial and polar views, *Englerina holstii* is much more similar to *Agelanthus* than *Englerina oedostemon*. Regarding sculpturing the opposite is true. The grains differ from those of the putative sister genus of *Englerina*, *Tapinanthus.*

**Figure 57.**
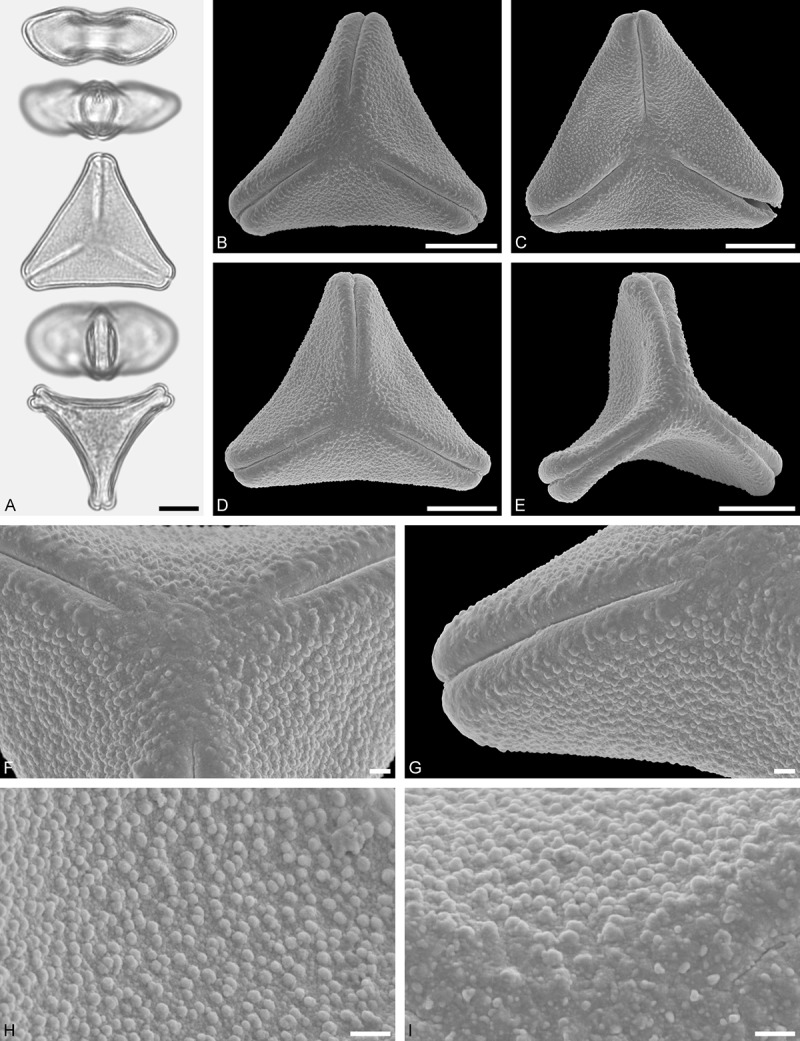
LM (A) and SEM (B–I) micrographs of *Oncocalyx schimperi* (WU 037621). **A.** Two pollen grains in equatorial and polar view. **B–E.** Pollen grains in polar view. **F.** Close-up of central polar area. **G.** Close-up of apex. **H, I.** Close-ups of mesocolpium. Scale bars – 10 µm (A–E), 1 µm (F–I).

*Oncocalyx schimperi* (Hochst. ex A.Rich.) M.G.Gilbert (Figure 57)

#### Description

Pollen, oblate, straight-triangular to concave-triangular in polar view, slightly emarginate in equatorial view, equatorial apices obcordate; size medium, polar axis 11.7–21.7 µm long in LM, equatorial diameter 28.3–36.7 µm in LM, 23.9–34.3 µm in SEM; zono-(3)colpate, colpi very long; exine 1.1–1.5 µm thick, nexine thinner than sexine (LM); tectate; sculpturing psilate in LM, nano-verrucate to nano-echinate and granulate in area of mesocolpium in SEM, verrucae/echini 0.2–0.5 µm long, 0.2–0.5 µm wide at base, verrucae/echini composed of conglomerate granula; margo well developed, margo psilate and partly nano-verrucate/echinate to granulate (SEM). – Pollen Type B.

#### Note

Pollen surface features seen in the LM micrographs are not reflecting sculptural elements but cell contents and flower material, due to incomplete acetolisation. Fully processed grains had the tendency to rupture and lose their form, so we opted to interrupt the acetolising process for LM photography.

**Figure 58.**
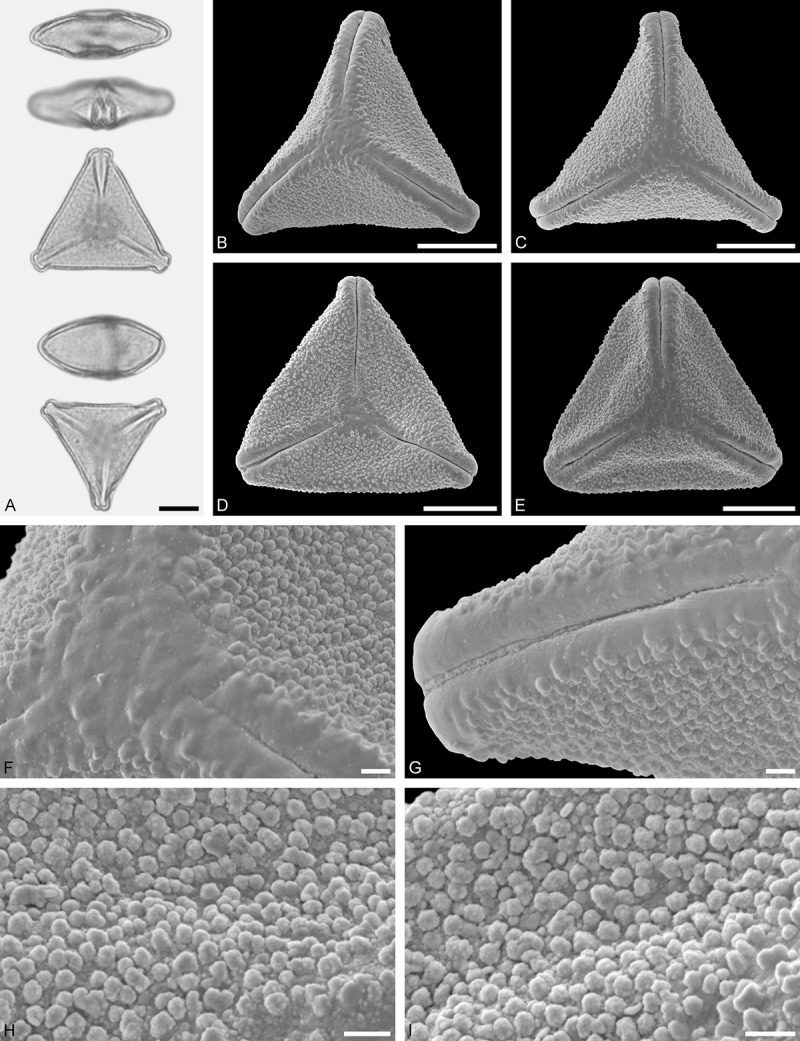
LM (A) and SEM (B–I) micrographs of *Oncocalyx welwitschii* (WU 039064). **A.** Two pollen grains in equatorial and polar view. **B–E.** Pollen grains in polar view. **F.** Close-up of central polar area. **G.** Close-up of apex. **H, I.** Close-ups of mesocolpium. Scale bars – 10 µm (A–E), 1 µm (F–I).

*Oncocalyx welwitschii* (Engl.) Polhill et Wiens (Figure 58)

#### Description

Pollen, oblate, straight-triangular to concave-triangular in polar view, elliptic to rhombic in equatorial view, equatorial apices obcordate; size medium, polar axis 11.7–18.3 µm long in LM, equatorial diameter 25.0–31.7 µm in LM, 26.1–30.3 µm in SEM; zono-(3)colpate, colpi very long; exine 1.0–1.3 µm thick, nexine thinner than sexine, nexine thickened in central polar area (LM); tectate; sculpturing psilate in LM, nano-verrucate to granulate in area of mesocolpium in SEM, verrucae 0.3–0.6 µm in diameter, verrucae composed of conglomerate granula; margo well developed, margo psilate; colpus membrane granulate (SEM).

#### Remark

Pollen Type B. Being very similar in general appearance, the sculpturing elements are as a trend larger in *Oncocalyx welwitschii* and *O. schimperi*. The general type is shared with the other taxa with zonocolpate grains (*Agelanthus, Actinanthella*; Figures 51–54), resolved as close relatives *Oncocalyx* based on molecular data (Figure 8).

**Figure 59.**
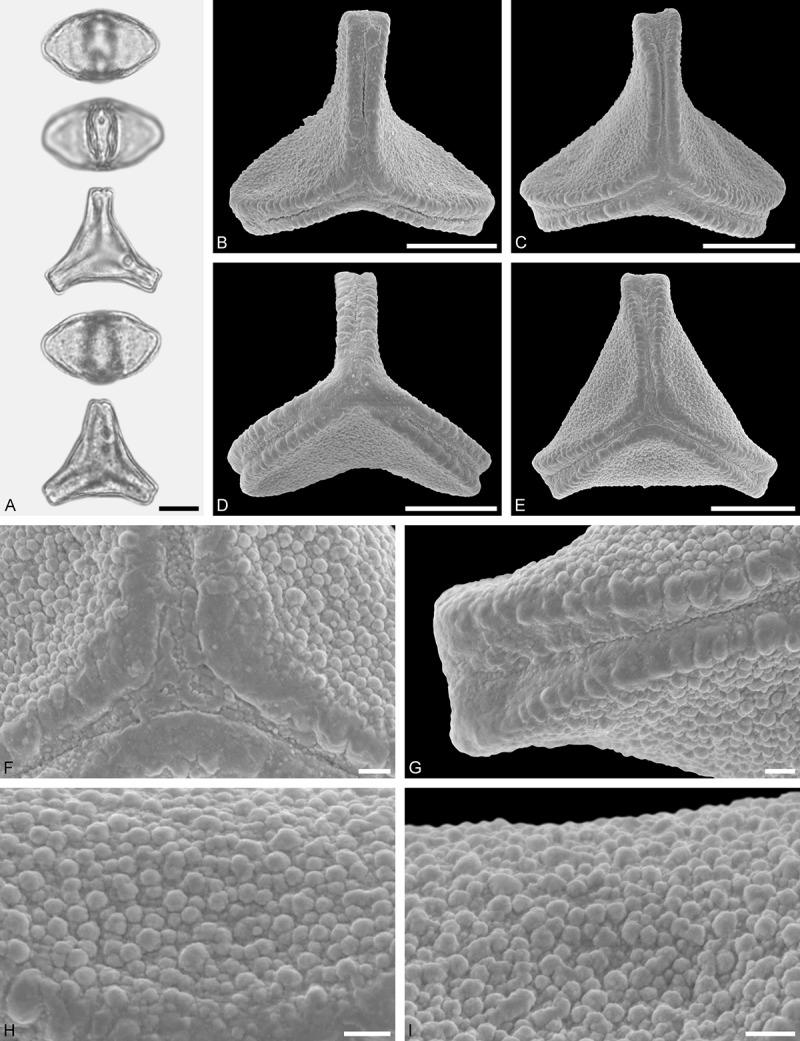
LM (A) and SEM (B–I) micrographs of *Plicosepalus acacia* (WU: from Palestine, coll. H. Boyko, s.n.). **A.** Two pollen grains in equatorial and polar view. **B–E.** Pollen grains in polar view. **F.** Close-up of central polar area. **G.** Close-up of apex. **H, I.** Close-ups of mesocolpium. Scale bars – 10 µm (A–E), 1 µm (F–I).

*Plicosepalus acaciae* (Zucc.) Wiens et Polhill (Figure 59)

#### Description

Pollen, oblate, trilobate to straight-triangular in polar view, elliptic in equatorial view, equatorial apices obcordate; size small to medium, polar axis 16.7–20.0 µm long in LM, equatorial diameter 23.3–28.3 µm in LM, 19.5–25.5 µm in SEM; syn-(3)colpate; exine 1.1–1.5 µm thick, nexine thinner than sexine (LM); tectate; sculpturing psilate in LM, micro-verrucate to nano-verrucate and granulate in area of mesocolpium in SEM, verrucae 0.2–0.7 µm in diameter, verrucae composed of conglomerate granula; margo well developed, margo segmented perpendicular to colpi (SEM); colpus membrane nano-verrucate and granulate (SEM). – Pollen Type B.

#### Note

Pollen surface features seen in the LM micrographs are not reflecting sculptural elements but cell contents and flower material, due to incomplete acetolisation. Fully processed grains had the tendency to rupture and lose their form, so we opted to interrupt the acetolising process for LM photography.

**Figure 60.**
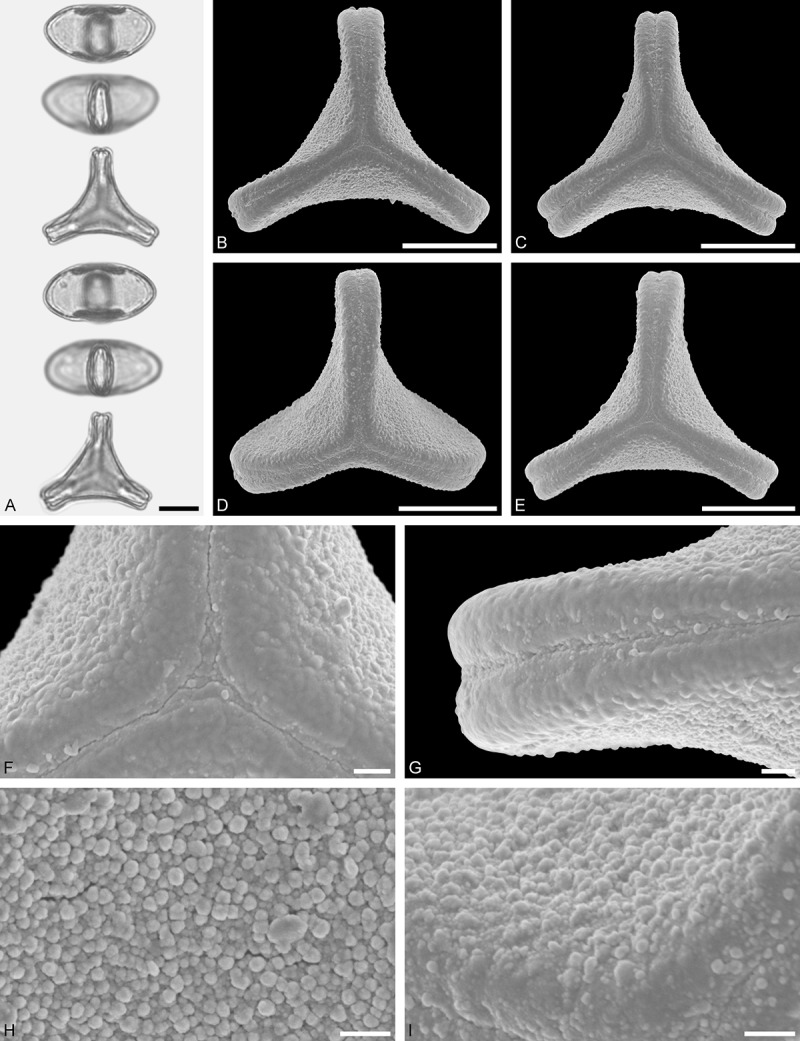
LM (A) and SEM (B–I) micrographs of *Plicosepalus curviflorus* (WU: from Kenya, coll. J. B. Gillett, s.n.). **A.** Two pollen grains in equatorial and polar view. **B–E.** Pollen grains in polar view. **F.** Close-up of central polar area. **G.** Close-up of apex. **H, I.** Close-ups of mesocolpium. Scale bars – 10 µm (A–E), 1 µm (F–I).

*Plicosepalus curviflorus* (Benth. ex Oliv.) Tiegh (Figure 60)

#### Description

Pollen, oblate, trilobate to concave-triangular in polar view, elliptic in equatorial view, equatorial apices obcordate; size small, polar axis 11.7–15.0 µm long in LM, equatorial diameter 20.0–25.0 µm in LM, 18.1–21.6 µm in SEM; syn-(3)colpate; exine 0.9–1.3 µm thick, nexine thinner than sexine, triradial nexine thickening in polar area (LM); tectate; sculpturing psilate in LM, nano-verrucate and granulate in area of mesocolpium in SEM, verrucae 0.1–0.4 µm in diameter, verrucae composed of conglomerate granula; margo well developed, margo psilate to weakly micro-rugulate (SEM); colpus membrane granulate (SEM).

#### Remark

Pollen Type B. Pollen of *Plicosepalus curviflorus* match those of *P. acaciae* in most features. A striking dissimilarity is the coarsely segmented margo of *P. acaciae* (Figure 59G), not found in any other Lorantheae.

**Figure 61.**
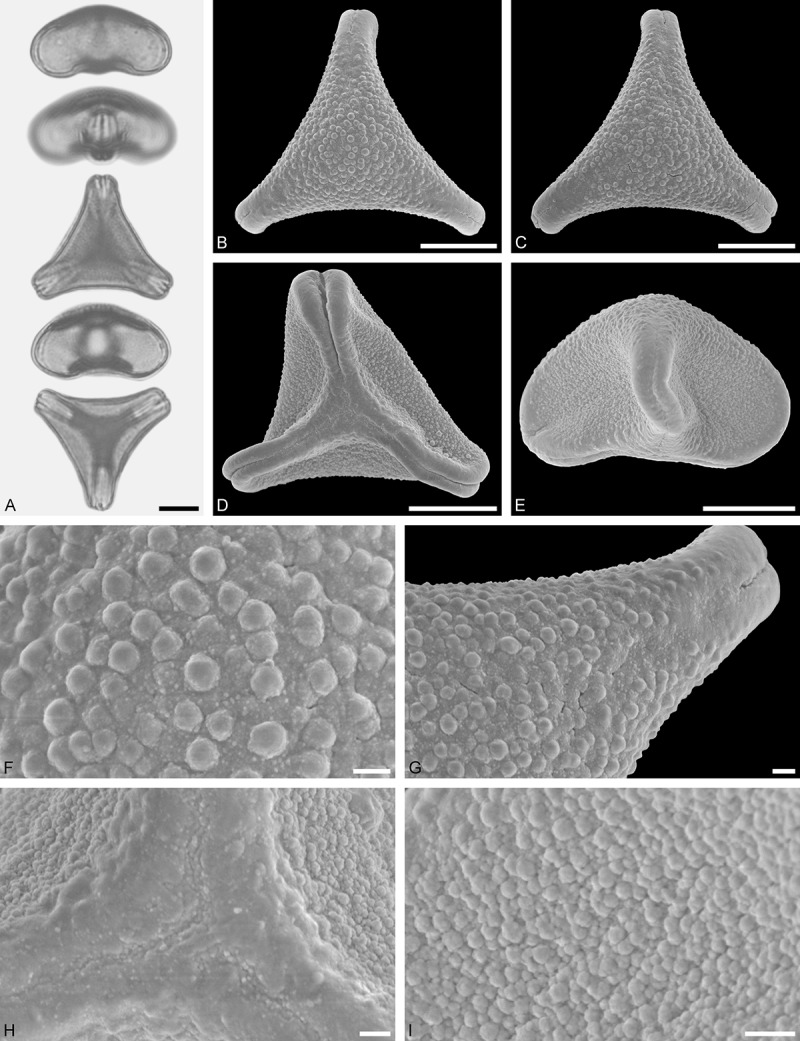
LM (A) and SEM (B–I) micrographs of *Plicosepalus sagittifolius* (WU: from Kenya, coll. M. G. Gilbert, s.n.). **A.** Two pollen grains in equatorial and polar view. **B, C.** Pollen grains in polar view, distal side. **D.** Pollen grain in polar view, proximal side. **E.** Pollen grain in equatorial view. **F.** Close-up of central polar area, distal side. **G.** Close-up of apex, distal side. **H.** Close-up of central polar area, proximal side. **I.** Close-up of mesocolpium. Scale bars – 10 µm (A–E), 1 µm (F–I).

*Plicosepalus sagittifolius* (Engl.) Danser (Figure 61)

#### Description

Pollen, heteropolar, oblate, concave-triangular in polar view, bean-shaped in equatorial view, equatorial apices obcordate; size medium, polar axis 16.7–20.0 µm long in LM, equatorial diameter 28.3–31.7 µm in LM, 23.8–27.3 µm in SEM; syn-(3)colpate on proximal side, zono-(3)colpate on distal side, colpi very short on distal side; exine 1.1–1.8 µm thick, nexine thinner than sexine, nexine markedly thickened in polar areas (LM); tectate; sculpturing psilate in LM, sculpturing elements in area of mesocolpium varying in size on proximal vs. distal side of grains under SEM, distal side verrucate and granulate, verrucae on distal side 0.5–1.2 µm in diameter, proximal side nano-verrucate and granulate, verrucae on proximal side 0.1–0.4 µm in diameter, verrucae decreasing in density from proximal to distal polar area, verrucae composed of conglomerate granula; margo well developed, margo psilate (SEM); colpus membrane nano-verrucate to granulate (SEM).

#### Remark

Pollen Type B. In constrast to the other two species (*Plicosepalus acaciae*, *P. curviflorus*), grains of *P. sagittifolius* are heteropolar. The only other Lorantheae so far with heteropolar grains is *Loranthus*, which has heteropolar and isopolar grains. A further characteristic feature of *P. sagittifolius* is the gradual change seen in the size and density of sculptural elements on the proximal vs. distal side (Figure 61F, I).

**Figure 62.**
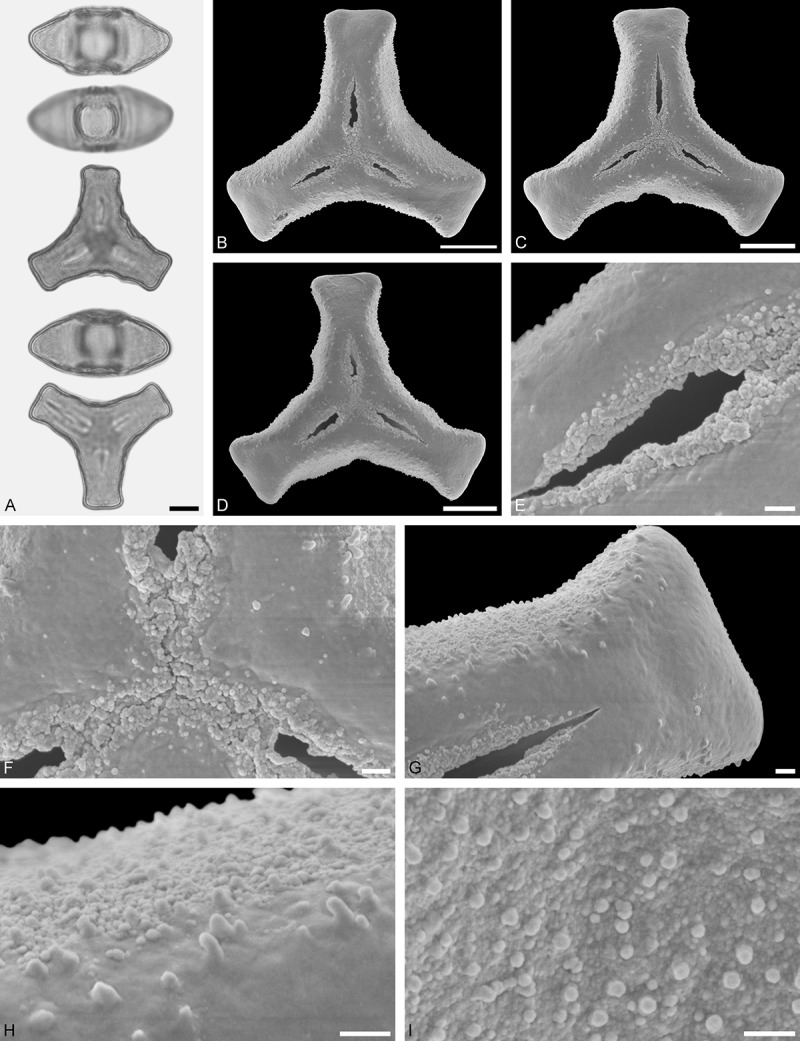
LM (A) and SEM (B–I) micrographs of *Tapinanthus bangwensis* (WU: from Liberia, collector unknown, WU s.n.). **A.** Two pollen grains in equatorial and polar view. **B–D.** Pollen grains in polar view. **E.** Close-up of colpus and membrane. **F.** Close-up of central polar area. **G.** Close-up of apex. **H, I.** Close-ups of mesocolpium. Scale bars – 10 µm (A–D), 1 µm (E–I).

*Tapinanthus bangwensis* (Engl. et K.Krause) Danser (Figure 62)

#### Description

Pollen, oblate, trilobate in polar view, elliptic to (sub)rhombic in equatorial view, equatorial apices T-shaped; size medium, polar axis 20.0–25.0 µm long in LM, equatorial diameter 35.0–40.0 µm in LM, 32.7–35.5 µm in SEM; demisyn-(3)colpate, colpi very short, tapering towards pole and equator; exine 1.2–1.5 µm thick, nexine thinner than sexine, nexine hexagonally thickening in polar area (LM); tectate; sculpturing psilate in LM, nano-/micro-echinate to nano-/micro-baculate and granulate in area of mesocolpium in SEM, echini/bacula 0.3–0.8 µm long, 0.3–0.5 µm wide at base; margo well developed, very broad in equatorial area, margo psilate with very few nano-/micro-echini or nano-/micro-bacula, margo with triangular protrusion in central polar area (SEM); colpus membrane nano-verrucate and granulate (SEM).

#### Remark

Pollen Type B. This pollen shows a unique feature not seen in any other Loranthaceae so far. The margo is extremely broad and well developed, covering most of the polar surface, and is distinctly flattened, not inflated/roundish or indistinct as in other Loranthaceae. It is also the only Lorantheae pollen with demisyncolpate apertures.

*Tapinanthus ogowensis* (Engl.) Danser

(Figure 63)

**Figure 63.**
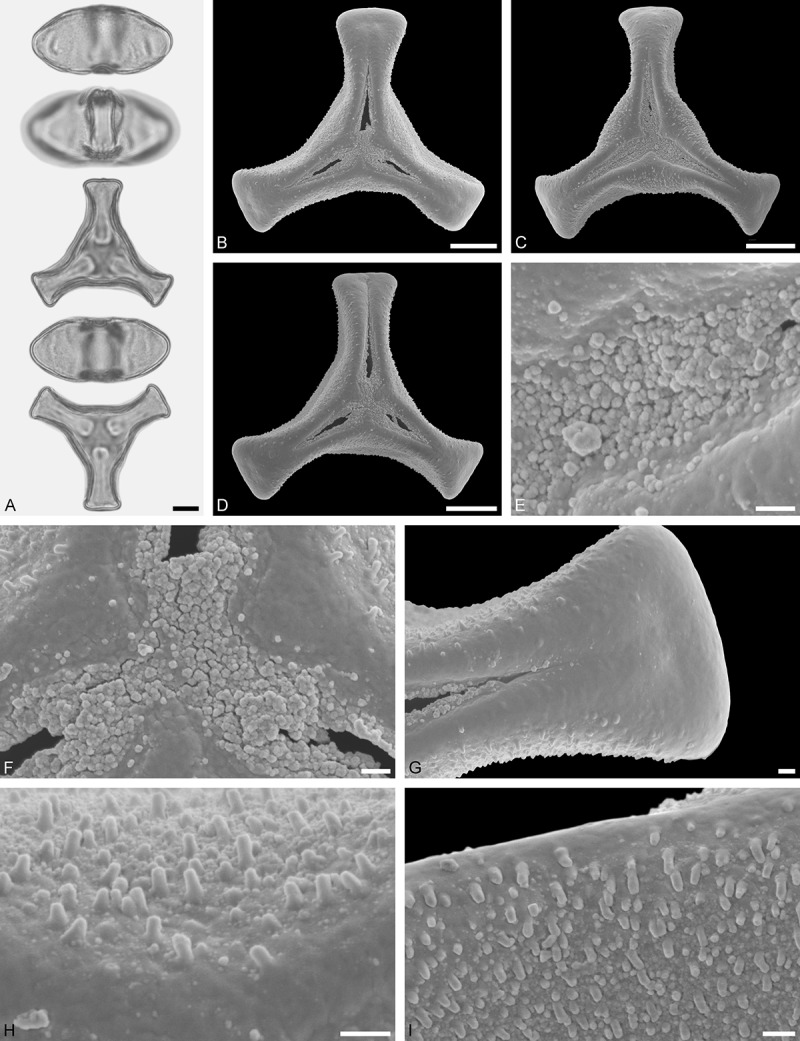
LM (A) and SEM (B–I) micrographs of *Tapinanthus ogowensis* (WU: from Cameroun, collector unknown, det. S. Balle, s.n.). **A.** Two pollen grains in equatorial and polar view. **B–D.** Pollen grains in polar view. **E.** Close-up of colpus and membrane. **F.** Close-up of central polar area. **G.** Close-up of apex. **H, I.** Close-ups of mesocolpium. Scale bars – 10 µm (A–D), 1 µm (E–I).

#### Description

Pollen, oblate, trilobate in polar view, elliptic to (sub)rhombic in equatorial view, equatorial apices T-shaped; size medium, polar axis 23.3–26.7 µm long in LM, equatorial diameter 40.0–45.0 µm in LM, 34.3–42.1 µm in SEM; demisyn-(3)colpate, colpi very short, tapering towards pole and equator; exine 1.3–1.6 µm thick, nexine thinner than sexine, nexine hexagonally thickening in polar area, sexine thickened in area of mesocolpium (LM); tectate; sculpturing psilate in LM, nano-/micro-echinate to nano-/micro-baculate and granulate in area of mesocolpium in SEM, echini/bacula 0.3–1.0 µm long, 0.2–0.3 µm wide at base; margo well developed, very broad in equatorial area, margo psilate with very few nano-/micro-echini or nano-/micro-bacula, margo with triangular protrusion in central polar area (SEM); colpus membrane nano-verrucate and granulate (SEM).

#### Remark

Pollen Type B. Pollen of *Tapinanthus ogowensis* is virtually identical to pollen of *T. bangwensis* except that the apices are slightly narrower.

## Discussion

### Pollen apertures of Loranthaceae – definition and clarification

Feuer and Kuijt (1979, 1980, 1985) used more than a dozen terms to describe aperture organisation in Loranthaceae (other authors used again additional terms). In most grains we studied using high-resolution SEM imaging, the colpi transverse the equatorial area at the tips of the lobes/apices of the triangular pollen types. The minuteness of colpi in the equatorial area of small, compact grains such as found in *Passovia* (*s.str*., species with B-type pollen), *Cladocolea, Struthanthus* and *Peristethium* may obscure their existence and explains why earlier researchers have described a great variety of aperture types, often within the same genus or even species. Singly, the grains of Type C are prominently demicolpate (but never diploporate), and this type is (currently) restricted to two species of *Passovia* and to *Dendropemon*, the sister of *Oryctanthus. Oryctanthus* is the only genus with Type D pollen and minute demicolpi; the three genera are grouped in the same, high-supported clade (Vidal-Russell & Nickrent 2008b; Su et al. 2015; this study). As already observed by Feuer and Kuijt (1985), the minute apertures of *Oryctanthus* are placed on the ridges (lophae) between the polar depressions (lacunae; Figure 25). Feuer and Kuijt (1985, p. 196) classified this pollen type as ‘compound diploaperturate’ assuming that the apertures are not connected via the pollen equator. This seems, however, not to be the case, and hence, the *Oryctanthus* pollen can be considered as highly ornamented version of the tricolpate pollen found in *Dendropemon* and some species of *Passovia*. We also see no evidence for colporate or porate pollen in Loranthaceae in contrast to what is stated in Feuer and Kuijt (1985) who classified about 15 species of the Psittacanthinae genera *Cladocolea, Passovia, Phthirusa* and *Struthanthus* (including one species moved to *Peristethium*) as ‘syncolporate’ and ‘diplosyndemi-colporate/-colporoidate’.

### Taxonomic value of pollen for the identification of Loranthaceae at the genus level

The circumscription of species and genera in Loranthaceae has undergone many changes in the past and is still in flow. Of the more than 190 species figured in the works of Feuer and Kuijt (1978, 1979, 1980, 1985), Muller et al. (1989), Liu and Qiu (1993), Han et al. (2004), Roldán and Kuijt (2005), Caires (2012) and Caires et al. (2012, 2014), 24 are currently treated as synonyms of other species of the same genus (File S4; Tropicos.org 2016), and an additional 24 species have been moved to a different genus (see also Table XI, including further examples not covered in earlier studies). In three cases, the current view (Tropicos.org 2016) is rejected by pollen morphology and either confirms more traditional views or calls for more detailed investigation of the respective taxon. Caires (2012) figured three pollen of the Brazilian genus *Oryctina* including *O. scabrida* (Eichler) Tiegh. According to Tropicos.org (2016) and references provided there, *O. scabrida* is a member of genus *Oryctanthus*, which has long been considered a close relative of *Oryctina*. All nine *Oryctanthus* species studied have pollen of Type D, readily distinguishable from the common Lorantheae pollen Type B, the latter also found in all *Oryctina* studied so far; *Oryctanthus scabrida* has no Type D pollen, and hence, its systematic placement in *Oryctina* (Caires 2012) makes fully sense. No sequence data of *Oryctina* are openly available (File S2) but according to the cladograms shown in Caires (2012), the genus is also genetically distinct from *Oryctanthus* and *O. scabrida* falls within the *Oryctina* subtree. *Taxillus vestitus* (Wall.) Danser (figured by Han et al. 2004) is a synonym of *Loranthus vestitus* Wall. (Qiu & Gilbert 2003; Tropicos.org 2016). Like all members of the Lorantheae, these two genera have pollen of Type B. Nevertheless, pollen of *Loranthus* (Loranthinae; clade G) are distinct in several aspects from the common pollen types in the Lorantheae core clade, which includes *Taxillus* (clade I + J; Figures 2, 3, 7). Based on the figured pollen, *T. vestitus* is better kept in *Taxillus* rather than being moved to *Loranthus. Phthirusa lepidobotrys* figured by Feuer and Kuijt (1985, figures 1, 52, 53); has been placed in synonymy of *Phthirusa pyrifolia* (Kunth) Eichler, a species now included in the revived *Passovia* (Kuijt 2011). In contrast to *Phthirusa* (*Passovia*) *lepidobotrys, Passovia pyrifolia* differs from other *Passovia* species by having pollen of Type C (Figure 28; Feuer & Kuijt 1985), which otherwise are only found in *Dendropemon*, a sister of *Passovia* (Figures 2, 6; Wilson & Calvin 2006; Vidal-Russell & Nickrent 2008b; Su et al. 2015). The phylogenetic position of the revived *Passovia* (Kuijt 2011) is uncertain, since the only species sequenced so far is *Passovia pyrifolia* with the Type C pollen (Wilson & Calvin 2006; Vidal-Russell & Nickrent 2008b; File S2). In light of the overall diversity of Loranthaceae pollen, which is typically conserved at the genus level as far as studied, one may argue against the inclusion of *Phthirusa lepidobotrys* in *Passovia pyrifolia*, and question the inclusion of the latter in the same genus than species with pollen of Type B. Analogously, it is very unlikely that a species producing an Type A pollen (*Phthirusa hutchisonii*), should be congeneric with species producing Type B pollen (*Phthirusa clandestina*, this study; *Phthirusa inconspicua* [Benth.] Eichler; Feuer & Kuijt 1985). Also for this case, the currently available molecular data is unsatisfying: the pollen of the only sequenced *Phthirusa* (*s.str*.) species, *Phthirusa inorna* is unknown. *Phthirusa inorna* is a species that is genetically very distinct from all other Psittacanthinae (Figure 2; Wilson & Calvin 2006) and used to be part of the dissolved genus *Ixocactus*, which included *Phthirusa hutchisonii* (Kuijt 2011).

**Table XI.** Species that have been moved from one genus to another, with notes on the systematic affinities of their pollen.

Currently accepted name	Name in original publication	Reference for pollen	Pollen would suggest
*Aetanthus dichotomous, A. mutisii*	*Psittacanthus nodosus, P. holtoni[i]*	Feuer and Kuijt (1979), only TEM figured	*Psittacanthus*^b^
*Agelanthus discolor*	*Tapinanthus discolor*	This study	*Oncocalyx*, a close relative of *Agelanthus*
*Cladocolea micrantha*	*Phthirusa micrantha*	Feuer and Kuijt (1985, figure 54)	*Cladocolea/Struthanthus*
*Loranthus vestitus*	*Taxillus vestitus*	Han et al. (2004, figure 79)	Scurrulinae (*Taxillus*)
*Oryctanthus scabridus*	*Oryctina scabrida*	Caires (2012, figures 1–16C)	*Oryctina*
*Panamanthus panamensis*	*Struthanthus panamensis*	Feuer and Kuijt (1985, figures 19, 79, 90–92)	Leaning to *Cladocolea*
*Passovia podoptera*^a^	*Struthanthus (Phthirusa) pterygopus (-a)*	Feuer and Kuijt (1985), not figured, but described as ‘striato-rugulate’	*Struthanthus*
*Passovia pyrifolia*	*Phthirusa pyrifolia*	This study	Leaning to *Dendropemon*
*Passovia ovata, P. pedunculata [P. stelis]*	*Phthirusa ovata, P. retroflexa*	This study	Fits with other *Passovia* types
*Peristethium leptostachyum*	*Struthanthus ~us*	Feuer and Kuijt (1985, figure 13); this study	Unique character suite
*Phthirusa inconspicua*	*Cladocolea inconspicua*	Feuer and Kuijt (1985, figure 5)	*Passovia/Phthirusa*
*Phthirusa clandestina*	*Ixocactus clandestina*	This study	*Passovia/Phthirusa*
*Phthirusa hutchisonii*	*Ixocactus hutchisonii*	Feuer and Kuijt (1985, figures 98–104; this study)	Related to Tupeia

Note: Currently accepted name follows Tropicos.org (2016) and references given there; ^a^ Valid name according Kuijt (2011); ^b^ According to the text describing the pollen characterised as ‘Type Ia’.

In other cases, pollen features have anticipated later taxonomic revision (Table XI). Feuer and Kuijt (1985) noted that the pollen features of *Struthanthus panamensis* bring this somewhat (morphologically) isolated species closer to *Cladocolea* than any other species of *Struthanthus*. Because of its generally particular morphology, Kuijt (1991) moved the species to its own genus, *Panamanthus*. A similar case is *S. leptostachyus* (Feuer & Kuijt 1985; this study). The pollen shows a unique character suite not found in any other pollen of the genus, and is the only one in the group of small-flowered species with striae. The species has recently been moved to *Peristethium* (Tropicos.org 2016; anonymous reviewer, personal communication). Another case within the Psittacanthinae where pollen conflicts with generic association is the recently described third, red-flowered species of *Tripodanthus* (the other two species are white flowered): *Tripodanthus belmirensis* (Roldán & Kuijt 2005, figure 2). The figured pollen lacks all diagnostic features seen in the other two species of the genus (Figure 34; Feuer & Kuijt 1980). From its form and sculpturing, it would better fit within the *Struthanthus* lineage. Roldán and Kuijt (2005, p. 207) note that the sculpturing of the mesocolpium is ‘irregularly depressed rugulate-verrucate’, which is in stark contrast to the situation in the two white-flowered species, but fit with part of *Struthanthus*. The authors highlight further morphological affinities to *Tristerix* (a Ligarinae of uncertain phylogenetic position; Figures 2, 3). Molecular data (Amico et al. 2012) confirm that the species is related to the other two *Tripodanthus* species, but also recognise it as genetically distinct. Amico et al. (2012) did opt for not including any other Psittacanthinae, which would have allowed testing the monophyly of the three *Tripodanthus* species. Instead, they relied on several more or less distant outgroups (see Figure 2; Wilson & Calvin 2006; Su et al. 2015) to root their tree. Outgroup–ingroup long-branch attraction would explain why the critical branch, the one that sorts the three species and nests *Tripodanthus belmirensis* in *Tripodanthus*, receives unambiguous support from parsimony bootstrapping (BSp = 99), but low support from maximum likelihood bootstrapping and Bayesian-inferred posterior probabilities (BS_ML_ = 57 and PP = 0.55). Hence, the third *Tripodanthus* species could well represent a taxon intermediate between the white-flowered *Tripodanthus* species and the more derived species of the *Strutanthus-Cladocolea* lineage (in analogy to *Panamanthus*). Such a treatment would however be in too strong contrast with the gross-morphology of the species (anonymous reviewer, personal communication, 2016). Furthermore, the next diverging lineage is *Psittacanthus* and not *Cladocolea-Struthanthus* (e.g. Figure 2). So the unique pollen of *Tripodanthus belmirensis* more likely represents a convergent (or parallel) development towards more compact pollen in this early diverging genus of the Psittacanthinae.

**Figure 64.**
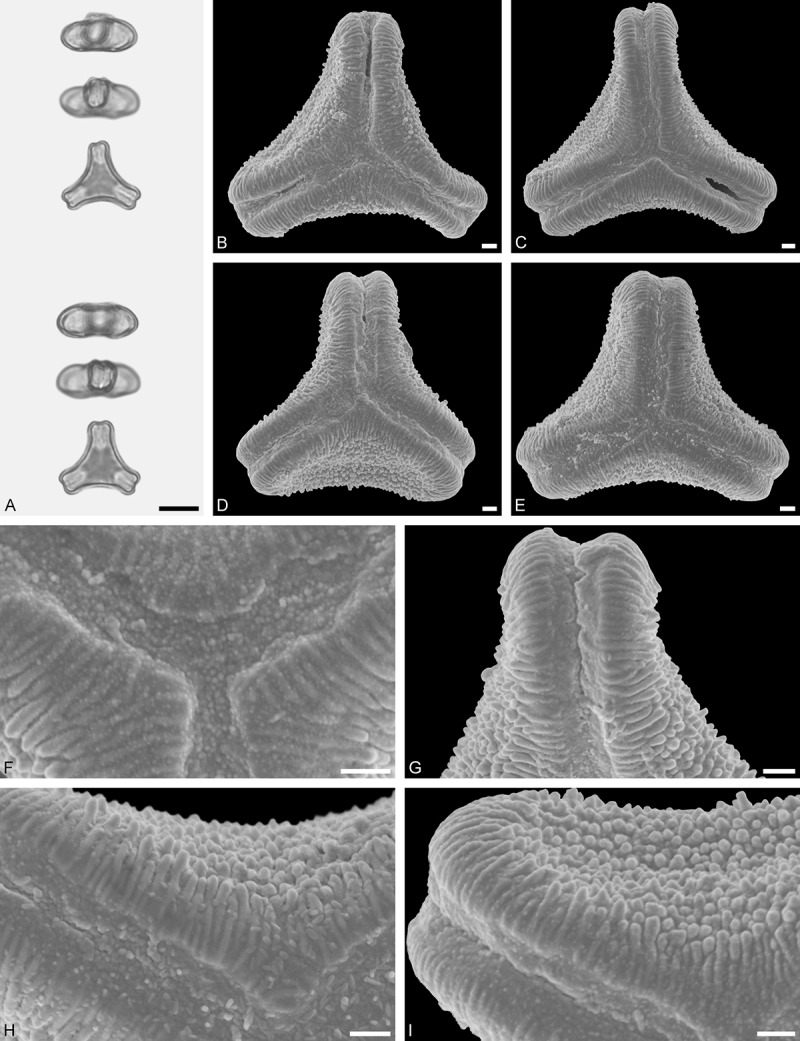
LM (A) and SEM (B–I) micrographs of pollen grains from an isotype of ‘*Struthanthus’ mapirensis* Rusby (WU 029706), a likely *Gaiadendron*. **A.** Two pollen grains in equatorial and polar view. **B–E.** Pollen grains in polar view. **F.** Close-up of central polar area. **G.** Close-up of apex. **H, I.** Close-ups of margo and mesocolpium. Scale bars – 10 µm (A), 1 µm (B–I).

Figure 64 shows the pollen of an isotype of a species from Bolivia originally described by Rusby (1896) as *Struthanthus mapirensis*, still treated as a valid taxon (Tropicos.org 2016). The plant on the voucher is conspicuously large-flowered, hence, surely not a member of *Struthanthus*. The pollen is indistinguishable from those of the root-parasitic, large-flowered *Gaiadendron* (compare Figures 11 and 64), which used to be monotypic (a second species has recently been described by Kuijt & Graham 2015 from central Peru). We also failed to find other substantial differences between the isotype and vouchers of *Gaiadendron*, and hence, conclude that *Struthanthus mapirensis* is to be treated as yet another synonym of *Gaiadendron punctatum*. A check-up in the JSTOR Global Plants Database (https://plants.jstor.org/) revealed that at least one of the isotypes of *Struthanthus mapirensis* has, indeed, been relabelled to *Gaiadendron mapirensis* but without providing any authority. Although the holotypes and isotypes of *Struthanthus mapirensis* found in several herbaria are probably not more than collection curiosities (we were unable to find any further, more recent publication listing or referring to this taxon), this example proves further the utility of palynology in the identification of taxonomic issues in the Loranthaceae, such as the association of species to certain genera.

### Systematic value of pollen types in Loranthaceae at higher hierarchical levels – preliminary correlation of pollen evolution and phylogeny

The phylogenetic relationships inferred from the currently available molecular data, even though being ambiguous in many aspects (Figures 2, 3; File S1), are in agreement with some of the hypotheses of Feuer and Kuijt (1980, 1985) about pollen evolution in Loranthaceae. The syncolpate organisation (Type B) is indeed the basic feature of Loranthaceae pollen except for the Type A pollen taxa *Tupeia antarctica* and *Phthirusa hutchisonii*. All root-parasitic genera show Type B pollen, and *Atkinsonia* is no exeption (cf. Feuer & Kuijt 1980). The origin of the unique, aberrant Type A shared by the monotypic *Tupeia* from New Zealand and an enigmatic species of South America (*P. hutchisonii*) remains obscure. Feuer and Kuijt (1985) noted that origin of the 4–5-colpate *P. hutchisonii* pollen is unknown, but represents an independent development within the Loranthaceae. However, the pollen of *Tupeia* with its similarity not only to *P. hutchisonii* but also to pollen of the former Eremolepidaceae (Feuer & Kuijt 1978; now included in Santalaceae) represents a very unlikely convergent evolution within the Loranthaceae, and the same holds for the unique Type B, C and D (see next section). Interestingly, *Tupeia* was not included in the framework established by Feuer and Kuijt (1985), although they note in the earlier work that *Tupeia* combines characters of both Eremolepidaceae and Loranthaceae.

The comparison of pollen morphologies with the molecular-based tree confirms several trends Feuer and Kuijt (1980, 1985) intuitively based on their knowledge of the group, even though their basic assumption that the small- and large-flowered neotropical taxa form respective natural groups turned out to be wrong. The essentially trilobed (deeply concave-triangular) pollen grains with striate ornamentation are limited to putatively ‘basal’ taxa within the Loranthaceae (Figure 4). Within the New World clade, a clear trend is seen towards less deeply lobed to convex-triangular, compact, but essentially still syncolpate (to parasyncolpate) grains ([*Aetanthus*-]*Psittacanthus-Cladocolea-Struthanthus*[-*Peristethium*] lineage) and demicolpate-lobed and -circular grains (*Passovia-Dendropemon-Oryctanthus* lineage). Pollen grains of these two lineages show more and more psilate surfaces, whereas the early diverging *Tripodanthus* evolved in a different direction (Figures 6, 22–34). Pollen grains as still found in *Notanthera*, one candidate for a sister of the Psittacanthinae (Figure 4; Feuer & Kuijt 1980; Su et al. 2015), may really represent a still primitive form of the Psittacanthinae pollen. The unique pollen type of *Phthirusa hutchisonii* indicate that at least this former *Ixocactus* species, is not closely related to the other Psittacanthinae, but represents an isolated, early diverged lineage such as *Tupeia*.

Molecular data fails to unambiguously resolve the position of the other two tribes within the Loranthaceae (File S1; Wilson & Calvin 2006; Vidal-Russell & Nickrent 2008b; Su et al. 2015; this study). One important observation is that the Elytrantheae, which show in general relatively limited overall molecular divergence and are, hence, poorly supported as a clade here and in the tree of Su et al. (2015), have also similar pollen (Figure 5; Table IV). The Elytrantheae are much better supported (BS > 80; PP = 1.0) in earlier studies using matrices without or less-dominated by (more conserved) coding regions (Wilson & Calvin 2006; Vidal-Russell & Nickrent 2008b; File S1). Hence, the pollen provides further evidence for the common origin of this group (Nickrent et al. 2010). Another case is *Aetanthus*, a sure member of the Psittacanthinae with according pollen and *mat* K sequence (non-chimeric part; Su et al. 2015, figure 1B), and erroneously resolved in our tree as sister to *Desmaria* (see also Su et al. 2015, figure S7). This is a missing data artefact: the included 18S data are uninformative and the only available *mat*K accession is partly problematic (artificial chimera) and thus not included in our data set (see File S2).

The monophyly of the Lorantheae is a commonly accepted fact, and finds its representation in the molecular data, which produces a pronounced, unambiguously supported root for this tribe, despite notable genetic divergence within the different subtrees (Figure 2; Wilson & Calvin 2006; Su et al. 2015). This is reflected by pollen morphology. Even though individual variation exists, most Lorantheae studied so far show the same basic pollen types (variants of Type B). One can observe a variation in outline and size of ornamental elements (Figures 7, 8, 35–63; Tables VIII–X; see also the relatively low quality images in Liu & Qiu 1993 and Han et al. 2004), but this does not match the diversity seen in the Psittacantheae (Figures 6, 22–34; Tables V–VII; Feuer & Kuijt 1980, 1985). Pollen features are generally conserved traits that can remain nearly unchanged for 40 or more million years, as e.g. documented for Aponogetaceae (Alismatales; Grímsson et al. 2014), Fagaceae (Fagales; Denk & Grimm 2009; Bouchal et al. 2014; Grímsson et al. 2015), Lythraceae (Grímsson et al. 2011, 2012), and several Santalales lineages (next section). However, this does not explain the disparity between pollen diversity and genetic diversity in the Lorantheae in comparison to the Psittacantheae.

An explanation could be that the modern taxonomic and genetic diversity of the essentially Old World Lorantheae represent a second, more recent phase of radiation and diversification. This radiation and subsequent rapid speciation involved substantial genetic drift, but lineage sorting did not result in the evolution of significantly new pollen morphologies. This is in contrast to the situation in the New World where a high diversity in pollen morphologies goes hand-in-hand with substantial genetic diversity (Figure 2; Su et al. 2015). Nevertheless, we observe a convergent trend to more compact grains with straight to convex-triangular outline in polar view analogous to what is seen in the New World Psittacanthinae.

A notable exception is *Muellerina*, a genus from eastern Australia with pollen strikingly similar to that of the distantly related South American root parasite *Gaiadendron*. However, with regard to the uncertainties about the principal relationships within the Loranthaceae (Figures 2, 3; File S1), a simple explanation could be that the Lorantheae lineage to which *Muellerina* belongs retained an ancestral, underived pollen type from which all other pollen types in the Lorantheae evolved. Vidal-Russell and Nickrent (2008b) found that the Ileostylinae are sister to the remaining Lorantheae, albeit with low support (BS < 60, PP = 0.69); Su et al. (2015) found low to moderate support (BS_ML_ = 60; PP = 0.79) for Loranthinae as the first diverging lineage within the Lorantheae (mainly supported by signal from the 25S data partition; File S6). Using the gene-jackknifing experiment with taxon-reduced matrix, it can be explored that different gene regions prefer different placements, but also that the signal from the concatenated matrix is perfectly ambiguous: all three alternatives have BS ≈ 33 (File S1). Until more discriminative molecular data becomes available (note the very short branch for the Ileostylinae-Loranthinae clade in Figure 2), the pollen could be used as argument to prefer the topology in Vidal-Russell and Nickrent (2008b) over the one seen here (Figure 2) and in Su et al. (2015). That ancestral pollen types are found in isolated species in Australasia and Africa/South America is not entirely unusual. For instance, the oldest known *Aponogeton* pollen shows a type, which today is also only found in two phylogenetically only distant-related Madagascan and south-western Australian species (Grímsson et al. 2014; Chen et al. 2015).

### Systematic relevance of Santalales pollen in context of the latest molecular-phylogenetic framework of the order

*‘Olacaceae s.l.’ grade. –* The pollen of the genera (*Scorodocarpus, Strombosia, Diogoa, Tetrastylidium, Strombosiopsis*) comprising the possibly earliest diverged Santalales lineage, the Strombosiaceae (Su et al. 2015, figure 1A), are homogeneous in form and sculpturing (Feuer 1977; File S7). They are all isopolar, small to medium in size, suboblate to subprolate, dipyramidal-spheroid in form with a straight- to convex-triangular outline in polar view, and an elliptic to circular outline in equatorial view. The grains are usually 3-colpate, rarely 3-colporoidate, and with SEM sculpturing ranging from psilate-perforate to reticulate. The pollen grains of Erythropalaceae (*Erythropalum, Heisteria*) are generally similar to those of Strombosiaceae. The grains of some *Heisteria* species can be heteropolar regarding sculpturing and apertures showing more variable sculpturing in SEM (can be verrucate or rugulate) and including grains that are syncolpate on one polar face (Feuer 1977).

The pollen of *Octoknema* (monotypic Octoknemaceae) is of the same basic form as in Strombosiaceae and Erythropalaceae, but shows more 3-colporoidate grains with rugulate SEM sculpturing; a feature also observed in members of the latter family (File S7). *Ximenia* (Ximeniaceae) pollen shows the same general morphological features than the reticulate pollen seen in most Strombosiaceae and Erythropalaceae (Maguire et al. 1974; Feuer 1977). That the same basic pollen type is shared by various early diverging lineages of the Santalales (formerly included in the ‘Olacaceae *s.l*.’ grade; Feuer 1977; APG 2003) indicates that the 3-colpate, triangular bipyramidal-spheroidal pollen grains with psilate-perforate to reticulate sculpturing represent the most primitive pollen type of the Santalales. This primitive type has been conserved in these early diverging lineages (primary divergences have been dated to *c*. 100 ± 20 Ma; see references provided by Stevens 2001 onwards), and only modified to some degree in some species of *Heisteria*.

The Aptandraceae (*Ongokea, Harmandia, Chaunochiton, Anacolosa, Phanerodiscus, Cathedra*) is the first family of the ‘Olacaceae *s.l*.’ grade showing a clear differentiation in pollen morphology from the primitive type. All pollen grains are still small to medium in size, but a trend to (distinctly) oblate grains is seen (P/E ratios, ratio of polar to equatorial axis, range between 0.36 and 0.84; File S7). The pollen grains of *Chaunochiton*, the earliest diverging genus in the *Aptandra* clade of the Aptandraceae, are still 3-colpate and have the ancestral form (triangular bipyramidal-spheroidal), but show exine thinnings in the mesocolpial (3×) and polar (2×) areas accompanied by exine thickenings along the colpi encircling the polar thinnings. This gives the pollen a unique coarsely ridged appearance not observed in any other genus of the Santalales (Feuer 1977, figures 28–37). The pollen of *Aptandra, Ongokea* and *Harmandia* (also *Aptandra* clade) is very alike and appears to be more derived: the grains are clearly oblate, predominantly 4-aperturate (?poroid) and quadrangular in polar view, and showing micro-reticulate to reticulate sculpturing in SEM. The pollen of *Aptandra* and *Ongokea*, recognised as sister genera with high support, are mostly heteropolar with one polar side more flattened than the other. The predominant quadrangular outline of these pollen grains in polar view is very rare within Santalales (Maguire et al. 1974; Feuer 1977; File S7). The pollen of *Anacolosa, Phanerodiscus* and *Cathedra* of the second Aptandraceae clade, the *Anacolosa* clade, is clearly oblate and predominantly di-3-porate, with a set of three pori on each polar face of the pollen. This clade-characteristic pollen type can be traced until the latest Cretaceous (Maastrichtian) of Germany (Malécot & Lobreau-Callen 2005). The outline in polar view ranges from concave- to straight-triangular as in the pollen of the earlier diverging lineages. Pollen grains of *Cathedra*, resolved as sister to the other two genera (Su et al. 2015, figure 1A), are convex-triangular as in the primitive Santalales pollen type, whereas those of *Phanerodiscus* are concave-triangular (almost lobate) in polar view; a feature also found in pollen of Schoepfiaceae and Loranthaceae Type B (pro parte) and C (File S7). Pollen grains of its sister *Anacolosa* are straight- to convex-triangular to triangular, being intermediate between *Phanerodiscus* and *Cathedra*. Pollen of these three genera have similar non-conspicuous SEM sculpturing (psilate, perforate, granulate, rugulate; Maguire et al. 1974; Feuer 1977; Malécot & Lobreau-Callen 2005).

The general pollen form, outline and sculpturing of Coulaceae (*Coula, Minquartia, Ochanostachys*) is again very similar to that of the early diverging lineages (Strombosiaceae to Ximeniceae), but with the 3-colporoidate apertures as in some Erythropalaceae and *Ximenia*. Unique to this group are the large verrucae along the colpi and in the polar regions that can be observed in some species (Feuer 1977). Most Olacaceae are 3-porate and the shape of the grains can be very oblate (e.g. *Olax*); in addition, di-3-porate grains – an aperture organisation otherwise only found in members of the *Anacolosa* clade of Aptandraceae – can be found in *Ptychopetalum* and *Olax linderi* Hutch. et Dalz. (File S7). The reticulate SEM sculpturing of *Ptychopetalum* and *Olax linderi*, and the inconspicuous psilate to perforate SEM sculpturing of *Dulacia* and the remaining Olax species, is comparable to that observed in the other lineages of the ‘Olacaceae *s.l*.’ grade (Feuer 1977, 1978; Malécot & Lobreau-Callen 2005).

*Santalales core clade: Balanophoraceae s.str. –* Su et al. (2015) found high support for a clade, henceforth called ‘Santalales core clade’, including several families formerly part of the Santalaceae as well as both subclades of the Balanophoraceae *s.l*. (‘Balanophoraceae A’ = Balanophoraceae *s.str*., ‘Balanophoraceae B’ = Mystropetalaceae), Loranthaceae, Misodendraceae, Schoepfiaceae and Viscaceae. Paralleling the higher genetic divergence observed in this clade compared to the ‘Olacaceae *s.l*.’ grade, lineages of the Santalales core clade differ by their pollen grains. Pollen of extremely long-branching Balanophoraceae (*s.str*.), the first diverging lineage within the Santalales core clade, is quite variable. The sister genera *Corynaea* and *Helosis* still have the 3-colpate aperture arrangement as observed in the families of the ‘Olacaceae *s.l*.’ grade. The pollen of *Lathrophytum* (not included in Su et al. 2015’s tree), and *Ombrophytum *+ *Lophophytum* (sister clade of *Corynaea *+ *Heliosus*) are distinctly 3-(6-)colporate, an aperture arrangement that becomes predominant in the core Santalales.

In contrast, *Langsdorfia* (4–5-porate) and *Scybalium* (6–8-porate; spheroid grains) are multi-porate/pantoporate. The latter two genera are not included in Su et al. (2015)’s tree, which is unfortunate given the uniqueness of their pollen compared to other Balanophoraceae; spheroid, pantoporate grains are otherwise only found in *Misodendrum* (Misodendraceae), one of the sistergroups of the Loranthaceae. *Langsdorffia* is one of the very few Santalales genera (including *Aptandra, Ongokea, Harmandia*; all members of the same Aptandraceae subclade) producing pollen grains that are quadrangular in polar view (Hansen 1980; González et al. 2013). The basic SEM sculpturing type in Balanophoraceae is weakly to strongly rugulate (*Corynaea, Helosis, Lophophytum, Ombrophytum, Lathrophytum*) and also verrucate (e.g. *Ombrophytum*). An exception is again *Langsdorffia* pollen with its echinate SEM sculpturing, which is also seen in the pantoporate pollen of *Misodendrum*.

#### Santalales core clade: Loranthaceae and sister groups

The pollen of *Misodendrum* (monogeneric Misodendraceae) equals in form, size and aperture organisation that of the Balanophoraceae *Scybalium*, but taking the number of pori to an extreme (up to19-porate). The pollen of *Misodendrum* is also echinate in SEM, a sculpturing type that seems to have evolved several times (*Langsdorffia* [Balanophoraceae], Misodendraceae, Loranthaceae pollen Type A, Opiliaceae, Amphorogynaceae, Santalaceae, Viscaceae) following the divergence of core Santalales. The sister clade of *Misodendrum*, the Schoepfiaceae (*Quinchamalium, Arjona, Schoepfia*) is characterised by a very different, heteropolar pollen with syn-3-colpate (+modified) aperture arrangements and often convex-triangular outline in polar view as found in grains of the main Loranthaceae pollen Type B (Swamy 1949; Erdtman 1952; Feuer 1977; Halbritter 2016). It differs from the Loranthaceae pollen Type B by being triangular dipyramid-spheroid (*Quinchamalium*, first diverging lineage within the Schoepfiaceae), i.e. showing the primitive pollen form of the Santalales, triangular dipyramid (*Arjona*), or distinctly tetrahedral (*Schoepfia*, sister of *Arjona*), in form. The tetrahedral grains of *Schoepfia* are syn-3-colpate over the conical polar face with a zonasulculus running around the equator connecting the equatorial apices (Feuer 1977; Halbritter 2016). The pollen of *Arjona* shows a similar construction (Erdtman 1952; Feuer 1977), but with three short colpal branches running from the zonasulculus onto the non-syncolpate face that are positioned intermediate between the equatorial apices. The two genera are the only Santalales with zonasulculi. *Quinchamalium* pollen is syn-3-colpate, the apertures can be short and trilete in outline and confined to a single polar face or they can stretch over the equator onto the distal face of the pollen (Swamy 1949; Erdtman 1952; Feuer 1977) approaching the aperture organisation in putatively derived Loranthaceae pollen of Type B (e.g. *Loranthus* and *Plicosepalus* [Lorantheae]).

Interestingly, the pollen of the three genetically very distinct Mystropetalaceae genera (*Dactylanthus, Hachettea, Mystropetalon*) are not only different to each other but differ also markedly from the basic pollen types of their sister family Loranthaceae, as well as their next-closest relatives Schoepfiaceae + Misodendraceae. Pollen of *Dactylanthus* is spheroid in form and 3–13-pantoporate, hence, overall similar to pollen of *Misodendrum* and the genetically not studied *Scybalium* (Balanophoraceae), but those have different SEM sculpturing (echinate in *Misodendrum*; granulate in *Scybalium*). The 2–4-porate pollen of its sister genus *Hachettea* is poorly documented, and it is uncertain if its shape, form, outline, or sculpturing corresponds in any way to that of *Dactylanthus* or the also occasionally 4-porate pollen of the genetically not studied *Langsdorffia* or the Type A pollen of Loranthaceae. The 3–5-colpate pollen of *Mystropetalon* is unique within Santalales, being a triangular prism, cube or a pentagonal prism in form, and with a rectangular outline in equatorial view (Erdtman 1952; Macphail & Mildenhall 1980).

#### Santalales core clade: subclade including the remaining families

The remaining families of the Santalales comprise the sister clade to the clade including the Loranthaceae. The pollen of Opiliaceae (*Agonandra, Anthobolus*), the first diverging lineage within this clade, is mostly spheroid in form. *Agonandra* pollen is 3-colporate (long colpi) with an echinate SEM sculpturing, hence is similar to the Type A pollen of the Loranthaceae (*Tupeia, Phthirusa hutchisonii*; colpate, short colpi), but *Anthobolus* pollen can also be 3-poroidate or 3-porate with a reticulate basal sculpturing and an echinate suprasculpture. This combination is currently not known from any other genus of Santalales; it may represent a combination of the basic sculpturing of the primitive Santalales pollen (reticulate) of a more derived sculpturing (tectate[?], echinate, as seen e.g. in *Antholobus, Misodendron* and Loranthaceae pollen Type A).

The relationship of the next three lineages to each other is not resolved in the study of Su et al. (2015). The pollen of Comandraceae is 3-colporoidate (*Geocaulon*) or 3-colpate (*Comandra*) and either shows the non-conspicuous psilate/perforate/microrugulate or the reticulate SEM sculpturing typical for the most members of the earlier diverged families of Santalales. The general outline, shape and form of the pollen are also consistent with that observed in the putatively primitive Santalales pollen (Feuer 1977). In Thesiaceae, the pollen of *Buckleya* differs from those of *Osyridicarpos, Thesidium* and *Thesium*; again perfectly mirroring the genetic divergence patterns observed in this family (Su et al. 2015, figure 1C). The pollen of *Buckleya*, genetically most distinct from the remainder of the family and resolved as sister to a relatively long-rooted clade including all other genera, is 3-colporate and mostly showing a striate SEM sculpturing. The pollen of *Osyridicarpos, Thesidium* and *Thesium* are, in contrast, mostly 3-colpate and showing a non-distinct psilate/perforate/foveolate sculpturing. Both pollen of *Osyridicarpos* and *Thesium* can be heteropolar in outline and tetrahedral in form (File S7), hence, approaching in this aspect the pollen of the distantly related Schoepfiaceae, with bifurcating colpi on one polar face. Some *Thesium* species have reticulate pollen that can also have unusually large lumina compared to the size of the pollen (Feuer 1977). The pollen of Cervantesiaceae (*Acanthosyris, Cervantesia, Jodina, Pyrularia, Scleropyrum*) are all very similar, showing the primitive Santalales form and SEM sculpturing.

The last Santalales clade includes four families with partly different pollen and one widespread genus and family with increased pollen variation compared to what can be seen elsewhere in the Santalales with exception of the Loranthaceae. The pollen of *Mida* (Nanodeaceae) is tetrahedral in form and concave-triangular to rectangular in outline in equatorial view, with one polar face flattened and the other conical. The flattened face is syncolpate (vs. syncolpate on the conical polar face in *Schoepfia*) and the colpi stretch over the equator of the pollen on to the conical face where they are bifurcating. The combination of form, outline, SEM sculpturing and aperture arrangement of *Mida* pollen is very distinctive and unique within Santalales, but the SEM sculpturing (perforate, reticulate) is comparable to that observed in many other families of this order (Feuer 1977; File S7).

In Santalaceae (*Antidaphne, Colpoon, Eubrachion, Exocarpos, Ixidium, Lepidoceras, Myoschilos, Nestronia, Omphacomeria, Osyris, Rhoiacarpos, Santalum*; File S7), there is a clear differentiation in pollen morphology and SEM sculpturing between the genera. The pollen of *Omphacomeria, Exocarpos* and *Nestronia*, which form a high supported subclade with a very prominent root branch, are very alike. They are prolate (shape), spheroid (form), with a 3-colporate aperture arrangement and a non-conspicuous psilate/perforate/granulate/microrugulate SEM sculpturing. The pollen of *Osyris, Colpoon* (same subclade) and *Eubrachion* (placed in a sister clade but critical branches with insufficient support) are of similar morphology, mostly spheroidal to prolate in shape, but more triangular in outline in polar view and therefore approaching the primitive Santalales pollen form. They also have a more conspicuous rugulate (seen in many other Santalales) to reticulate (putatively ancestral) SEM sculpturing. Pollen grains of *Myoschilos* (oblate, ±tetrahedral, rhombic-heteropolar; phylogenetically unresolved) and *Santalum* (prolate, spheroid, elliptic; sister to *Antidaphne*) pollen share the same psilate sculpturing around apertures and the polar regions and they both have micro-echinate sculpturing occurring in areas of mesocolpium. The 3-colporate pollen of *Lepidoceras, Ixidium* (not included in Su et al. 2015’s dataset), and *Antidaphne* (sister of *Santalum*) are mostly spheroid in form with an echinate SEM sculpturing, the length and conspicuousness of the colpi varies considerably between the genera and so does the size and frequency of echini (Feuer 1977; Feuer & Kuijt 1978). The Type A pollen of Loranthaceae falls within the morphological range observed in these genera under LM and SEM in all aspects, the Type A pollen grains only differ by the lack of endopori (all Loranthaceae are colpate) and the ultrastructure of the pollen wall as observed in TEM (Feuer & Kuijt 1978).

The Viscaceae (*Viscum, Arceuthobium*) are characterised by ±spheroidal, 3-colporate and echinate pollen. Their basic pollen type is very similar to Loranthaceae pollen Type A, some Santalaceae and Opiliaceae. The pollen of *Arceuthobium* are consistently equipped with the pseudocolpi situated midway between the functional colpi. *Viscum* is a large, widespread genus and its pollen is not only echinate, but shows various different types of SEM sculpturing (including rugulate, verrucate, baculate, clavate), forming the only lineage so far paralleling in this aspect the variation seen in Loranthaceae. Some species also bear pseudocolpi similar to those observed in *Arceuthobium* (Hawksworth & Wiens 1972; Feuer & Kuijt 1982; Feuer et al. 1982; Munro et al. 2014).

With respect to the genetic divergence expressed by members of the Viscaceae (Su et al. 2015, figure 1C), the other genera should be studied palynologically. The pollen of Amphorogynaceae (*Amphorogyne, Choretrum, Daenikera, Dendromyza, Dendrotrophe, Dufrenoya, Leptomeria, Phacellaria, Spirogardnera*) shows consistently the primitive Santalales pollen form. The grains are small, suboblate to subprolate in shape, triangular dipyramid-spheroid in form, and straight- to convex-triangular in polar view. The pollen is mostly 3-colporate as in many members of the Santalales core clade, except for *Choretrum* (p.p.) and *Phacellaria* (3-colpate as in most members of the ‘Olacaceae *s.l*.’ grade), *Dendromyza* (partly 3-porate) and *Leptomeria* (partly syn-3-colporate as in many Type B Loranthaceae and Schoepfiaceae). The pollen of *Daenikera* differs from pollen of the other genera in having a clavate SEM sculpturing, which is otherwise only found in some species of *Viscum* (Viscaceae). The SEM pollen sculpturing of *Amphorogyne, Choretrum, Phacellaria* and *Spirogardnera* is similar to that of the more or most primitive Santalales pollen, showing also the non-conspicuous psilate/perforate/foveolate/microrugulate or perforate to reticulate (e.g. *Choretrum*) SEM sculpturing. The genera *Dufrenoya, Dendrotrophe* and *Dendromyza* all produce two different types of pollen, one that is comparable to pollen of *Amphorogyne* and *Phacellaria*, the other with a clear echinate SEM sculpturing. Interestingly, species of *Leptomeria*, a genus deeply nested in the Amphorogynaceae (Su et al. 2015, figure 1C), produce pollen showing either of the three basic SEM sculpturing types observed in the other genera of this family. One taxon, *Leptomeria drupacea* (Labill.) Druce has fused colpi on one polar face resulting in a syn-3-colporate aperture arrangement (Feuer 1977).

Based on the summary presented here on the pollen morphology of Santalales, the following can be deduced. (i) Relatively small, 3-colpate, triangular dipyramid-spheroid pollen with psilate-perforate to reticulate sculpturing represent the ancestral, non-derived pollen type of the Santalales; all other pollen features appear to be derived. (ii) There is a general match between genetic divergence/derivedness, as expressed by branch lengths and root-tip distances in Su et al. (2015)’s tree, and the probability of accumulating derived pollen features and distinct (unique) pollen types; this is exemplarily exhibited by the former Balanophoraceae *s.l*., which are genetically very distinct and can show very different pollen types; note also root-tip distances for Loranthaceae and Viscaceae, the other two families with increased pollen variation. (iii) The probability of putatively derived pollen features, such as strongly oblate shapes, (panto)porate pollen, >3 apertures, concave outlines in polar view, non-elliptical outlines in equatorial view, clavate/echinate/distinctly rugulate/striate sculpturing, increases the ‘higher’ one goes up in the Santalales tree. (iv) Individual morphological traits occasionally have been evolved in parallel; however, they can be restricted to a subtree of the Santalales tree. (v) There is no evidence for back-mutation to (more) ancestral forms; pollen evolution appears to be unidirectional. (vi) Genera with strongly different pollen do not belong to the same family/phylogenetic lineage or represent closely related taxa, with one notable exception: the Type A vs. Type B pollen of the Loranthaceae. (vii) Often when pollen variation is found within a lineage (family), types more similar to the ancestral Santalales pollen are found in addition to the derived types; the least derived pollen is then usually found in species close, in phylogenetic or absolute terms, to the root node of the lineage. (viii) It is clear that the characteristic Type B pollen of Loranthaceae, and the Type C and Type D pollen derived from it within the Psittacanthinae, is unique within the Santalales and it cannot be confused with pollen of any other Santalales family. Some of its individual morphological aspects, oblateness, convex-triangular to lobate outline in polar view, syncolpate aperture organisation, striate sculpturing (see Figure 4), can be found in their relatively close (Schoepfiaceae) or more distant relatives within the Santalales core clade, but the combination of these features are unique. However, pollen very similar to the Type A pollen of *Tupeia* and *Phthirusa hutchisonii*, or easily derived from/to such a pollen type can be found in other members of the Santalales core clade, in particular within the Opiliaceae (the first diverging lineage of the sister clade of Loranthaceae and sister groups) and Santalaceae (Feuer 1977; Feuer & Kuijt 1978; this study).

With regard to the general diversity patterns of pollen morphologies in Santalales and their lineages as summarised in this section, it is unlikely that theType A pollen evolved from the Type B pollen within the Loranthaceae. This brings it in conflict with the currently accepted, outgroup-inferred Loranthaceae root. The most plausible explanation is that Type A and Type B pollen of the Loranthaceae are either directly derived from the primitive Santalales pollen type or from an intermediate, extinct pollen type that shared more features with the Type A than the Type B pollen of Loranthaceae and could represent a link between the two main subclades within the core Santalales.

### How reliable is the outgroup-inferred Loranthaceae root in current molecular trees?

With respect to all other Santalales (preceeding section, File S7), the Type B pollen of Loranthaceae can be considered to be one of the most derived pollen within the order and represents a shared, derived characteristic, a potential synapomorphy, of nearly all Loranthaceae, whereas the Type A pollen and similar pollen types are either ancestral within the core Santalales or have been evolved in parallel several times from the primitive Santalales pollen encountered in the ‘Olacaceae *s.l*.’ grade. If there would be a meaningful probability that an Type A pollen can evolve in parallel from a Type B pollen, one should find pollen at least somewhat similar to the Type B pollen of Loranthaceae also in other lineages of the second main clade of the core Santalales such as Opiliaceae, Santalaceae (in particular) and Viscaceae, but this is not the case (File S7). We also are unaware of any other angiosperm lineage where pollen similar to the Type B of the Loranthaceae evolved into a pollen similar to the Type A pollen of *Tupeia* and various other core Santalales. It is also unlikely that Type B (and its derivates Type C and D) should have evolved independently several times in the Loranthaceae lineage from a pollen similar to Type A. If we assume that the currently accepted root (Vidal-Russell & Nickrent 2008b; Su et al. 2015) is correct, Type B would need to have evolved at least six times from the (more) ancestral Type A pollen (e.g. within the root parasitic lineages, the Psittacantheae, the Elytrantheae and the Lorantheae), and at least once convergently in the Paleogene (Macphail et al. 2012; based on divergence estimates by Vidal-Russell & Nickrent 2008a). An interesting analogy to Type A vs. Type B pollen in Loranthaceae is that the Schoepfiaceae, which share some characteristics with Type B pollen of Loranthaceae, also have a pollen very distinct from their sister clade, *Misodendron*/Misodendraceae, the latter with more resemblance to Type A than found in any other member of the Loranthaceae-including clade of the Santalales core group so far. Hence, the simplest explanation for the Type A pollen of *Tupeia* would be that the outgroup-inferred Loranthaceae root (Wilson & Calvin 2006; Vidal-Russell & Nickrent 2008b; Su et al. 2015) is misinformed due to ingroup–outgroup branching artefacts such as long-branch attraction (e.g. Sanderson et al. 2000; Lockhart et al. 2006); and that both the Type A and Type B pollen are confined to mututally monophyletic sister lineages. In other words, *Tupeia* represents the first diverging branch in the (extant) Loranthaceae and not the root parasite *Nuytsia* as inferred based on molecular cladograms/phylograms including outgroups.

Primary relationships in Loranthaceae are poorly resolved even using concatenated oligo-gene data sets (Figures 2, 3; Vidal-Russell & Nickrent 2008b; Su et al. 2015). The recently established sister clade of the Loranthaceae (Mystropetalaceae; ‘Balanophoraceae B’ in Su et al. 2015, figure 1B) is distinctly long-branched, and earlier used outgroups Misodendraceae and Schoepfiaceae (Vidal-Russell & Nickrent 2008b) are genetically very distant from Loranthaceae as well. The data provided by Su et al. (2015) are strongly indicative for ingroup–outgroup long-branch attraction involving *Nuytsia* (details provided in File S6). *Nuytsia* is the only member of the Loranthaceae and sistergroups subtree with data covering all seven gene regions included in Su et al.’s (2015) study.

Despite this, the outgroup (all other Santalales and non-Santalales) + *Nuytsia* vs. all other Loranthaceae split, which defines the Loranthaceae root, is only poorly supported (BS_ML_ < 50, PP << 1; Su et al. 2015, figure 1B; File S7). This is however not due to the substantial data gaps or low-amplitude signals: when the outgroup is limited to the putative sisterclades of Loranthaceae, i.e. using a less comprehensive outgroup sample, the support for the *Nuytsia* root becomes unambiguous (Table XII). Comprehensive outgroup samples can compensate (to some degree) for ingroup–outgroup long-branch attraction (e.g. Felsenstein 2004, and references cited therein), hence, the fact that a comprehensively sampled outgroup (representatives of all other Santalales lineages) produces much lower support than one including only the next-relatives is a first indication of a misinformed outgroup-based root.

**Table XII.** Differential support for critical branches regarding the placement of the Loranthaceae root based on the data provided by Su et al. (2015).

	Su et al. (2015)		Loranthaceae + sistergroups^b^				
	7-gene	5-gene AA^a^	7-gene	*matK*	7-gene		6-gene
				Excluding:	*Nuytsia*	*Nuytsia + Atkinsonia*	*Nuytsia*
	BS_ML_/PP	BS_ML_	BS_ML_	BS_ML_	BS_ML_	BS_ML_	BS_ML_
PREF: OG + *Nuytsia* | remaining Loranthaceae	<50/0.65	99	100	100	[N/A]	[N/A]	[N/A]
PREF: OG + *Atkinsonia + Nuytsia* | remaining Loranthaceae	53/0.99	[n.f.]	62	75	47	[N/A]	<20
ALT: Gaiandreae clade	≤47/≤0.01	[n.f.]	<20	<20	27	[N/A]	37
PREF: OG + root parasites | aerial parasites	<50/0.52	[n.f.]	60	66	30	<20	<20
ALT: OG + Elytrantheae | remaining Loranthaceae^c^	?/≤0.35	≤1	0	0	15	39	40

Note: Shown is the support based on non-parametric bootstrapping under maximum likelihood (BS_ML_) and Bayesian-inferred posterior probabilities (PP) for preferred (PREF: seen in Su et al.’s [2015] 7-gene tree) and alternative (ALT: competing) splits relating to the outgroup-inferred Loranthaceae root. Further abbreviations: [n.f.] = not found in the according tree, value unknown; [N/A] = not applicable. If the Loranthaceae root would be well-informed and not prone to data and branching artefacts, support for the critical branches listed here should increase and not decrease with comprehensive outgroup sample (Su et al. 2015, 7-gene) and remain high independent of which root parasitic taxon is included in the study; ^a^ Maximum likelihood (ML) analysis based on the sequence data for five of the seven gene region translated into amino-acid sequences; ^b^ Analysis done here using the data and matrix provided by Su et al. (2015) on Loranthaceae and their sistergroups Mystropetalaceae, Misodendraceae, and Schoepfiaceae; ^c^ Topology seen in the ML tree after *Nuytsia* is removed from the data set (see Fig. S6–S8 in File S6).

Furthermore, the ingroup–outgroup split and the support of the branches defining the subsequent root parasitic grade is solely informed by signal from the nucleotide sequences of the plastid *mat*K gene (File S6), but there are no plastid data on the Mystropetalaceae. When the long-branching *Nuytsia* is removed from the data set including Loranthaceae and their sister groups, the support for the root parasitic grade collapses again. The same is observed when excluding the *mat*K data (Table XII). Thus, ingroup–outgroup long-branch attraction may be inevitable in the case of Loranthaceae and one should consider the possibility that the outgroup-informed Loranthaceae root may be wrong (see Bomfleur et al. 2015 for an example with a fully resolved molecular phylogenetic framework and perfectly misleading outgroup-defined root).

*Tupeia*, and possibly *Phthirusa hutchisonii*, could be remnants of largely extinct lineages and sisters to the remaining Loranthaceae as evidence by their Type A pollen. Extinction of several lineages involving in a first, fast radiation would also explain the general problem to resolve deep relationships within the Loranthaceae (e.g. Shavit et al. 2007). Rerooting the best-resolved, regarding deeper relationships, current Loranthaceae tree (Su et al. 2015) with *Tupeia* would result in recognising a primary split between a lineage of root parasites (Nuytsiaea and Gaidendreae) and New World epiphytes (Psittacantheae) as sister to a Old World-Australasian clade of epiphytes (Elytrantheae and Lorantheae). The Type B pollen would be a synapomorphy of the Loranthaceae *s.str.* (explaining its continuous record from now till the Paleogene), and the shift to epiphytism, a shift that convergently happened in Santalales several times between and within lineages (Vidal-Russell & Nickrent 2008a), would have needed to happen just three times (*Tupeia*, Elytrantheae/Lorantheaea, Psittacantheae) instead of once (Vidal-Russell & Nickrent 2008b; but see also Wilson & Calvin 2006). The three root parasites would form a moderately supported clade, sister to an equally supported New World Clade.

## Conclusion and guidelines for further analyses of Loranthaceae

Pollen morphology and ultrastructure of Loranthaceae is in general agreement with unambiguous and potential phylogenetic relationships as inferred from molecular data. Some of the putatively derived pollen types are highly diagnostic, limited to molecular clades with ample support, allowing straightforward identification of the systematic affiliation of the plant that produced the corresponding pollen. Pollen of some genera show unique, highly diagnostic (autapomorphic) features not found in any other Loranthaceae (or angiosperm as far as known). Thus, the here established framework provides a first basis for the re-investigation of the Loranthaceae fossil pollen record. Moreover, the currently available genetic and palynological data calls for a further refinement of Loranthaceae taxonomy, which is so far primarily based on flower characteristics (e.g. Nickrent et al. 2010; Kuijt 2011) and, in parts, poorly supported by molecular evidence and in conflict with pollen morphological evidence. A final reconciliation of Loranthaceae systematics and evolution will require a better genetic sampling – more representatives of so far undersampled lineages, additional gene regions that provide less ambiguous signals regarding the deep relationships or in-depth analysis of incompatible signals (Files S1, S5) – accompanied by the study of pollen of each so far and in future sequenced species. The general conservation of pollen morphologies over long periods of time and within phylogenetic lineages (e.g. Denk & Grimm 2009; Friis et al. 2011; Grímsson et al. 2011; this study) make them more valuable as taxonomic indicators than any other morphological feature at the supergeneric level. An example of the general high diagnostic value of pollen morphology in Santalales are the studies of Maguire et al. (1974) and Feuer (1977), which document the diverse pollen of ‘Olacaceae’ now placed in distinct families/molecular-defined clades (Aptandraceae [*Aptandra* and *Anacolosa* clades], Strombosiaceae, Ximeniaceae; [e.g. APG 2009; Su et al. 2015]). It is particular intriguing that Feuer (1977) pointed out which genera/groups are related and which are not based on the pollen morphology and ultrastructure, and partly anticipated re-classifications done only three decades later based on molecular data. The palynologically well-studied Psittacanthinae would much profit from in-depth (species-level) molecular analyses. In particular, comprehensive genetic and palynological data needs to be produced for the former species of *Ixocactus* now re-included in *Phthirusa* to clarify the position of *Phthirusa hutchisonii* with its atypical pollen. Palynological data of *Phthirusa inorna* and genetic data on *Passovia* species are needed to verify hypotheses about pollen evolution in the Psittacanthinae, and molecular data on further species of the other genera would allow testing hypotheses about pollen evolution in this lineage and generic concepts. For completeness, pollen morphology of *Cecarria* (clade G), *Ileostylus* (clade H) and further Amyeminae (clade I) needs to be studied, in order to round up our knowledge of pollen diversity in the Lorantheae and to which degree it relates to genetic divergence.

## Supplementary Material

Grimsson et al. Online supplementary material
