# Supplementary material for: Evolution of pollen morphology in Loranthaceae
Source: Grana. 2017 Feb 20;57(1-2):16–116. doi: 10.1080/00173134.2016.1261939 (PMC5771552; doi:10.1080/00173134.2016.1261939)
Supplement: Grimsson et al. Online supplementary material [file SGRA_A_1261939_SM9964.zip › File_S2_Curation&Setup.pdf]

NCBI GenBank Harvest, 4/3/2014

Search strings taxa and gene regions

Taxa

Loranthaceae: txid3963[Organism:exp]

Gene regions

*mat*K gene: … AND matK [gene]
*rbc*L gene[1]: … AND rbcL [gene] NOT "atpB-rbcL" [title]
*trn*L-*trn*F spacer … AND "trnL-trnF" [title]
18S rDNA: … AND (18S [title] or "small subunit" [title]) NOT "internal" [title]
ITS region of the 35S rDNA[2]: … AND "5.8S" [title]
25S rDNA: … AND 26S [title] NOT "internal" [title]

[1] Four sequences of *Benthamina* sp. SH-2010 (Tobe et al., unpubl.; AB586377–AB586380) not caught by search and were manually added.
[2] One ITS2 accession (*Tristerix penduliflorus*, DQ442975, Amico et al., *Am. J. Bot.*, 2007) comprising 197 bp (ITS2 only) will not be caught. Being only a short fragment without extra information, the sequence was not manually added.

Processing

Genebank flatfiles transferred into FASTA-format using GBK2FAS (Göker et al. 2009, see batch file for options). Auto-alignments done with MAFFT v. 6.935b (Katoh et al. 2005; Katoh & Standley 2013), optimal algorithm chosen by the programme.

Dataset	Algorithm
[1] 18S	L-INS-i
[2] ITS	FFT-NS-i
[3] 25S	FFT-NS-i
[4] matK	L-INS-i
[5] trnL-trnF	FFT-NS-i
[6] rbcL	L-INS-i

All alignments were inspected by eye, and their ends were trimmed. As a rule we truncated the alignments for less than 4 accession on the 5' and 3' ends.

Nuclear-encoded 18S rDNA

General — The data comprise near-complete 18S rDNA sequences showing only restricted length-polymorphism at pos. 184–189 (CT-dom. motif), 223–233 (upstream-GC, downstream CT) and within the terminal loop of stem 49 (tl49) at the 3' end of the 18S rDNA. Several single-nt gaps are introduced in which a single of the sequences shows an extra nucleotide typically the same then before or after the gap.
Curation—Isolated single-nt gaps are likely due to sequencing/editing artefact rather than representing actual mutations and have been corrected for. Right-aligned the length-polymorphic motives at pos. 184ff; left-aligned pos. 229ff; centre-aligned tl49-motives. Several accessions (*Gaiadendron punctatum*, L24143; *Helicanthes elastica*, EU544328; *Erianthemum dregei*, L25679; *Oedina pendens*, EU544336; *Ligaria cuneifolia*, L24152; *Struthanthus oerstedii*, L24421) show increased amount of deviations within strongly conserved stem regions. Where conclusive comparative data was lacking, the sequence was kept as-is, otherwise blanked out (replaced by missing data symbol).

Nuclear-encoded ITS region

General—The nrDNA spacers are highly divergent and length-polymorphic at the family level, resulting in a very gappy auto-alignment. The ITS1 cleavage site region, the 5.8S rDNA, and the generally more conserved parts of the ITS2 are successfully recognised as alignable blocks due to their high conservation level and overall lack of length-polymorphism.

The data was not included in family-wide analyses due to potential alignment artefacts on the overall topology.

Curation—Except for right-aligning the start of the 5.8S rDNA, the alignment was left un-changed.

Problematic data—Three accessions show a number of likely pseudogenous mutations in the 5.8S rDNA (*Dendrophthoe constricta*, DQ333840, 3' part; *Helixanthera parasitica*, DQ333823, central part; *Psittacanthus schiedeanus*, DQ333859, 3' part, 5' part with small missing data blocks).

In case of DQ333840 mutational patterns linked to pseudogeny are also apparent in the ITS1 and start of ITS2 (identifiable by comparison with the second accession of the genus and other members of the Lorantheae).

In the case of DQ333823, pseudogeny is less obvious in the ITS1 and ITS2: the sequence appears to be a mix of ITS variants with different levels of pseudogeny as indicated by a number of polymorphic calls (Y and R at overall conserved C and G positions).

DQ333859 may be a chimera (or recombinant) of a non-pseudogenous and (weak) pseudogenous ITS variants: the area of overlap between typical forward and reverse reads has been blanked out by the authors indicating problems with the direct PCR sequencing. Lacking comparative data of this genus/species, the sequence was kept as-is.

The 3' portion of the ITS2 in *Notanthera heterophylla* DQ333855 is entirely different from other accession of the subtribe/tribe and markedly off-alignment. The strand portion finds no similar hits in gene banks. Based on the general distinctness of the ITS of this taxon the 3' ITS2 may be genuine and has been kept as-is.

One of the two accessions of *Taxillus* (*T. chinensis*, JX177497) differs strongly from the other sequence and is essentially off-alignment within ITS1 and the larger part of ITS2. Subsequent MEGABLAST identifies the sequence as *Viscum* (same order, family Viscaceae).

Nuclear-encoded 25S rDNA data

General—Data comprise near-complete 25S rDNA sequences. Variation and minor length-polymorphism is limited to the D1–D4 regions of the 25S rDNA. Markedly divergent patterns in generally sequential- and structural-conserved regions are limited to a few accessions (→ **Problematic data**)

Curation—Left-aligned C-rich motif at pos. 2078ff.

Problematic data—Accessions of *Diplatia furcata* (EU544368), *Helicanthes elastica* (EU544375), *Lepidaria* cf. *forbesii* (EU544378), and *Ixocactus sp.* (EU5443779) show an increased number of substitutions in generally conserved regions. In the case of *Ixocactus* the sequence inflicts single-nt gaps. In case EU544368 many deviations are indicative for pseudogeny (G→A, C→T) in addition to deviations that may be linked to bad sequence reads/editing artefacts (e.g. AATTA at pos. 485–489 instead of the consensual ATTGA). The sequence was excluded from analysis.

EU544375 represents a unique 25S rDNA fragment, with best BLAST hits of 92–93% identity covering a wide range of taxa and clades (Solanales: *Schizanthus,* Apiales: *Bolax*; Saxifragales: *Saxifraga, Telesonix*; Ranuculales: *Borax*; etc.); top hits did not include members of Loranthaceae. The sequence was tentatively excluded from analyses as putative contaminant.

Accession EU544378 shows increasing amount of pseudogenous positions (G→A, C→T mutations in generally conserved regions) towards the 3' part. The sequence was excluded from analysis as showing tendency towards pseudogeny.

Accession EU5443779 is composed of two strands. The first strand (170 nt from the 5' end) finds best BLAST-hits among the Loranthaceae, but lower identity than usually found in when BLASTing a member of the family. Then comes a sequencing gap and the following remaining c. 1200 nt find highest identities (>90%; obtained with MEGABLAST) only with members of other families of the Santalales (*Osyris,* Santalaceae; *Haloragis*, Halogaraceae) and unrelated singletons such as *Eucnide bartonioides* (JF321124), *Loasa vulcanica* (JF321125; Cornales), *Fendlera rupicola* (AY260041), *Dryas octopetala* (JF317384; Rosales), and *Eucryphia lucida* (AF036494, Oxalidales). Based on this

result it is uncertain whether the sequence is genuine or a mislabelling/artificial chimera; hence, it was tentatively excluded from all analysis.

Plastid *mat*K gene

General—Data includes up-to-near-complete *mat*K sequences starting with the first codon in three accessions (stop codon seems to be not covered in any accession). More than 3-taxon coverage starts at the 27th–xxx codon (alignment was accordingly truncated). Length-polymorphism is restricted to singleton duplications or eliminations.
Curation—Regions with duplications/elimination, the general alignment were adapted to codon positions when necessary. Defect (due to missing single-nucleotides) and incomplete codons were corrected for.
Problematic data—The 5' part of accession EU544409 (*Aetanthus nodosus*) is markedly different from other members of its tribe and the entire family. MEGABLAST revealed that the first c. 260 nt are highly identical to accession of several species of *Acacia* (Fabales). The remainder of the sequence falls within the variation of the Loranthaceae. Thus, the accession is a (artificial) chimera of contaminated material and unverified *Aetanthus* (no comparative data available of the genus), and has to be excluded from analysis.

Plastid *trn*L-*trn*F intergenic spacer

General—The data includes the *trn*L intron, the 3' *trn*L exon (identified by comparison to *Panax ginseng* complete plastome, KC686331), the *trn*L-*trn*F intergenic spacer and up to nearly complete *trn*F genes (15–21 nt missing in longest sequences). Most length-polymorphism in the intron is linked to duplications in a single or few sequences and a prominent multiple-A motif (pos. 190ff)
Curating—Alignment in singletons at pos 132–166 (in three accessions) were corrected for duplication patterns. Left-aligned pos. 638–653

Plastid *rbc*L gene

General—The matrix covers the 469 codons of the *rbc*L gene (starting with the 8th codon, last three codons of complete *rbc*L not represented in GenBank data; reference sequence *Panax ginseng* KC686331). Most variable sites (~75%) refer to the 3rd codon position.
Curating—Alignment of *rbc*L is straightforward (generally no length polymorphism). The region is generally conserved at the genus/subtribe level, which facilitates the identification of putative mislabelled sequences. The data includes one unidentified Loranthacea *rbc*L, which was not considered for further analyses.
Problematic data— Three sequences of *Taxillus chinensis* (JF949992; JN687568; KF447376) differ markedly from the other eight sequences of the species and other members of the genus/tribe (visible from pos. 250 onwards). MEGABLAST identified JF949992 as Elytrantheae/Gaiadendreae (99% identity; verified on the alignment) and JN687568 as Viscaceae, another family of the Santalales (most similar to *Viscum*, 97% identity; no Loranthaceae among the top hits). The sequences either represent mislabelled or misidentified specimens. KF447376 received no significant hit with identity >93% (best hits with *Scurulla*, the sister genus of *Taxillus*, generic affinity verified at hand of the full alignment). The sequence represents either a bad sequence read or a strongly aberrant *rbc*L gene (potential pseudogene). All three sequences were excluded from analyses.
A similar sequence type than in JN687568 is found in the only accession of *Oryctanthus cordifolius* (JQ592409), differing from the otherwise near-identical sequence of Psittacanthinae. MEGABLAST identifies the sequence accordingly as Viscaceae *rbc*L (*Phoradendron*; 98% identity). The sequence is either mislabelled or *Oryctanthus* is not a Loranthaceae and has been omitted.
Another problematic sequence is the one representing *Macrosolen brandisianus* (JN687566) which differs increasingly towards the 3' end from other accessions of the genus/tribe. Deviation is usually related to same-nucleotide repeats (e.g. G**GT**GGT instead G**TG**GGT in all other Lorantheae at pos. 349ff; AA**T**TTG instead AA**A**TTG as pos. 460ff). The problematic sequence part encompasses 50% of the total sequenced strand (c. 500 nt), the first half of the sequence is identical to other Lorantheae. The sequence was tentatively excluded from analyses.

Set-up for phylogenetic analyses

Modal and strict genus-consensus sequences were computed from the basic alignments using the programme G2CEF (Göker & Grimm 2008); gaps were treated as missing data or fifth state (batch and files are included in the subfolder *Consensing* in this Online Supplementary Archive, OSA). For further analyses the strict consensus sequences with gaps treated as missing were used.

Phylogenetic inferences relied on maximum likelihood (ML) as the optimality criterion. Following the 2nd author's experience with numerous oligonucleotide datasets below the family level, we did not perform any substitution model test or partition-finding test. All analyses, files can be found in subfolder *ML* in OSA, were done using RAxML v.7.2.6 (Stamatakis 2006a; Stamatakis et al. 2008). RAxML was invoked on a Win 7 operating system using batch files (included in subfolder *ML*). Based on the concatenated dataset, unpartitioned and partitioned (using nine partitions based on the primary genetic properties of the included gene regions) inferences were done in addition to single-gene analyses. In addition, a first-level gene-jackknifing procedure was applied. This allowed to check for potential incongruence and data artefacts in the concatenated data set. All inferences used the -f a option that performs a (in our case fast, option -x) bootstrapping analyses with subsequent inference of the "best-known" ML tree under a general-time-reversible substitution model allowing for site rate variation, modelled using a gamma distribution (GTR+Γ model) in the final optimisation step. During bootstrapping and the first (fast) tree inference step, the per-site approximation for the gamma distribution was used (option -GTRCAT; Stamatakis 2006b). Number of necessary bootstrap replicates were determined by the extended majority rule consensus criterion (Pattengale et al. 2009), bootstrap analyses were automatically cut off when reaching a maximum of 1000 replicates.

There is no strong incongruent signal between the concatenated gene regions, nevertheless, data gaps and signal strength issues hinder the inference of a finely resolved molecular tree. In particular deeper relationships remain ambiguous. Since our focus was not to provide a "better" molecular-phylogenetic hypothesis but to qualitatively assess to which degree pollen morphology agrees with signal from molecular data, we did not aim to resolve the resolution issues. Instead, we investigated and visualised competing support for topological alternatives using bipartition (bootstrap support) networks (Grimm et al. 2006), a special form of consensus networks (Holland & Moulton 2003). Bipartition networks were computed based on all ML bootstrap replicate samples using SPLITSTREE v. 4.13.1 (Huson & Bryant 2006). The bipartition network based on the partitioned, concatenated data analysis forms the basis for the qualitative mapping of pollen (text-Figures 4, 15, 22, 33, 34).

Note on the signal in current molecular data

Already the inferences produced by Vidal-Russell and Nickrent (2008) revealed two sources of incompatible signal and low support: low-amplitude signals and occasional incongruence between the concatenated 25S rDNA and plastid sequence data (Table S1). Being primarily interested in the general correlation of pollen types with currently accepted phylogenetic-systematic groups, we refrain here from an in-depth exploration of incompatible signal and low support. Nevertheless, we ascertain, based on our molecular re-analysis of available data, that a phylogenetic hypothesis aiming at resolving deep relationships or the refined placement of problematic taxa would require independent analysis of nuclear and plastid data. Furthermore, the genetic coherence of genera would need to be further tested.
Hence, the phylogenetic inferences in this study should be viewed as being preliminary. They show which aspects of the Loranthaceae tree are more or less well understood and where the signal from the molecular data is (highly) ambiguous allowing for two or more alternatives. Thus, we did not map our pollen types on the molecular tree, but on the bipartition (bootstrap consensus) network that is able to visualise potentially competing splits inflicted by the, partly (to various degrees) conflicting, underlying molecular data.

Table S1. Tabulation of support for preferred, partly conflicting phylogenetic splits based on the inferences provided in Vidal-Russel and Nickrent (2008)

	25S rDNA			rbcL/matK/trnLF			Conc. 5-gene		
	MP-BS	ML-BS	PP	MP-BS	ML-BS	PP	MP-BS	ML-BS	PP
Ground parasites | aerial parasites	N/A	N/A	N/A	<50	<50	81	<50	<50	83
Nuytsia+Clades A, B | Clade C	<50	<50	80	N/A	N/A	N/A	N/A	N/A	N/A
Gaiadendron sister to aerial parasites	N/A	N/A	N/A	69	72	98	<50	<50	73
Gaiadendron sister to Clade D	<50	<50	58	N/A	N/A	N/A	N/A	N/A	N/A
A-B subtree	N/A	N/A	N/A	78	94	100	94	93	100
Clade A (*Alepis, Peraxilla*)	83	87	88	100	100	100	100	100	100
Clade B (Elythranthinae; *Amylotheca* through *Lysiana*)	72	77	100	99	100	100	99	100	100
Clade C	<50	<50	80	N/A	N/A	N/A	<50	<50	86
Clade D acc. 25S tree (excl. *Ligar.-Trist.*)	<50	<50	77	N/A	N/A	N/A	N/A	N/A	N/A
Clade D acc. plastid tree (incl. *Ligar.-Trist.*)	N/A	N/A	N/A	<50	<50	72	<50	<50	55
Desmaria-Tupeia	<50	<50	94	*Tupeia* not included			<50	<50	<50
Ligar.-Trist.-Nothanth.-Clade E	N/A	N/A	N/A	<50	<50	63	<50	<50	<50
Ligaria-Tristerix-Notanthera	N/A	N/A	N/A	<50	<50	61	N/A	N/A	N/A
Notanthera-Clade E	<50	<50	91	N/A	N/A	N/A	<50	<50	74
Clade E	88	90	96	100	100	100	99	99	100
Tripodanthus sister to rem.	76	80	100	<50	<50	63	83	72	100
Ligaria-Tristerix-Clade F	<50	<50	69	N/A	N/A	N/A	N/A	N/A	N/A
Clade F	77	92	100	100	100	100	100	100	100
Clade G (*Cecarria-Loranthus*)	73	87	100	100	100	100	100	100	100
Clade H-I-J	78	80	100	N/A	N/A	N/A	N/A	N/A	N/A
Clade H *(Iliostylus-Muellerina*)	90	91	100	100	100	100	100	100	100
Clade I-J	<50	64	99	N/A	N/A	N/A	100	100	100
Clade I acc. 25 S tree (incl. *Baranthus*)	<50	94	100	N/A	N/A	N/A	<50	59	59
Clade I acc. plastid tree (excl. *Baranthus*)	64	73	100	80	93	100	88	92	74
Clade J acc. 25 S tree*	<50	<50	100	<50	58	92	<50	55	66
Clade J acc. plastid tree	N/A	N/A	N/A	<50	79	100	<50	73	74
Dendrophthoe-Helixanthera cocc.-Tolypanthus	*Tolypanthus* not incl.			100	100	100	<50	63	73
Dendrophthoe-Helixanthera cocc.	*H. cocc.* not incl.			[Not annotated]			<50	76	95
Scurulla-subclade	<50	<50	52	N/A	N/A	N/A	N/A	N/A	N/A
Scurulla-Taxillus	<50	<50	80	100	100	100	100	100	100
Dendrophthoe-Helixanthera cyl.	<50	<50	80	N/A	N/A	N/A	N/A	N/A	N/A
"Core J"	<50	<50	76	<50	<50	<50	<50	<50	75

Note: The Ligarinae (highlighted by red font) show markedly incongruent nuclear and plastid signals.
* Including *Helixanthera cyl., Scurulla, Taxillus*

Documentation

All analysis files are included in the OSA in corresponding subfolders. See ReadMe.txt in top folder for file labels and brief description of content. The OSA is mirrored at www.palaeogrimm.org/data, for questions contact the 2nd author (mail-to: loranths@palaeogrimm.org).

Göker M, García-Blázquez G, Voglmayr H, Tellería MT, Martín MP. 2009. Molecular taxonomy of phytopathogenic fungi: a case study in *Peronospora*. PLoS ONE 4: e6319.

Göker M, Grimm GW. 2008. General functions to transform associate data to host data, and their use in phylogenetic inference from sequences with intra-individual variability. BMC Evolutionary Biology 8: 86.

Grimm GW, Renner SS, Stamatakis A, Hemleben V. 2006. A nuclear ribosomal DNA phylogeny of *Acer* inferred with maximum likelihood, splits graphs, and motif analyses of 606 sequences. Evolutionary Bioinformatics 2: 279-294.

Holland B, Moulton V. 2003. Consensus networks: A method for visualising incompatibilities in collections of trees. In: Benson G, Page R, eds. Algorithms in Bioinformatics: Third International Workshop, WABI, Budapest, Hungary. Proceedings, 165-176. Berlin, Heidelberg, Stuttgart: Springer Verlag.

Huson DH, Bryant D. 2006. Application of phylogenetic networks in evolutionary studies. Molecular Biology and Evolution 23: 254-267.

Katoh K, Kuma K, Toh H, Miyata T. 2005. MAFFT version 5: improvement in accuracy of multiple sequence alignment. Nucleic Acids Research 33: 511–518.

Katoh K, Standley DM. 2013. MAFFT multiple sequence alignment software version 7: improvements in performance and usability. Molecular Biology and Evolution 30: 772–780.

Pattengale ND, Masoud A, Bininda-Emonds ORP, Moret BME, Stamatakis A. 2009. How many bootstrap replicates are necessary? In: Batzoglou S, ed. RECOMB 2009., 184–200. Berlin, Heidelberg: Springer-Verlag.

Stamatakis A. 2006a. RAxML-VI-HPC: Maximum-Likelihood-based phylogenetic analyses with thousands of taxa and mixed models. Bioinformatics 22: 2688-2690.

Stamatakis A, Hoover P, Rougemont J. 2008. A rapid bootstrap algorithm for the RAxML web servers. Systematic Biology 57: 758–771.

Stamatakis A. 2006b. Phylogenetic models of rate heterogeneity: A high performance computing perspective.

Vidal-Russell R, Nickrent DL. 2008. Evolutionary relationships in the showy mistletoe family (Loranthaceae). American Journal of Botany 95: 1015–1029.
