# Supplementary material for: Evolution of pollen morphology in Loranthaceae
Source: Grana. 2017 Feb 20;57(1-2):16–116. doi: 10.1080/00173134.2016.1261939 (PMC5771552; doi:10.1080/00173134.2016.1261939)
Supplement: Grimsson et al. Online supplementary material [file SGRA_A_1261939_SM9964.zip › File_S6_Reanalysis_of_Su_et_al_loranth_data.pdf]

File S6: 'Quick-and-dirty' re-analysis of the Su et al. (2015) data of Loranthaceae and their sister groups by GWG

Following the dispute with two anonymous reviewers regarding the quality of the molecular data used for our study and the showed results, the data of Su et al. (2015) is re-investigated using the same approach than used to study our data set to *(i)* assess the potential of the newly sequenced gene regions for elucidating relationships within the Loranthaceae, *(ii)* explore what is behind the non-unambiguous support of most branches in the Loranthaceae subtree of Su et al., and *(iii)* investigate how the fragmentary data of the sister groups informs the Loranthaceae root. This allows me to a) reject the *Nuytsia* root as a likely branching artefact and b) show that the Su et al. data would be in no aspect better than our data set regarding the questions raised in and the purpose of our paper.

Ambiguous signals from new gene regions sequenced by Su et al. (2015)

It has been suggested by one anonymous reviewer that the additional gene regions used by Su et al. (2015) helped to stabilise the backbone topology of the Loranthaceae subtree, hence, the higher support for critical branches in Su et al.'s tree compared to our analysis and data set (text-**Figs 2, 3**). Two of the additional genes, the nuclear *RPB2* gene and the *matR* are crucial for recognising the Mystropetalaceae as sister to Loranthaceae and enforcing the outgroup-inferred root, as they increase the (absolute) distance between *Nuytsia* and the remainder of the family, hence, make the matrix more vulnerable to ingroup-outgroup long-branch attraction (LBA). Regarding the Loranthaceae, most variable sites within these additional gene regions show unique mutations, i.e. a single of the sampled accessions deviates from what is seen in the others. One (*RPB2*), five (*matR*), and nine (*accD*) sites, respectively, agree with actual clades in the tree by Su et al, the remaining variable sites (total of 195 in *RPB2, 98* in *matR* and 122 for *accD*) show unique (parsimony-uninformative) or stochastic mutations (same mutation occurring in two or more distantly related taxa[1]).

The **mitochondrial *matR* gene** provides an near unambiguous bootstrap (BS) support for a split between *Nuytsia* + *Macrosolen* and *Gaiadendron* + Lorantheae, i.e. rejects simultaneously the root parasitic clade and the sister relationship of Elytrantheae and Lorantheae in Su et al's tree (**Fig. S6-1**). The *matR* data for loranths and their sistergroups are nevertheless very interesting (see also Su et al., fig. S7), particular with respect to the huge indels seen in the data, something not seen in e.g. all-angiosperm matrices such as the one of Soltis et al. (2011). Note that these gaps nevertheless could enforce an ingroup-outgroup LBA with *Nuytsia*, and thereby increase the support along the root parasitic grade branches in the concatenated tree, simply because there are no *matR* for most other Loranthaceae including

[1] This is by the way the reason why MP BS is generally low. Already from Su et al.'s alignment, it is blatantly obvious that these data are not the result of rare convergent or even synapomorphic changes accumulating along trivial branching events (which would be necessary for applying MP). The only reason MP doesn't get everything wrong is because of LBA: it will always join the most distinct taxa, and for the deeper relationships in Santalales this may not be the worst choice. Its indecisiveness (see Su et al., ES1) compared to ML/BI may also be due to the fact that there are too many long-branching lineages, so MP gets literally lost in LBA. In this situation, and if MP would be deemed necessary, an informative comparison would have been to plot the ML-BS/PP vs. the MP-BS values for all bipartitions found in the BI sampled topologies and ML and MP bootstrap samples.

many critical ones regarding the primary splits (*Atkinsonia*, Ligarinae, Notantherinae, *Tupeia*).

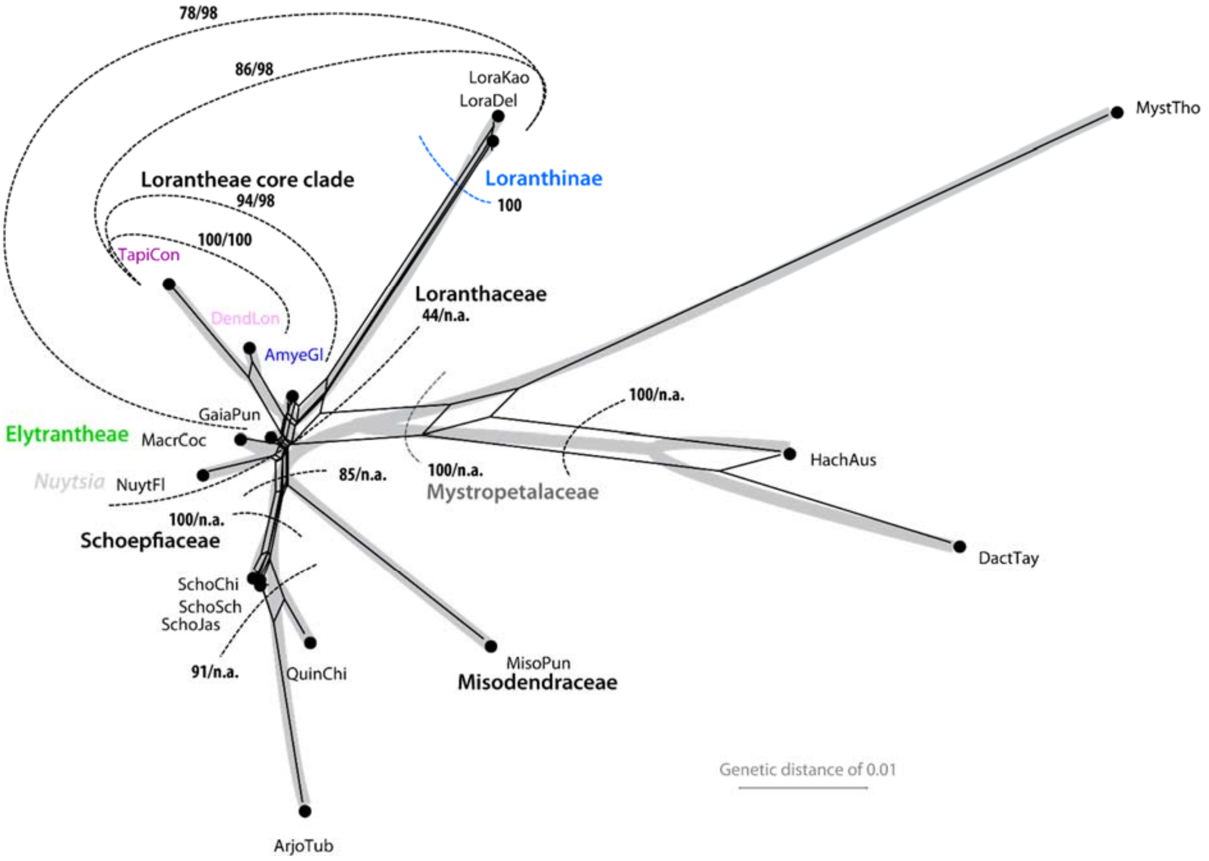

Figure S6-1: Information content of the mitochondrial region (*matR* gene) included by Su et al., for relationships within Loranthaceae (data not included in our study due to its poor taxonomic coverage). Shown is a distance network based on uncorrected pairwise distances; a tree topology optimised under maximum likelihood (ML) is sketched in the background, with ML bootstrap support values given for relationships recognised in the tree (sistergroups included/sistergroups excluded). Note the substantial difference between sistergroups and Loranthaceae and poor differentiation within the latter. An exception are the two *Loranthus* accessions, which show an unusual amount of unique indels and non-synonymous mutations (i.e. mutations that will change the aminoacid sequence)[2].

The data from the five loranth ***RPB2*** **accessions** recognise *Nuytsia* as clearly distinct as well, but lack any clear signal to even differentiate between the usually shorter branched Elytrantheae and the usually long-branched, clearly distinct Lorantheae (e.g. text-**Fig. 2**). *Taxillus* (core Lorantheae) is placed as sister to *Macrosolen* (Elytrantheae) but not *Loranthus*

---

[2] The distinctness of *Loranthus matR* sequences is highly conspicuous. Only 90-91% identity and max scores of > 2400 are found with *Amyema, Gaiadendron, Macrosolen* but also *Nestronia* (Santalaceae), *Minquartia* (Coulaceae), *Erythropetalum* (Erythropalaceae) and *Scorodocarpus* (Strombosiaceae) when MEGABLASTing the *Loranthus matR.* The overall scores are well beyond what is usually found for members of the same family or even angiosperm order for this gene region. For instance, *Nuytsia*, which is one of the most distinct Loranthaceae, has a max score of c. 3000 and 99% identity with *Gaiadendron, Macrosolen,* and the Santalaceae *Nestronia,* its identity with many other Santalales (100% coverage) and the remaining Loranthaceae is ≥ 98%! Identity as low as 90% in a coding mitochondrial gene as found for the two sequenced *Loranthus* specimens is a direct indication for pseudogeny or paralogy (e.g. *matR*-like gene re-localised to the nucleome), and demonstrates the naïve data use by Su et al. in contrast to our approach (see **Files S1, S2**).

(Lorantheae: Loranthinae; **Fig. S6-2**). So far, there is no reason to assume that *RPB2* has any potential for elucidating the deep relationships within the Loranthaceae.

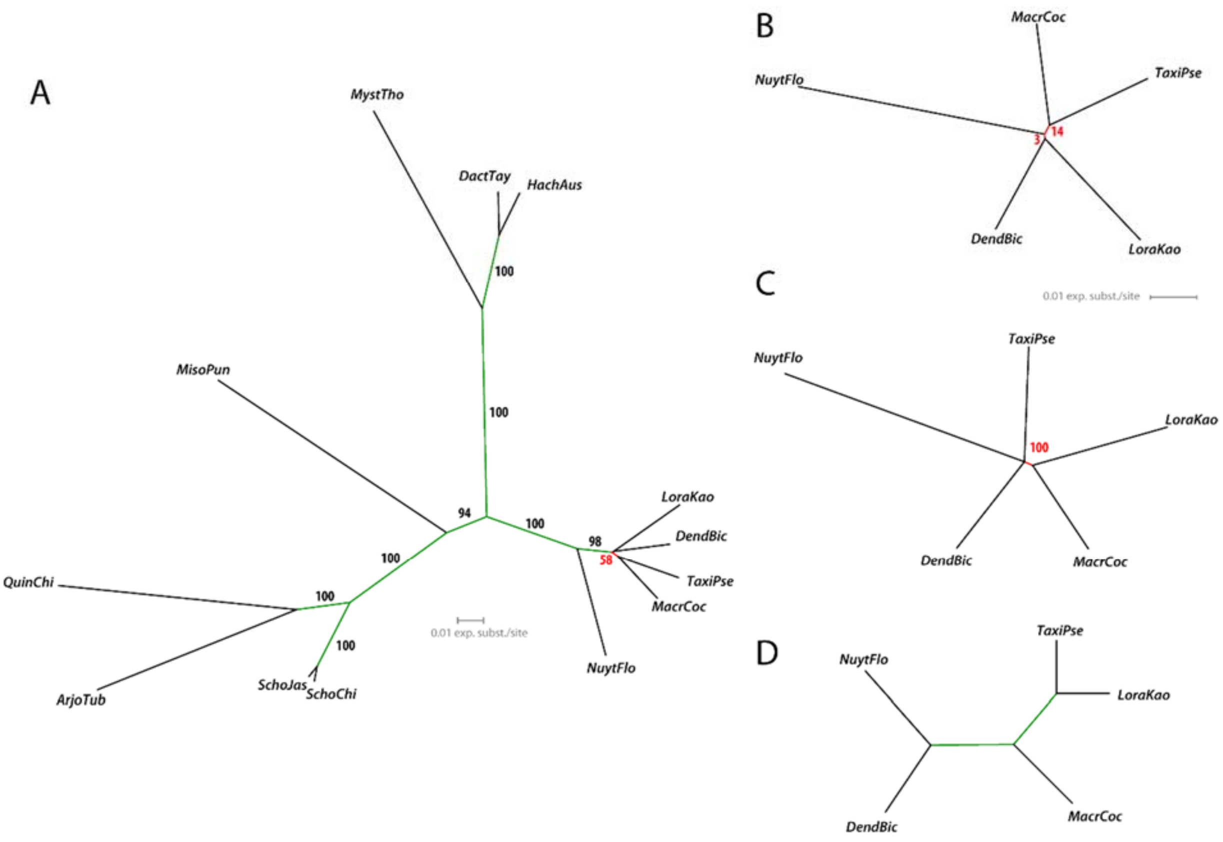

Figure S6-2: Information content of the additional nuclear gene (*RPB2*) included by Su et al., for relationships within Loranthaceae (data not included in our study due to its poor taxonomic coverage). Branches in line with Su et al.'s 7-gene tree in green, those in conflict with Su et al.'s up-to-7-gene tree in red. **A.** ML tree based on the data with sistergroups included. Note the similarity with the *matR* graph (**Fig. S6-1**): same relationships are inferred outside the Loranthaceae; and as it is the case for *matR,* differentiation within Loranthaceae is much lower. Interfamily relationships are essentially unresolved, but *Nuytsia* is recognised as very distinct (i.e. these data would enforce possible ingroup-outgroup LBA) **B.** ML tree based on only the Loranthaceae data. Note the complete lack of resolution. **C.** ML tree based on the same data than B, but with 1st, 2nd and 3rd codon position treated as a single partition (i.e. following the same substitution model). One, unambiguously supported, but wrong branch emerges (split between *Loranthus* + Elytrantheae *Macrosolen* and the other two Lorantheae + root parasite *Nuytsia*). This demonstrates that there is no discriminative signal in the *RPB2* to discern relationships within Loranthaceae, but the risk of a strong bias towards the 3rd codon position. **D.** Optimal graph for the 5-taxon problem in B and C, which would be in line with Su et al.'s tree.

The ***accD*** appears to be the most prospective gene region for the future when it comes to investigating relationships between the major Lorantheae lineages: The two Psittacanthinae, *Dendropemon* and *Tripodanthus*, distant relatives within their clade, are unambiguously supported as sisters with short terminal branches but a long root branch (**Fig. S6-3**), in contrast to the usual situation in this clade based on other gene regions or the concatenated data set which show short root branches (more or less support, but not free of conflict) with much longer terminal branches[3]. In the tree by Su et al., which does not include any data on *Phthirusa* since the authors do not accept Kuijt's (2011) resurrection of *Passovia* and regard

---

[3] Also this observation should have cautioned the authors against showing any MP results: how should a method that aims to minimise convergent unweighted changes along a tree be able to work with a data set where most of the signal is encoded as unique, parsimony-uninformative mutations?

Passovia pyrifolia as a valid representative of the genus *Phthirusa* (s.l.), *Tripodanthus* is moderately supported (BS_{ML} = 61, PP = 0.76) as the first diverging lineage of the Psittacanthinae and *Dendropemon* is deeply nested in this clade. We found the same based on our data set (text-**Figs 2, 3**). However, this is not always the case (e.g. *matK* nests *Tripodanthus* deeper in the Psittacanthinae and prefers *Aetanthus/Psittacanthus* as first diverging lineage). Furthermore, a very well-resolved topology is obtained for the Loranthaceae subtree (**Fig. R6-3**; all branches with BS_{ML} > 80, which is very high for a single-gene analysis). Unfortunately, it's entirely wrong from the perspective of Su et al.: the root parasitic grade is rejected (BS_{ML} < 5), instead *Gaiadendron* is resolved as sister of *Desmaria* and the two closely related Elytrantheae (*Alepis* and *Peraxilla*) with high BS support (BS_{ML} = 87 if sistergroups are included; **Fig. R6-3**).

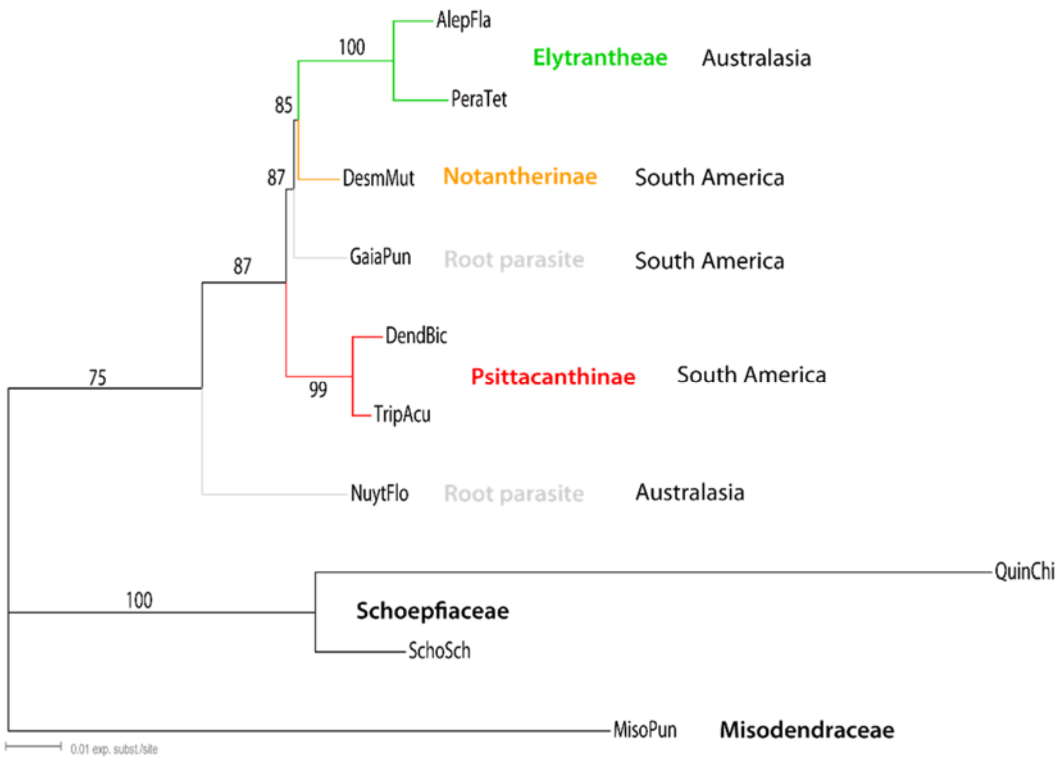

Figure S6-3: Information content of the addition plastid gene (*accD*) included by Su et al., for relationships within Loranthaceae (data not included in our study due to its poor taxonomic coverage). Shown is a ML tree based on the data, rooted with the sistergroups (no plastid data are available for the the direct sisterclade of Loranthaceae). Note the high(er) support typical for few-taxa trees, which however rejects with high support (BS_{ML} =87) the root parasitic grade, recognises the Psittacanthinae as first diverging group after *Nuytsia.* and associates the South American *Desmaria* with Elytrantheae (BS_{ML} = 85) which must be equally wrong than the association of *Notanthera* with this clade (see original comment *14,13* of reviewer #1).

If there is an enforcing effect for relationships within Loranthaceae, it lies in that these additional gene regions just add no discriminating signal at all for ingroup relationships (*matR, RPB2*) or for new conflicting splits (*accD*) not strongly supported by any other gene

partition so far and limited to a non-overlapping set of taxa, so the that the dominant topological alternative, mainly informed by signal from the *matK* gene (**Fig. S6-4**), prevails.

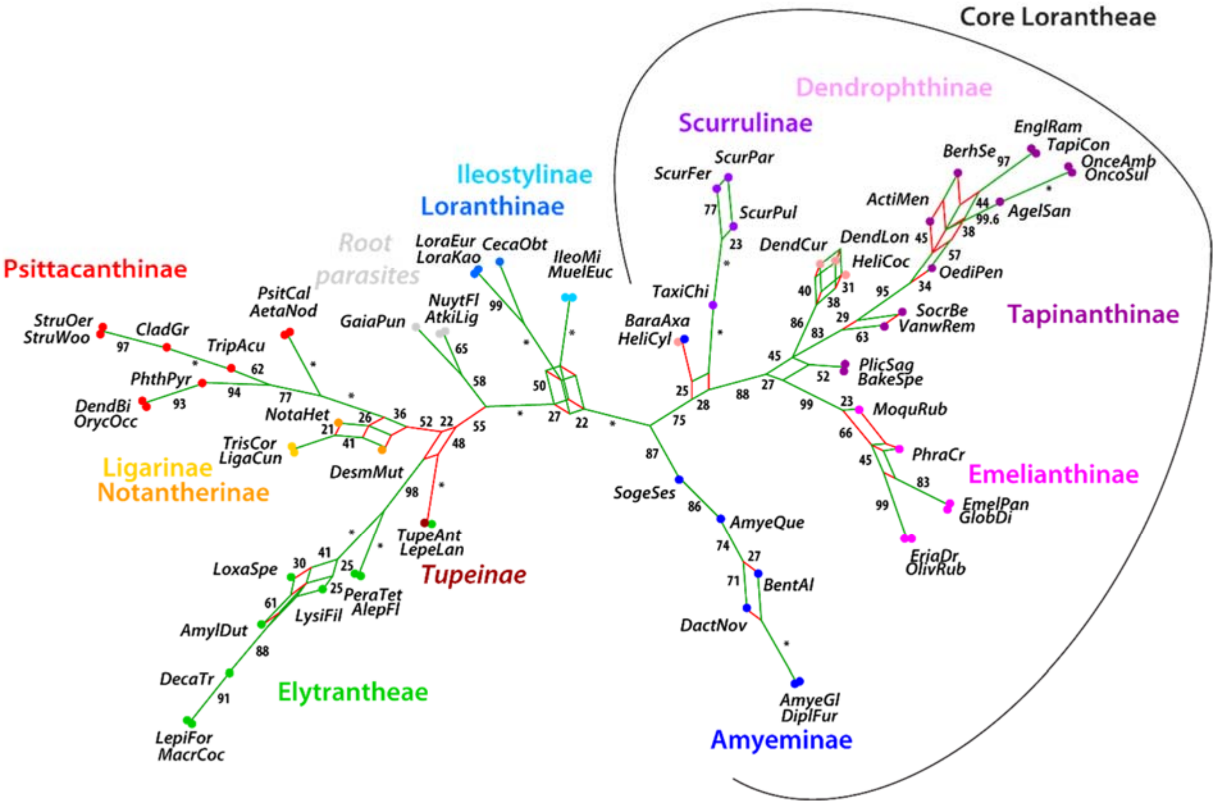

Figure S6-4: Maximum likelihood (ML) bootstrap (BS) support network using the Loranthaceae *matK* data and subset of the Su et al. matrix; trivial splits are collapsed and only splits are shown that were found in at least 20% of the bootstrap replicate trees. Edges also found in the bootstrap support network based on the concatenated 7-gene data (**Fig. S6-5**) in green, those not found (splits with BS support under ML, BS_{ML} < 20 based on the concatenated data) in red. Note the generally good match regarding most preferred and even alternative relationships (green boxes). Notable exceptions are the moderate but unchallenged support of a per-Gondwana epiphytic clade (BS_{ML} = 55, all alternative splits with BS_{ML} < 20, hence, no box visible) including all South American and (exclusively) Australasian aerial parasites (= tribe Psittacantheae according Nickrent et al. 2010) and supporting Vidal-Russell & Nickrent's (2007) original biogeographic scenario. The per-Gondwana epiphytic clade is not supported based on the concatenated data set because of conflicting signals from other gene regions. Note also the unambiguous support for a sisterrelationship between *Tupeia* and *Lepeostegeres*, which is a missing data artefact (only a very conservative part of the *matK* gene has been sequenced for the latter; and *Tupeia* generally shows least-derived sequences).

The conflicting signal then just add up to the length of the terminal branches and prevents unambiguous support (at least regarding the ML-BS support). Most other conflicting aspects (**File S1,** see next section) are simply not covered by any data in the additional gene regions. That such effects can eventually lead to an unambiguously supported but wrong branch in concatenated data sets is long known (e.g. Delsuc et al. 2005), and a principal, often ignored problem of analyses based on concatenated data. More important for the differences (compare **Fig. S6-5** to our **in-text Fig. 3**) is that the Su et al. (2015) matrix does not include the most variable, alignable, and relatively well-studied plastid region (being unalignable at the order level in Santalales), the noncoding trnL/LF region (see **Table R1**). That a noncoding plastid

region does not enforce the signal from the most variable coding plastid region but diminishes it (**File S1**), cannot be underestimated or ignored.[4]

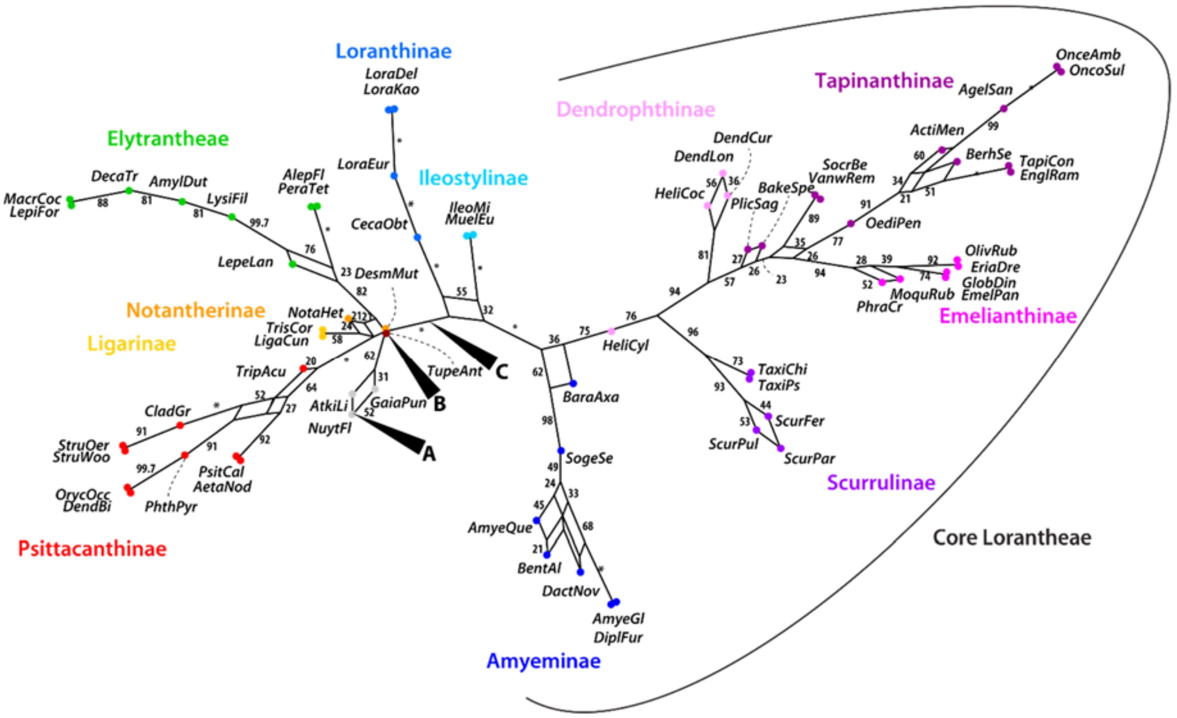

Figure S6-5: Maximum likelihood (ML) bootstrap (BS) support network using the Loranthaceae data and subset of the Su et al. matrix; trivial splits are collapsed and only splits are shown that were found in at least 20% of the bootstrap replicate trees. This reconstruction is equivalent to our text-**Fig. 3**; it shows the reason for non-unambiguous support of branches seen in Su et al.'s tree such as low-amplitude signal (just one alternative received BS > 20, tree-like portions) or ambiguous signal (more than one alternative received BS > 20, portions with boxes). The anonymous reviewers' preferred, outgroup-informed root is indicated (**A**), as well as the alternative roots informed by pollen morphology (**B**; as discussed in our manuscript) and by a molecular-clock approach (**C;** P. Kapli, G.W.Grimm; analysis done following Renner et al. 2008). Values at edges (corresponding to alternative, conflicting branches in trees) show BS support under ML; those marked by asterisks received

[4] Different partitioning schemes can also trigger topological differences or differences in support. The partition scheme used by Su et al. is not clear. On p. 492 they note "*For the ML and BI analyses, appropriate substitution models for each individual gene dataset were estimated using jModelTest v.2.1.3 (Posada, 2008, 2009)*" and on p. 493 they state that the "*The BI analyses were performed with MrBayes v.3.1.2 (Ronquist & Huelsenbeck, 2003) and best-fitting substitution models for the combined datasets were estimated using PartitionFinder v.1.1.1 (Lanfear & al., 2012) for each gene and codon position*". For the hypothesis testing, they note on the same page "*The ML tree for the 7-gene dataset, partitioned by gene, was estimated in Garli under a topological constraint that enforced monophyly for Balanophoraceae*". PartitionFinder usually decreases the number of partitions and with oligogene data can come up with artificial partitions that have little biological sense (pers. obs.), so it makes little sense to have used PartitionFinder and then say gene-wise paritions have been used. The finally used partition sets are not reported, it is also not clear whether BI and ML+BS with GARLI and ML-BS with RAxML. used the same partition schemes. RAxML does not allow to constrain the substitution model; to my opinion, for far the most datasets we work with these days at this level, pre-analysis testing and constraining the nucleotide substitution models is a waste of time. This may be the reason, why the programmers of RAxML did never bother to employ anything but the most general model for nucleotide sequence data: if a data set follow a more restricted model such as HKY, RAxML will optimise a model that approaches a HKY model.
Defining partitions for which the same model is used is more critical, at least in multigene datasets with tenths or hundreds of gene regions. The partition scheme I used may be rightfully criticised as over-partitioning (see arguments provided by Landfear et al. for employing PartitionFinder, although they devised the program for real multigene datasets, and it can be questioned whether this should also be used for 7-genes). Hence, as standard I always do a fully partitioned and unpartitioned analysis, which usually are not that different in result at these low hierarchical levels (I assume it could be different for an all-Santalales set; would have been interesting to see).

unambiguous support from Su et al.'s Loranthaceae subset (an analyses including sistergroups is included in the **OSA folder 'Su_et_al')**.

The often ignored issue of non-unambiguous support in oligogene trees

Single-gene trees and analyses may be severely misleading, because they are much more effected by data and signal issues than oligo-gene or multi-gene analyses. A wrong (mislabelled) sequence will misplace a taxon in a single-gene tree, it will inflict ambiguous support patterns in a few-genes tree, but the more sequences are added with correct signals, the faster the support will become unambiguous (usually much faster for PP than BS). Based on all Loranthaceae data I have seen (the one I harvested from gene banks, and the Su et al. subset of them), I am confident that one can and should concatenate all available data in order to infer a best-possible tree despite local oddities and possibly actual conflict in the concatenated gene regions. Despite the partly chaotic signal, some taxa are always drawn together; furthermore one should be aware that accessions with incompletely sequenced gene regions are vulnerable for misplacing depending on the signal amplitude provided by one gene region. The 18S data of Loranthaceae is a key witness regarding this: a tree can be inferred which in general places most taxa correctly, although the root branches of the according clades are very vague and poorly supported (**Fig. S6-6**; see **'Folder ML'** for the corresponding tree based on our data set). Fixations of mutations in the 18S underlie very strong structural constraints, the 18S rDNA has limited capacity to evolve, hence, does not easily accumulate discriminatory signal for tree inferences. In the case of oddly placed taxa (such as *Aetanthus*), inspection of the alignment shows that this is not necessarily linked to an obviously wrong sequence but simply that the relative part of the 18S rDNA is simply not sufficient to clarify the affinities of the accession as it covers an uninformative part of the gene. The closer we come to the leaves of the Tree of Life, the better parsimony-based or distance methods will perform with such data, whereas probabilistic methods have not enough signal to perform anymore: the tree space become too flat as expressed by the generally low level of bootstrap support under ML and the fact that a tree is selected that includes branches with little or no support at all. Concatenating such data with more variable and discriminative data (25S, *matK*) will effectively filter the few sites that contain phylogenetic signal from those that show just random, stochastic mutations.[5] If this signal is compatible, otherwise it will effectively eliminate any signal from the 18S data partition. As seen in our **File S1** there are poorly supported splits based on individual gene regions that are additive and finally result in a high support for the branch when those gene regions are concatenated. These are relatively unproblematic, no matter how high the final support eventually will be. Nevertheless, when concatenating data one should always keep in-sight the miscellaneous signal from the combined gene regions.

[5] Another general problem of 18S data are that most of these sequences are very old and usually include sequencing/editing artefacts. It was mainly used in the early days because it was easy to amplify with standard primers. Most of the sequencing/editing artefacts remained, because once an individual was sequenced for a genus in the early days of phylogenetics, there was (and is) no thrive to complement these old data by further sequences from the same species or other species of the genus. In case of more variable regions, sequencing and editing artefacts have little importance: they usually constitute purely random modifications, hence, cannot compete with the genuine signal in a sequence. In low-divergent regions they may eventually distort branch support as the match or exceed in number those sites that comprise genuine differentiation signal and add to the genuinely stochastic mutations concentrated in the regions coding for the central and terminal loops of the 18S rRNA.

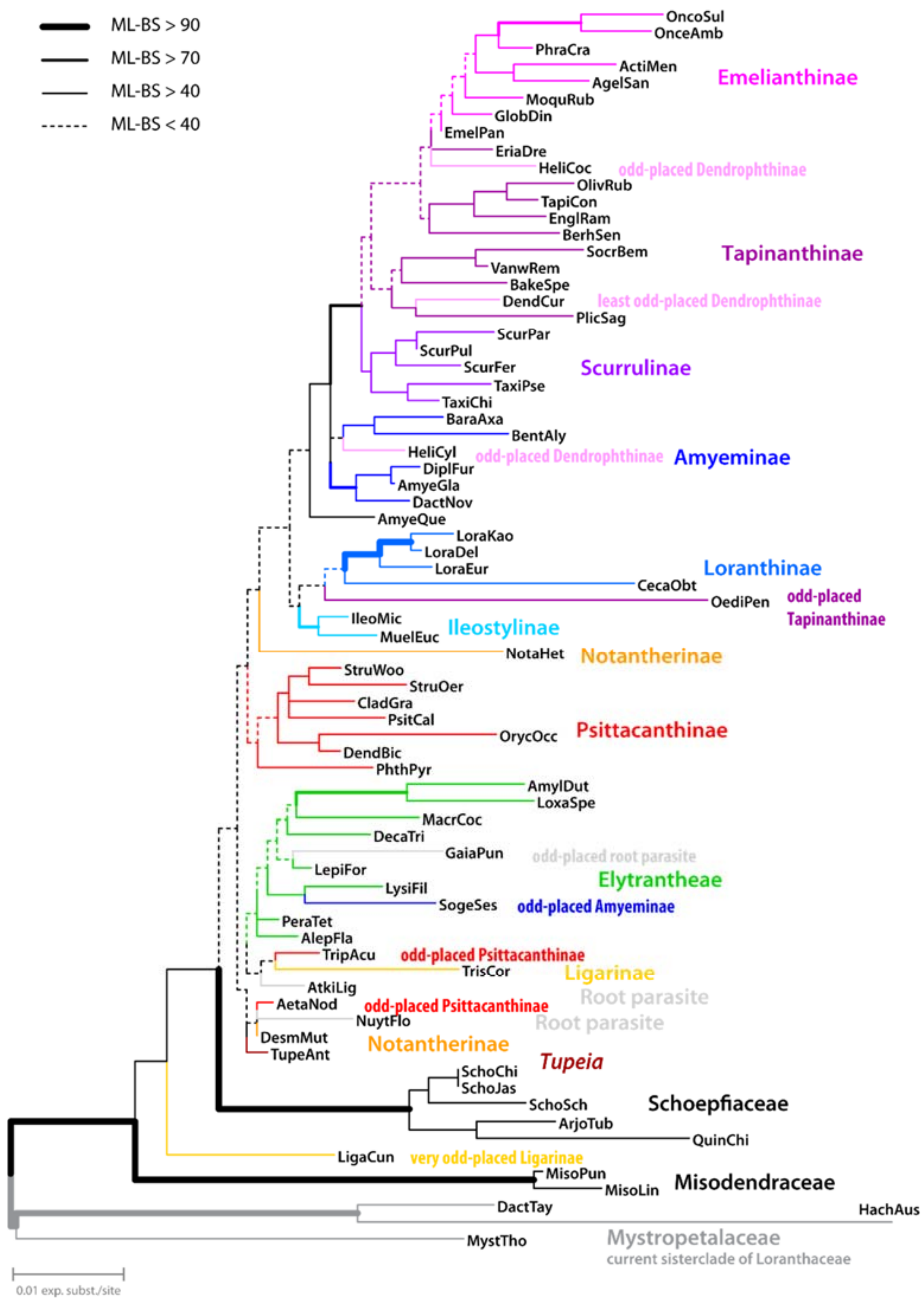

Figure S6-6: ML tree inferred from the 18S data of Loranthaceae and sister groups included in the Su et al. (2s015) matrix, rooted with the longest-branched subtree, the Mystropetalaceae[6]. Note that most branches in the tree are poorly supported, which directly relates to the generally high conservation (and usual little phylogenetic use) of the 18S rRNA genes in plants: mutations are strongly restricted in the stem regions, and, hence, accumulate in the terminal loop regions. Mutations in the stem regions are not rarely compensated, i.e. mutations seen at certain sites in the alignment may be linked. Nevertheless, the tree can show much: (i) despite lacking

---

[6] An optimal rooting is not possible as there is a moderate BS support (BS_{ML} = 51) supporting Misodendraceae + *Ligaria* + Mystropetalaceae | Schoepfiaceae + all other Loranthaceae split, but no support for a Misodendraceae + Schoepfiaceae | Mystropetalaceae + Loranthaceae split, the latter unambiguously supported in Su et al.'s up-to-7-gene tree. Re-rooting the tree e.g. with Schoepfiaceae would lead to a moderately supported Misodendraceae-*Ligaria*-Mystropetalaceae clade sister to Loranthaceae.

support, members of the major clades are generally grouped; (ii) long terminal branches, short roots → fast ancient radiation and/or high percentage of stochastic mutations, note the odd-placed accessions and relatively long branches with (very) low support; (iii) *Tupeia* is genetically close(st) to the Loranthaceae root, the hypothetical common ancestor, at least closer than all root parasites; and (iv) the association of *Aetanthus* with *Desmaria* in text-**Figs 2, 3** is a missing data artefact: the sequenced part of the 18S fails to resolve the affinity of the genus with *Psittacanthus* as is represents a very conserved part with essentially no discriminative signal.

The root parasitic grade, a must-be (according anonymous reviewer #1), generally accepted (Nickrent et al. 2010), but poorly supported—Problematic are branches where the support from the individual gene regions is not cumulative or generally low (**Table S6-1**), which in particular applies to the root parasitic grade, where interesting observations can be made. Only one of the three splits (branches, internodes) defining this grade received a near unambiguous support (PP = 0.99) from Bayesian analysis, in the other two cases the BS supports and PP are indicative for non-consistent signal (BS < 50, PP << 0.95; PP are based on the full data matrix, BS supports are based on artificial, resampled matrices). First, the elimination of distantly related (topologically speaking) Santalales from the data set, leads to an increase in bootstrap support for the root parasitic grade branches (BS_{ML} < 50/55/<50 to BS_{ML} = 100/62/60). This is a first sign that the root parasitic grade is a branching artefact: ingroup-outgroup LBA can be avoided to some degree by using larger outgroup samples. By adding other Santalales, i.e. providing a much more comprehensive outgroup sample, we reduce ingroup-outgroup LBA between *Nuytsia* and the sistergroups of the Loranthaceae, hence, the false unambiguous support for the according primary split tumbles substantially. It tumbles less under Bayesian inference than ML-BS (very rarely a branch with an unambiguous ML-BS support has a PP << 1.0) because PP are overestimating and BS underestimating, the latter replicates will eventually include matrices where the sites inflicting the LBA play a lesser role. The MCMC chain however does not have this corrective, nevertheless the non-unambiguous PP for two of the three branches in Su et al.'s tree show that there is some signal issue.[7]

Another evidence for a branching artefact is that the root parasitic grade collapses when using the aminoacid sequences instead of the nucleotide sequences, whereas *Nuytsia*, also clearly distinct in its (putative) aminoacid sequences and the only Loranthaceae with full coverage for this data set, remains firmly as sister to all other Loranthaceae (Su et al., fig. S7).

---

[7] Another reason not involving ingroup-outgroup branching artefacts would be that Su et al.'s inferences did not explore deep enough the tree space, hence, failed to provide sufficient support. But since they used GARLI, probably the programme with the highest accuracy (A. Stamatakis, pers. comm., 2010), to infer the ML tree and RAxML for bootstrapping (also used here, generating high support for the subsets), I doubt that this was the case. It may also be due to different partitions schemes used, depending how the 3rd codon position was treated. Su et al note to have used jModeltest and PartitionFinder, but don't document the results of these programmes nor the finally used partition schemes. Support for the primary split at least increased when fast evolving sites were excluded (Su et al., fig. S6); this is unsurprising when looking at the alignments and comparing the *Nuytsia* sequences directly to those of other Loranthaceae. However, the root parasitic grade dissolved completely and the backbone support collapsed when the codons were translated into aminoacid sequences and the two ribosomal DNA regions subsequently excluded from the analysis (Su et al., fig. S7; see **Table S6-1**). I, on the other hand, found similar supports for the 7-gene subset for an completely unpartitioned analyses (**Table S6-1**), which indicates that the subset is fairly stable against different partition schemes.

Table S6-1: Ambiguous support for the root parasitic grade (PREF = split seen in Su et al.'s and reviewer #1's preferred tree; ALT = topological alternative indicated by Su et al.'s filtered and aminoacid trees, or with high support from a single gene; PTD = fully partitioned analyses, all codon positions and gene treated as data partitions; UP = unpartitioned analyses: one substitution model is optimised for the entire data set). "?" indicates values that cannot be estimated from the provided documentation (e.g. a split competing with a preferred split with a BS_{ML} < 50, PP < 0.5 (i.e. 0–49/0–0.49) can have a BS_{ML}/PP of 0–100 and 0–1.0)

	Split/clade	Su et al., fig 1B		—, fig. S6	—, fig. S7[b]	Re-analysis of subset of Su et al. (see **OSA 'folder Su_et_al'**) Including sistergroups (SG)					Excluding SG				
		BS_{ML}	PP	BS_{ML}	BS_{ML}	7-gene PTD/UP	18S	25S	*rbcL* PTD	*matK* PTD	7-gene PTD	18S	25S	*rbcL* PTD	*matK* PTD
PREF	SG + *Nuytsia* | remaining Loranthaceae	<50	0.65	94	99	100/100	<20	47	97	100	N/A	N/A	N/A	N/A	N/A
PREF	SG + *Nuytsia* + *Atkinsonia* | rem. Loranthaceae	53	0.99	54	?	62/53	<20	<20	21	75	52	<20	<20	<20	65
ALT	Gaiadendreae clade	≤47	≤0.01	≤46	?	<20/<20	<20	46	<20	<20	<20	<20	48	<20	<20
PREF	Aerial parasite clade	<50	0.52	49	?	60/54	<20	<20	<20	66	62	<20	<20	<20	58
PREF	*Desmaria* (S. Am.) sister to *Tupeia* (NZ)	<50	<0.5	?	<20	<20/<20	<20	<20	<16[$]	0[$]	<20	<20	<20	<20	<20
ALT	SAAP clade including *Desmaria*	?	?	40	?	<20/<20	<20	<20	<20	51	<20	<20	<20	<20	52
PREF	SAAP clade excluding *Desmaria*	<50	0.91	?	?	22/<20	<20	<20	35	36	21	<20	<20	<20	36
PREF	*Notanthera* + Psittacanthinae	<50	0.75	?	?	21/21	<20	25	<20	<20	<20	<20	30	<20	41
ALT	Ligarinae + Notantherinae	?	<0.1	35	33[§]	<20/<20	<20	<20	<20	26	<20	<20	<20	<20	26
PREF	Ligarinae	<50	0.96	?	78	65/44	<20	<20	N/A	62	58	20	<20	N/A	62
ALT	*Notanthera* sister to *Tristerix*	?	≤0.04	45	<22	<20/<20	<20	<20	N/A	<20	<20	<20	<20	N/A	<20
PREF	*Tripodanthus* sister to rem. Psittacanthinae	61	0.76	45	?	67/68	30	62	71	<20	64	29	62	84	<20
ALT	*Psittacanthus (+Aetanthus)* sister to rem. Psittacanthinae	≤39	≤0.24	?	52	<20/<20	<20	<20	N/A	71	20	<20	<20	N/A	77

* Fast evolving sites excluded

† Aminoacid tree (i.e. not including 18S and 25S data)

§ This clade also includes *Aetanthus* (Psittacanthinae) and is placed with very low support as sister to *Taxillus* (Lorantheae: Scurrulinae)

$ The *rbcL* data produces BS_{ML} = 84 for a sister relationship between *Tupeia* and *Moquinella* (core Lorantheae); the *matK* for a sister relationship between *Tupeia* and *Lepeostegeres* (Elytrantheae)

When the sistergroups are removed, the support does not increase, which shows that inclusion/exclusion of sistergroups has little effect on branch supports. However, by removing the sistergroups, it should have become easier to sort out the basic relationships, because we now infer a tree solely for the Loranthaceae and not a tree that also tries to connect the sistergroups to the Loranthaceae subtree. So why is the support still not unambiguous? Because out of the better-sampled gene regions (see preceding section for the signal from the poorly sample genes), it is only the *matK* that supports the splits forming the grade (**Table S6-1**), the other genes only recognise *Nuytsia* as distinct (which is a trivial split, when only Loranthaceae are included). And despite the usually strong and prevailing signal from the *matK* (see **Fig. S6-1**), the little data on *accD*, another plastid gene that rejects a split between root and aerial parasites (**Fig. S6-3; Table S6-1),** are apparently sufficient to decrease the support for two of the three branches defining the clade, although no other gene region produces a markedly conflicting signal and has little idea where to put *Atkinsonia* or *Gaiadendron.* The competing support for a Gaiadendreae clade from the 25S is partly compatible with the grade, since this is just a rearrangement within essentially the same subtree.

The (South) American aerial-parasitic (SAAP) clade—Su et al.'s tree shows a clade comprising all aerial parasitic taxa from (South) America except for *Desmaria*. Also here the BS support of the root branch is low ($BS_{ML} < 50$ for the full data, $BS_{ML} = 40$ for the aminoacid dataset and including *Desmaria*), but nearly unambiguous when using BI (PP = 0.99). Let's first concentrate on *Desmaria*. As mentioned above *accD* sees in this taxon a sister of the Australasian Elytrantheae, and it is more or less placed accordingly in Su et al.'s tree outside the SAAP clade in a poorly supported (BS < 50, PP = 0.52) clade with *Tupeia*. Ironically, it is the *matK* in Su et al.'s data set that decreased the support, the latter favouring a SAAP clade that includes *Desmaria*. So the funny thing here is: one well sampled plastid gene, *matK,* wants to place *Desmaria* with the SAAPs, another *accD,* poorly sampled, prefers a conflicting placement (**Fig. S6-3**). The remainder of the genes has simply no idea where to place *Desmaria*. The BS support and PP to a lesser degree reflect this uncertainty: neither the placement of *Desmaria* with *Tupeia* nor the placement of *Desmaria* with the SAAPs receives any support based on the 7-gene data. A bootstrap support network (**Fig. S6-5**) fully captures this uncertainty, but the tree is completely uninformative in this respect.

If we go deeper into the SAAP subtree, we get another lesson on how PP can react differently to conflict or signal amplitude than BS, and why all PP not approaching 1.0 (in oligo-gene datasets) may be indicative for conflict or data issues. The SAAP clade is unambiguously supported by PP although there is not a single gene region that provides any strong support for a clade including *Notanthera,* the Ligarinae, and Psittacanthinae; just a slight preference of the better-sampled plastid genes *matK* and *rbcL*, which agree on this aspect, but disagree on others regarding the same group of taxa. To decide whether the SAAP clade is valid or not should be easy to test by adding *accD* data for the critical taxa (*Notanthera,* the Ligarinae, *Phthirusa, Tripodanthus*), but such data are not available. The PP = 0.75 for the *Notanthera* + Psittacanthinae relationship and PP = 0.96 for the Ligarinae is clearly overconfident, with

only a single of the seven genes supporting these branches (**Table S6-1**). The latter, a Ligarinae clade, is further supported by the aminoacid tree, which naturally uses a more conservative signal, so we have little reason at this point to doubt it. But the aminoacid tree fails to resolve *Notanthera* as sister of the Psittacanthinae, as does *matK* which prefers to associate *Notanthera* with *Desmaria* and the Ligarinae (Su et al., fig. S7).

Into the Psittacanthinae, we run into a strong and illuminating signal conflict between *matK* and the rest of the genes regarding the placement of *Tripodanthus*. The nuclear 18S, 25S and the plastid *rbcL* team up and overrule a competing *matK* signal, but the BS and PP remain below the usually enforced thresholds of high support (BS > 70, PP > 0.95) for the concatenated data. Having analysed the signal from the different concatenated gene regions, we can see that the ambiguous support in this case is not big deal from a phylogenetic point of view: *matK* simply would like to nest *Tripodanthus* deeper into the subtree it already belongs to, but this messes up the whole subtree (note the boxes in **Fig. S6-4**). With the knowledge that *rbcL* and *matK* are both plastid genes, we can assume that the *matK* signal is misleading (a branching artefact) and *Tripodanthus* still well placed as sister to all other Psittacanthinae.[8] On the other hand, we also see that it takes a strong signal from three gene regions to outcompete a partly conflicting signal from the *matK* gene. This exemplifies that the Su et al.'s Loranthaceae subtree should not be viewed as the result of a seven-gene analysis, but as a *matK* topology enforced or weakened by supporting or conflicting signals from six more genes (compare **Figs S6-4** and **S6-5**).

***Tupeia* and the Elytrantheae—**Given that they are still relatively geographically close, one could have expected that at least the plastid gene regions produce support for a *Tupeia*-Elytrantheae clade, but this is not the case (**Tables S6-1, S6-2**). The *rbcL* data is unfortunately victim of a fundamental signal issue. Being substantially more conserved than *matK,* the length of the sequenced part of *rbcL* can be crucial for a proper placement of a taxon. For the Su et al. data, the insufficiency to place *Tupeia* is partly due to data gaps: the *rbcL* promotes an unlikely sister relationship between *Tupeia* and one of the Emelianthinae (core Lorantheae), and places this two-taxon clade with diffuse support in the vicinity of the Elytrantheae, but also *Desmaria* and the root parasite *Gaiadendron*[9]. The latter is an interesting parallel to the *accD* tree. The *matK* on the other hand is 100%-sure that *Tupeia* must be the sister of *Lepeostegeres*, and places them in a sisterclade to the remaining Elytrantheae, but with low support (**Table S6-2**)). For *Lepeostegeres* only a second gene region has been sequenced, the 25S, which places the taxon firmly within Elytrantheae Clade B according Vidal-Russell and Nickrent (2008).

---

[8] In fact, a glimpse on Su et al.'s, fig. 1b and fig. S7, shows that the nesting is less an issue of the *matK* data of *Tripodanthus* but the *matK* data of *Aetanthus* and *Psittacanthus.* The *matK* data recognises them as sisters, but this is not the case when the nucleotide sequences are translated into aminoacid sequences. So the data sees *Aetanthus* as sister of *Psittacanthus,* the latter clearly a Psittacanthinae but not the former due to some miscellaneous signal (which explains the odd placement in the aminoacid tree), and resolves this incompatibility by placing both as sister to the remainder of the clade and then has to find a new optimal position for *Tripodanthus.*

[9] The diffuse support is the direct consequence of the fact that both taxa have different affinities regarding minor signals in their *rbcL* sequences (*Tupeia* having a unique, but not very derived *rbcL,* the misplaced Emelianthinae showing a indiscriminative part with very few mutations that may be typical for Lorantheae once a better taxon sampling is available.

Table S6-2: Ambiguous support *Tupeia* and the Elytrantheae (PTD = fully partitioned analyses, all codon positions and gene treated as data partitions; UP = unpartitioned analyses: one substitution model is optimised for the entire data set)

Split/clade		Su et al., fig 1B		—, fig. S6	—, fig. S7[b]	Re-analysis of subset of Su et al. (see **OSA 'folder Su_et_al'**) Including sistergroups (SG)					Excluding SG				
		BS_{ML}	PP	BS_{ML}	BS_{ML}	7-gene PTD/UP	18S	25S	*rbcL* PTD	*matK* PTD	7-gene PTD	18S	25S	*rbcL* PTD	*matK* PTD
PREF	*Desmaria*-*Tupeia*-Elytrantheae clade	<50	0.52	?	?	<20/<20	<20	<20	26[$]	<20	<20	<20	<20	<20	<20
PREF	*Tupeia* sister to *Desmaria*	<50	<0.5	<5	<20	<20/<20	<20	<20	<16	0	<20	<20	<20	<20	0
ALT	*Tupeia* sister to *Lepeostegeres*	<26	<0.43	94	82	<20/<20	N/A	<20	N/A	100	<20	<20	<20	<20	100
ALT	*Tupeia* sister to *Moquinella*	?	?	?	?	<20/<20	<20	<20	84	0	<20	<20	<20	80	0
ALT	*Tupeia*-Elytrantheae clade	?	?	75	?	<20/<20	<20	<20	<20[#]	45	<20	<20	<20	<20	48
ALT	*Tupeia* + *Lepeostegeres* + Psittacanthinae	?	?	<25	35	<20/<20	N/A	<20	N/A	<20	<20	<20	<20	<20	<20
PREF	Elytrantheae	74	0.57	<5	?	78/82	<20	70	<20	<1	82	<20	75	<20	98
PREF	*Lepeostegeres* + Clade B subclade	67	0.57	<5	<18	73/76	N/A	76	N/A	<1	76	N/A	80	N/A	N/A
ALT	Clade A + Clade B	<26	<0.43	95	39	27/23	[s.a.]	<20	[s.a.]	99	23	[s.a.]	<20	[s.a.]	[s.a.]
PREF	Clade A (*Alepis* + *Peraxilla*)						<20	80	N/A	100	100	<20	82	N/A	100
PREF	Clade B (remainder)						<20	<20[§]	36[#]	100	99.7	<20	<20	47	100

* Fast evolving sites excluded

[†] Aminoacid tree (i.e. not including 18S and 25S data)

[§] *Lepeostegeres* best-supported as sister of *Decaisnea* (BS_{ML} = 51)

[$] Includes *Gaiadendron* and *Moquinella* (core Loranthaceae)

[#] A *Tupeia*-Elytrantheae Clade B (represented by two taxa) clade would receive BS_{ML} = 42

In the concatenated data set these partly conflicting, and strong signals, lead to a sisterrelationship of *Lepeostegeres* with Clade B (not supported by any of the gene regions) and the complete failure to place *Tupeia* (but see also next section); because this is the topology that has the least conflict with both the *matK*- vs the 25S-preferred topologies. The fact that the support for the *Tupeia-Lepeostegeres* sisterrelationship is higher for the tree excluding the fast-evolving sites and the aminoacid tree indicates that it is primarily the signal from the *matK* data that is problematic.

Going to the alignment, one can see that the sequenced part of the *matK* that is available for *Lepeostegeres* is not the most informative one. While the relatively divergent 25S sequence firmly places the taxon with Elytrantheae B, the *matK* draws a somewhat dubious connection to *Tupeia* as anonymous reviewer #1 nicely pointed out in both review rounds: "*… Again, it's* [Lepeostegeres] *position* [in our text-Fig. 2] *is clearly wrong (as sister to Tupeia). It was present in Elytrantheae, as supported by floral morphology, in Su et al. (2015). "* So the situation we face with *Tupeia/Lepeostegeres* are misleading *matK* sequences, equally misleading *rbcL* sequences (the sister relationship with *Moquiniella* is also "cleary wrong"), leaving us with the 25S to correctly place *Lepeostegeres*, which is not enough for high support.

And no gene holds any real clue what to do with *Tupeia,* despite of the fact that the latter is one of the best-represented Loranthaceae in Su et al.'s matrix (and ours). I find this most interesting, and a proper sampled *Tupeia* would be crucial when it comes to address deep relationships in Loranthaceae. Unfortunately there are no *matR* and *accD* sequences of *Tupeia* and the isolated South American taxa.

Basic relationships within the Lorantheae—From Su et al.'s tree, the basic relationships within the Lorantheae subtree are relatively clear: the most-distinct (within this clade) Loranthinae are placed as sister to Ileostylinae and the core Lorantheae (Clade I+J according Vidal-Russell & Nickrent 2008). Withinthe core Lorantheae, we see a split between the Amyeminae and the remaining subtribes (Clade J). The support of the defining branches appears to be moderately to very high ($BS_{ML} \geq 60$, $PP \geq 0.79$). The lower supports ($BS_{ML} < 80$, $PP < 0.95$) are inconspicuous as they are associated with relatively short branches. The same hold for the decreasing supports when the fast-evolving sites are eliminated or the amino-acid, we are now approaching the leaves, and genes and proteins are not likely to resolve shallow branches with the same strength than the deeper ones. However, the aminoacid tree of Su et al. does produce a poorly supported sister relationship between Loranthinae and Ileostylinae ($BS_{ML} = 24$) and moves *Baranthus,* the first diverging Amyeminae according to Su et al.'s fig. 1B, away from its clade as sister to *Helixanthera cylindrica* ($BS_{ML} = 48$), the first branching lineage in Clade J. Analogously, we can note a decrease in BS_{ML} for the preferred splits, when the Su et al. subset is analysed using a single data partition for the entire data. This is directly indicative for signal issues with the used data.

Table S6-3: Differential support for basic relationships within the Lorantheae (PTD = fully partitioned analyses, all codon positions and gene treated as data partitions; UP = unpartitioned analyses: one substitution model is optimised for the entire data set)

Split/clade		Su et al., fig 1B		—, fig. S6	—, fig. S7[b]	Re-analysis of subset of Su et al. (see **OSA 'folder Su_et_al'**) Including sistergroups (SG)					Excluding SG				
		BS_{ML}	PP	BS_{ML}	BS_{ML}	7-gene PTD/UP	18S	25S	*rbcL* PTD	*matK* PTD	7-gene PTD	18S	25S	*rbcL* PTD	*matK* PTD
PREF	Lorantheae	>80	>0.95	100	66	100/100	<20	77	88	100	100	30[$]	84	83	100
PREF	Ileostylinae + core Lorantheae	60	0.79	67	?	71/49	<20	88	31	<20	55	<20	89	31	22
ALT	Loranthinae + Ileostylinae	<40	<0.2	<33	24	<20/38	<20	<12	49	46	32	<20	<11	50	50
ALT	Loranthinae + core Lorantheae	<40	<0.2	<33	?	<20/<20	<20	<20	<20	35	<20	<20	<11	<20	27
PREF	Loranthinae	>80	>0.95	100	92	100/100	37	86	100	100	100	37	87	99.8	100
PREF	Ileostylinae	>80	>0.95	100	99	100/100	73	93	99	100	100	71	93	99	100
PREF	Core Lorantheae	>80	>0.95	100	91	100/100	36	79	99	100	100	34	78	99	100
ALT	*Baratanthus* sister to *Helix. cyl.*	<30	<0.1	<46	48	<20/27	<20	<20	N/A	53	<20	<20	<20	N/A	61
PREF	Amyeminae	66	0.87	38	?	59/59	<20	75	N/A	<20	62	<20	74	N/A	<20
PREF	All but *Barathanthus*	>80	>0.95	66	39?	95/96	<20	74	N/A	84	98	<20	80	N/A	87
PREF	All other subtribes	70	0.89	54	53?	75/67	<20[§]	70	66	<20	75	88[§]	66	81	<20
ALT	All other subtribes + *Baratanthus*	<30	<0.1	<46	?	<20/<20	<20	<20	<20	74	36	<12	<20	N/A	75
PREF	All but *Helixanthera cyl.*	81	1.00	73	?	77/83	82	<20	N/A	<20	76	<20	<20	N/A	28

* Fast evolving sites excluded

[†] Aminoacid tree (i.e. not including 18S and 25S data)

[§] *Helixanthera cylindrica* groups with some of the Amyeminae

[$] Does not include *Soegerianthe*

Our **File S1**, which compiles the results of our first level gene-jackknifing analyses using our genus-consensus sequence data matrix and competing support patterns from earlier studies, shows that the situation is really not that crystal-clear and that an alternative topology would be e.g. a Loranthinae+Ileostylinae clade sister to the remainder of the Lorantheae. It also shows that in our data some gene regions severely misplaced the one or other core Lorantheae; and the same holds for the matrix used by Su et al. despite the convincing support values in the 7-gene tree (**Table S6-3**). Minor issues are that the 18S fragment sequenced for *Soegerianthe* is insufficient to place the taxon, the same holds for *Oedina,* which is placed with *Loranthus* (**Fig. S6-6**). As already pointed above, the *rbcL* fragment of *Moquiniella* links it with *Tupeia.* More interesting is why *Baratanthus* is placed as sister to the other Amyeminae. The 18S has little potency to resolve affinities of the Lorantheae beyond the trivial, the 25S supports the inclusion of *Baratanthus* in the Amyeminae (a sure thing from a morphological point of view, see Nickrent et al., 2010), but the otherwise informative *matK* does not with an equal support. Providing much more signal for this part of the Loranthaceae subtree and preferring a topology less in conflict with the overall signal, i.e. the recognition of Amyeminae being apart from the other core Lorantheae, the 25S signal outcompetes the misleading *matK* signal. Since the critical specimens are not sequenced for any other gene region, a high support is produced for the likely correct relationship, the *matK* conflict is nevertheless seen in the bootstrap consensus network (**Fig. S6-5**).

But the data does not directly indicate e.g. that *Baranthus* is the first diverging genus in the Amyeminae as shown in the tree: it cannot be placed deeper within the Amyeminae because it has the "wrong" *matK* sequence, whereas all other Amyeminae have a *matK* sequence that fits their 25S sequences. Similarly, *Helixanthera cylindrica* is only placed as sister to the remaining members of Clade J because it does not have the proper 18S sequence, and a *matK* that links it to *Baranthus,* which otherwise is not a member of Clade J. The partial conflict is resolved by placing both taxa as sisters to the remainder of their clades. In doing so, both the Amyeminae as well as the remaining core Lorantheae root maybe misinformed.

Regarding the primary splits the non-unambiguous support is due to a similar conflict: the 18S has like usual no idea what to do here, the 25S is confident that Ileostylinae are sisters of the core Lorantheae, whereas the worse sampled *rbcL* can't decide whether to go for this or the alternative of a Ileostylinae+Loranthinae clade. Since the *matK* does only half-heartedly support the second *rbcL* alternative and favours the third remaining possibility (Loranthinae sister to core Lorantheae), the concatenated data values the 25S signal as superior (it's the only gene region producing a nearly unambiguous signal), and the 25S preferred topology is taken for the 7-gene tree, but without unambiguous support. These examples show that one should be very careful with any current branch in the Loranthaceae tree that has no unambiguous support or does not find ample low support from more than a single gene region.

The outgroup-inferred root, the signals of the concatenated gene regions

With respect to the high dependency of topologies on gene sampling as outlined in the last two sections, relying exclusively on extremely-divergent-outgroup-inferred roots in the case of Loranthaceae is naïve. In fact, the 25S tree topology, branch-lengths, and supports (**Fig. S6-7**) are stand-alone indicative for a branching artefact that joins *Nuytsia* with the Mystropetalaceae (such as LBA), whereas the Misodendraceae-Schoepfiaceae-informed root (all taxa that are still distinct, but not as much as the Mystropetalaceae) would be in better agreement with the remaining data. Either way, *Tupeia* is close – in absolute and topological terms – to both potential roots, the one indicated by the Mystropetalaceae and the one indicated by Misdendraceae + Schoepfiaceae; and this is something that can be seen in all single-gene and combined-data trees. Hardly a co-incidence, but supporting the hypothesis that *Tupeia* is a very early isolated, conservatively evolving genus within the Loranthaceae, as reflected by its pollen; independent whether it is sister to all other Loranthaceae or only most of them (as sketched by Vidal-Russell & Nickrent 2007), it is – by all evidence – the modern genus that is closest to the common ancestor of all Loranthaceae (extinct and extant), i.e. the Loranthaceae root. The *Nuytsia* root on the other hand, is most likely a branching artefact because we lack outgroups that are genetically close enough to the Loranthaceae to inform a sensible ingroup root, and/or gene regions not affected by branching and data artefacts with the capacity to resolve the initial splits within the Loranthaceae while at the same time resolving the relationship of the Loranthaceae to their long-branching sister clades.

Let us dissect the Su et al. data on Loranthaceae and their sister groups further: the 18S, one of the most conservative gene regions sequenced for angiosperms and the Loranthaceae, finely separates ingroup and sistergroups, with one exception: *Ligaria cuneifolia,* which has quite a distinct sequence, an unique pollen, but nevertheless is clearly a Loranthaceae. This shows that even at the interfamily level, notably conservative nuclear genes (18S sequences can be very similar between families that diverged in the late Cretaceous such as Juglandaceae and Myricaceae) struggle finding a clear signal; a direct indication for fast ancient radiation accompanied by poor signal sorting. In properly sorted, long-evolving or fast-evolving groups, such as foraminifers as an very extreme example, you can use nearly each mutation of the 18S rDNA to trace the clades in a combined tree (pers. obs.; but just take the time on your favourite data and look for yourself), because of the 18S' low mutation and fixation rate. But even this much more conserved nuclear ribosomal gene region places the Misodendraceae and Schoepfiaceae as a grade in between the Loranthaceae and their purported sister clade, the Mystropetalaceae, with moderate support (**Fig. S6-6**). Thus, it also rejects the preferred topology[10], but differently than the 25S. This is most challenging for the Su et al. tree since 18S and 25S are part of the same cistron (reading frame), i.e. effectively represent the same locus and should reflect the same genealogy at such a level. But they fail to do so. A simple explanation would be that the signal of the initial divergences has been partly overwritten by later radiation or severe bottleneck situations, hence, lost. As a result, it

[10] For those unfamiliar with the concept of re-rooting: if the 18S tree is re-rooted with the Schoefiaceae, the Misodendraceae become sister of the Mystropetalceae; if re-rooted with Misodendraceae, the Schoepfiaceae become sister of Loranthaceae. A rooting with both Misodendraceae and Schoepfiaceae is not possible, because an according split is not seen in the tree (nor supported by the BS analysis)

may be impossible to infer a meaningful outgroup-based root for the Loranthaceae using 18S and 25S rDNA data.

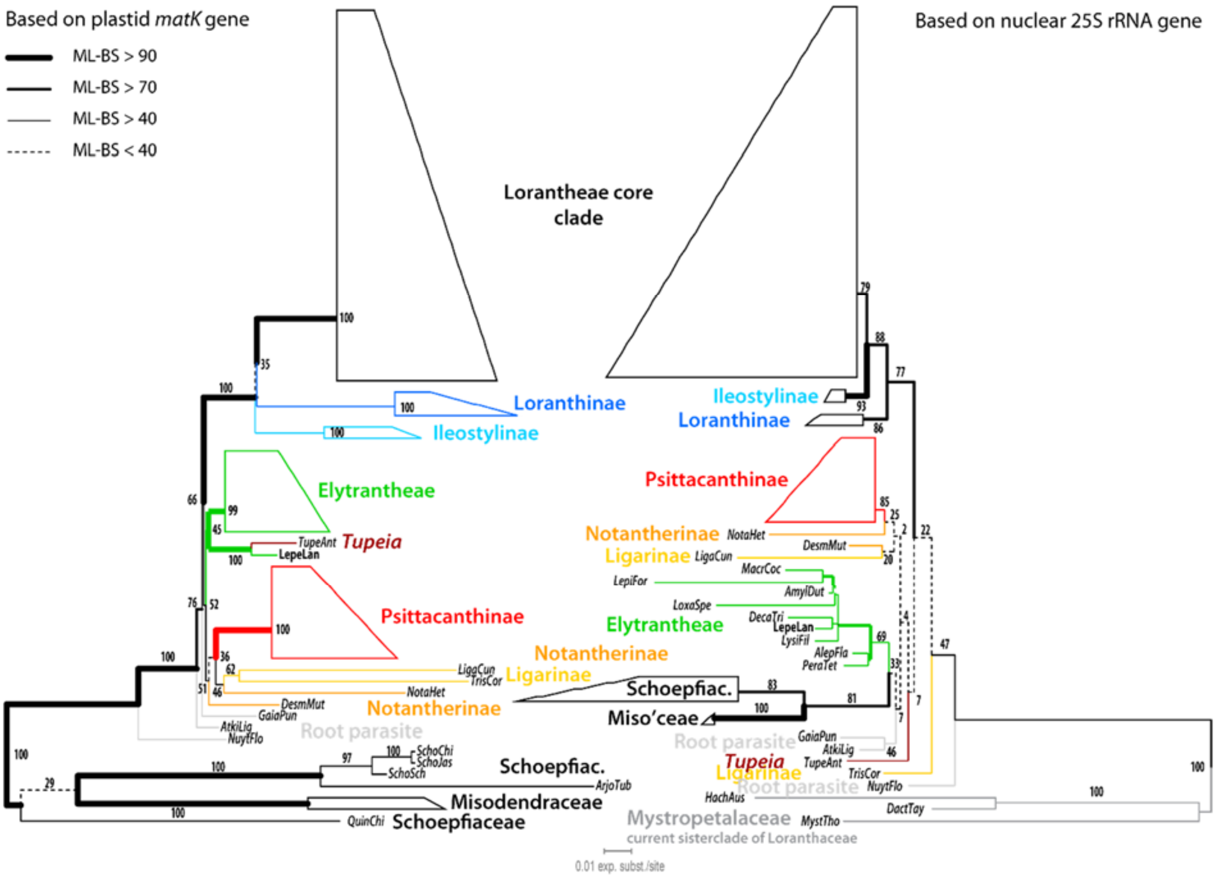

Figure S6-7: Maximum likelihood (ML) trees (terminal clades collapsed) inferred from the *matK* and 25S data for Loranthaceae and sistergroups included in the Su et al. matrix. Left, tree based on plastid *matK* data, rooted with Schoepfiaceae and Misodendraceae, the sisterclade of Loranthaceae and Mystropetalaceae. Note, the strong support for the root parasitic grade, boosting BS support values better(!) than those in Su et al.'s up-to-7-gene tree: adding the other gene regions and a full outgroup sample (all other Santalales) had a deteriorating effect for preferable – according anonymous reviewer #1 – in-Loranthaceae relationships (**Fig. S6-5**). *Tupeia* (see comment above) is unambiguously resolved as sister of *Lepeostegeres* as part of the low supported Elytrantheae clade. Right, tree based on the nuclear, more variable 25S rDNA data. The tree is rooted with the most divergent subtree, the Mystropetalaceae, the unambiguously inferred sisterclade of Loranthaceae in the study of Su et al. Note the extreme branch-lengths, the poor backbone support within Loranthaceae and the fact that the more distant (phylogenetically speaking) sistergroup is nested within the poorly resolved basis of the Loranthaceae subtree highlighting how particular single-gene trees can be misleading. The 25S data, equally divergent as the *matK* (same scale in both trees), fails to define an outgroup-inferred root. *Tupeia* is placed here unresolved close to the soft polytomy (short branches, no support) characterising the deep portion of the Loranthaceae subtree, whereas *Lepeostegeres* is nested within the Elytrantheae with moderately high support. Aside from these issues, there is no highly supported conflict between the *matK* and 25S data regarding inter-clade relationships, why we use the concatenated analysis (text-**Fig. 3**) as basis for our plots (text-**Figs 4, 12, 19, 33, 34**).

There are only two gene regions that unanimously support the topology shown in Su et al.'s tree and the Misodendraceae + Schoepfiaceae | Mystropetalaceae + Loranthaceae split; one is the new *RPB2* data, a single-copy nuclear gene. This is also the gene covering only five Loranthaceae (limited signal crossfire from the family with the most genera), while covering most of the sister groups. Maybe this is a gene region that has the highest potency for interfamily relationships in the Santalales, but for the Loranthaceae root question and subtree it may also be the one most severely affected by branching artefacts (**Fig. S6-2**). In an accordingly rooted tree, the root-tip distance of *Nuytsia* equals that of the Lorantheae and *Macrosolen*, which demonstrate the derivedness of *Nuytsia* also in this gene, hence, its vulnerability for ingroup-outgroup LBA.

One of the (still) most reliable gene for deep relationships in angiosperms is the plastid *rbcL* gene. Also here we can observed substantial (genetic and topological) distance between *Nuytsia* and the remainder of the Loranthaceae, which approaches the overall divergence within the rest of the family. Accordingly high is the support for a sistergroup (Misodendraceae + Schoepfiaceae) + *Nuytsia* vs. all other Loranthaceae split (BS_{ML} = 97). Relationships within the remaining Loranthaceae are inconspicuously unresolved for the 'basal' genera including *Gaiadendron* (cf. text-**Fig. 3** and **Fig. S6-5**), with better support only for the unambiguous ones (as far as sampled). The plastid genes *rbcL*, *matK*, and *accD* all show the same genetic distinctness regarding *Nuytsia* compared to all other Loranthaceae, which is the reason for the consistent recognition of this genetically distinct genus as sister to all other Loranthaceae and making it a good candidate for inevitable long-branch attraction such as *Amborella* has long been. But they do not agree on the placement of the other root parasites: the root parasite-aerial parasite split is only supported by *matK*. The *rbcL* data is undecided where to place *Atkinsonia*, but tends towards some association with *Nuytsia.* This is hardly surprising, both are root parasites, i.e. likely early diverged lineages, from the same geographic mega-region. The South American *Gaidendron,* however, is not joined with them but weakly linked to the Elytrantheae; the latter an Australasian clade with less discriminate, less-derived(?) sequences as illustrated by the often low support for their root (in particular, when using conservative gene regions such as the 18S, **Fig. S6-6**) and diminishing support for placing them with respect to the other major clades independent of the used gene region. Although there are only very few sequences, *accD*, the gene directly following the *rbcL* in the plastome, clearly rejects the root parasitic grade hypothesis with BS_{ML} = 87 for a split between Sistergroups + *Nuytsia* + Psittacanthinae | *Gaiadendron* + *Desmaria* + Elytrantheae (**Table S6-1; Fig. S6-7**). The Psittacanthinae are now the first diverging branch after *Nuytsia*, and the South American *Desmaria*, morphologically linked to *Notanthera*, is well supported (BS_{ML} = 85) as sister to the Australasian *Alepis* + *Peraxilla* (Elytrantheae clade A according Vidal-Russell & Nickrent 2008). Now if we would open our minds for possibilities rather than insisting on the root parasitic grade and the *Nuytsia*-root, it is an interesting co-incidence that both *rbcL* and *accD*, which are usually more conserved and reliable for deeper relationships that the faster evolving *matK*, put the South American *Gaidendron* in a clade (*accD*) or closer to (*rbcL*) *Desmaria* (a generally quite unique genus of South America; unique pollen, joined by Nickrent et al. (2010) with *Notanthera* based on morphology and some molecular evidence) and the Australasian Elytrantheae. Such a similarity does not necessarily mean that they have an inclusive common origin, but could evidence that these taxa isolated (diverged)

very close to the formation of the Loranthaceae as seen today. This brings us back to *Tupeia* and the 25S fragment of *Lepeostegeres.* Also here we see a genetic similarity that cannot be explained by a sister relationship according to anonymous reviewer #1 and – from a purely molecular-genetic point of view – I totally agree with him. All these similarities in more conserved portions of the genes[11], and the potentially false clades in single-gene (or other) trees, may simply reflect that these genera diverged close to the all-Loranthaceae root. But this also means that *Nuytsia* is *not* the sister to all other Loranthaceae.

The mitochondrial *matR*, a gene region normally so conservative that is does not resolve anything unless at very deep levels (see e.g. *matR* data included in Li et al.'s [2004] Fagales tree or Soltis et al.'s all-angiosperm matrices) finds support for Misodendraceae + Schoepfiaceae | Mystropetalaceae + Loranthaceae split also indicated by the *RPB2* gene and not challenged by the plastid data, because there is none on Mystropetalaceae; and not by the other two nuclear gene regions because they fail to agree with each other, so they can't oppose the signal from *matR* and *RPB2*. Thus, the unambiguous support of the according branches in the Su et al. tree and, since the signal comes from two different genomes, ensuring regarding the inter-family relationships: the Mystropetalaceae as sister group of the Loranthaceae. But this does not mean, that this family informs a good Loranthaceae root no matter which gene region is sampled (less variable like *RPB2* and *matR*, or more variable such as *matK*). Another analogy to the *RPB2* is that already the very limited *matR* data fails – if we belief the *matK*-informed basic topology – when it comes to the deepest relationships within the Loranthaceae. *Gaiadendron* is moderately high supported as sister of the Lorantheae (BS_{ML} = 78), whereas *Macrosolen* (Elytrantheae) and *Nuytsia* (again with a relatively long terminal branch) are unresolved as the 'base' of the Loranthaceae subtree, and the Loranthaceae clade itself finds an accordingly low support (BS_{ML} = 44). The latter means that *matR* has a problem to place the sistergroup subtree within the Loranthaceae tree, in other words, the few *matR* sequences already challenge the outgroup-inferred Loranthaceae root. Another piece of evidence indicating that the outgroup-defined root is problematic.

Knowing the pollen and with respect to what can been seen in the Su et al.'s data when analysed in more depth than usual (such as I have analysed our data, see **File S1**), one would be most curious to see the *RPB2* and *matR* sequences of *Tupeia, Phthirusa hutchisonii, Atkinsonia, Ligaria, Tristerix,* and *Notanthera.* Each one individual of five of these taxa were included in the Su et al matrix, but apparently the material was either unavailable or didn't work out for quick sequencing[12]. After all they were no priority for the study, which aimed at investigating the Balanophoraceae (even though none of their taxa cover all seven genes either) and not to provide a better idea about the phylogeny of Loranthaceae. The latter would however be very much needed, but I fear as long as people are happy with the trees and roots they have decide on what is looked at and researched in more detail, there will be little progress on that front.

[11] Somebody really interested in Loranthanceae phylogeny should think about running an inference with the rDNA that takes into account the potential secondary structure of the transcript.
[12] I noticed large, sequencing(?) gaps in the *matR* data and some odd accumulation of random mutations, same in *RPB2*, both usually signs for sequencing problems these days; and one would be very well advised to sequence fresher material of *Loranthus* to verify those very distinct *matR* sequences.

Some further experiments regarding the outgroup-inferred root

Let's do the classic test for LBA and remove *Nuytsia* from the dataset. ML comes now up with a tree that looks very different. Now the Australasian aerial parasitic Elytrantheae are the first branch within the Loranthaceae, followed the Psittacanthinae + Gaiadendreae (the tribe including the other two root parasites, *Atkinsonia* and *Gaiadendron*) sister to a cosmopolitan clade comprising the isolated South American genera + *Tupeia* as sister to the Lorantheae (**Fig. S6-8**). This tree demonstrates two problems anonymous reviewer #1 (and, partly, #3) are unaware of: a) the dependency of high support for the root parasitic grade on the signal from *Nuytsia*, such dependency on a single taxon is a direct indication for branching artefacts; and b) that it is not sufficient to just write <50 on a branch and that it should be obligatory to properly document bootstrapping results when support is not unambiguous. The support of the new topology is extremely low for the critical branches. A look at the bootstrap sample reveals that the alternative of a root parasitic grade is still alive, although (much) weakened with a BS_{ML} for the split *Atkinsonia* + sistergroups | remainder of Loranthaceae of 47 (partly incompatible with the alternative *matK* signal indicating a Gaiadendreae clade), and for root parasites + sistergroups | aerial parasitic Loranthaceae of 30 (bootstrap support network in **Fig. S6-8**). The outgroup subtree could probably be moved to any part of the Loranthaceae subtree that is generally characterised by indiscriminate supports linked to very short branch-lengths, where we (still) find *Tupeia*, *Desmaria* and the Ligarinae in addition to the roots of the well-supported clades Psittacanthinae, Elytrantheae, and Lorantheae.

What about the effect of missing data? The sisterclade of the Loranthaceae, the Mystropetalaceae only covers four of the seven gene regions in the concatenated data set. There is no plastid data for this clade. An analysis of the nuclear-mitochondrial subset, again without *Nuytsia*, produces only some support for the root placement seen in **Fig. S6-8** (Elytrantheae sister to all other Loranthaceae; $BS_{ML} = 44$), all other topological alternatives including an *Aktinsonia* or *Gaiadendron* root and, hence, the root parasitic grade, find no support ($BS_{ML} < 10$). Just by removing *Nuytsia* and limiting the data set to data actually covered by all sistergroups, we cripple that data's capacity to resolve the initial relationships in Loranthaceae, the well-established root parasitic grade, and the position of the Loranthaceae root.

A 7-gene analysis without *Nuytsia* and the sistergroups, the support for the Gaiadendreae increases again ($BS_{ML} = 77$, *Atkinsonia* and *Gaiadendron* may actually represent sister taxa; Nickrent et al. 2010), but not so for the other deepest divergences. In contrast to analysing datasets with *Nuytsia*, including or excluding outgroups has a direct effect on the support values for critical splits when *Nuytsia* is not included. Altogether, this is a very strong indication that the outgroup-inferred *Nuytsia* root is an artefact triggered by the fact that *Nuytsia* is simply a very distinct Loranthaceae and of largely uncertain affinity regarding the signal from most sequenced gene regions, but a *matK* that links it to the Gaiadendreae (**Fig. S6-4; Table S6-1**). Because of this distinctness, any non-Loranthaceae, which will be much more distinct to any Loranthaceae, will be inevitably drawn to *Nuytsia*, and the so inferred root changes a potential root parasitic clade into a highly supported (**Fig. S6-5**) or modestly supported (Su et al., fig. 1B) root parasitic grade.

This can only be partly compensated by adding a very large outgroup sample (Su et al. 2015, fig. 1B), which then finds lower support for the critical and biased (wrong) split.

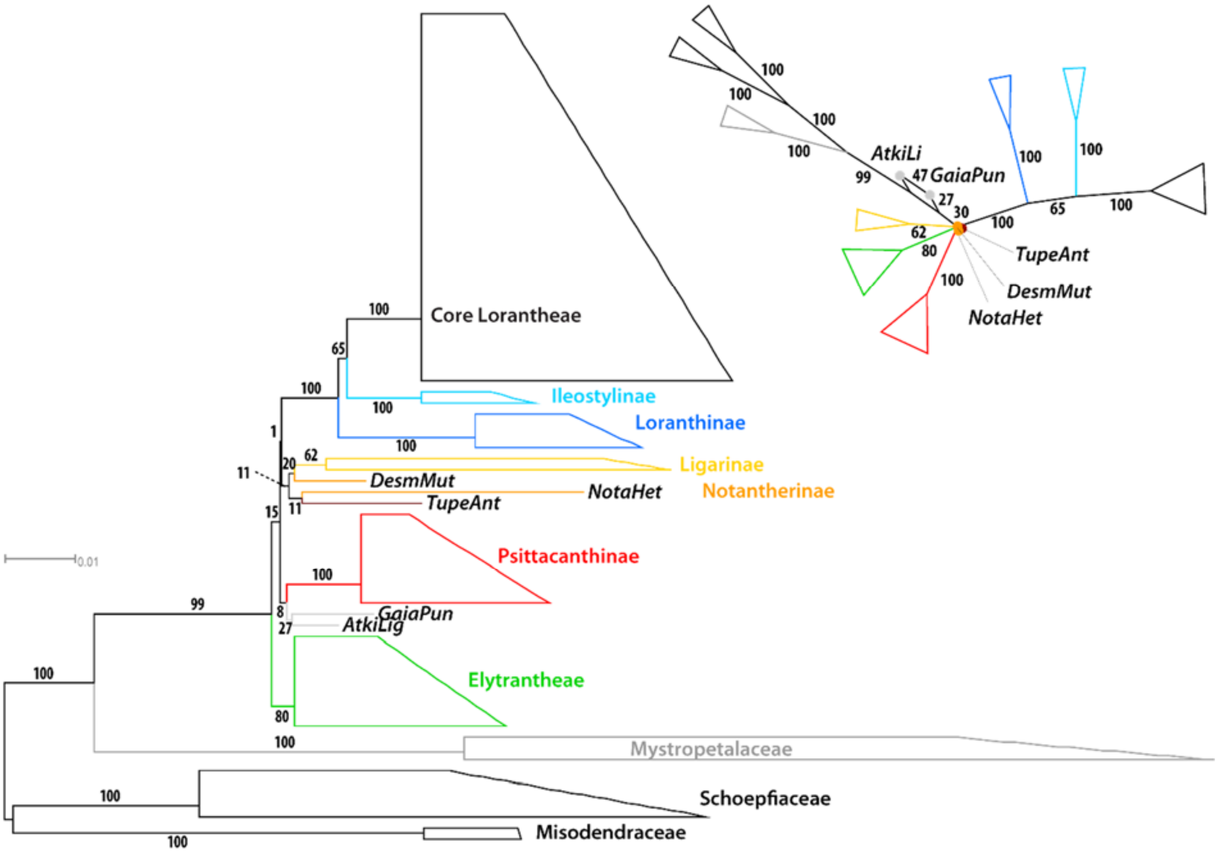

Figure S6-8: Maximum likelihood (ML) tree inferred based on Su et al.'s up-to-7-gene data on Loranthaceae and their sistergroups, but *Nuytsia* excluded from analysis. ML-BS support (BS_{ML}) is indicated along branches, terminal clades are represented by proportional (regarding number of taxa and minimal and maximal root-tip distance) parallelograms. On the upper right a bootstrap support network based on the same analysis showing that the tree is unrepresentative regarding the deepest splits, and the placement of the outgroup-inferred root. Note, however the substantial decrease in support for critical branches: The support of the *Atkinsonia* + sistergroups | remaining Loranthaceae split decreased from BS_{ML} = 62 to BS_{ML} = 47 and that for the root parasites + sistergroups | aerial parasitic Loranthaceae from BS_{ML} = 60 to BS_{ML} = 30. If *Atkinsonia* is excluded, too, the support for the latter becomes non-existent and the seen – in the tree – alternative of Elytrantheae + sistergroups | remaining Loranthacaeae split increases from BS_{ML} =15 (see tree) to BS_{ML} = 39 (weak but best supported alternative, any alternative placement with BS_{ML} < 20).

On the other hand, no matter what we do with the data set, we get stuck in a soft polytomy, which includes the genetically but not taxonomically diverse root parasites and South American genera of the Ligarinae and Notantherinae, *Tupeia* from New Zealand with its atypical, non-Loranthaceae pollen, and the roots of the diverse and more or well differentiated Loranthaceae clades (Elytrantheae, Lorantheae, and Psittacanthinae). Given the biology of the Loranthaceae and modern distribution, it is not a bad hypothesis that the group underwent a fast ancient radiation involving all these lineages, hence, that the actual root lies somewhere

in this soft polytomy. So the pollen-based root is definitely closer to the reality than the outgroup-inferred *Nuytsia* root. Rejecting the pollen evidence for an alternative root as "absurd" (anonymous reviewer #1) or "unnecessary" (anonymous reviewer #3) lacks any firm molecular basis.

Concluding remark—I here explored a subset of the Su et al. data including the Loranthaceae and their sistergroups using the same approach I used on our data to investigate why some branches in their tree have high support and others not. I added some experiments in order to test the trust anonymous reviewer #1 and #3 put into the *Nuytsia*-root for the Loranthaceae and the topological scenario of a root parasitic grade[13].

From these analysis, and the differences seen between the Loranthaceae subtrees in Su et al.'s figs 1B, S6 and S7, it is clear that their matrix has the same data and signal issues than ours. We will stick here to my data set as it is data-wise and signal-wise more comprehensive than the Su et al. data and aligns with my personal data and publishing ethics in contrast to the insufficient documentation provided by Su et al. (2015) for this complex data set and group. When it comes to reflect actual genetic differentiation within Loranthaceae, neither the Su et al. data nor their inference is superior to our study[14]. It is, however, more problematic as it reports relatively, probably too high support for conflicted branches between the combined gene regions. A branch supported by signal from a single of the seven included gene regions and not supported, sometimes even rejected by others, can result in moderate BS support and sometimes very high PP based on Su et al.'s matrix. The Su et al. tree shows why – in complex data situations – multi-method inferences are a waste of time, particular when using BI in addition to ML/BS analyses as often asked for by anonymous reviewers (here: anonymous reviewer #3, introduced as expert on phylogenetics). Any branch with non-unambiguous support should be free for any sort of discussion and investigated further. It is also not re-assuring that the same gene, *matK,* that informs most of the critical parts of the topology by Su et al. (such as the root parasitic grade), is also the one that messes up relationships otherwise clear. Regarding the latter, I am positive that this problem will decrease as soon as complete *matK* sequences become available: the proportion of gaps and completely undetermined characters in Su et al.'s Loranthaceae *matK* sample is 21% (and 55% for their concatenated data set).

The outgroup-inferred Loranthaceae root, the *Nuytsia* root, should be met with great scepticism until these discrepancies can be explained or vanish with the filling of the still huge data gaps. The currently available data indicates that the outgroup-inferred root is a topological artefact, mainly triggered by the distinctness of *Nuytsia*, which is, however, the only Loranthaceae that has been sequenced for all seven genes used by Su et al. The missing data poses a problem (**Fig. S6-8**), and one rather should use trees with less genes and taxa but

[13] A well-meaning tip: if you see a tree where the branch directly after the root branch has non-unambiguous support, don't take this root for granted. Particularly not if the sisterclade shows intra-clade diversity which shadows the one in your ingroup by the factor 3 or more. Ladder-like grades following such roots are also good indicators for a wrong outgroup-inferred root. How likely is it that for several divergences in a row one lineage survives until today and only the other one radiates? Do we really believe nature dices with a binary dice?

[14] This may be due to the fact that all data of the better-sampled regions have been considered when computing the genus consensus sequences for our data set, except for the artificially chimeric *matK* sequence of *Aetanthus*.

complete data coverage for testing topological alternatives (our **File S1**) for putting forward molecular-based phylogenetic hypothesis for the Loranthaceae.

References

Delsuc F, Brinkmann H, Philippe H. 2005. Phylogenomics and the reconstruction of the tree of live. Nature Reviews Genetics 6: 361-375.

Kuijt J. 2011. Pulling the skeleton out of the closet: resurrection of *Phthirusa* sensu Martius and consequent revival of *Passovia* (Loranthaceae). Plant Diversity and Evolution 129: 159–211.

Li R-Q, Chen Z-D, Lu A-M, Soltis DE, Soltis PS, Manos PS. 2004. Phylogenetic relationships in Fagales based on DNA sequences from three genomes. International Journal of Plant Sciences 165: 311-324.

Nickrent DL, Malécot V, Vidal-Russell R, Der JP. 2010. A revised classification of Santalales. Taxon 9: 538–558.

Renner SS, Grimm GW, Schneeweiss GM, Stuessy TF, Ricklefs RE. 2008. Rooting and dating maples (*Acer*) with an uncorrelated-rates molecular clock: Implications for North American/Asian disjunctions. Systematic Biology 57: 795-808.

Soltis DE, Smith SA, Cellinese N, Wurdack KJ, Tank DC, Brockington SF, Refulio-Rodriguez NF, Walker JB, Moore MJ, Carlsward BS, Bell CD, Latvis M, Crawley S, Black C, Diouf D, Xi Z, Rushworth CA, Gitzendanner MA, Sytsma KJ, Qiu YL, Hilu KW, Davis CC, Sanderson MJ, Beaman RS, Olmstead RG, Judd WS, Donoghue MJ, Soltis PS. 2011. Angiosperm phylogeny: 17 genes, 640 taxa. American Journal of Botany 98: 704–730.

Su H-J, Hu J-M, Anderson FE, Der JP, Nickrent DL. 2015. Phylogenetic relationships of Santalales with insights into the origins of holoparasitic Balanophoraceae. Taxon 64: 491–506.

Vidal-Russell R, Nickrent DL. 2007. The biogeographic history of Loranthaceae. Darwiniana 45: 52–54.

Vidal-Russell R, Nickrent DL. 2008. Evolutionary relationships in the showy mistletoe family (Loranthaceae). American Journal of Botany 95: 1015–1029.
